# Supplementary material for: Identifying potential biases in code sequences in primary care electronic healthcare records: a retrospective cohort study of the determinants of code frequency
Source: BMJ Open. 2023 Sep 27;13(9):e072884. doi: 10.1136/bmjopen-2023-072884 (PMC10537851; doi:10.1136/bmjopen-2023-072884)
Supplement: Supplementary data [file bmjopen-2023-072884supp001.pdf]

Appendix

Identifying potential biases in code sequences in primary care electronic healthcare records: a retrospective cohort study of the determinants of code frequency

Beaney T[1,2] (0000-0001-9709-7264), Clarke J[2], Salman D [1,3], Woodcock T[1], Majeed A[1], Barahona M[2], Aylin P[1]

1. Department of Primary Care and Public Health, Imperial College London, London, W6 8RP, United Kingdom
2. Centre for Mathematics of Precision Healthcare, Department of Mathematics, Imperial College London, London, SW7 2AZ, United Kingdom
3. MSk Lab, Department of Surgery and Cancer, Faculty of Medicine, Imperial College London, London, UK

Corresponding Author:
Dr Thomas Beaney
Department of Primary Care and Public Health, Imperial College London, London, W6 8RP, United Kingdom
Email: thomas.beaney@imperial.ac.uk

Beaney T, *et al. BMJ Open* 2023; 13:e072884. doi: 10.1136/bmjopen-2023-072884

Patients were included with continuous registration dates between 1st January 2014 and 31st December 2020. The 1st January 2014 was chosen to allow for a full one year of registration at a GP practice prior to follow-up, to reduce the potential impact of bias from newly registered patients having pre-existing conditions coded for the first time at their new practice. The end date of 31st December 2020 was chosen to provide at least one full year of follow-up for conditions newly diagnosed in 2019. Patients were followed up until the earliest date of death, deregistration and latest date of data extraction from their practice, if after 31st December 2020. The earliest possible censoring date for a patient was 1st January 2021 and the last date of follow-up for a patient was 21st March 2022.

Chronic conditions

Diseases were mapped using code lists developed for the CALIBER study, and adapted for use in multimorbidity in CPRD Aurum.[1,2] We reviewed the codes in these lists, and made amendments to the code lists for diabetes. The 'other/unspecified' diabetes code list contained codes specific to both Type 1 and Type 2 diabetes, and we removed these to ensure the list included only codes where a more specific Type 1 or Type 2 diagnosis was not stated. We added chronic primary pain to the set of included conditions and created a new code list. Previous studies of multimorbidity in primary care settings have found a high prevalence and burden of chronic pain.[3,4] However, in order to avoid double counting of pain related to another chronic condition included, we excluded secondary causes, and included only primary pain conditions.

Assignment to QOF

Diseases were classified as included or not included in QOF by two clinicians with experience working as GPs: TB and DS. The first QOF year in 2004/2005 included eleven diseases, with new conditions added in subsequent years.[5] Rheumatoid arthritis was added to QOF in 2013/2014, but there were no subsequent additions of any of the diseases included in this study.[6] However, hypothyroidism was included in QOF from its start until 2014/15 when it was removed.[7] The thyroid disease category from CALIBER included codes for both hypothyroidism and hyperthyroidism. We therefore excluded the thyroid disease category from comparisons of QOF to avoid any carry-over effect from prior inclusion in QOF, and dilution from non-hypothyroid conditions. The following QOF conditions from 2014/15 to 2019/20 were included:

1. Coronary Heart Disease
2. Left Ventricular Dysfunction / Heart Failure (from 2006)
3. Stroke (and TIA from 2006)
4. Hypertension
5. Diabetes
6. COPD
7. Epilepsy
8. Cancer
9. Mental Health
10. Asthma
11. Dementia
12. Depression
13. CKD
14. Atrial fibrillation
15. Obesity
16. Learning disabilities
17. Palliative care
18. Smoking
19. Cardio-vascular disease (primary prevention)
20. Peripheral Arterial Disease (PAD)
21. Osteoporosis
22. Rheumatoid arthritis

For analyses of counts per calendar year, the total counts of disease codes were calculated for the first and second year from diagnosis. Counts were stratified according to whether a condition was included in QOF. A patient was included for a given calendar year if they had at least one QOF or non-QOF condition diagnosed in that year, as shown in Table A1.

Table A1: example of the stratification of condition and calendar year for each newly diagnosed condition for three hypothetical patients

Patient	Age	Condition	Calendar year	Count in year one	Count in year two
1	67	QOF	2015	0	0
1	68	QOF	2016	2	0
1	70	QOF	2018	4	2
1	67	Non-QOF	2015	1	1
2	28	Non-QOF	2019	1	2
3	52	QOF	2017	5	4
3	52	Non-QOF	2017	2	2

Beaney T, *et al*. *BMJ Open* 2023; 13:e072884. doi: 10.1136/bmjopen-2023-072884

Statistical analyses

Mixed effects negative binomial models were constructed. We considered use of a zero-inflated model, but coefficients from the logit and negative binomial components of the model were similar, and so in the interests of interpretable findings, the more parsimonious negative binomial model was selected.

Equation for the mixed effects negative binomial regression model, including fixed effects for calendar year and GP practice and random effects for patient:

$$log(y_{i,j}) = \beta_0 + \beta_1 age_{i,j} + \beta_2 gender_{i,j} + \beta_3 ethnicity_{i,j} + \beta_4 IMD_{i.j} + \beta_5 year_{i.j} + \beta_6 GP_{i.j} + u_j$$

where *i* represents QOF or non-QOF conditions newly diagnosed in patient *j* and $y_{i,j}$ is the count of codes in the given year.

Beaney T, *et al. BMJ Open* 2023; 13:e072884. doi: 10.1136/bmjopen-2023-072884

A2: Frequency and percentage of pre-existing diseases (as of 1st January 2015) for all 3,113,724 eligible patients

Pre-existing disease	Frequency	Percentage
Hypertension	751009	24.12%
Enthesopathy and synovial disorder	736087	23.64%
Dermatitis	710945	22.83%
Depression	568871	18.27%
Anxiety disorders	507406	16.30%
Allergic and chronic rhinitis	477053	15.32%
Asthma	456335	14.66%
Osteoarthritis (excl spine)	444668	14.28%
Gastro-oesophageal reflux disease	301839	9.69%
Obesity	294916	9.47%
Diabetes Mellitus: other or not specified	285681	9.17%
Hearing loss	279470	8.98%
Migraine	270415	8.68%
Type 2 Diabetes Mellitus	255578	8.21%
Irritable bowel syndrome	246744	7.92%
Abdominal Hernia	237968	7.64%
Acne	225183	7.23%
Chronic sinusitis	212496	6.82%
Thyroid Disease	204639	6.57%
Spondylosis	181722	5.84%
Gastritis and duodenitis	181668	5.83%
Cataract	160486	5.15%
Chronic Kidney Disease	158134	5.08%
Coronary Heart Disease (not otherwise specified)	144806	4.65%
Seborrheic dermatitis	143168	4.60%
Urinary Incontinence	137919	4.43%
Alcohol Misuse	132717	4.26%
Psoriasis	132694	4.26%
Diaphragmatic hernia	131539	4.22%
Diverticular Disease	131332	4.22%
Tinnitus	123308	3.96%
Gout	120568	3.87%
Stable Angina	120309	3.86%
Intervertebral disc disorders	117787	3.78%
Anaemia: other	116859	3.75%
Diabetic Eye Disease	102901	3.30%
Rosacea	96511	3.10%
Dysmenorrhoea	94881	3.05%

Beaney T, *et al. BMJ Open* 2023; 13:e072884. doi: 10.1136/bmjopen-2023-072884

Benign Prostatic Hyperplasia	92304	2.96%
Osteoporosis	91850	2.95%
Primary Malignancy: Skin	89500	2.87%
COPD	84482	2.71%
Atrial Fibrillation	80645	2.59%
Peripheral Neuropathy	77117	2.48%
Chronic Fatigue Syndrome	67489	2.17%
Myocardial Infarction	67215	2.16%
Vitamin B12 deficiency anaemia	64015	2.06%
Glaucoma	58081	1.87%
Epilepsy	53058	1.70%
Stroke: not otherwise specified	50614	1.63%
Substance Misuse	50251	1.61%
Primary Malignancy: Breast	49737	1.60%
Venous thromboembolic disease (Excl PE)	47013	1.51%
Transient ischaemic attack	44616	1.43%
Fibromatosis	42701	1.37%
Neuropathic Bladder	42008	1.35%
Raynaud's syndrome	38879	1.25%
Endometriosis	37868	1.22%
Sleep apnoea	35743	1.15%
Heart failure	35364	1.14%
Peripheral Vascular Disease	32852	1.06%
Rheumatoid Arthritis	32070	1.03%
Macular degeneration	30761	0.99%
Chronic primary pain	29506	0.95%
Anterior and Intermediate Uveitis	28838	0.93%
Visual impairment and blindness	28372	0.91%
Polymyalgia Rheumatica	27447	0.88%
Primary Malignancy: Prostate	26288	0.84%
Ulcerative colitis	22236	0.71%
Nonrheumatic mitral valve disorders	20980	0.67%
Spinal stenosis	20820	0.67%
Nonrheumatic aortic valve disorders	20695	0.66%
Schizophrenia	20394	0.65%
Type 1 Diabetes Mellitus	19978	0.64%
Unstable Angina	18925	0.61%
Trigeminal neuralgia	18854	0.61%
Scleritis and episcleritis	18830	0.60%
Fatty Liver	18774	0.60%
Barrett's oesophagus	18152	0.58%
Supraventricular tachycardia	18128	0.58%
Intellectual disability	18073	0.58%
Pancreatitis	18043	0.58%

Beaney T, *et al. BMJ Open* 2023; 13:e072884. doi: 10.1136/bmjopen-2023-072884

Bronchiectasis	18006	0.58%
Primary Malignancy: Melanoma	17594	0.57%
Personality disorders	17448	0.56%
Alopecia areata	17111	0.55%
Primary Malignancy: Bowel	16746	0.54%
Obsessive-compulsive disorder	15553	0.50%
Polycystic ovarian syndrome	14606	0.47%
Crohn's disease	14445	0.46%
Folate deficiency anaemia	13853	0.44%
Retinal vascular occlusions	13829	0.44%
Obstructive and reflux uropathy	13725	0.44%
Ischaemic stroke	13451	0.43%
Hidradenitis suppurativa	13305	0.43%
Vitiligo	13218	0.42%
Meniere's Disease	13192	0.42%
Bipolar affective disorder and mania	12856	0.41%
Coeliac disease	12625	0.41%
Diabetic Neuropathy	12517	0.40%
Chronic viral hepatitis	11885	0.38%
Thrombophilia	11527	0.37%
Psoriatic Arthritis	11201	0.36%
Eating Disorders	11171	0.36%
Dementia	10297	0.33%
Spondylolisthesis	10229	0.33%
Secondary Thrombocytopaenia	9800	0.31%
Congenital Septal Defect	9203	0.30%
Sarcoidosis	9090	0.29%
Multiple sclerosis	9070	0.29%
Benign essential tremor	9008	0.29%
Right bundle branch block combinations	8160	0.26%
Primary Malignancy: Bladder	8066	0.26%
Primary Malignancy: other	8021	0.26%
Glomerulonephritis	7950	0.26%
Autism and Asperger's syndrome	7920	0.25%
Non-Hodgkin Lymphoma	7579	0.24%
Hyperparathyroidism	7437	0.24%
Pleural effusion	7368	0.24%
Hyperkinetic disorders	7056	0.23%
Ankylosing spondylitis	7044	0.23%
Lupus Erythematosus	6976	0.22%
Cirrhosis	6768	0.22%
Alcoholic liver disease	6621	0.21%
Left bundle branch block	6512	0.21%
Subarachnoid haemorrhage	6158	0.20%

Beaney T, *et al. BMJ Open* 2023; 13:e072884. doi: 10.1136/bmjopen-2023-072884

Collapsed vertebra	6082	0.20%
Autonomic Neuropathy	5496	0.18%
Cardiomyopathy: other	5465	0.18%
Parkinson's disease	5333	0.17%
Leukaemia	5243	0.17%
Giant Cell arteritis	5225	0.17%
Hyposplenism	4737	0.15%
HIV	4697	0.15%
Endometrial hyperplasia and hypertrophy	4655	0.15%
Primary Malignancy: Uterus	4589	0.15%
Sjogren's Syndrome	4559	0.15%
Spina bifida	4427	0.14%
Cerebral Palsy	4011	0.13%
Primary Thrombocytopaenia	3979	0.13%
Pleural plaque	3972	0.13%
Abdominal Aortic Aneurysm	3931	0.13%
Atrioventricular blocks	3920	0.13%
Chronic Cystitis	3892	0.12%
Intracerebral haemorrhage	3815	0.12%
Primary Malignancy: Ovary	3689	0.12%
Primary Malignancy: Cervix	3500	0.11%
Asbestosis	3358	0.11%
Other haemolytic anaemias	3152	0.10%
Primary Malignancy: Testis	3133	0.10%
Thalassaemia	3055	0.10%
Hypertrophic Nasal Turbinates	3022	0.10%
Primary Malignancy: Kidney	2988	0.10%
Polycythaemia vera	2864	0.09%
Primary Malignancy: Oropharyngeal	2809	0.09%
Autoimmune liver disease	2792	0.09%
Ventricular tachycardia	2720	0.09%
Secondary polycythaemia	2625	0.08%
Posterior Uveitis	2540	0.08%
Pulmonary Fibrosis	2523	0.08%
Hodgkin Lymphoma	2384	0.08%
Hypersplenism	2362	0.08%
Dilated cardiomyopathy	2359	0.08%
Primary Malignancy: Lung	2244	0.07%
Primary Malignancy: Thyroid	2172	0.07%
Rheumatic Valve Disorder	2034	0.07%
Secondary Malignancy_other	1975	0.06%
Down's syndrome	1928	0.06%
Multiple valve disorder	1834	0.06%
Idiopathic Intracranial Hypertension	1823	0.06%

Hypertrophic Cardiomyopathy	1779	0.06%
Oesophageal varices	1716	0.06%
Plasma Cell Malignancy	1610	0.05%
Scleroderma	1566	0.05%
Pericardial Effusion	1509	0.05%
Myasthenia gravis	1407	0.05%
Primary pulmonary hypertension	1345	0.04%
Sick sinus syndrome	1231	0.04%
Aplastic anaemias	1172	0.04%
Primary Malignancy: Brain	1131	0.04%
Immunodeficiencies	1071	0.03%
Cystic Fibrosis	985	0.03%
Primary Malignancy: Oesophageal	955	0.03%
Myelodysplastic Syndrome	927	0.03%
Portal hypertension	919	0.03%
Sickle Cell Disease	887	0.03%
Secondary pulmonary hypertension	824	0.03%
Angiodysplasia of colon	777	0.02%
Primary Malignancy: Bone	741	0.02%
Primary Malignancy: Stomach	694	0.02%
Hepatic failure	632	0.02%
Secondary Malignancy: Lymph Nodes	565	0.02%
Secondary Malignancy: Liver	491	0.02%
Tubulo-interstitial nephritis	365	0.01%
Motor neurone disease	347	0.01%
Primary Malignancy: Pancreas	302	0.01%
Enteropathic arthropathy	291	0.01%
Primary Malignancy: Liver	233	0.01%
Secondary Malignancy: Lung	223	0.01%
Secondary Malignancy: Bone	187	0.01%
Primary Malignancy: Biliary Tract	129	<0.01%
Secondary Malignancy: Brain	50	<0.01%
Secondary Malignancy: Peritoneum	24	<0.01%
Secondary Malignancy: Bowel	11	<0.01%
Secondary Malignancy: Adrenal Gland	*	<0.01%
Primary Malignancy: Multiple Sites	*	<0.01%
Primary Malignancy: Mesothelioma	*	<0.01%
Secondary Malignancy: Pleura	*	<0.01%

* diseases with frequency <10 suppressed as small counts

Beaney T, *et al. BMJ Open* 2023; 13:e072884. doi: 10.1136/bmjopen-2023-072884

Table A3: characteristics of the 3,060,391 ineligible patients with no incident diseases over the study period

Patient characteristic	Total	Percent
**Age (years)**		
18-40	1476341	48.2%
40-49	689779	22.5%
50-59	435517	14.2%
60-69	291093	9.5%
70-79	129375	4.2%
80+	38286	1.3%
**Gender**		
Female	1357049	44.3%
Male	1703284	55.7%
Indeterminate	58	0.0%
**Total**	**3060391**	

Table A4: distribution of yearly codes over the whole follow-up period for each condition, ordered by median

Disease	5th centile	Median	95th centile	Mean	Standard deviation
Diabetes Mellitus_other or not specified	0.00	2.99	6.88	3.08	2.22
Polymyalgia Rheumatica	0.00	1.05	6.32	1.82	2.29
Motor neurone disease	0.00	0.95	12.15	2.86	5.41
Dementia	0.00	0.93	4.36	1.39	1.80
Type 2 Diabetes Mellitus	0.00	0.89	4.59	1.41	1.73
Type 1 Diabetes Mellitus	0.00	0.88	6.31	1.71	2.41
Depression	0.00	0.83	4.54	1.36	1.76
COPD	0.00	0.77	3.77	1.17	1.43
Heart failure	0.00	0.73	5.48	1.46	2.21
Rheumatoid Arthritis	0.00	0.70	5.50	1.43	2.23
Primary Malignancy_Mesothelioma	0.00	0.67	9.16	1.78	3.18
Primary Malignancy_Pancreas	0.00	0.67	13.41	2.63	5.12
Primary Malignancy_Brain	0.00	0.66	10.60	2.15	3.96
Primary Malignancy_Oesophageal	0.00	0.64	10.86	2.44	4.95
Myasthenia gravis	0.00	0.62	5.61	1.48	2.66
Multiple sclerosis	0.00	0.59	5.63	1.40	2.41
Parkinson's disease	0.00	0.59	4.52	1.20	1.77
Vitamin B12 deficiency anaemia	0.00	0.56	4.60	1.24	1.67
Bipolar affective disorder and mania	0.00	0.56	4.99	1.30	2.15
Plasma Cell Malignancy	0.00	0.54	10.32	2.15	4.67
Hypertension	0.00	0.54	2.95	0.88	1.12
Atrial Fibrillation	0.00	0.51	3.47	0.97	1.47
Primary Malignancy_Prostate	0.00	0.51	6.11	1.46	2.48
Intellectual disability	0.00	0.49	5.19	1.47	1.91
Primary Malignancy_Lung	0.00	0.45	8.17	1.73	3.55
Primary Malignancy_Biliary Tract	0.00	0.45	8.96	1.89	4.73
Giant Cell arteritis	0.00	0.44	5.73	1.36	2.47
Crohn's disease	0.00	0.42	5.41	1.24	2.32
Primary Malignancy_Breast	0.00	0.39	5.25	1.21	2.47
Hodgkin Lymphoma	0.00	0.38	5.41	1.24	2.55
Ulcerative colitis	0.00	0.38	4.27	1.00	1.87
Primary Malignancy_Oropharyngeal	0.00	0.37	6.84	1.44	2.95
Non-Hodgkin Lymphoma	0.00	0.37	5.52	1.22	2.53
Leukaemia	0.00	0.37	5.19	1.17	2.58
Secondary Malignancy_Brain	0.00	0.37	7.68	1.45	2.74
Stroke_not otherwise specified	0.00	0.34	2.11	0.59	0.89
Idiopathic Intracranial Hypertension	0.00	0.34	3.81	0.92	1.76
Thyroid Disease	0.00	0.33	2.56	0.68	1.16
Asthma	0.00	0.32	2.33	0.63	0.99
Primary Malignancy_Stomach	0.00	0.32	6.93	1.45	3.30
Chronic primary pain	0.00	0.32	3.23	0.79	1.34
Coronary Heart Disease (not otherwise specified)	0.00	0.31	2.02	0.56	0.85
Epilepsy	0.00	0.31	3.66	0.92	1.95
Psoriatic Arthritis	0.00	0.30	3.68	0.87	1.63

Chronic Fatigue Syndrome	0.00	0.29	3.22	0.76	1.31
Primary Malignancy_Bowel	0.00	0.29	5.25	1.15	2.88
Anxiety disorders	0.00	0.29	2.99	0.73	1.29
Primary Malignancy_Thyroid	0.00	0.28	4.05	0.88	1.76
Personality disorders	0.00	0.28	4.35	0.99	2.05
Schizophrenia	0.00	0.27	3.36	0.78	1.52
Primary Malignancy_Cervix	0.00	0.27	5.26	1.17	2.77
Autoimmune liver disease	0.00	0.26	3.63	0.85	1.82
Myelodysplastic Syndrome	0.00	0.26	4.88	1.15	2.95
Bronchiectasis	0.00	0.24	3.03	0.70	1.31
Hyperkinetic disorders	0.00	0.24	3.11	0.72	1.34
Primary Malignancy_Ovary	0.00	0.24	6.15	1.24	2.87
Primary Malignancy_Liver	0.00	0.23	3.64	0.95	2.99
Coeliac disease	0.00	0.23	2.13	0.52	0.85
Lupus Erythematosus	0.00	0.22	3.52	0.83	1.87
Myocardial Infarction	0.00	0.21	2.44	0.58	1.04
Primary Malignancy_Bone	0.00	0.21	4.03	0.97	3.29
Secondary Malignancy_other	0.00	0.21	5.92	1.18	2.65
Peripheral Vascular Disease	0.00	0.20	2.73	0.75	2.53
Ankylosing spondylitis	0.00	0.20	3.00	0.69	1.47
Primary Malignancy_Bladder	0.00	0.20	4.38	0.90	2.05
Primary Malignancy_Testis	0.00	0.20	3.58	0.81	1.50
Sarcoidosis	0.00	0.19	3.36	0.72	1.53
Abdominal Hernia	0.00	0.19	1.55	0.40	0.68
Secondary Malignancy_Peritoneum	0.00	0.19	4.21	1.30	3.31
Scleroderma	0.00	0.19	3.00	0.71	1.88
Primary Malignancy_Melanoma	0.00	0.18	3.06	0.67	1.71
Gout	0.00	0.17	1.74	0.43	0.73
Barrett's oesophagus	0.00	0.16	1.40	0.35	0.57
Glomerulonephritis	0.00	0.16	3.26	0.74	1.69
Osteoporosis	0.00	0.15	1.52	0.38	0.65
Primary Malignancy_Uterus	0.00	0.15	3.90	0.81	2.16
Cirrhosis	0.00	0.15	2.88	0.63	1.40
Diabetic Eye Disease	0.00	0.15	1.61	0.40	0.68
Intracerebral haemorrhage	0.00	0.15	2.58	0.56	1.10
Primary Malignancy_Kidney	0.00	0.14	2.93	0.66	1.67
Dilated cardiomyopathy	0.00	0.14	1.99	0.46	0.93
Eating Disorders	0.00	0.14	4.03	0.84	2.38
Abdominal Aortic Aneurysm	0.00	0.00	1.35	0.26	0.58
Acne	0.00	0.00	1.26	0.30	0.50
Alcohol Misuse	0.00	0.00	0.94	0.20	0.66
Alcoholic liver disease	0.00	0.00	1.90	0.42	1.09
Allergic and chronic rhinitis	0.00	0.00	0.56	0.10	0.27
Alopecia areata	0.00	0.00	0.87	0.17	0.45
Anaemia_other	0.00	0.00	1.49	0.33	0.78
Angiodysplasia of colon	0.00	0.00	0.87	0.17	0.49
Anterior and Intermediate Uveitis	0.00	0.00	1.18	0.25	0.66
Aplastic anaemias	0.00	0.00	2.19	0.47	1.42
Asbestosis	0.00	0.00	0.96	0.20	0.65
Atrioventricular blocks	0.00	0.00	0.64	0.11	0.33

Beaney T, *et al. BMJ Open* 2023; 13:e072884. doi: 10.1136/bmjopen-2023-072884

Autism and Asperger's syndrome	0.00	0.00	1.10	0.25	0.58
Autonomic Neuropathy	0.00	0.00	2.46	0.47	1.34
Benign Prostatic Hyperplasia	0.00	0.00	1.08	0.25	0.50
Benign essential tremor	0.00	0.00	1.11	0.22	0.53
Cardiomyopathy_other	0.00	0.00	1.94	0.41	0.90
Cataract	0.00	0.00	1.16	0.27	0.50
Cerebral Palsy	0.00	0.00	0.73	0.16	0.48
Chronic Cystitis	0.00	0.00	1.88	0.37	1.03
Chronic Kidney Disease	0.00	0.00	1.16	0.26	0.65
Chronic sinusitis	0.00	0.00	0.72	0.13	0.39
Chronic viral hepatitis	0.00	0.00	1.89	0.40	0.90
Collapsed vertebra	0.00	0.00	1.64	0.34	0.77
Congenital Septal Defect	0.00	0.00	1.21	0.24	0.62
Cystic Fibrosis	0.00	0.00	2.21	0.31	1.00
Dermatitis	0.00	0.00	0.76	0.15	0.43
Diabetic Neuropathy	0.00	0.00	1.62	0.38	1.44
Diaphragmatic hernia	0.00	0.00	0.81	0.17	0.38
Diverticular Disease	0.00	0.00	0.96	0.20	0.51
Down's syndrome	0.00	0.00	0.48	0.10	0.19
Dysmenorrhoea	0.00	0.00	0.78	0.15	0.38
Endometrial hyperplasia and hypertrophy	0.00	0.00	0.90	0.17	0.57
Endometriosis	0.00	0.00	2.08	0.44	1.06
Enteropathic arthropathy	0.00	0.00	1.28	0.38	0.99
Enthesopathy and synovial disorder	0.00	0.00	0.86	0.18	0.43
Fatty Liver	0.00	0.00	0.75	0.14	0.34
Fibromatosis	0.00	0.00	0.85	0.17	0.39
Folate deficiency anaemia	0.00	0.00	0.52	0.09	0.25
Gastritis and duodenitis	0.00	0.00	0.73	0.14	0.39
Gastro-oesophageal reflux disease	0.00	0.00	0.88	0.18	0.43
Glaucoma	0.00	0.00	1.46	0.31	0.62
HIV	0.00	0.00	2.07	0.41	0.92
Hearing loss	0.00	0.00	0.77	0.16	0.34
Hepatic failure	0.00	0.00	2.22	0.46	1.07
Hidradenitis suppurativa	0.00	0.00	1.92	0.43	1.11
Hyperparathyroidism	0.00	0.00	1.84	0.41	0.84
Hypersplenism	0.00	0.00	0.99	0.21	0.58
Hypertrophic Cardiomyopathy	0.00	0.00	2.23	0.49	1.00
Hypertrophic Nasal Turbinates	0.00	0.00	0.28	0.04	0.16
Hyposplenism	0.00	0.00	1.50	0.34	0.71
Immunodeficiencies	0.00	0.00	1.62	0.36	1.11
Intervertebral disc disorders	0.00	0.00	1.75	0.36	0.91
Irritable bowel syndrome	0.00	0.00	0.66	0.13	0.32
Ischaemic stroke	0.00	0.00	2.03	0.46	0.99
Left bundle branch block	0.00	0.00	0.77	0.15	0.39
Macular degeneration	0.00	0.00	1.16	0.25	0.71
Meniere's Disease	0.00	0.00	1.58	0.33	0.77
Migraine	0.00	0.00	1.21	0.25	0.61
Multiple valve disorder	0.00	0.00	0.49	0.09	0.33
Neuropathic Bladder	0.00	0.00	0.74	0.15	0.36

Beaney T, *et al. BMJ Open* 2023; 13:e072884. doi: 10.1136/bmjopen-2023-072884

Nonrheumatic aortic valve disorders	0.00	0.00	1.42	0.31	0.68
Nonrheumatic mitral valve disorders	0.00	0.00	0.84	0.16	0.52
Obesity	0.00	0.00	0.71	0.15	0.44
Obsessive-compulsive disorder	0.00	0.00	2.55	0.56	1.21
Obstructive and reflux uropathy	0.00	0.00	1.10	0.23	0.63
Oesophageal varices	0.00	0.00	1.62	0.38	0.74
Osteoarthritis (excl spine)	0.00	0.00	1.53	0.34	0.70
Other haemolytic anaemias	0.00	0.00	3.09	0.62	1.64
Pancreatitis	0.00	0.00	2.00	0.44	1.09
Pericardial Effusion	0.00	0.00	1.12	0.21	0.56
Peripheral Neuropathy	0.00	0.00	1.22	0.26	0.81
Pleural effusion	0.00	0.00	1.55	0.32	0.90
Pleural plaque	0.00	0.00	0.74	0.14	0.48
Polycystic ovarian syndrome	0.00	0.00	0.86	0.20	0.34
Polycythaemia vera	0.00	0.00	2.49	0.54	1.30
Portal hypertension	0.00	0.00	0.91	0.18	0.46
Posterior Uveitis	0.00	0.00	1.46	0.33	1.02
Primary Malignancy_Multiple Sites	0.00	0.00	0.00	0.00	0.00
Primary Malignancy_Skin	0.00	0.00	1.30	0.31	0.78
Primary Malignancy_other	0.00	0.00	4.42	0.90	2.44
Primary Thrombocytopaenia	0.00	0.00	2.41	0.59	1.96
Primary pulmonary hypertension	0.00	0.00	1.62	0.32	1.00
Psoriasis	0.00	0.00	1.44	0.32	0.75
Pulmonary Fibrosis	0.00	0.00	2.38	0.53	1.34
Raynaud's syndrome	0.00	0.00	0.85	0.16	0.45
Retinal vascular occlusions	0.00	0.00	1.93	0.42	0.93
Rheumatic Valve Disorder	0.00	0.00	0.70	0.13	0.41
Right bundle branch block combinations	0.00	0.00	0.47	0.08	0.25
Rosacea	0.00	0.00	0.93	0.20	0.41
Scleritis and episcleritis	0.00	0.00	0.70	0.13	0.49
Seborrheic dermatitis	0.00	0.00	0.61	0.11	0.31
Secondary Malignancy_Adrenal Gland	0.00	0.00	1.68	0.42	1.01
Secondary Malignancy_Bone	0.00	0.00	4.78	0.93	2.34
Secondary Malignancy_Bowel	0.00	0.00	6.36	1.41	2.42
Secondary Malignancy_Liver	0.00	0.00	4.82	0.91	2.26
Secondary Malignancy_Lung	0.00	0.00	6.04	1.10	2.27
Secondary Malignancy_Lymph Nodes	0.00	0.00	2.40	0.40	1.31
Secondary Malignancy_Pleura	0.00	0.00	5.69	0.94	2.50
Secondary Thrombocytopaenia	0.00	0.00	0.89	0.19	0.48
Secondary polycythaemia	0.00	0.00	1.64	0.32	0.78
Secondary pulmonary hypertension	0.00	0.00	1.29	0.27	0.83
Sick sinus syndrome	0.00	0.00	0.79	0.14	0.40
Sickle Cell Disease	0.00	0.00	0.98	0.29	1.07
Sjogren's Syndrome	0.00	0.00	1.48	0.31	0.68
Sleep apnoea	0.00	0.00	0.92	0.19	0.43
Spina bifida	0.00	0.00	0.48	0.11	0.44
Spinal stenosis	0.00	0.00	2.34	0.50	1.06
Spondylolisthesis	0.00	0.00	1.22	0.23	0.63
Spondylosis	0.00	0.00	1.01	0.21	0.57
Stable Angina	0.00	0.00	1.62	0.37	0.78

Beaney T, *et al. BMJ Open* 2023; 13:e072884. doi: 10.1136/bmjopen-2023-072884

Subarachnoid haemorrhage	0.00	0.00	2.41	0.51	1.05
Substance Misuse	0.00	0.00	1.42	0.32	1.34
Supraventricular tachycardia	0.00	0.00	1.55	0.35	0.78
Thalassaemia	0.00	0.00	0.31	0.05	0.19
Thrombophilia	0.00	0.00	0.75	0.15	0.53
Tinnitus	0.00	0.00	0.85	0.17	0.43
Transient ischaemic attack	0.00	0.00	1.56	0.35	0.70
Trigeminal neuralgia	0.00	0.00	2.16	0.47	1.05
Tubulo-interstitial nephritis	0.00	0.00	2.70	0.50	1.23
Unstable Angina	0.00	0.00	1.17	0.23	0.58
Urinary Incontinence	0.00	0.00	0.87	0.18	0.38
Venous thromboembolic disease (Excl PE)	0.00	0.00	1.85	0.41	1.05
Ventricular tachycardia	0.00	0.00	1.64	0.32	0.75
Visual impairment and blindness	0.00	0.00	0.73	0.13	0.31
Vitiligo	0.00	0.00	0.73	0.14	0.32

Figure A1: Boxplots of observed and expected mean yearly codes at a GP practice level for QOF conditions in year 1 (A) and year 2 (B) and non-QOF conditions in year 1 (C) and year 2 (D) following diagnosis

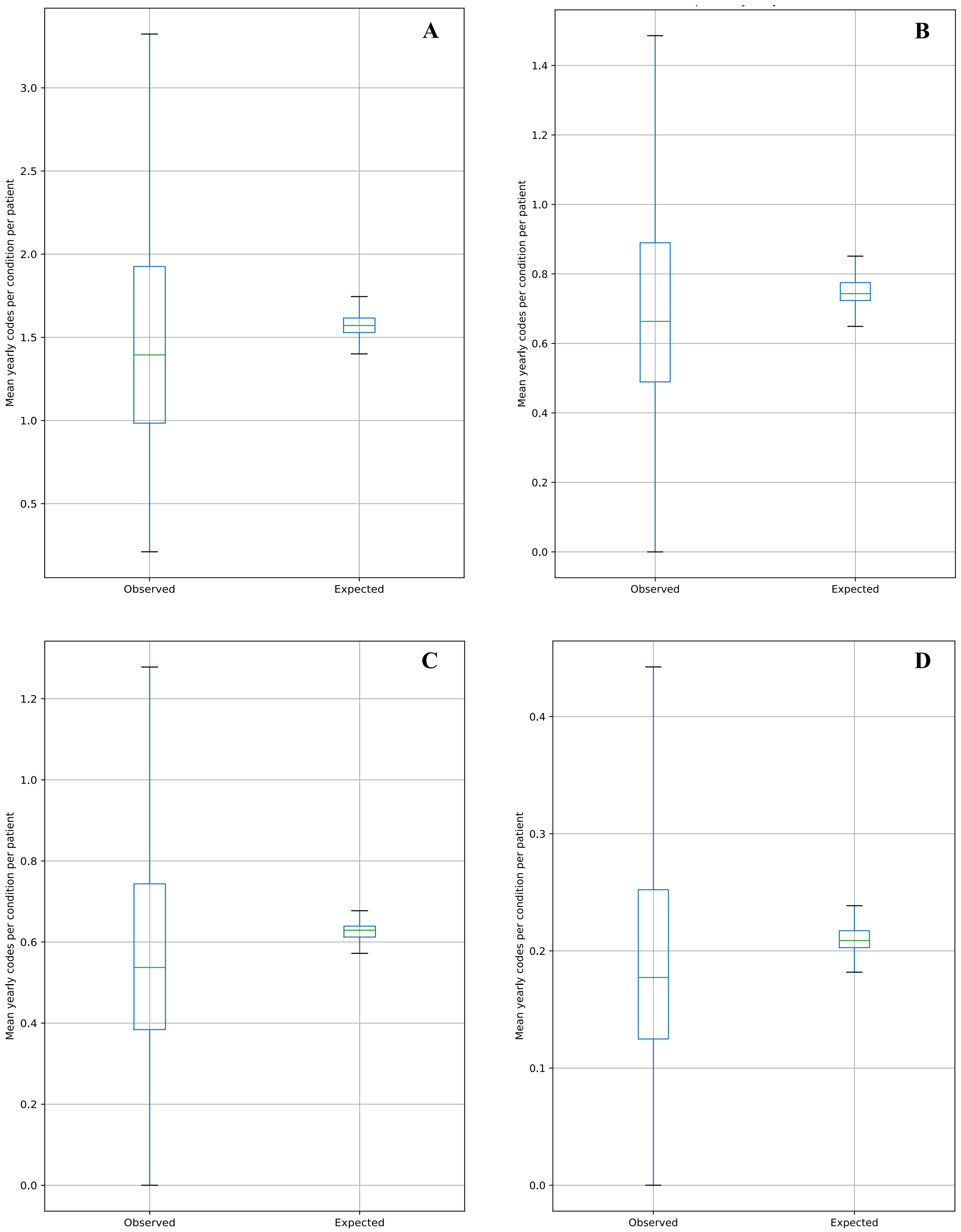

Figure A2: ratio of mean yearly codes in year 1 following diagnosis to subsequent years for QOF conditions

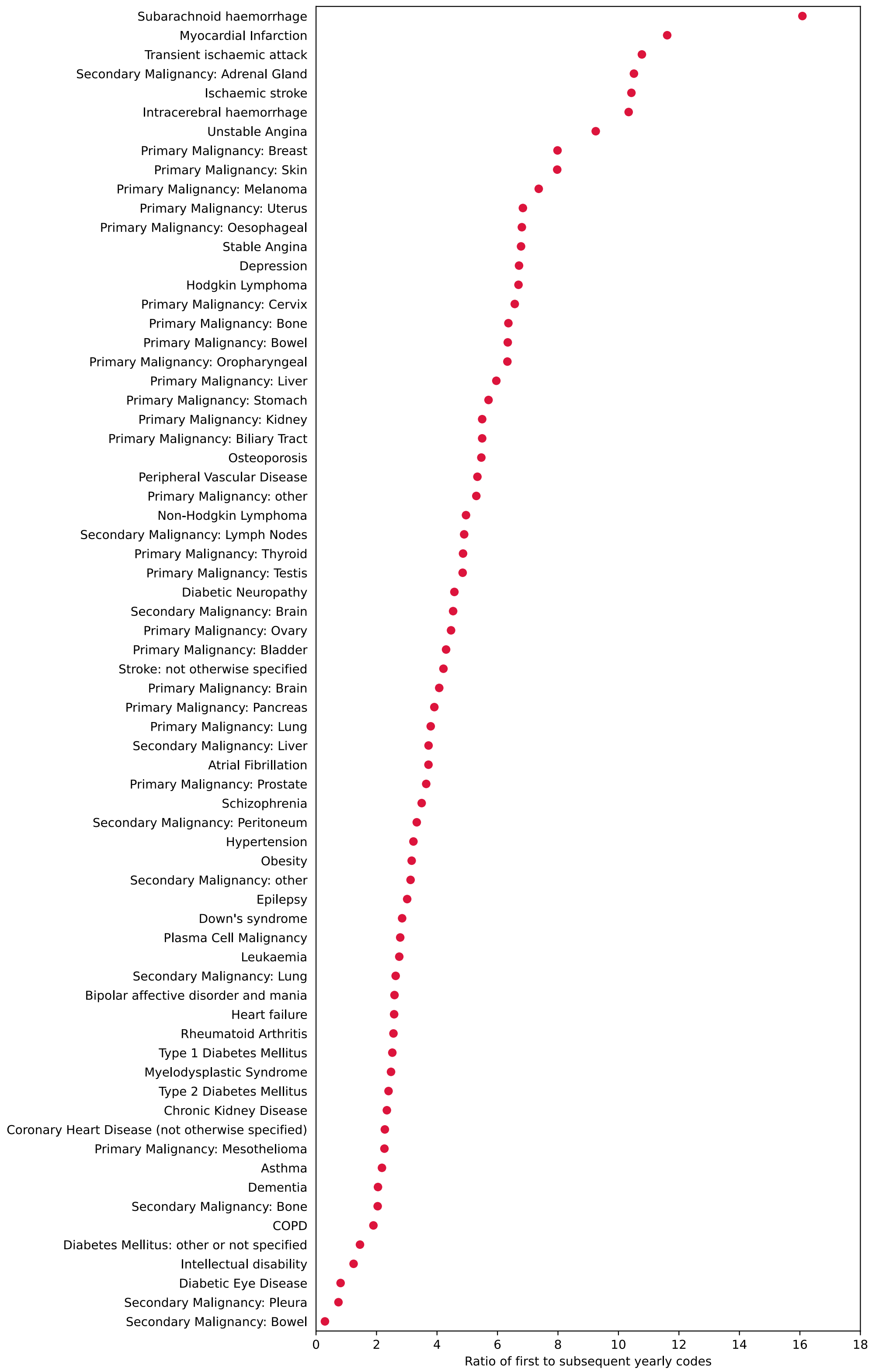

Figure A3: Ratio of mean yearly codes in year 1 following diagnosis to subsequent years for non-QOF conditions

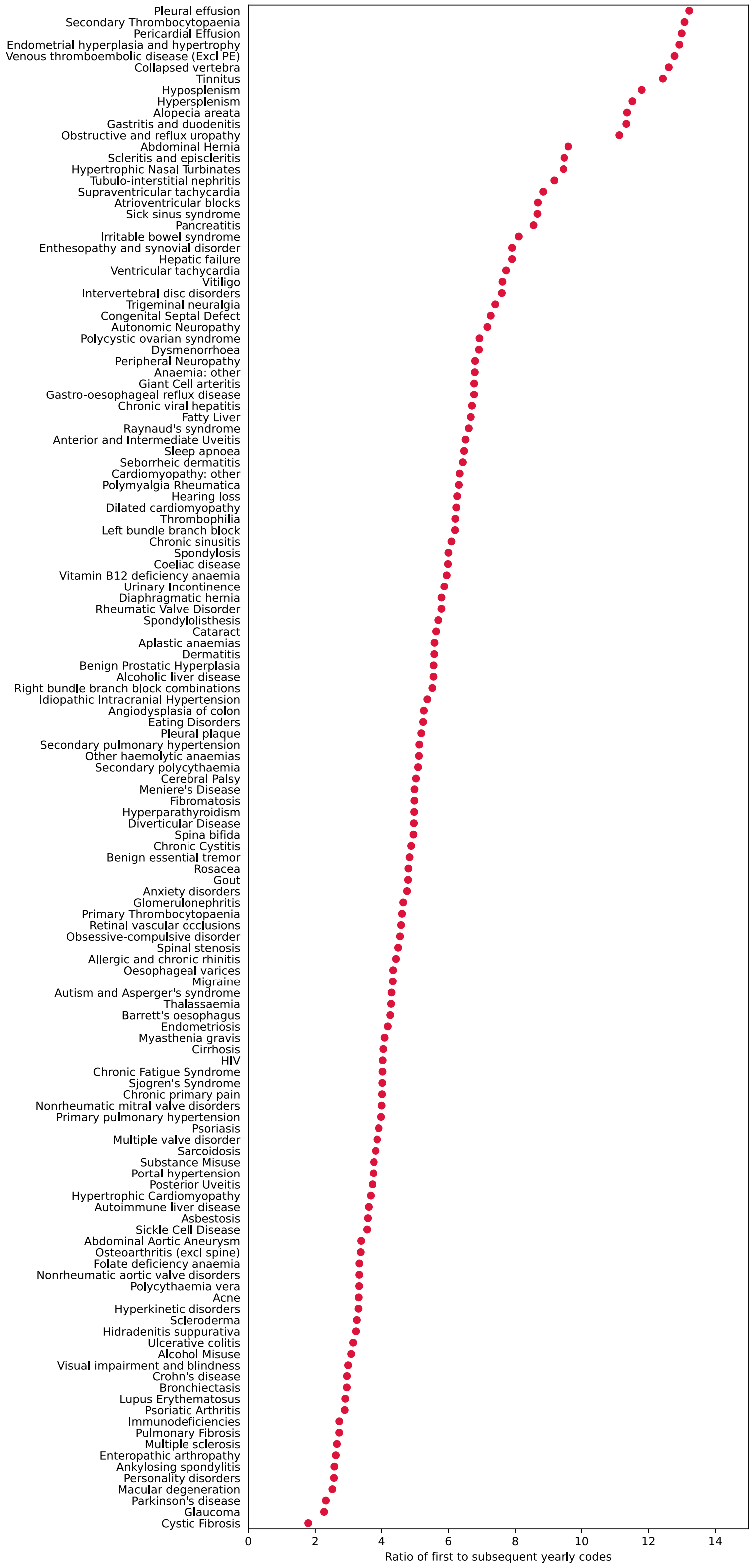

Beaney T, *et al. BMJ Open* 2023; 13:e072884. doi: 10.1136/bmjopen-2023-072884

Figure A4: Boxplots of the distribution of mean yearly codes following diagnosis for newly diagnosed conditions by GP practice stratified by inclusion in QOF

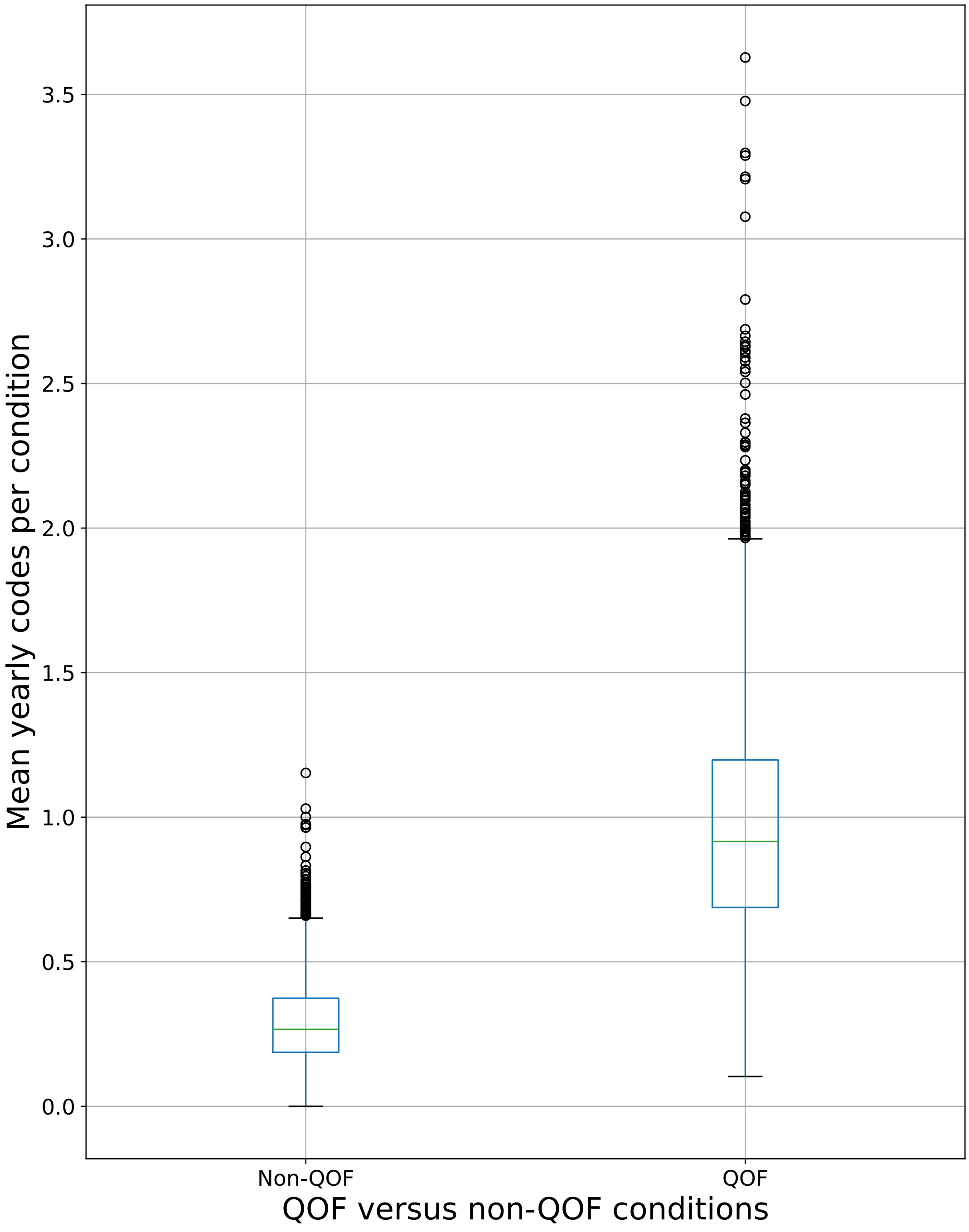

Beaney T, *et al. BMJ Open* 2023; 13:e072884. doi: 10.1136/bmjopen-2023-072884

Figure A5: boxplots of mean yearly codes at a GP practice level by practice level Index of Multiple Deprivation decile (1 = most deprived, 10 = least deprived)

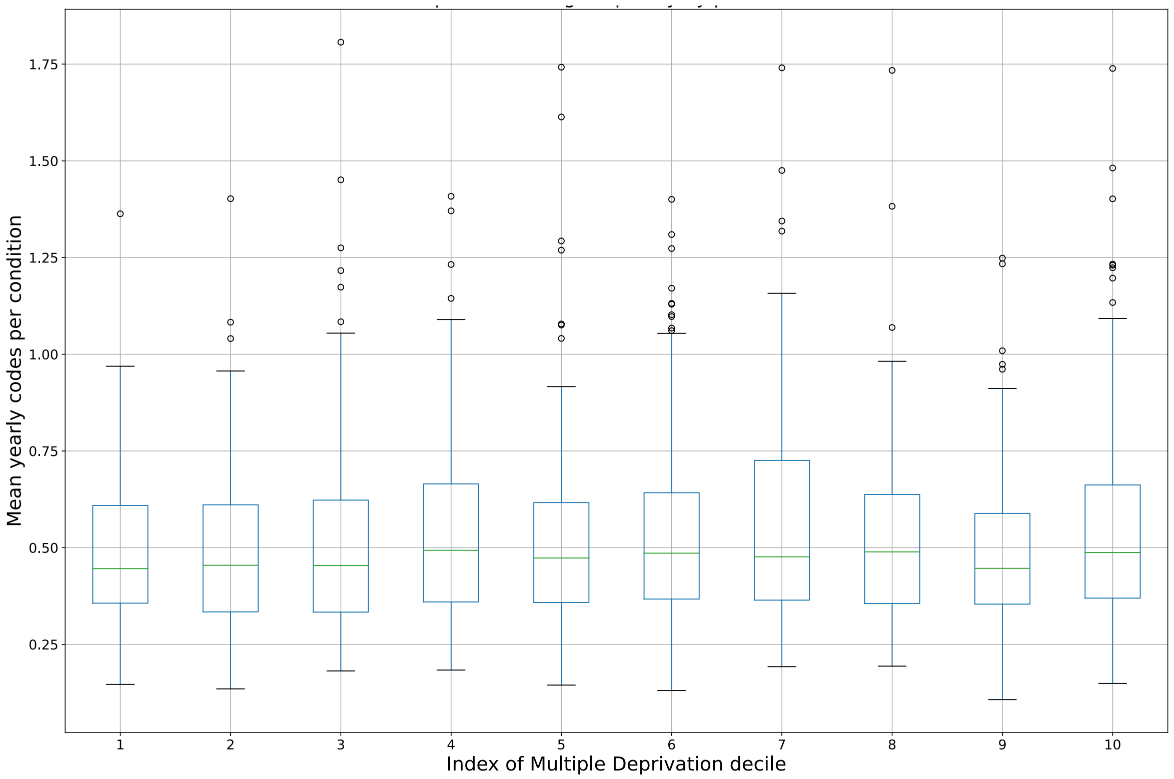

Footnote: combines QOF and non-QOF conditions

Table A5: Associations of rate of codes in year one following diagnosis for conditions included in QOF (N=1730485)

	Primary analysis				Sensitivity analysis including consultation number			
			95% CI				95% CI	
**Variable**	**IRR**	**P-value**	**Lower**	**Upper**	**IRR**	**P-value**	**Lower**	**Upper**
**Age category (years)**								
Under 40	1.33	0.00	1.32	1.34	1.30	0.00	1.29	1.31
40-49	1.15	0.00	1.14	1.15	1.14	0.00	1.13	1.15
50-59	1.08	0.00	1.07	1.08	1.07	0.00	1.07	1.08
60-69 (reference)	-	-	-	-	-	-	-	-
70-79	0.96	0.00	0.95	0.96	0.94	0.00	0.93	0.95
80 or more	0.91	0.00	0.90	0.92	0.88	0.00	0.87	0.88
**Sex**								
Female (reference)	-	-	-	-	-	-	-	-
Male	1.03	0.00	1.02	1.03	1.10	0.00	1.10	1.11
**Ethnicity category**								
White (reference)	-	-	-	-	-	-	-	-
South Asian	0.96	0.00	0.95	0.97	0.92	0.00	0.91	0.93
Black	0.94	0.00	0.93	0.95	0.94	0.00	0.93	0.95
Other	0.95	0.00	0.93	0.97	0.96	0.00	0.94	0.98
Mixed	0.98	0.03	0.95	1.00	0.97	0.00	0.95	0.99
Missing	0.98	0.00	0.97	0.99	1.01	0.00	1.00	1.02
**IMD decile**								
1 (most deprived)	-	-	-	-	-	-	-	-
2	1.01	0.19	1.00	1.01	1.00	0.95	0.99	1.01
3	1.02	0.00	1.01	1.03	1.01	0.08	1.00	1.02
4	1.02	0.00	1.01	1.03	1.01	0.01	1.00	1.02
5	1.02	0.00	1.01	1.03	1.01	0.06	1.00	1.02
6	1.03	0.00	1.02	1.04	1.01	0.02	1.00	1.02
7	1.04	0.00	1.03	1.05	1.02	0.00	1.01	1.03
8	1.04	0.00	1.03	1.05	1.01	0.01	1.00	1.02
9	1.05	0.00	1.04	1.06	1.02	0.00	1.01	1.03
10 (least deprived)	1.05	0.00	1.04	1.06	1.01	0.06	1.00	1.02
**Number of QOF diseases**								
0 (reference)	-	-	-	-	-	-	-	-
1	0.90	0.00	0.90	0.91	0.87	0.00	0.86	0.87
2	0.80	0.00	0.80	0.81	0.75	0.00	0.75	0.76
3	0.71	0.00	0.70	0.71	0.66	0.00	0.65	0.66
4 or more	0.63	0.00	0.62	0.63	0.56	0.00	0.55	0.56
**Number of non-QOF diseases**								
0 (reference)	-	-	-	-	-	-	-	-
1	1.16	0.00	1.16	1.17	1.08	0.00	1.07	1.08
2	1.13	0.00	1.12	1.14	1.02	0.00	1.01	1.02
3	1.12	0.00	1.11	1.12	0.97	0.00	0.96	0.98
4 or more	1.13	0.00	1.12	1.13	0.90	0.00	0.89	0.90
**Calendar year of diagnosis**								
2015 (reference)	-	-	-	-	-	-	-	-
2016	1.00	0.89	0.99	1.01	1.02	0.00	1.02	1.03
2017	1.00	0.34	1.00	1.01	1.05	0.00	1.04	1.05
2018	1.00	0.18	0.99	1.00	1.06	0.00	1.06	1.07
2019	0.95	0.00	0.94	0.96	1.04	0.00	1.04	1.05
**Average number of consultations in year 1**								
Less than 1 (reference)	-	-	-	-	-	-	-	-
1-2	-	-	-	-	1.62	0.00	1.60	1.63
3-4	-	-	-	-	2.21	0.00	2.19	2.23
5-9	-	-	-	-	2.87	0.00	2.84	2.89
10 or more	-	-	-	-	3.75	0.00	3.71	3.79

From negative binomial regression models, including practice-level fixed effects (not shown)

Beaney T, *et al. BMJ Open* 2023; 13:e072884. doi: 10.1136/bmjopen-2023-072884

Table A6: Associations of rate of codes in year two following diagnosis for conditions included in QOF (N=1714684)

	Primary analysis				Sensitivity analysis including consultation number			
			95% CI				95% CI	
**Variable**	**IRR**	**P-value**	**Lower**	**Upper**	**IRR**	**P-value**	**Lower**	**Upper**
**Age category (years)**								
Under 40	0.87	0.00	0.86	0.87	0.86	0.00	0.86	0.87
40-49	1.01	0.03	1.00	1.02	1.01	0.22	1.00	1.01
50-59	1.03	0.00	1.02	1.04	1.03	0.00	1.02	1.04
60-69 (reference)	-	-	-	-	-	-	-	-
70-79	0.95	0.00	0.94	0.96	0.93	0.00	0.93	0.94
80 or more	0.89	0.00	0.88	0.90	0.86	0.00	0.85	0.87
**Sex**								
Female (reference)	-	-	-	-	-	-	-	-
Male	1.11	0.00	1.11	1.12	1.18	0.00	1.17	1.18
**Ethnicity category**								
White (reference)	-	-	-	-	-	-	-	-
South Asian	1.26	0.00	1.25	1.28	1.22	0.00	1.20	1.23
Black	1.17	0.00	1.16	1.19	1.17	0.00	1.15	1.19
Other	1.13	0.00	1.10	1.16	1.14	0.00	1.11	1.17
Mixed	1.12	0.00	1.08	1.15	1.11	0.00	1.07	1.14
Missing	0.89	0.00	0.88	0.90	0.93	0.00	0.92	0.93
**IMD decile**								
1 (most deprived)	-	-	-	-	-	-	-	-
2	1.02	0.00	1.01	1.04	1.02	0.00	1.01	1.03
3	1.03	0.00	1.02	1.05	1.03	0.00	1.02	1.04
4	1.05	0.00	1.03	1.06	1.04	0.00	1.03	1.05
5	1.05	0.00	1.04	1.07	1.04	0.00	1.03	1.06
6	1.06	0.00	1.05	1.07	1.05	0.00	1.04	1.07
7	1.08	0.00	1.06	1.09	1.06	0.00	1.05	1.08
8	1.09	0.00	1.07	1.10	1.07	0.00	1.06	1.08
9	1.11	0.00	1.10	1.13	1.09	0.00	1.08	1.11
10 (least deprived)	1.14	0.00	1.12	1.15	1.11	0.00	1.09	1.12
**Number of QOF diseases**								
0 (reference)	-	-	-	-	-	-	-	-
1	1.05	0.00	1.04	1.06	1.00	0.79	0.99	1.01
2	1.07	0.00	1.06	1.08	0.99	0.05	0.98	1.00
3	0.99	0.15	0.98	1.00	0.91	0.00	0.90	0.92
4 or more	0.87	0.00	0.86	0.88	0.77	0.00	0.76	0.78
**Number of non-QOF diseases**								
0 (reference)	-	-	-	-	-	-	-	-
1	1.05	0.00	1.04	1.06	0.99	0.11	0.98	1.00
2	1.04	0.00	1.03	1.05	0.96	0.00	0.95	0.97
3	1.04	0.00	1.03	1.05	0.93	0.00	0.92	0.94
4 or more	1.05	0.00	1.04	1.06	0.88	0.00	0.87	0.89
**Calendar year of diagnosis**								
2015 (reference)	-	-	-	-	-	-	-	-
2016	1.00	0.45	0.99	1.01	1.02	0.00	1.01	1.03
2017	0.99	0.00	0.98	0.99	1.02	0.00	1.01	1.03
2018	0.91	0.00	0.90	0.92	0.96	0.00	0.95	0.97
2019	0.79	0.00	0.79	0.80	0.86	0.00	0.86	0.87
**Average number of consultations in year 1**								
Less than 1 (reference)					-	-	-	-
2					1.53	0.00	1.52	1.55
3-4					1.87	0.00	1.85	1.89
5-9					2.17	0.00	2.15	2.20
10 or more					2.59	0.00	2.57	2.62

From negative binomial regression models, including practice-level fixed effects (not shown)

Beaney T, *et al. BMJ Open* 2023; 13:e072884. doi: 10.1136/bmjopen-2023-072884

Table A7: Associations of rate of codes in year one following diagnosis for conditions not included in QOF (N=3617348)

	**Primary analysis**				**Sensitivity analysis including consultation number**			
			**95% CI**				**95% CI**	
**Variable**	**IRR**	**P-value**	**Lower**	**Upper**	**IRR**	**P-value**	**Lower**	**Upper**
**Age category (years)**								
Under 40	1.10	0.00	1.10	1.11	1.09	0.00	1.08	1.10
40-49	1.01	0.00	1.00	1.02	1.02	0.00	1.01	1.03
50-59	0.98	0.00	0.98	0.99	0.99	0.09	0.99	1.00
60-69 (reference)	-	-	-	-	-	-	-	-
70-79	1.05	0.00	1.05	1.06	1.03	0.00	1.02	1.03
80 or more	1.02	0.00	1.02	1.03	0.98	0.00	0.97	0.99
**Sex**								
Female (reference)	-	-	-	-	-	-	-	-
Male	1.00	0.03	0.99	1.00	1.13	0.00	1.12	1.13
**Ethnicity category**								
White (reference)	-	-	-	-	-	-	-	-
South Asian	0.95	0.00	0.94	0.96	0.89	0.00	0.88	0.90
Black	0.89	0.00	0.88	0.90	0.86	0.00	0.85	0.87
Other	0.90	0.00	0.88	0.91	0.89	0.00	0.88	0.91
Mixed	0.95	0.00	0.93	0.97	0.92	0.00	0.91	0.94
Missing	0.99	0.14	0.99	1.00	1.06	0.00	1.05	1.06
**IMD decile**								
1 (most deprived)	-	-	-	-	-	-	-	-
2	1.00	0.86	0.99	1.01	0.99	0.06	0.98	1.00
3	1.01	0.01	1.00	1.02	1.00	0.82	0.99	1.01
4	1.02	0.00	1.01	1.03	1.00	0.42	0.99	1.01
5	1.02	0.00	1.01	1.03	1.00	0.86	0.99	1.01
6	1.03	0.00	1.02	1.04	0.99	0.26	0.99	1.00
7	1.03	0.00	1.02	1.04	0.99	0.08	0.98	1.00
8	1.04	0.00	1.03	1.06	0.99	0.15	0.98	1.00
9	1.06	0.00	1.05	1.07	0.99	0.19	0.98	1.00
10 (least deprived)	1.06	0.00	1.05	1.07	0.98	0.00	0.97	0.99
**Number of QOF diseases**								
0 (reference)	-	-	-	-	-	-	-	-
1	1.16	0.00	1.15	1.16	1.02	0.00	1.02	1.03
2	1.09	0.00	1.08	1.09	0.94	0.00	0.93	0.94
3	1.06	0.00	1.05	1.07	0.90	0.00	0.89	0.91
4 or more	1.04	0.00	1.03	1.04	0.85	0.00	0.84	0.85
**Number of non-QOF diseases**								
0 (reference)	-	-	-	-	-	-	-	-
1	1.02	0.00	1.01	1.02	0.93	0.00	0.92	0.94
2	1.02	0.00	1.02	1.03	0.87	0.00	0.87	0.88
3	1.04	0.00	1.03	1.05	0.83	0.00	0.82	0.84
4 or more	1.06	0.00	1.06	1.07	0.74	0.00	0.74	0.75
**Calendar year of diagnosis**								
2015 (reference)	-	-	-	-	-	-	-	-
2016	1.00	0.55	0.99	1.00	1.03	0.00	1.02	1.03
2017	0.99	0.00	0.99	1.00	1.05	0.00	1.04	1.05
2018	0.99	0.00	0.98	0.99	1.07	0.00	1.06	1.07
2019	0.94	0.00	0.94	0.95	1.06	0.00	1.06	1.07
**Average number of consultations in year 1**								
Less than 1 (reference)					-	-	-	-
1-2					2.38	0.00	2.36	2.40
3-4					3.49	0.00	3.45	3.52
5-9					4.67	0.00	4.62	4.71
10 or more					6.37	0.00	6.31	6.44

From negative binomial regression models, including practice-level fixed effects (not shown)

Beaney T, *et al. BMJ Open* 2023; 13:e072884. doi: 10.1136/bmjopen-2023-072884

Table A8: Associations of rate of codes in year two following diagnosis for conditions not included in QOF (N=3593019)

	Primary analysis				Sensitivity analysis including consultation number			
			95% CI				95% CI	
**Variable**	**IRR**	**P-value**	**Lower**	**Upper**	**IRR**	**P-value**	**Lower**	**Upper**
**Age category (years)**								
Under 40	1.27	0.00	1.26	1.28	1.26	0.00	1.25	1.28
40-49	1.03	0.00	1.02	1.04	1.03	0.00	1.02	1.04
50-59	0.98	0.00	0.97	0.99	0.99	0.10	0.98	1.00
60-69 (reference)	-	-	-	-	-	-	-	-
70-79	1.06	0.00	1.05	1.07	1.03	0.00	1.02	1.04
80 or more	1.06	0.00	1.05	1.08	1.01	0.18	1.00	1.02
**Sex**								
Female (reference)	-	-	-	-	-	-	-	-
Male	0.93	0.00	0.93	0.94	1.08	0.00	1.07	1.09
**Ethnicity category**								
White (reference)	-	-	-	-	-	-	-	-
South Asian	0.99	0.17	0.97	1.00	0.92	0.00	0.91	0.94
Black	0.94	0.00	0.92	0.95	0.91	0.00	0.89	0.92
Other	0.88	0.00	0.86	0.91	0.89	0.00	0.86	0.92
Mixed	0.94	0.00	0.91	0.98	0.92	0.00	0.89	0.95
Missing	0.96	0.00	0.95	0.97	1.05	0.00	1.03	1.06
**IMD decile**								
1 (most deprived)	-	-	-	-	-	-	-	-
2	1.01	0.10	1.00	1.03	1.00	0.79	0.99	1.02
3	1.03	0.00	1.02	1.05	1.02	0.00	1.01	1.04
4	1.04	0.00	1.02	1.05	1.02	0.01	1.01	1.04
5	1.05	0.00	1.04	1.07	1.03	0.00	1.01	1.04
6	1.06	0.00	1.04	1.08	1.03	0.00	1.01	1.04
7	1.07	0.00	1.06	1.09	1.03	0.00	1.01	1.05
8	1.10	0.00	1.08	1.11	1.04	0.00	1.03	1.06
9	1.13	0.00	1.11	1.14	1.06	0.00	1.04	1.08
10 (least deprived)	1.14	0.00	1.12	1.16	1.06	0.00	1.04	1.08
**Number of QOF diseases**								
0 (reference)	-	-	-	-	-	-	-	-
1	1.19	0.00	1.18	1.21	1.05	0.00	1.04	1.06
2	1.15	0.00	1.14	1.16	0.98	0.00	0.97	0.99
3	1.13	0.00	1.12	1.15	0.95	0.00	0.94	0.96
4 or more	1.16	0.00	1.14	1.17	0.93	0.00	0.92	0.94
**Number of non-QOF diseases**								
0 (reference)	-	-	-	-	-	-	-	-
1	1.04	0.00	1.03	1.06	0.94	0.00	0.93	0.95
2	1.09	0.00	1.08	1.11	0.90	0.00	0.89	0.91
3	1.13	0.00	1.11	1.14	0.86	0.00	0.85	0.87
4 or more	1.21	0.00	1.20	1.23	0.80	0.00	0.79	0.81
**Calendar year of diagnosis**								
2015 (reference)	-	-	-	-	-	-	-	-
2016	1.00	0.56	0.99	1.01	1.03	0.00	1.02	1.04
2017	1.00	0.43	0.99	1.01	1.06	0.00	1.05	1.07
2018	0.91	0.00	0.90	0.92	1.01	0.01	1.00	1.02
2019	0.79	0.00	0.79	0.80	0.93	0.00	0.92	0.94
**Average number of consultations in year 1**								
Less than 1 (reference)					-	-	-	-
1-2					2.76	0.00	2.72	2.81
3-4					4.06	0.00	4.00	4.12
5-9					5.40	0.00	5.32	5.48
10 or more					7.35	0.00	7.24	7.47

From negative binomial regression models, including practice-level fixed effects (not shown)

References

1. Head, A. *et al.* Inequalities in incident and prevalent multimorbidity in England, 2004–19: a population-based, descriptive study. *The Lancet Healthy Longevity* **2**, e489–e497 (2021).
2. Kuan, V. *et al.* A chronological map of 308 physical and mental health conditions from 4 million individuals in the English National Health Service. *The Lancet Digital Health* **1**, e63–e77 (2019).
3. Bisquera, A. *et al.* Inequalities in developing multimorbidity over time: A population-based cohort study from an urban, multi-ethnic borough in the United Kingdom. *Lancet Reg Health Eur* **12**, 100247 (2021).
4. Ashworth, M. *et al.* Journey to multimorbidity: longitudinal analysis exploring cardiovascular risk factors and sociodemographic determinants in an urban setting. *BMJ Open* **9**, (2019).
5. NHS Health and Social Care Information Centre. National Quality and Outcomes Framework Statistics for England 2004/05. https://files.digital.nhs.uk/publicationimport/pub01xxx/pub01946/qof-eng-04-05-rep.pdf.
6. Health & Social Care Information Centre. Quality and Outcomes Framework – Prevalence, Achievements and Exceptions Report. England, 2013-14. https://files.digital.nhs.uk/publicationimport/pub15xxx/pub15751/qof-1314-report-v1.1.pdf.
7. Health & Social Care Information Centre. Quality and Outcomes Framework – Prevalence, Achievements and Exceptions Report, England, 2014-15. https://files.digital.nhs.uk/publicationimport/pub18xxx/pub18887/qof-1415-report%20v1.1.pdf (2015).
