# Supplementary material for: Identifying potential biases in code sequences in primary care electronic healthcare records: a retrospective cohort study of the determinants of code frequency
Source: BMJ Open. 2023 Sep 27;13(9):e072884. doi: 10.1136/bmjopen-2023-072884 (PMC10537851; doi:10.1136/bmjopen-2023-072884)
Supplement: Author's manuscript [file bmjopen-2023-072884.draft_revisions.pdf]

BMJ Open is committed to open peer review. As part of this commitment we make the peer review history of every article we publish publicly available.

When an article is published we post the peer reviewers' comments and the authors' responses online. We also post the versions of the paper that were used during peer review. These are the versions that the peer review comments apply to.

The versions of the paper that follow are the versions that were submitted during the peer review process. They are not the versions of record or the final published versions. They should not be cited or distributed as the published version of this manuscript.

BMJ Open is an open access journal and the full, final, typeset and author-corrected version of record of the manuscript is available on our site with no access controls, subscription charges or pay-per-view fees (http://bmjopen.bmj.com).

If you have any questions on BMJ Open's open peer review process please email info.bmjopen@bmj.com

BMJ Open

Determinants of disease code frequency in the primary care electronic healthcare record: a retrospective cohort study

Journal:	*BMJ Open*
Manuscript ID	bmjopen-2023-072884
Article Type:	Original research
Date Submitted by the Author:	16-Feb-2023
Complete List of Authors:	Beaney, Thomas; Imperial College London, Department of Primary Care and Public Health; Imperial College London, Department of Mathematics<br>Clarke, Jonathan; Imperial College of Science Technology and Medicine, Institute of Global Health Innovation<br>Salman, David; Imperial College London Department of Primary Care and Public Health; Imperial College London Faculty of Medicine, MSk lab<br>Woodcock, Thomas; Imperial College London, Department of Primary Care and Public Health<br>Majeed, Azeem; Imperial College London, Department of Primary Care and Public Health<br>Barahona, Mauricio; Imperial College London, Centre for Mathematics of Precision Healthcare; Imperial College London, Department of Mathematics<br>Aylin, Paul; Imperial College London, Department of Primary Care and Public Health
Keywords:	EPIDEMIOLOGY, Primary Health Care, Health informatics < BIOTECHNOLOGY & BIOINFORMATICS, STATISTICS & RESEARCH METHODS

SCHOLARONE™
Manuscripts

I, the Submitting Author has the right to grant and does grant on behalf of all authors of the Work (as defined in the below author licence), an exclusive licence and/or a non-exclusive licence for contributions from authors who are: i) UK Crown employees; ii) where BMJ has agreed a CC-BY licence shall apply, and/or iii) in accordance with the terms applicable for US Federal Government officers or employees acting as part of their official duties; on a worldwide, perpetual, irrevocable, royalty-free basis to BMJ Publishing Group Ltd ("BMJ") its licensees and where the relevant Journal is co-owned by BMJ to the co-owners of the Journal, to publish the Work in this journal and any other BMJ products and to exploit all rights, as set out in our licence.

The Submitting Author accepts and understands that any supply made under these terms is made by BMJ to the Submitting Author unless you are acting as an employee on behalf of your employer or a postgraduate student of an affiliated institution which is paying any applicable article publishing charge ("APC") for Open Access articles. Where the Submitting Author wishes to make the Work available on an Open Access basis (and intends to pay the relevant APC), the terms of reuse of such Open Access shall be governed by a Creative Commons licence – details of these licences and which Creative Commons licence will apply to this Work are set out in our licence referred to above.

Other than as permitted in any relevant BMJ Author's Self Archiving Policies, I confirm this Work has not been accepted for publication elsewhere, is not being considered for publication elsewhere and does not duplicate material already published. I confirm all authors consent to publication of this Work and authorise the granting of this licence.

For peer review only

Determinants of disease code frequency in the primary care electronic healthcare record: a retrospective cohort study

Beaney T[1,2] (0000-0001-9709-7264), Clarke J[2], Salman D[1,3], Woodcock T[1], Majeed A[1], Barahona M[2], Aylin P[1]

1. Department of Primary Care and Public Health, Imperial College London, London, W6 8RP, United Kingdom
2. Centre for Mathematics of Precision Healthcare, Department of Mathematics, Imperial College London, London, SW7 2AZ, United Kingdom
3. MSk Lab, Department of Surgery and Cancer, Faculty of Medicine, Imperial College London, London, UK

Word count: 3,840

Corresponding Author:
Dr Thomas Beaney
Department of Primary Care and Public Health, Imperial College London, London, W6 8RP, United Kingdom
Email: thomas.beaney@imperial.ac.uk

For peer review only

Abstract

Objectives

To determine whether the frequency of diagnostic codes in primary care electronic health records (EHRs) is associated with i) disease coding incentives, ii) GP practice, iii) patient socio-demographic characteristics and iv) calendar year of diagnosis.

Design

Retrospective cohort study.

Setting

General practices in England from 2015 to 2022 contributing to the Clinical Practice Research Datalink Aurum dataset.

Participants

All patients registered to a GP with at least one incident disease diagnosed between 01/01/2015 and 31/12/2019.

Primary and secondary outcome measures

The number of diagnostic codes for a condition in i) the first and ii) the second year following diagnosis, stratified by inclusion in the Quality and Outcomes Framework (QOF) financial incentive programme.

Results

3,113,724 patients were included, with 7,723,365 incident diseases. Conditions included in QOF had higher rates of annual coding than conditions not included in QOF (1.03 vs 0.32 per year, $p<0.0001$). There was significant variation in code frequency by GP practice which was not explained by patient socio-demographics. We found significant associations with patient socio-demographics, with a trend towards lower coding rates in people living in areas of higher deprivation for both QOF and non-QOF conditions. Code frequency was lower for conditions with follow-up time in 2020, associated with the onset of the COVID-19 pandemic.

Conclusions

Code frequency for newly diagnostic diseases was strongly associated with patient socio-demographics, disease inclusion in QOF, GP practice, as well as with the onset of the COVID-19 pandemic. Methods using disease sequences in structured data should consider accounting for these factors to reduce potential bias.

For peer review only

Strengths and limitations

This study used a large and representative sample of patients in England and included 208 clinical conditions. However, we could not determine whether differences in code frequency represent true differences in clinical need.

For peer review only

Background

Methods developed in natural language processing (NLP) are increasingly being employed to analyse high dimensional healthcare data, such as data recorded during clinical encounters in the Electronic Healthcare Record (EHR).[1–5] These methods show promise across a range of tasks, including prediction of health outcomes, or in clustering of similar diseases.[6–8] Although designed for the analysis of free text data as found in 'unstructured' medical records, NLP methods can also be applied to the coded or 'structured' data, such as the SNOMED-CT or ICD terminologies commonly found in many EHR databases. Unlike many cross-sectional approaches, these methods make explicit use of repeated codes in the record: the sequence of codes can be regarded analogous to a sentence or document of words representing a person's life course, although without the same syntactic and semantic rules of natural language.[5] The continued evolution of transformer-based models opens up the possibility to determine the similarity between people and diseases based not only on co-occurrence of disease, as has been done in the past,[9] but on the sequence of disease acquisition,[2,5,10] which may be particularly relevant when considering preventive approaches or identifying opportunities for shared management.

In the structured medical record in primary care, a diagnostic code is presumed to indicate presentation by a patient for that condition. However, it may not be a fully objective indicator of the content of a presentation but is likely influenced by patient characteristics as well as the preferences and incentives of the clinician entering data and organisational policies; factors which may vary over time.[11,12] In England, the Quality and Outcomes Framework (QOF) was introduced in the National Health Service for General Practices (GPs) in 2004, providing financial incentives for meeting targets for a set of chronic conditions, including regular clinical reviews, and has been credited with improvements to data collection for these conditions.[13,14] Codes for conditions in QOF may occur more frequently than for conditions not included in the incentive scheme, which could affect sequence-based methods using recurrent codes.

Biases in coding may result in analytical models representing some people better than others, but little is known about the comparative frequency of medical codes for different long-term conditions (LTCs) or determinants of frequency in the primary care EHR. This study aims to compare the frequency of codes for a common set of LTCs and to determine whether coding

For peer review only

frequency varies according to i) disease inclusion in QOF, ii) GP practice, iii) patient socio-demographic characteristics, and iv) calendar year of diagnosis.

Methods

Data source

This study used data from the Clinical Practice Research Datalink (CPRD) Aurum dataset, which contains primary care data for GP practices using EMIS Web software.[15] We included all research acceptable patients with a continuous period of registration at a GP practice in CPRD between 1st January 2014 and 31st December 2020. Patients were eligible if aged 18 years or over with at least one incident disease diagnosed between 1st January 2015 and 31st December 2019, allowing for at least one full year of practice registration before disease diagnosis and at least one full year of follow-up for each condition. We focussed on incident diseases to reduce the potential for confounding from historic conditions, some of which may no longer be active. Patients were followed up until the earliest of death, de-registration or the date of latest data extraction from their GP practice. Further information on the cohort structure is given in the appendix (p2).

Disease definitions

We included a total of 208 LTCs. These were defined based on a set of disease codes from Head *et al* (2021), who selected 211 chronic conditions from 308 acute and chronic disease phenotypes developed for the CALIBER study.[16,17] We reviewed codes and made changes to the code-lists for diabetes and added a new condition of 'chronic primary pain' (see appendix p2-3). We excluded conditions based only on laboratory results or anthropometric measurement codes as these may have different characteristics of coding frequency. As a result, measures of raised cholesterol used in the original CALIBER study were excluded. We also excluded BMI and eGFR measurements but included the diagnostic codes for obesity and Chronic Kidney Disease. We considered a single code as diagnostic for each condition and defined the diagnosis date for each condition as the date of the earliest code for that condition. Diseases were stratified according to whether they appeared in QOF by two primary care clinicians, TB and DS (see appendix p2-3).

For peer review only

Statistical analysis

Descriptive statistics

For each disease newly diagnosed during the study period, we calculated the yearly number of subsequent codes (excluding the first code representing diagnosis) during follow-up:

$$y_i = \frac{\sum_{j=1}^{N} c_{i,j}}{\sum_{j=1}^{N} f_{i,j}}$$

where y_i is the yearly number of codes following diagnosis for condition i, $c_{i,j}$ is the count of codes for condition i in patient j, and $f_{i,j}$ is the number of years of follow-up for condition i in patient j. T-tests were used to compare the mean yearly number of codes for QOF versus non-QOF conditions.

To examine variation in disease coding frequency by GP practice, we calculated, for each practice k, the mean number of codes per year for newly diagnosed diseases, p_k:

$$p_k = \frac{\sum_{j=1}^{N} \sum_{i=1}^{M} c_{i,j,k}}{\sum_{j=1}^{N} \sum_{i=1}^{M} f_{i,j,k}}$$

where $c_{i,j,k}$ is the count of codes for condition i in patient j in practice k, and $f_{i,j,k}$ is the number of years of follow-up for condition i in patient j in practice k. We then calculated the Pearson correlation coefficient between the mean number of codes per year in each practice for QOF versus non-QOF conditions. We also compared the mean number of yearly codes in each practice stratified by the 2019 Index of Multiple Deprivation (IMD) decile of the GP practice.[18] For conditions with at least two years of follow-up after the date of diagnosis, we calculated the ratio of the number of codes in the first year of diagnosis to the number of codes in subsequent years.

Regression analyses

Data were formatted as panel data with patients measured over multiple calendar years (appendix Table A1). We used mixed effects negative binomial regression to analyse the association between code frequency of newly diagnosed conditions in i) the first year following diagnosis and ii) the second year following diagnosis, with patient factors and calendar year of diagnosis. We separated the outcome variable (code frequency) into first and second year after diagnosis due to preliminary analyses indicating significant differences over time. We also stratified the regression analyses by QOF inclusion, given our hypothesis that

For peer review only

it may be an effect modifier of the relationships. To account for cases where a patient may have more than one QOF or non-QOF condition diagnosed within the same year, we averaged the code frequency for all newly diagnosed QOF or non-QOF conditions in each calendar year.

Included as covariates in the model were patient socio-demographic factors including age, sex, ethnicity and IMD decile of residence. We also included the count of QOF and non-QOF conditions for each patient. Due to small numbers, we excluded patients with gender recorded in CPRD as 'indeterminate' or with missing IMD deciles. Age and the count of QOF and non-QOF conditions were time-updated at the start of each calendar year, and other covariates were held fixed. We incorporated random effects for patient and fixed effects for calendar year as we wished to explicitly model the effect of time. Use of a Poisson model was considered, but the conditional variance was found to be significantly higher than the conditional mean ($p<0.001$) indicating a negative binomial to have better fit.[19] Model fit was assessed by calculating randomized quantile residuals, which indicated no departure from normality on quantile-quantile plots.[20,21]

For each regression model, we calculated the predicted count of disease codes for each patient per year and then calculated the mean for each GP practice. This indicated that significant variation remained in the mean counts according to GP practice (appendix Figure A1). We therefore incorporated fixed effects for GP practice within the regression models to account for practice-level variation (see appendix p5 for model equation). We also compared the Akaike Information Criteria (AIC) of models with and without practice fixed effects.

To assess whether code frequency was a function of overall number of primary care consultations, we conducted a sensitivity analysis including average number of yearly consultations (irrespective of condition) in year 1 or year 2 added as a covariate into the main regression models (categorised into <1, 1-2, 3-4, 5-9 or 10 or more). Python version 3.10.6 and Pandas version 1.4.3 were used in data processing and plots and Stata version 17.0 and R studio version 4.2.1 were used for regression analyses.

Patient and Public Involvement

For peer review only

This research programme is supported by a patient and public advisory group who fed back to the researchers on the diseases included in the study but were not directly involved in this study.

For peer review only

Results

A total of 6,174,115 patients aged 18 years or over and with a continuous registration period between 1st January 2014 and 31st December 2020 were eligible for inclusion in the study. Of these, 3,113,724 (50.4%) had at least one incident disease diagnosed between 1st January 2015 and 31st December 2019. Characteristics of the eligible population are shown in Table 1. 21.4% of patients were aged between 18-40 years as of the study start date, and 7.0% were aged 80 years or over. There were more women than men (54.1% versus 45.9%), most (76.7%) were of White ethnicity and there were relatively more patients in more deprived IMD deciles (51.7% in the most deprived half).

Table 1: Socio-demographic characteristics of patients included in the study

Patient characteristic	Total	Percent
**Age (years)**		
18-40	665,543	21.4%
40-49	562,934	18.1%
50-59	604,284	19.4%
60-69	585,062	18.8%
70-79	476,626	15.3%
80+	219,275	7.0%
**Gender**		
Female	1,684,942	54.1%
Indeterminate	48	<0.1%
Male	1,428,734	45.9%
**Ethnicity**		
White	2,388,332	76.7%
South Asian	194,477	6.2%
Black	103,504	3.3%
Other	36,430	1.2%
Mixed	27,572	0.9%
Missing	363,409	11.7%
**IMD decile**		
1 (most deprived)	358,948	11.5%
2	320,042	10.3%
3	320,340	10.3%
4	323,782	10.4%
5	287,114	9.2%
6	303,798	9.8%
7	304,044	9.8%
8	298,185	9.6%
9	305,563	9.8%
10 (least deprived)	290,214	9.3%
Missing	1,694	0.1%
**Total**	**3,113,724**	

For peer review only

Code frequency by disease and by time from diagnosis

A total of 7,723,365 diseases were diagnosed during the study period with follow-up times for each disease ranging from 1.0 to 7.2 years (mean 4.1 years). There was substantial variation in the yearly code frequency after diagnosis for each condition diagnosed during the study period. Diabetes (types 1, 2 and unspecified), polymyalgia rheumatica, motor neurone disease and dementia had the highest median number of codes per year (appendix Table A2). For many chronic diseases, yearly code frequency was low, for example, only 5% of patients with spina bifida had ≥0.5 codes per year. Conditions included in QOF on average had significantly higher mean number of yearly codes (1.03) than conditions not included in QOF (0.32; $p<0.0001$).

The number of codes was higher in the first year after diagnosis than in subsequent years for almost all conditions, except for secondary bowel or pleural malignancy and diabetic eye disease, for which code frequency was higher on average after the first year of diagnosis. QOF conditions on average had lower ratios of codes in the first compared to subsequent years than non-QOF conditions (4.8 versus 5.7 times higher in year 1). However, diseases representing major cardiovascular events, such as myocardial infarction, were coded much more frequently in the first year from diagnosis than in subsequent years (appendix Figure A2 and Figure A3).

Variation in coding frequency by GP practice

There was a wide range in the mean yearly number of codes per condition between GP practices, with higher code frequency for QOF compared to non-QOF conditions (appendix Figure A4). There was a strong correlation ($r = 0.88$) between GP practice mean code frequency for QOF and non-QOF conditions (Figure 1). There was no observed trend according to the GP practice-level IMD decile (appendix Figure A5).

Figure 1: Scatterplot of mean yearly number of codes following diagnosis for QOF versus non-QOF conditions for each GP practice

We calculated the expected counts of codes for new diseases in year 1 and year 2 following diagnosis, predicted from negative binomial regression models. Expected mean counts per

For peer review only

condition at GP practice level showed substantially less variation compared to the observed mean counts for both QOF and non-QOF conditions in year 1 and year 2 (appendix Figure A1) indicating substantial residual practice level variation independent of patient socio-demographic factors.

Variation in disease frequency by socio-demographics and over time

We found significant associations between code frequency in year 1 and year 2 following diagnosis with patient socio-demographic factors and calendar year of diagnosis for both QOF and non-QOF diseases from mixed effects negative binomial regression, after adjustment for number of pre-existing conditions (Figures 2 and 3, and appendix Tables A3 – A6). Inclusion of GP practice fixed effects in the regression models resulted in very similar coefficients for patient sociodemographic factors, and a significantly lower AIC indicating better model fit and so results are presented including practice-level effects.

Associations with QOF conditions

Younger patients tended to have a higher frequency of codes in the first year following diagnosis compared to older patients (Figure 1). However, in the second year from diagnosis, there was a U-shaped relationship with age, with the youngest and oldest age groups having the lowest rate of codes. Males had on average a small 3% increase (95% CI: 1.03 – 1.03) in the incidence rate of codes in year 1 and 11% (95% CI: 1.11 – 1.12) increase in year 2 compared with females. There was a strong relationship with ethnicity, with people of non-White ethnicities having lower rates of code frequency than people of White ethnicity in year 1, but higher rates in year 2. There was a strong trend towards higher code frequency in year 1 and year 2 with decreasing levels of deprivation.

Associations with non-QOF conditions

For conditions not included in QOF, relationships were more consistent across year 1 and year 2 following diagnosis (Figure 2). The 18–40-year age group had the highest rate of codes in both year 1 and year 2, with only small differences between other age groups. There was no difference in the rate of codes in males and females in year 1, but males had a lower rate of codes in year 2. Lower rates of codes were found in people of non-White ethnicities compared to people of White ethnicity, except for South Asian ethnicity in year 2. Similar to

For peer review only

QOF conditions, there was a strong trend towards higher code rates in year 1 and year 2 with decreasing deprivation.

Associations with calendar year

For both QOF and non-QOF conditions, code rates were similar for conditions diagnosed in 2016 and 2017 compared with 2015 (Figures 1 and 2). For codes in year 1, rates for conditions diagnosed in 2018 were similar to 2015, but rates for diseases diagnosed in 2019 were 5% and 6% lower than 2015 for QOF and non-QOF conditions, respectively. For codes in year 2, rates were significantly lower in 2018 (9% and 9% lower for QOF and non-QOF, respectively) and 2019 (21% and 21% lower for QOF and non-QOF, respectively) compared to 2015.

Adjustment for total number of consultations

A sensitivity analysis was used to adjust for total number of consultations in year 1 or year 2 from diagnosis (Tables A3-A6). Total number of consultations in each year were strongly linked to the rate of codes. For newly diagnosed QOF conditions, the associations with age, sex and ethnicity in years 1 and 2 remained significant after adjustment (Tables A3-A4). However, the association with deprivation was attenuated, although there remained an association with higher rates of codes with lower deprivation in year 2. For newly diagnosed non-QOF conditions, after adjustment for consultations, age and ethnicity remained significantly associated, but males had significantly higher rates of codes than females (Tables A5-A6). Associations with deprivation were attenuated, but there remained a small but significant association in year 2.

Figure 2: Associations of rate of codes in year one and year two following diagnosis with patient characteristics and calendar year, for conditions included in the Quality and Outcomes Framework (QOF)

Figure 3: Associations of rate of codes in year one and year two following diagnosis with patient characteristics and calendar year, for conditions not included in the Quality and Outcomes Framework (QOF)

For peer review only

Discussion

With an increased use of methods incorporating information on disease sequence, we need to better understand the structure and frequency of occurrence of diagnostic codes within the primary care EHR. Our study demonstrates significant associations in the frequency of codes for newly diagnosed conditions according to patient socio-demographic factors, GP practice, disease inclusion in QOF, and calendar year.

Patient socio-demographics

Patient characteristics including age, sex and ethnicity were strongly linked to code frequency, although associations were inconsistent across QOF and non-QOF conditions, and for QOF conditions, were not consistent across the first and second year from diagnosis. People of non-White ethnicity, for example, had lower code rates for QOF conditions in year 1, but higher in year 2, compared to people of White ethnicity. We found consistent patterns with deprivation, with lower code frequency in people living in more deprived areas. A sensitivity analysis adjusting for total number of consultations attenuated the association with deprivation, suggesting that the relationship of code frequency with deprivation was partially explained by total primary care contacts.

These findings likely point to differences in the mix of conditions between patient groups, healthcare seeking behaviours, or access to care. For example, people living in areas of socio-economic deprivation may be less likely to attend for screening, preventive care and ongoing management of chronic diseases. Previous research also suggests that although rates of appointments are similar across deciles of socioeconomic deprivation,[22] the rate of missed appointments increases and consultation length decreases with increasing deprivation, which may impact on code frequency for these groups, rather than indicating differences in healthcare need.[23,24]

GP practice

Substantial variation was found in the frequency of codes between GP practices, which persisted after accounting for differences in patient mix in terms of age, sex, deprivation, ethnicity, number of chronic conditions and in year of diagnosis. Although this may indicate unmeasured confounding in the characteristics of patients between practices, it likely represents policies and practices that influence coding which vary between organisations and clinicians.[11] For example, some GP practices may be more rigorous about coding data in

For peer review only

clinical consultations and in correspondence from specialist services. Previous research has suggested that clinicians are more similar to those in the same practice than they are to clinicians in different practices with respect to treatment and diagnostic decisions.[25] Variation between clinicians in coding practices is likely to be significant both within and between practices, but this information was not accessible for the study, and its analysis would introduce multiple hierarchical dependencies outside the scope of this work. Future work could consider individual clinician effects on coding practices in the her.

QOF and non-QOF conditions

Code frequency was significantly higher for conditions included in QOF compared to conditions not included. Previous research has highlighted changes to policies and procedures within GP practices to meet targets, including improved disease registries, which may lead to an increased likelihood of a code being entered for a given condition.[14] We found substantial variation between GP practices in the mean code frequency for QOF conditions, but interestingly, this was strongly correlated (r=0.88) with code frequency for non-QOF conditions, suggesting that practice-level effects impact on coding across all conditions, rather than specifically those incentivised by QOF. However, it is not possible in our study to determine whether differences in code frequency between QOF and non-QOF conditions are explained by greater healthcare need and contacts for QOF conditions or are explained by higher likelihood of coding when a patient presents.

Calendar year

Accounting for calendar time in analyses of patient trajectories is a methodological concern, as the further back in time in the medical record, particularly before the advent of the EHR and QOF, the greater the chance that coding practices, and even disease categories, vary.[26] Although our study started relatively recently in 2015, and we cannot infer code frequency before this time, we found consistency in code frequency over a short time-span from 2015-2017. The decline in year 1 codes in 2019, and year 2 codes in 2018 and 2019 likely relates to the impact of the COVID-19 pandemic which impacted significantly on health services in England from March 2020.[27] Previous studies have shown reductions in patients presenting with particular conditions, and a reduction in appointment numbers in primary and secondary healthcare in England.[28] Analyses reliant on coding frequency should therefore consider using calendar year in addition to patient age in modelling patient trajectories, or limiting analyses to defined time period.

For peer review only

Strengths and limitations

A strength of our study is the inclusion of a large number of patients from a representative sample of primary care in England which will make our findings generalisable to the national population.[15] We included only patients with newly incident diseases to minimise potential confounding from diseases diagnosed historically, some of which might no longer be active. We also only included patients with continuous follow-up over the study period and with at least one year of full practice registration. We also excluded patients who died less than one year from a new diagnosis, which may impact on disease frequency estimates for disease which have poor survival. We considered using annualised rates for those with less than a full year of follow-up, but this resulted in very high annualised counts for some individuals with short follow-up, and might introduce additional bias if patients were to seek out care in advance of re-registering at another GP practice.

Our study has focussed on structured healthcare data, whereas much of the consultation is recorded as unstructured 'free-text'.[29] Although unstructured primary care data contains much richer information on the details of a presentation that may not be fully reflected in the coded entries, this information is not currently available from CPRD, but research in future could examine the agreement between structured and unstructured primary care EHR data. We stratified conditions according to QOF status given our hypothesis that it may influence coding frequency. However, we also found variation within categories; for example, polymyalgia rheumatica and motor neurone disease, which are not included in QOF, had high number of yearly codes, whereas cardiovascular events such as Transient Ischaemic Attack, included in QOF, had low yearly codes. Given the general, comparative nature of this paper, and its aim to examine relationships over many conditions, a condition-specific analysis of coding frequency was out of scope.

Implications

Our findings have implications for researchers using code sequences recorded in primary care structured data. The frequency of repeated diagnostic codes relate to patient and condition-specific factors, coding incentives and practice-level factors. Although we cannot determine if these findings represent disease burden and healthcare need, it is likely that biases in coding operate at various levels. Specific approaches to reduce the impact of bias will depend on the methodology, but our work does suggest general principles.

For peer review only

Firstly, to consider the potential for bias within the data source and whether stratification may reduce it, for example, by selecting a smaller number of healthcare organisations or a narrower time period. Secondly, to consider adjustment or inclusion of patient, condition, GP practice and calendar year variables within analytical models. However, such an approach is not always recommended, particularly if prediction is the aim, as inclusion of factors such as ethnicity in algorithms may reinforce existing bias.[30] In NLP, text style transfer is often used as a method to control for different styles of writing, which may have relevance to approaches to account for the different coding styles of clinicians.[31] However, these approaches are complicated within the EHR as people are likely to see multiple different clinicians over time, with a small set of codes recorded at each visit. Finally, it is vital that generated representations or predictions from modelling are evaluated in different patient subgroups.

Conclusion

Our study found significant variation in the frequency of diagnostic codes recorded in the primary care medical record after diagnosis, related to patient socio-demographics, coding incentives and GP practice and a significant reduction in the frequency of codes associated with the onset of the COVID-19 pandemic. Methods using sequences of recurrence of codes in the medical record should consider accounting for these factors to reduce the risk of bias.

Funding

This research is funded through a clinical PhD fellowship awarded to TB from the Wellcome Trust 4i programme at Imperial College London (grant number N/A). JC acknowledge support from the Wellcome Trust (grant number N/A). MB acknowledges support from EPSRC grant EP/N014529/1 supporting the EPSRC Centre for Mathematics of Precision Healthcare. TW, AM and PA acknowledge support from the National Institute for Health and Care Research (NIHR) under the Applied Research Collaboration (ARC) Northwest London (grant number N/A). The views expressed in this publication are those of the authors and not necessarily those of the NHS, the NIHR, the Wellcome Trust or the Department of Health and Social Care.

Competing interests

The authors have no competing interests to declare

For peer review only

Contributor statement

TB conceptualised the study, conducted the data management and formal analysis and wrote the first draft of the manuscript. All authors contributed to the study design, methodology, interpretation of findings and reviewing and editing the manuscript. TB is the guarantor and accepts full responsibility for the work and the conduct of the study, had access to the data, and controlled the decision to publish. The corresponding author attests that all listed authors meet authorship criteria and that no others meeting the criteria have been omitted.

Data sharing

The data used in this study are not publicly available as access is subject to approval processes. More information is available from CPRD: https://cprd.com/research-applications

Ethics approval

Data access to the Clinical Practice Research Datalink (CPRD) and ethical approval was granted by CPRD's Research Data Governance Process on 28th April 2022 (Protocol reference: 22_001818).

Acknowledgements

Data management was provided by the Big Data and Analytical Unit (BDAU) at the Institute of Global Health Innovation (IGHI).

References

1. Li, Y. *et al.* BEHRT: Transformer for Electronic Health Records. *Sci Rep* **10**, 7155 (2020).
2. Solares, J. R. A. *et al.* Transfer Learning in Electronic Health Records through Clinical Concept Embedding. 1–14 (2021).
3. Altuncu, M. T., Mayer, E., Yaliraki, S. N. & Barahona, M. From free text to clusters of content in health records: an unsupervised graph partitioning approach. *Applied Network Science* **4**, 2 (2019).
4. Kraljevic, Z. *et al.* Multi-domain clinical natural language processing with MedCAT: The Medical Concept Annotation Toolkit. *Artif Intell Med* **117**, 102083 (2021).

For peer review only

5. Rasmy, L., Xiang, Y., Xie, Z., Tao, C. & Zhi, D. Med-BERT: pretrained contextualized embeddings on large-scale structured electronic health records for disease prediction. *npj Digit. Med.* **4**, 1–13 (2021).

6. Choi, E. *et al.* Multi-layer representation learning for medical concepts. *Proceedings of the ACM SIGKDD International Conference on Knowledge Discovery and Data Mining* **13-17-Augu**, 1495–1504 (2016).

7. Choi, E. *et al.* RETAIN: An Interpretable Predictive Model for Healthcare using Reverse Time Attention Mechanism. Preprint at https://doi.org/10.48550/arXiv.1608.05745 (2017).

8. Cai, X. *et al.* Medical Concept Embedding with Time-Aware Attention. Preprint at http://arxiv.org/abs/1806.02873 (2018).

9. Busija, L., Lim, K., Szoeke, C., Sanders, K. M. & McCabe, M. P. Do replicable profiles of multimorbidity exist? Systematic review and synthesis. *European Journal of Epidemiology* **34**, 1025–1053 (2019).

10. Bhattacharya, M., Jurkovitz, C. & Shatkay, H. Co-occurrence of medical conditions: Exposing patterns through probabilistic topic modeling of snomed codes. *Journal of biomedical informatics* **82**, 31–40 (2018).

11. Verheij, R. A., Curcin, V., Delaney, B. C. & McGilchrist, M. M. Possible Sources of Bias in Primary Care Electronic Health Record Data Use and Reuse. *J Med Internet Res* **20**, e185 (2018).

12. Bots, S. H., Groenwold, R. H. H. & Dekkers, O. M. Using electronic health record data for clinical research: a quick guide. *European Journal of Endocrinology* **186**, E1–E6 (2022).

For peer review only

13. Forbes, L. J., Marchand, C., Doran, T. & Peckham, S. The role of the Quality and Outcomes Framework in the care of long-term conditions: a systematic review. *Br J Gen Pract* **67**, e775 (2017).

14. Dzudie, A. *et al.* MMM17-Cameroon, analysis and opportunities-Sub-Saharan Africa. *European heart journal supplements : journal of the European Society of Cardiology* (2019) doi:10.1093/eurheartj/suz081.

15. Wolf, A. *et al.* Data resource profile: Clinical Practice Research Datalink (CPRD) Aurum. *International Journal of Epidemiology* **48**, 1740–1740g (2019).

16. Head, A. *et al.* Inequalities in incident and prevalent multimorbidity in England, 2004–19: a population-based, descriptive study. *The Lancet Healthy Longevity* **2**, e489–e497 (2021).

17. Kuan, V. *et al.* A chronological map of 308 physical and mental health conditions from 4 million individuals in the English National Health Service. *The Lancet Digital Health* **1**, e63–e77 (2019).

18. Ministry of Housing & Communities & Local Government. English indices of deprivation 2019. https://www.gov.uk/government/statistics/english-indices-of-deprivation-2019.

19. Dean, C. B. Testing for Overdispersion in Poisson and Binomial Regression Models. *Journal of the American Statistical Association* **87**, 451–457 (1992).

20. Dunn, P. K. & Smyth, G. K. Randomized Quantile Residuals. *Journal of Computational and Graphical Statistics* **5**, 236–244 (1996).

21. Feng, C., Li, L. & Sadeghpour, A. A comparison of residual diagnosis tools for diagnosing regression models for count data. *BMC Med Res Methodol* **20**, 175 (2020).

22. Fisher, R., Dunn, P., Asaria, M. & Thorlby, R. Comparing general practice in areas of high and low socioeconomic deprivation in England. 30.

For peer review only

23. Ellis, D. A., McQueenie, R., McConnachie, A., Wilson, P. & Williamson, A. E. Demographic and practice factors predicting repeated non-attendance in primary care: a national retrospective cohort analysis. *The Lancet Public Health* **2**, e551–e559 (2017).

24. Gopfert, A., Deeny, S. R., Fisher, R. & Stafford, M. Primary care consultation length by deprivation and multimorbidity in England: an observational study using electronic patient records. *Br J Gen Pract* **71**, e185–e192 (2021).

25. Jong, J. de, Groenewegen, P. & Westert, G. Medical practice variation: does it cluster within general practitioners' practices? in *Morbidity, Performance and Quality in Primary Care* (CRC Press, 2006).

26. Gluckman, P. D. Evolving a definition of disease. *Archives of Disease in Childhood* **92**, 1053 (2007).

27. Majeed, A., Maile, E. J. & Bindman, A. B. The primary care response to COVID-19 in England's National Health Service. *J R Soc Med* **113**, 208–210 (2020).

28. Clarke, J., Beaney, T., Majeed, A., Darzi, A. & Barahona, M. Identifying naturally occurring communities of primary care providers in the English National Health Service in London. *BMJ Open* **10**, (2020).

29. Tayefi, M. *et al.* Challenges and opportunities beyond structured data in analysis of electronic health records. *WIREs Computational Statistics* **13**, e1549 (2021).

30. Obermeyer, Z., Powers, B., Vogeli, C. & Mullainathan, S. Dissecting racial bias in an algorithm used to manage the health of populations. *Science* **366**, 447–453 (2019).

31. Jin, D., Jin, Z., Hu, Z., Vechtomova, O. & Mihalcea, R. Deep Learning for Text Style Transfer: A Survey. Preprint at http://arxiv.org/abs/2011.00416 (2021).

Figure 1 legend:

Note: different ranges used in each axis

For peer review only

Figure 2 legend:

Note: Points represent estimates of the incidence rate ratio and bars represent 95% confidence intervals from negative binomial regression models. Corresponding values and coefficients for pre-existing QOF and non-QOF conditions are given in appendix Tables A3 and A4.

Figure 3 legend:

Note: Points represent estimates of the incidence rate ratio and bars represent 95% confidence intervals from negative binomial regression models. Corresponding values and coefficients for pre-existing QOF and non-QOF conditions are given in appendix Tables A5 and A6.

For peer review only

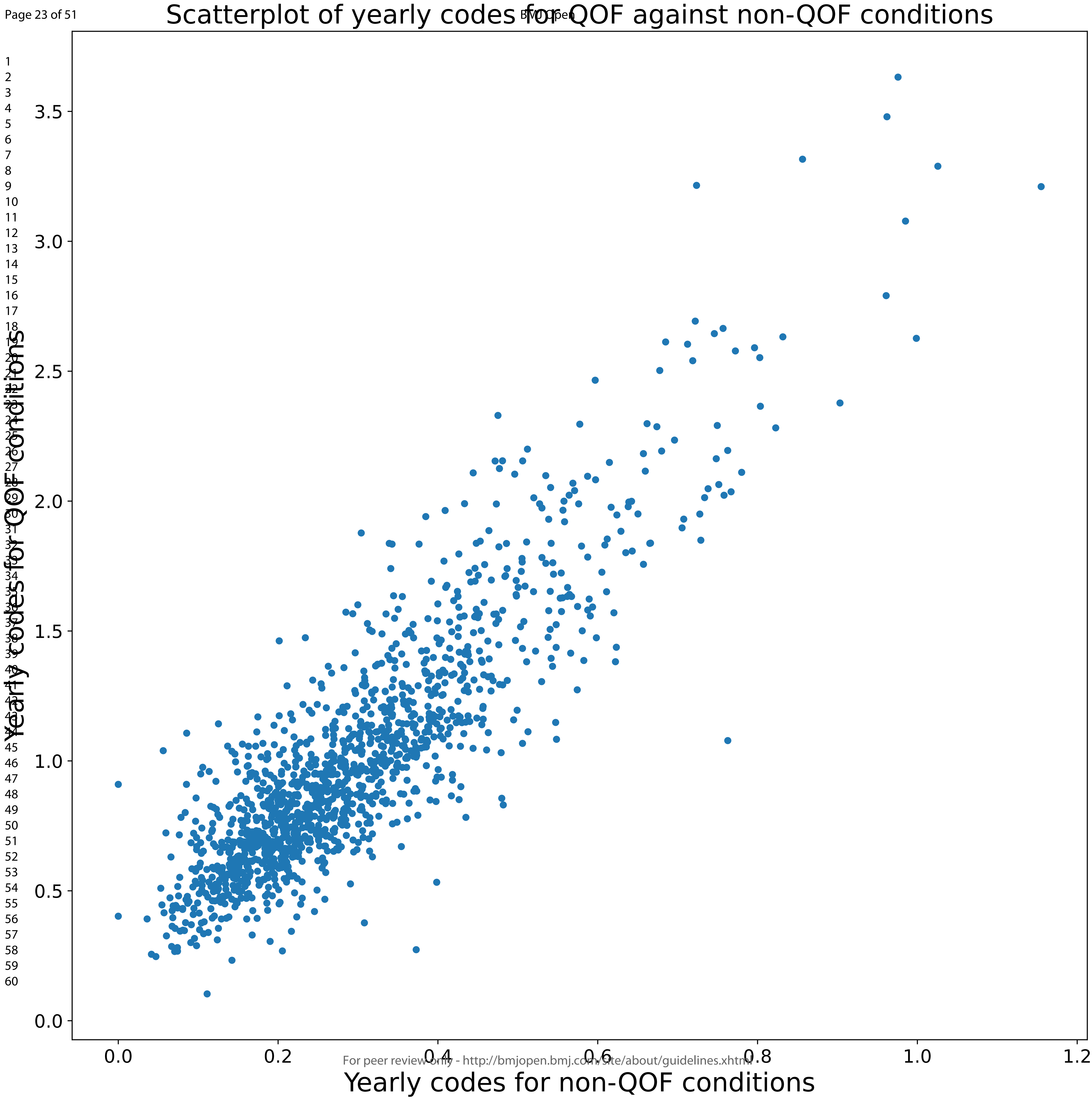
Scatterplot of yearly codes for QOF against non-QOF conditions
Yearly codes for QOF conditions
Yearly codes for non-QOF conditions
0.0
0.5
1.0
1.5
2.0
2.5
3.0
3.5
0.2
0.4
0.6
0.8
1.0
1.2

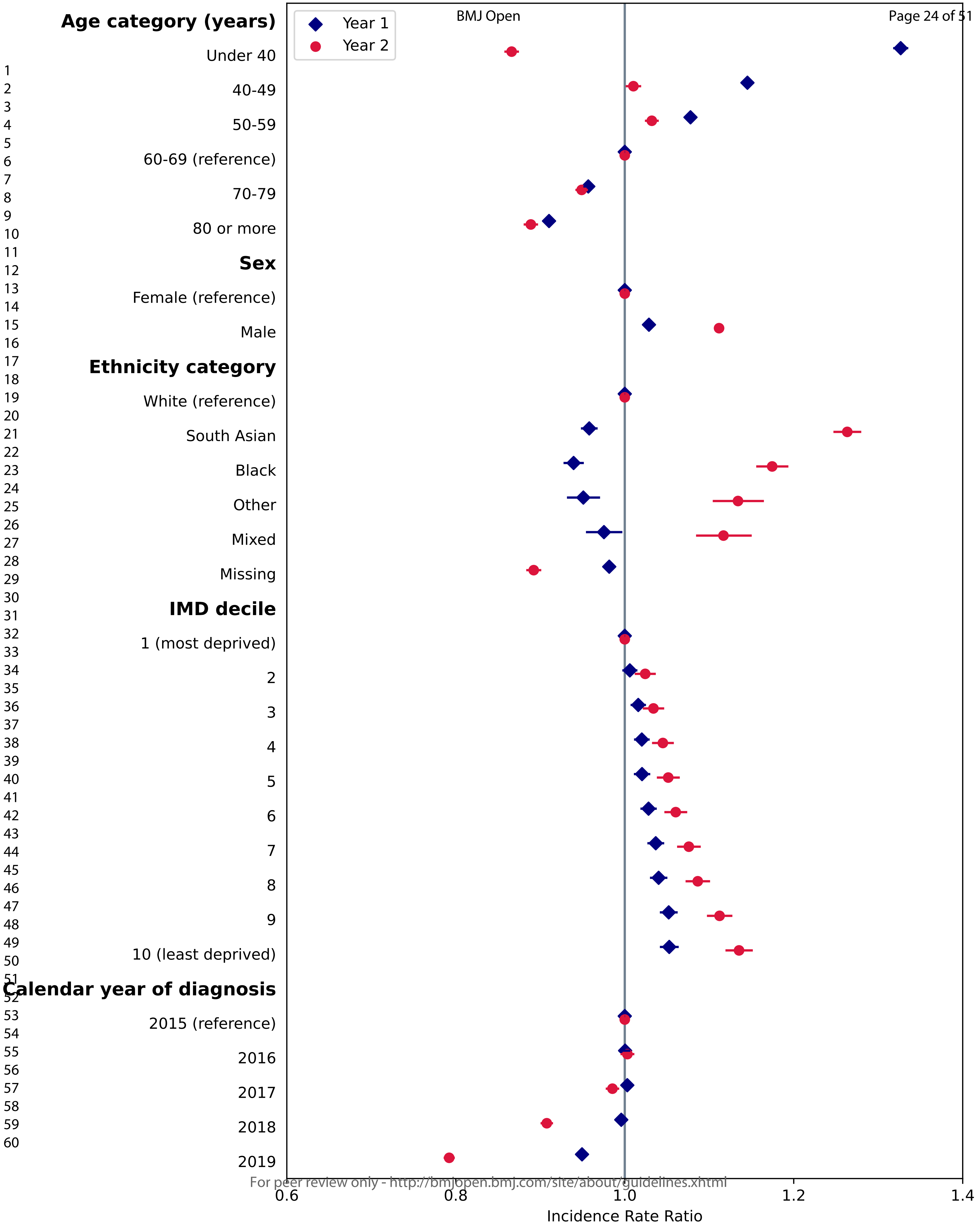
Year 1
Year 2
Age category (years)
Under 40
40-49
50-59
60-69 (reference)
70-79
80 or more
Sex
Female (reference)
Male
Ethnicity category
White (reference)
South Asian
Black
Other
Mixed
Missing
IMD decile
1 (most deprived)
2
3
4
5
6
7
8
9
10 (least deprived)
Calendar year of diagnosis
2015 (reference)
2016
2017
2018
2019
0.6
0.8
1.0
1.2
1.4
Incidence Rate Ratio

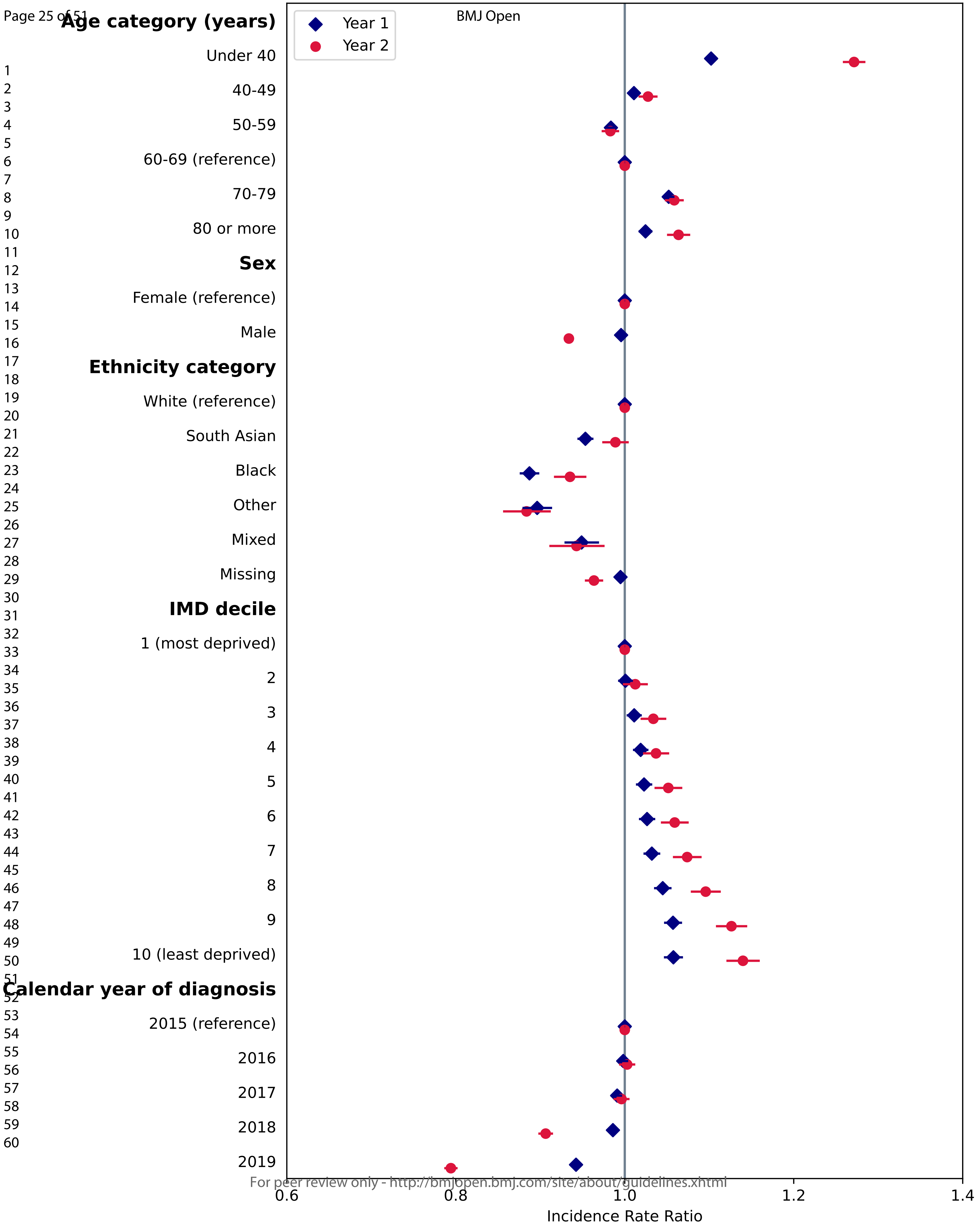
Age category (years)
Under 40
40-49
50-59
60-69 (reference)
70-79
80 or more
Sex
Female (reference)
Male
Ethnicity category
White (reference)
South Asian
Black
Other
Mixed
Missing
IMD decile
1 (most deprived)
2
3
4
5
6
7
8
9
10 (least deprived)
Calendar year of diagnosis
2015 (reference)
2016
2017
2018
2019
Year 1
Year 2
0.6
0.8
1.0
1.2
1.4
Incidence Rate Ratio

Appendix

Determinants of disease code frequency in the primary care electronic healthcare record: a retrospective cohort study

Beaney T[1,2] (0000-0001-9709-7264), Clarke J[2], Salman D[1,3], Woodcock T[1], Majeed A[1], Barahona M[2], Aylin P[1]

1. Department of Primary Care and Public Health, Imperial College London, London, W6 8RP, United Kingdom
2. Centre for Mathematics of Precision Healthcare, Department of Mathematics, Imperial College London, London, SW7 2AZ, United Kingdom
3. MSk Lab, Department of Surgery and Cancer, Faculty of Medicine, Imperial College London, London, UK

Corresponding Author:

Dr Thomas Beaney

Department of Primary Care and Public Health, Imperial College London, London, W6 8RP, United Kingdom

Email: thomas.beaney@imperial.ac.uk

For peer review only

Patients were included with continuous registration dates between 1st January 2014 and 31st December 2020. The 1st January 2014 was chosen to allow for a full one year of registration at a GP practice prior to follow-up, to reduce the potential impact of bias from newly registered patients having pre-existing conditions coded for the first time at their new practice. The end date of 31st December 2020 was chosen to provide at least one full year of follow-up for conditions newly diagnosed in 2019. Patients were followed up until the earliest date of death, deregistration and latest date of data extraction from their practice, if after 31st December 2020. The earliest possible censoring date for a patient was 1st January 2021 and the last date of follow-up for a patient was 21st March 2022.

Chronic conditions

Diseases were mapped using code lists developed for the CALIBER study, and adapted for use in multimorbidity in CPRD Aurum.[1,2] We reviewed the codes in these lists, and made amendments to the code lists for diabetes, to remove Type 1 and Type 2 codes from the other/unspecified code list. We added chronic primary pain to the set of included conditions and created a new code list. Previous studies of multimorbidity in primary care settings have found a high prevalence and burden of chronic pain.[3,4] However, in order to avoid double counting of pain related to another chronic condition included, we excluded secondary causes, and included only primary pain conditions.

Assignment to QOF

Diseases were classified as included or not included in QOF by two clinicians with experience working as GPs: TB and DS. The first QOF year in 2004/2005 included eleven diseases, with new conditions added in subsequent years.[5] Rheumatoid arthritis was added to QOF in 2013/2014, but there were no subsequent additions of any of the diseases included in this study.[6] However, hypothyroidism was included in QOF from its start until 2014/15 when it was removed.[7] The thyroid disease category from CALIBER included codes for both hypothyroidism and hyperthyroidism. We therefore excluded the thyroid disease category from comparisons of QOF to avoid any carry-over effect from prior inclusion in QOF, and dilution from non-hypothyroid conditions. The following QOF conditions from 2014/15 to 2019/20 were included:

1. Coronary Heart Disease
2. Left Ventricular Dysfunction / Heart Failure (from 2006)

For peer review only

3. Stroke (and TIA from 2006)
4. Hypertension
5. Diabetes
6. COPD
7. Epilepsy
8. Cancer
9. Mental Health
10. Asthma
11. Dementia
12. Depression
13. CKD
14. Atrial fibrillation
15. Obesity
16. Learning disabilities
17. Palliative care
18. Smoking
19. Cardio-vascular disease (primary prevention)
20. Peripheral Arterial Disease (PAD)
21. Osteoporosis
22. Rheumatoid arthritis

For analyses of counts per calendar year, the total counts of disease codes were calculated for the first and second year from diagnosis. Counts were stratified according to whether a condition was included in QOF. A patient was included for a given calendar year if they had at least one QOF or non-QOF condition diagnosed in that year, as shown in Table A1.

For peer review only

Appendix Table A1: example of the stratification of condition and calendar year for each newly diagnosed condition for three hypothetical patients

Patient	Age	Condition	Calendar year	Count in year one	Count in year two
1	67	QOF	2015	0	0
1	68	QOF	2016	2	0
1	70	QOF	2018	4	2
1	67	Non-QOF	2015	1	1
2	28	Non-QOF	2019	1	2
3	52	QOF	2017	5	4
3	52	Non-QOF	2017	2	2

For peer review only

For peer review only - http://bmjopen.bmj.com/site/about/guidelines.xhtml

Statistical analyses

Mixed effects negative binomial models were constructed. We considered use of a zero-inflated model, but coefficients from the logit and negative binomial components of the model were similar, and so in the interests of interpretable findings, the more parsimonious negative binomial model was selected.

Equation for the mixed effects negative binomial regression model, including fixed effects for calendar year and GP practice and random effects for patient:

$$log(y_{i,j}) = \beta_0 + \beta_1 age_{i,j} + \beta_2 gender_{i,j} + \beta_3 ethnicity_{i,j} + \beta_4 IMD_{i.j} + \beta_5 year_{i.j} + \beta_6 GP_{i.j} + u_j$$

where *i* represents QOF or non-QOF conditions newly diagnosed in patient *j* and $y_{i,j}$ is the count of codes in the given year.

For peer review only

Appendix Table A2: distribution of yearly codes over the whole follow-up period for each condition, ordered by median

Disease	5th centile	Median	95th centile	Mean	Standard deviation
Diabetes Mellitus_other or not specified	0.00	2.99	6.88	3.08	2.22
Polymyalgia Rheumatica	0.00	1.05	6.32	1.82	2.29
Motor neurone disease	0.00	0.95	12.15	2.86	5.41
Dementia	0.00	0.93	4.36	1.39	1.80
Type 2 Diabetes Mellitus	0.00	0.89	4.59	1.41	1.73
Type 1 Diabetes Mellitus	0.00	0.88	6.31	1.71	2.41
Depression	0.00	0.83	4.54	1.36	1.76
COPD	0.00	0.77	3.77	1.17	1.43
Heart failure	0.00	0.73	5.48	1.46	2.21
Rheumatoid Arthritis	0.00	0.70	5.50	1.43	2.23
Primary Malignancy_Mesothelioma	0.00	0.67	9.16	1.78	3.18
Primary Malignancy_Pancreas	0.00	0.67	13.41	2.63	5.12
Primary Malignancy_Brain	0.00	0.66	10.60	2.15	3.96
Primary Malignancy_Oesophageal	0.00	0.64	10.86	2.44	4.95
Myasthenia gravis	0.00	0.62	5.61	1.48	2.66
Multiple sclerosis	0.00	0.59	5.63	1.40	2.41
Parkinson's disease	0.00	0.59	4.52	1.20	1.77
Vitamin B12 deficiency anaemia	0.00	0.56	4.60	1.24	1.67
Bipolar affective disorder and mania	0.00	0.56	4.99	1.30	2.15
Plasma Cell Malignancy	0.00	0.54	10.32	2.15	4.67
Hypertension	0.00	0.54	2.95	0.88	1.12
Atrial Fibrillation	0.00	0.51	3.47	0.97	1.47
Primary Malignancy_Prostate	0.00	0.51	6.11	1.46	2.48
Intellectual disability	0.00	0.49	5.19	1.47	1.91
Primary Malignancy_Lung	0.00	0.45	8.17	1.73	3.55
Primary Malignancy_Biliary Tract	0.00	0.45	8.96	1.89	4.73
Giant Cell arteritis	0.00	0.44	5.73	1.36	2.47
Crohn's disease	0.00	0.42	5.41	1.24	2.32
Primary Malignancy_Breast	0.00	0.39	5.25	1.21	2.47
Hodgkin Lymphoma	0.00	0.38	5.41	1.24	2.55
Ulcerative colitis	0.00	0.38	4.27	1.00	1.87
Primary Malignancy_Oropharyngeal	0.00	0.37	6.84	1.44	2.95
Non-Hodgkin Lymphoma	0.00	0.37	5.52	1.22	2.53
Leukaemia	0.00	0.37	5.19	1.17	2.58
Secondary Malignancy_Brain	0.00	0.37	7.68	1.45	2.74
Stroke_not otherwise specified	0.00	0.34	2.11	0.59	0.89
Idiopathic Intracranial Hypertension	0.00	0.34	3.81	0.92	1.76
Thyroid Disease	0.00	0.33	2.56	0.68	1.16
Asthma	0.00	0.32	2.33	0.63	0.99
Primary Malignancy_Stomach	0.00	0.32	6.93	1.45	3.30
Chronic primary pain	0.00	0.32	3.23	0.79	1.34
Coronary Heart Disease (not otherwise specified)	0.00	0.31	2.02	0.56	0.85
Epilepsy	0.00	0.31	3.66	0.92	1.95

For peer review only - http://bmjopen.bmj.com/site/about/guidelines.xhtml

Psoriatic Arthritis	0.00	0.30	3.68	0.87	1.63
Chronic Fatigue Syndrome	0.00	0.29	3.22	0.76	1.31
Primary Malignancy_Bowel	0.00	0.29	5.25	1.15	2.88
Anxiety disorders	0.00	0.29	2.99	0.73	1.29
Primary Malignancy_Thyroid	0.00	0.28	4.05	0.88	1.76
Personality disorders	0.00	0.28	4.35	0.99	2.05
Schizophrenia	0.00	0.27	3.36	0.78	1.52
Primary Malignancy_Cervix	0.00	0.27	5.26	1.17	2.77
Autoimmune liver disease	0.00	0.26	3.63	0.85	1.82
Myelodysplastic Syndrome	0.00	0.26	4.88	1.15	2.95
Bronchiectasis	0.00	0.24	3.03	0.70	1.31
Hyperkinetic disorders	0.00	0.24	3.11	0.72	1.34
Primary Malignancy_Ovary	0.00	0.24	6.15	1.24	2.87
Primary Malignancy_Liver	0.00	0.23	3.64	0.95	2.99
Coeliac disease	0.00	0.23	2.13	0.52	0.85
Lupus Erythematosus	0.00	0.22	3.52	0.83	1.87
Myocardial Infarction	0.00	0.21	2.44	0.58	1.04
Primary Malignancy_Bone	0.00	0.21	4.03	0.97	3.29
Secondary Malignancy_other	0.00	0.21	5.92	1.18	2.65
Peripheral Vascular Disease	0.00	0.20	2.73	0.75	2.53
Ankylosing spondylitis	0.00	0.20	3.00	0.69	1.47
Primary Malignancy_Bladder	0.00	0.20	4.38	0.90	2.05
Primary Malignancy_Testis	0.00	0.20	3.58	0.81	1.50
Sarcoidosis	0.00	0.19	3.36	0.72	1.53
Abdominal Hernia	0.00	0.19	1.55	0.40	0.68
Secondary Malignancy_Peritoneum	0.00	0.19	4.21	1.30	3.31
Scleroderma	0.00	0.19	3.00	0.71	1.88
Primary Malignancy_Melanoma	0.00	0.18	3.06	0.67	1.71
Gout	0.00	0.17	1.74	0.43	0.73
Barrett's oesophagus	0.00	0.16	1.40	0.35	0.57
Glomerulonephritis	0.00	0.16	3.26	0.74	1.69
Osteoporosis	0.00	0.15	1.52	0.38	0.65
Primary Malignancy_Uterus	0.00	0.15	3.90	0.81	2.16
Cirrhosis	0.00	0.15	2.88	0.63	1.40
Diabetic Eye Disease	0.00	0.15	1.61	0.40	0.68
Intracerebral haemorrhage	0.00	0.15	2.58	0.56	1.10
Primary Malignancy_Kidney	0.00	0.14	2.93	0.66	1.67
Dilated cardiomyopathy	0.00	0.14	1.99	0.46	0.93
Eating Disorders	0.00	0.14	4.03	0.84	2.38
Abdominal Aortic Aneurysm	0.00	0.00	1.35	0.26	0.58
Acne	0.00	0.00	1.26	0.30	0.50
Alcohol Misuse	0.00	0.00	0.94	0.20	0.66
Alcoholic liver disease	0.00	0.00	1.90	0.42	1.09
Allergic and chronic rhinitis	0.00	0.00	0.56	0.10	0.27
Alopecia areata	0.00	0.00	0.87	0.17	0.45
Anaemia_other	0.00	0.00	1.49	0.33	0.78
Angiodysplasia of colon	0.00	0.00	0.87	0.17	0.49
Anterior and Intermediate Uveitis	0.00	0.00	1.18	0.25	0.66
Aplastic anaemias	0.00	0.00	2.19	0.47	1.42

For peer review only - http://bmjopen.bmj.com/site/about/guidelines.xhtml

Asbestosis	0.00	0.00	0.96	0.20	0.65
Atrioventricular blocks	0.00	0.00	0.64	0.11	0.33
Autism and Asperger's syndrome	0.00	0.00	1.10	0.25	0.58
Autonomic Neuropathy	0.00	0.00	2.46	0.47	1.34
Benign Prostatic Hyperplasia	0.00	0.00	1.08	0.25	0.50
Benign essential tremor	0.00	0.00	1.11	0.22	0.53
Cardiomyopathy_other	0.00	0.00	1.94	0.41	0.90
Cataract	0.00	0.00	1.16	0.27	0.50
Cerebral Palsy	0.00	0.00	0.73	0.16	0.48
Chronic Cystitis	0.00	0.00	1.88	0.37	1.03
Chronic Kidney Disease	0.00	0.00	1.16	0.26	0.65
Chronic sinusitis	0.00	0.00	0.72	0.13	0.39
Chronic viral hepatitis	0.00	0.00	1.89	0.40	0.90
Collapsed vertebra	0.00	0.00	1.64	0.34	0.77
Congenital Septal Defect	0.00	0.00	1.21	0.24	0.62
Cystic Fibrosis	0.00	0.00	2.21	0.31	1.00
Dermatitis	0.00	0.00	0.76	0.15	0.43
Diabetic Neuropathy	0.00	0.00	1.62	0.38	1.44
Diaphragmatic hernia	0.00	0.00	0.81	0.17	0.38
Diverticular Disease	0.00	0.00	0.96	0.20	0.51
Down's syndrome	0.00	0.00	0.48	0.10	0.19
Dysmenorrhoea	0.00	0.00	0.78	0.15	0.38
Endometrial hyperplasia and hypertrophy	0.00	0.00	0.90	0.17	0.57
Endometriosis	0.00	0.00	2.08	0.44	1.06
Enteropathic arthropathy	0.00	0.00	1.28	0.38	0.99
Enthesopathy and synovial disorder	0.00	0.00	0.86	0.18	0.43
Fatty Liver	0.00	0.00	0.75	0.14	0.34
Fibromatosis	0.00	0.00	0.85	0.17	0.39
Folate deficiency anaemia	0.00	0.00	0.52	0.09	0.25
Gastritis and duodenitis	0.00	0.00	0.73	0.14	0.39
Gastro-oesophageal reflux disease	0.00	0.00	0.88	0.18	0.43
Glaucoma	0.00	0.00	1.46	0.31	0.62
HIV	0.00	0.00	2.07	0.41	0.92
Hearing loss	0.00	0.00	0.77	0.16	0.34
Hepatic failure	0.00	0.00	2.22	0.46	1.07
Hidradenitis suppurativa	0.00	0.00	1.92	0.43	1.11
Hyperparathyroidism	0.00	0.00	1.84	0.41	0.84
Hypersplenism	0.00	0.00	0.99	0.21	0.58
Hypertrophic Cardiomyopathy	0.00	0.00	2.23	0.49	1.00
Hypertrophic Nasal Turbinates	0.00	0.00	0.28	0.04	0.16
Hyposplenism	0.00	0.00	1.50	0.34	0.71
Immunodeficiencies	0.00	0.00	1.62	0.36	1.11
Intervertebral disc disorders	0.00	0.00	1.75	0.36	0.91
Irritable bowel syndrome	0.00	0.00	0.66	0.13	0.32
Ischaemic stroke	0.00	0.00	2.03	0.46	0.99
Left bundle branch block	0.00	0.00	0.77	0.15	0.39
Macular degeneration	0.00	0.00	1.16	0.25	0.71
Meniere's Disease	0.00	0.00	1.58	0.33	0.77
Migraine	0.00	0.00	1.21	0.25	0.61

For peer review only

Multiple valve disorder	0.00	0.00	0.49	0.09	0.33
Neuropathic Bladder	0.00	0.00	0.74	0.15	0.36
Nonrheumatic aortic valve disorders	0.00	0.00	1.42	0.31	0.68
Nonrheumatic mitral valve disorders	0.00	0.00	0.84	0.16	0.52
Obesity	0.00	0.00	0.71	0.15	0.44
Obsessive-compulsive disorder	0.00	0.00	2.55	0.56	1.21
Obstructive and reflux uropathy	0.00	0.00	1.10	0.23	0.63
Oesophageal varices	0.00	0.00	1.62	0.38	0.74
Osteoarthritis (excl spine)	0.00	0.00	1.53	0.34	0.70
Other haemolytic anaemias	0.00	0.00	3.09	0.62	1.64
Pancreatitis	0.00	0.00	2.00	0.44	1.09
Pericardial Effusion	0.00	0.00	1.12	0.21	0.56
Peripheral Neuropathy	0.00	0.00	1.22	0.26	0.81
Pleural effusion	0.00	0.00	1.55	0.32	0.90
Pleural plaque	0.00	0.00	0.74	0.14	0.48
Polycystic ovarian syndrome	0.00	0.00	0.86	0.20	0.34
Polycythaemia vera	0.00	0.00	2.49	0.54	1.30
Portal hypertension	0.00	0.00	0.91	0.18	0.46
Posterior Uveitis	0.00	0.00	1.46	0.33	1.02
Primary Malignancy_Multiple Sites	0.00	0.00	0.00	0.00	0.00
Primary Malignancy_Skin	0.00	0.00	1.30	0.31	0.78
Primary Malignancy_other	0.00	0.00	4.42	0.90	2.44
Primary Thrombocytopaenia	0.00	0.00	2.41	0.59	1.96
Primary pulmonary hypertension	0.00	0.00	1.62	0.32	1.00
Psoriasis	0.00	0.00	1.44	0.32	0.75
Pulmonary Fibrosis	0.00	0.00	2.38	0.53	1.34
Raynaud's syndrome	0.00	0.00	0.85	0.16	0.45
Retinal vascular occlusions	0.00	0.00	1.93	0.42	0.93
Rheumatic Valve Disorder	0.00	0.00	0.70	0.13	0.41
Right bundle branch block combinations	0.00	0.00	0.47	0.08	0.25
Rosacea	0.00	0.00	0.93	0.20	0.41
Scleritis and episcleritis	0.00	0.00	0.70	0.13	0.49
Seborrheic dermatitis	0.00	0.00	0.61	0.11	0.31
Secondary Malignancy_Adrenal Gland	0.00	0.00	1.68	0.42	1.01
Secondary Malignancy_Bone	0.00	0.00	4.78	0.93	2.34
Secondary Malignancy_Bowel	0.00	0.00	6.36	1.41	2.42
Secondary Malignancy_Liver	0.00	0.00	4.82	0.91	2.26
Secondary Malignancy_Lung	0.00	0.00	6.04	1.10	2.27
Secondary Malignancy_Lymph Nodes	0.00	0.00	2.40	0.40	1.31
Secondary Malignancy_Pleura	0.00	0.00	5.69	0.94	2.50
Secondary Thrombocytopaenia	0.00	0.00	0.89	0.19	0.48
Secondary polycythaemia	0.00	0.00	1.64	0.32	0.78
Secondary pulmonary hypertension	0.00	0.00	1.29	0.27	0.83
Sick sinus syndrome	0.00	0.00	0.79	0.14	0.40
Sickle Cell Disease	0.00	0.00	0.98	0.29	1.07
Sjogren's Syndrome	0.00	0.00	1.48	0.31	0.68
Sleep apnoea	0.00	0.00	0.92	0.19	0.43
Spina bifida	0.00	0.00	0.48	0.11	0.44
Spinal stenosis	0.00	0.00	2.34	0.50	1.06

For peer review only - http://bmjopen.bmj.com/site/about/guidelines.xhtml

Spondylolisthesis	0.00	0.00	1.22	0.23	0.63
Spondylosis	0.00	0.00	1.01	0.21	0.57
Stable Angina	0.00	0.00	1.62	0.37	0.78
Subarachnoid haemorrhage	0.00	0.00	2.41	0.51	1.05
Substance Misuse	0.00	0.00	1.42	0.32	1.34
Supraventricular tachycardia	0.00	0.00	1.55	0.35	0.78
Thalassaemia	0.00	0.00	0.31	0.05	0.19
Thrombophilia	0.00	0.00	0.75	0.15	0.53
Tinnitus	0.00	0.00	0.85	0.17	0.43
Transient ischaemic attack	0.00	0.00	1.56	0.35	0.70
Trigeminal neuralgia	0.00	0.00	2.16	0.47	1.05
Tubulo-interstitial nephritis	0.00	0.00	2.70	0.50	1.23
Unstable Angina	0.00	0.00	1.17	0.23	0.58
Urinary Incontinence	0.00	0.00	0.87	0.18	0.38
Venous thromboembolic disease (Excl PE)	0.00	0.00	1.85	0.41	1.05
Ventricular tachycardia	0.00	0.00	1.64	0.32	0.75
Visual impairment and blindness	0.00	0.00	0.73	0.13	0.31
Vitiligo	0.00	0.00	0.73	0.14	0.32

For peer review only

Appendix Figure A1: Boxplots of observed and expected mean yearly codes at a GP practice level for QOF conditions in year 1 (A) and year 2 (B) and non-QOF conditions in year 1 (C) and year 2 (D) following diagnosis

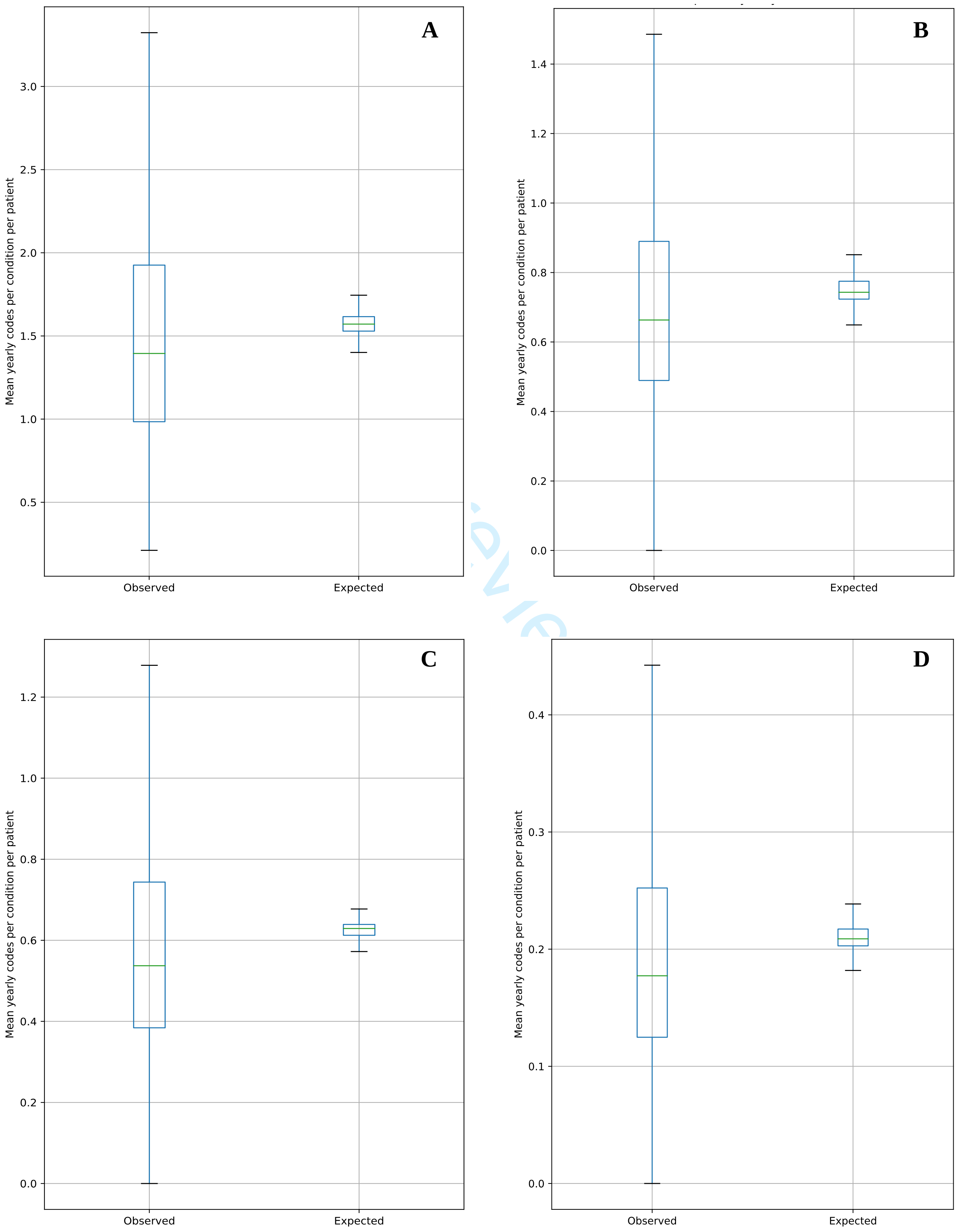

Appendix Figure A2: ratio of mean yearly codes in year 1 following diagnosis to subsequent years for QOF conditions

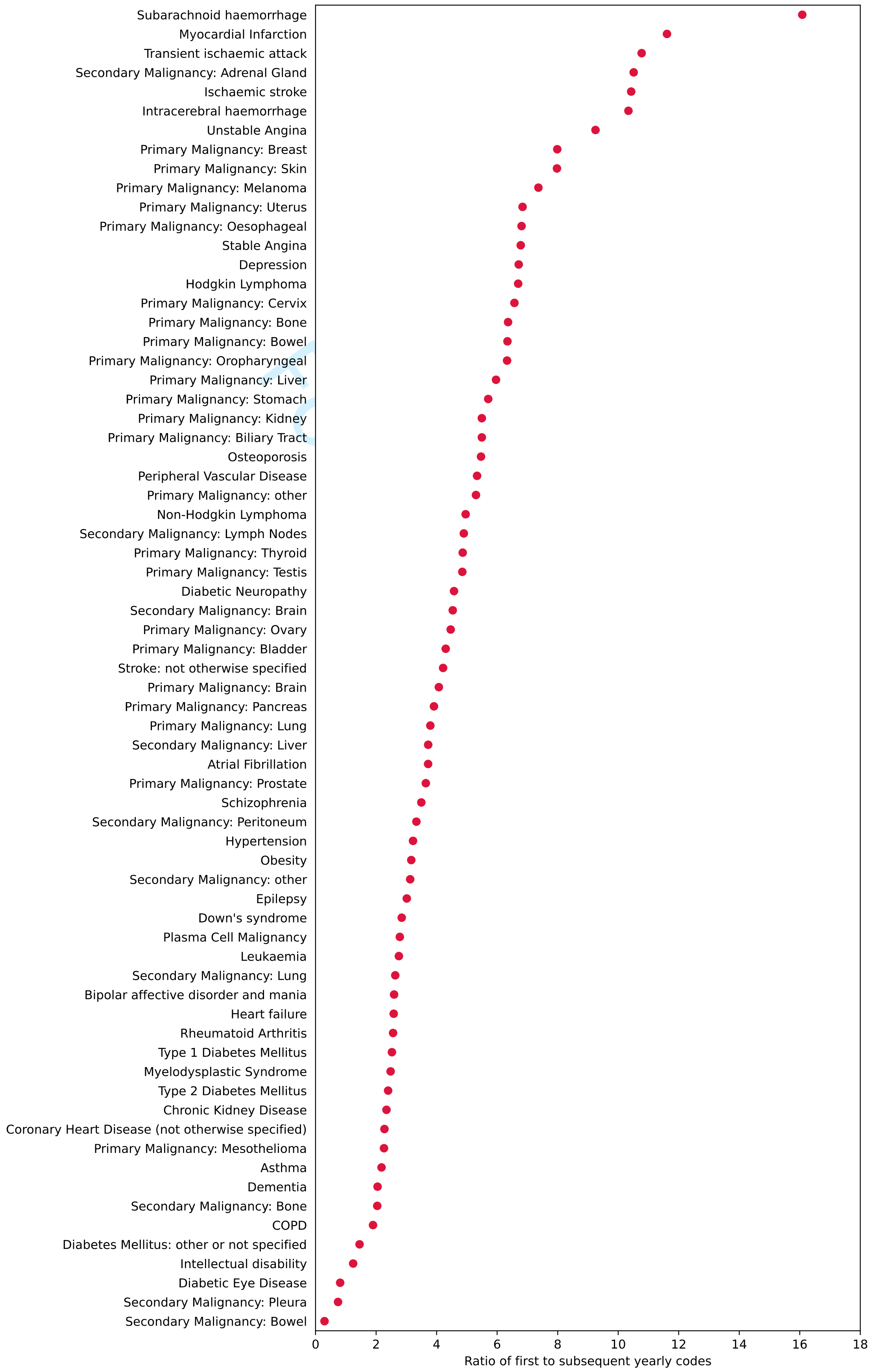

Appendix Figure A3: Ratio of mean yearly codes in year 1 following diagnosis to subsequent years for non-QOF conditions

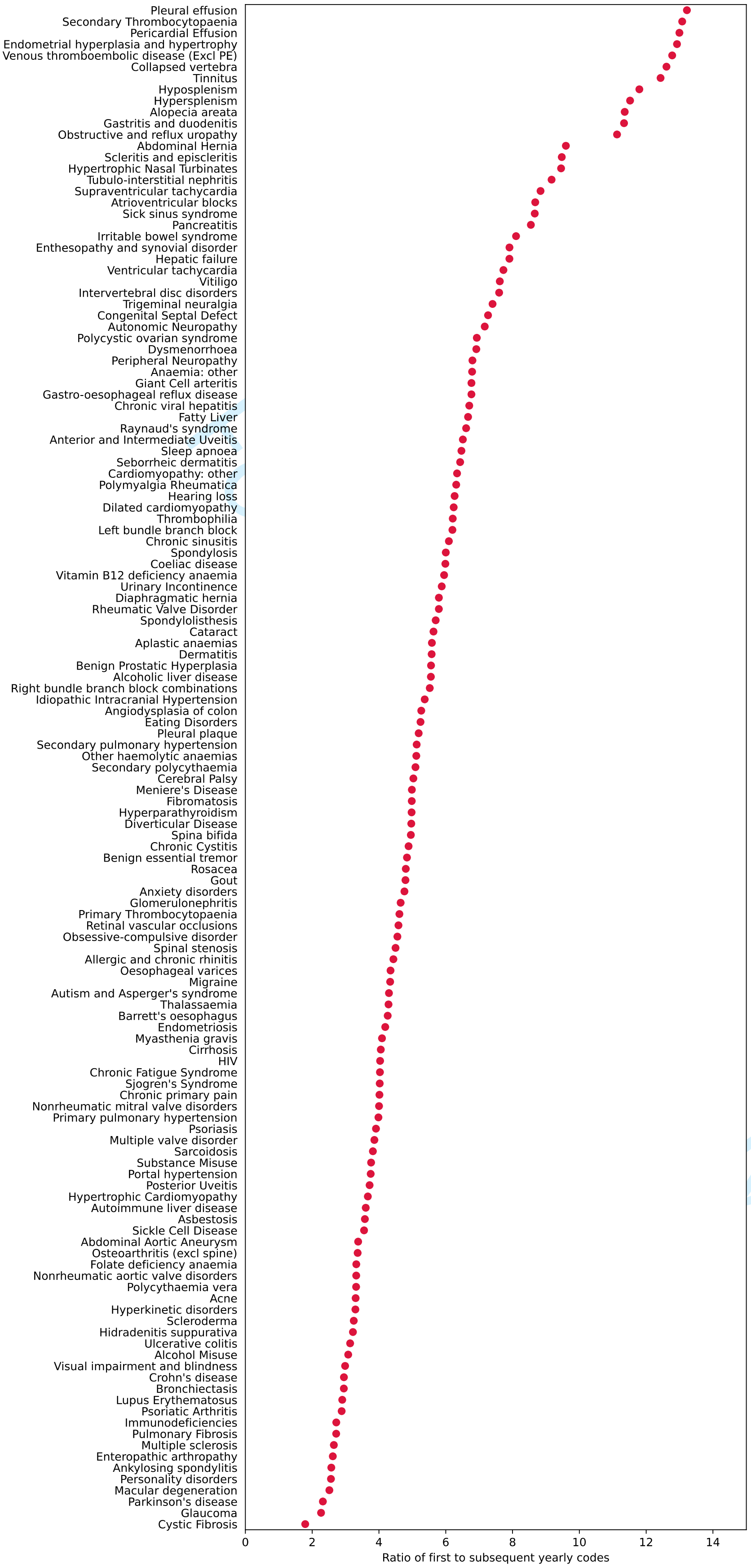

Appendix Figure A4: Boxplots of the distribution of mean yearly codes following diagnosis for newly diagnosed conditions by GP practice stratified by inclusion in QOF

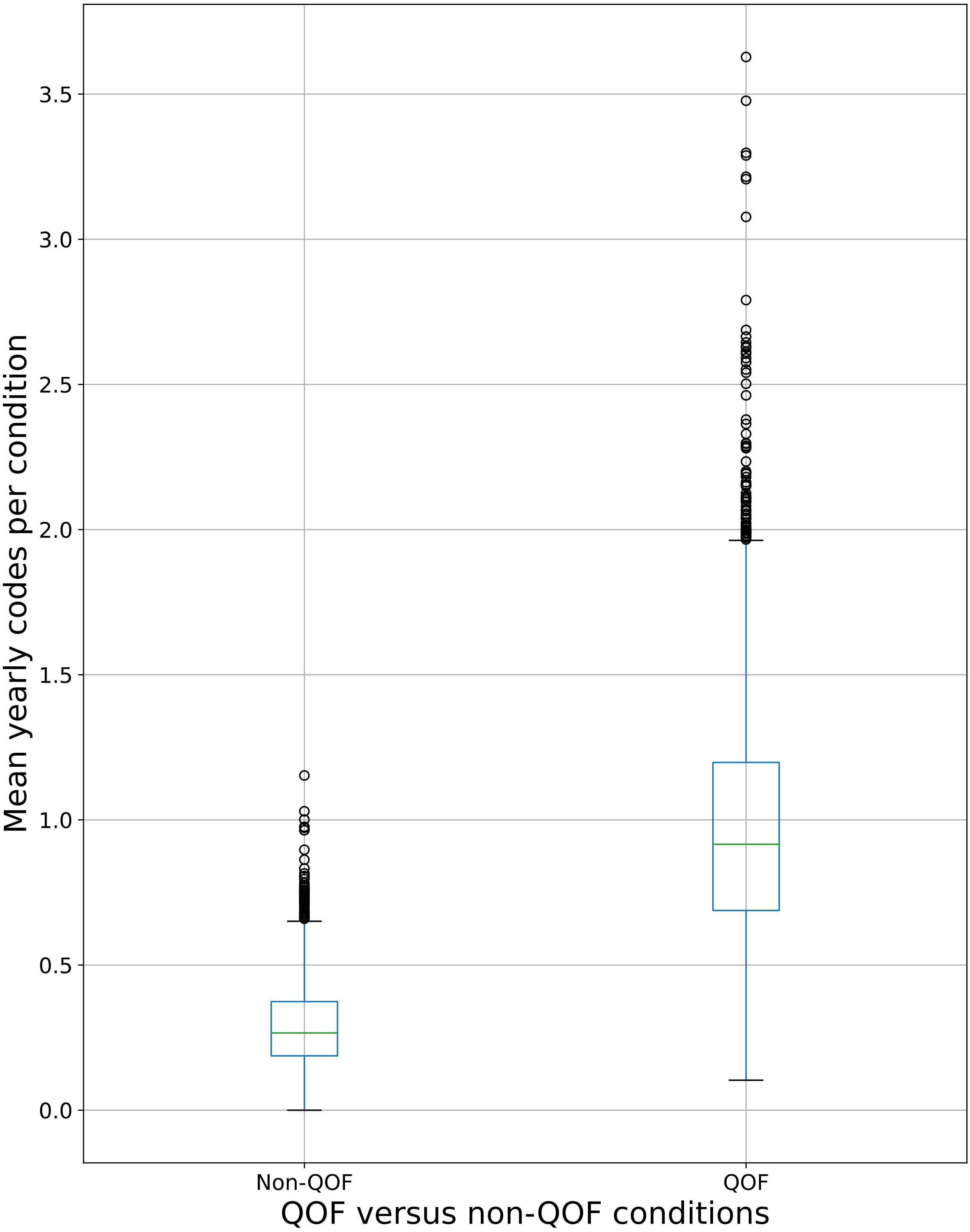

Appendix Figure A5: boxplots of mean yearly codes at a GP practice level by practice level Index of Multiple Deprivation decile (1 = most deprived, 10 = least deprived)

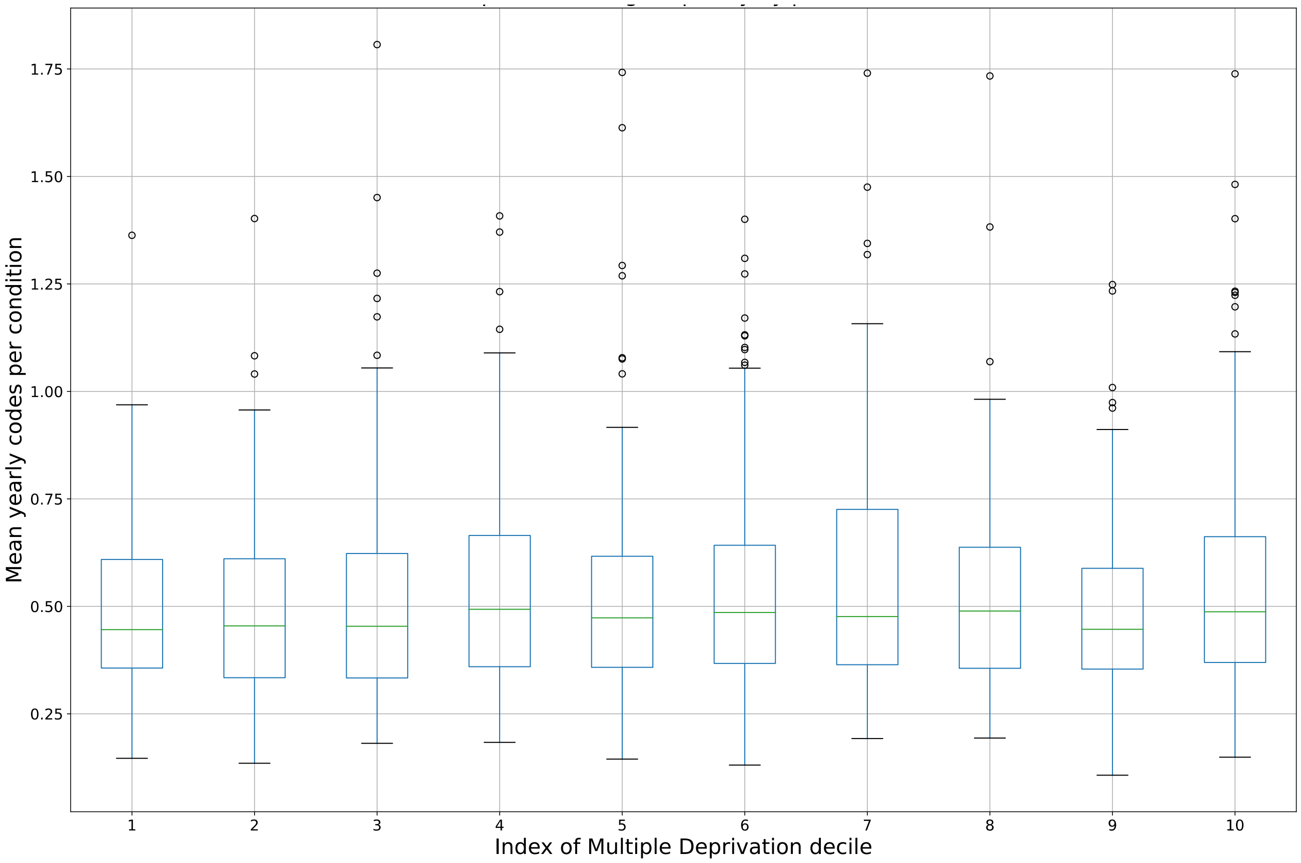

Footnote: combines QOF and non-QOF conditions

For peer review only - http://bmjopen.bmj.com/site/about/guidelines.xhtml

Table A3: Associations of rate of codes in year one following diagnosis for conditions included in QOF (N=1730485)

	Primary analysis				Sensitivity analysis including consultation number			
			95% CI				95% CI	
**Variable**	**IRR**	**P-value**	**Lower**	**Upper**	**IRR**	**P-value**	**Lower**	**Upper**
**Age category (years)**								
Under 40	1.33	0.00	1.32	1.34	1.30	0.00	1.29	1.31
40-49	1.15	0.00	1.14	1.15	1.14	0.00	1.13	1.15
50-59	1.08	0.00	1.07	1.08	1.07	0.00	1.07	1.08
60-69 (reference)	-	-	-	-	-	-	-	-
70-79	0.96	0.00	0.95	0.96	0.94	0.00	0.93	0.95
80 or more	0.91	0.00	0.90	0.92	0.88	0.00	0.87	0.88
**Sex**								
Female (reference)	-	-	-	-	-	-	-	-
Male	1.03	0.00	1.02	1.03	1.10	0.00	1.10	1.11
**Ethnicity category**								
White (reference)	-	-	-	-	-	-	-	-
South Asian	0.96	0.00	0.95	0.97	0.92	0.00	0.91	0.93
Black	0.94	0.00	0.93	0.95	0.94	0.00	0.93	0.95
Other	0.95	0.00	0.93	0.97	0.96	0.00	0.94	0.98
Mixed	0.98	0.03	0.95	1.00	0.97	0.00	0.95	0.99
Missing	0.98	0.00	0.97	0.99	1.01	0.00	1.00	1.02
**IMD decile**								
1 (most deprived)	-	-	-	-	-	-	-	-
2	1.01	0.19	1.00	1.01	1.00	0.95	0.99	1.01
3	1.02	0.00	1.01	1.03	1.01	0.08	1.00	1.02
4	1.02	0.00	1.01	1.03	1.01	0.01	1.00	1.02
5	1.02	0.00	1.01	1.03	1.01	0.06	1.00	1.02
6	1.03	0.00	1.02	1.04	1.01	0.02	1.00	1.02
7	1.04	0.00	1.03	1.05	1.02	0.00	1.01	1.03
8	1.04	0.00	1.03	1.05	1.01	0.01	1.00	1.02
9	1.05	0.00	1.04	1.06	1.02	0.00	1.01	1.03
10 (least deprived)	1.05	0.00	1.04	1.06	1.01	0.06	1.00	1.02
**Number of QOF diseases**								
0 (reference)	-	-	-	-	-	-	-	-
1	0.90	0.00	0.90	0.91	0.87	0.00	0.86	0.87
2	0.80	0.00	0.80	0.81	0.75	0.00	0.75	0.76
3	0.71	0.00	0.70	0.71	0.66	0.00	0.65	0.66
4 or more	0.63	0.00	0.62	0.63	0.56	0.00	0.55	0.56
**Number of non-QOF diseases**								
0 (reference)	-	-	-	-	-	-	-	-
1	1.16	0.00	1.16	1.17	1.08	0.00	1.07	1.08
2	1.13	0.00	1.12	1.14	1.02	0.00	1.01	1.02
3	1.12	0.00	1.11	1.12	0.97	0.00	0.96	0.98
4 or more	1.13	0.00	1.12	1.13	0.90	0.00	0.89	0.90
**Calendar year of diagnosis**								
2015 (reference)	-	-	-	-	-	-	-	-
2016	1.00	0.89	0.99	1.01	1.02	0.00	1.02	1.03
2017	1.00	0.34	1.00	1.01	1.05	0.00	1.04	1.05
2018	1.00	0.18	0.99	1.00	1.06	0.00	1.06	1.07
2019	0.95	0.00	0.94	0.96	1.04	0.00	1.04	1.05
**Average number of consultations in year 1**								
Less than 1 (reference)	-	-	-	-	-	-	-	-
1-2	-	-	-	-	1.62	0.00	1.60	1.63
3-4	-	-	-	-	2.21	0.00	2.19	2.23
5-9	-	-	-	-	2.87	0.00	2.84	2.89
10 or more	-	-	-	-	3.75	0.00	3.71	3.79

For peer review only

From negative binomial regression models, including practice-level fixed effects (not shown)

Table A4: Associations of rate of codes in year two following diagnosis for conditions included in QOF (N=1714684)

	Primary analysis				Sensitivity analysis including consultation number			
			95% CI				95% CI	
**Variable**	**IRR**	**P-value**	**Lower**	**Upper**	**IRR**	**P-value**	**Lower**	**Upper**
**Age category (years)**								
Under 40	0.87	0.00	0.86	0.87	0.86	0.00	0.86	0.87
40-49	1.01	0.03	1.00	1.02	1.01	0.22	1.00	1.01
50-59	1.03	0.00	1.02	1.04	1.03	0.00	1.02	1.04
60-69 (reference)	-	-	-	-	-	-	-	-
70-79	0.95	0.00	0.94	0.96	0.93	0.00	0.93	0.94
80 or more	0.89	0.00	0.88	0.90	0.86	0.00	0.85	0.87
**Sex**								
Female (reference)	-	-	-	-	-	-	-	-
Male	1.11	0.00	1.11	1.12	1.18	0.00	1.17	1.18
**Ethnicity category**								
White (reference)	-	-	-	-	-	-	-	-
South Asian	1.26	0.00	1.25	1.28	1.22	0.00	1.20	1.23
Black	1.17	0.00	1.16	1.19	1.17	0.00	1.15	1.19
Other	1.13	0.00	1.10	1.16	1.14	0.00	1.11	1.17
Mixed	1.12	0.00	1.08	1.15	1.11	0.00	1.07	1.14
Missing	0.89	0.00	0.88	0.90	0.93	0.00	0.92	0.93
**IMD decile**								
1 (most deprived)	-	-	-	-	-	-	-	-
2	1.02	0.00	1.01	1.04	1.02	0.00	1.01	1.03
3	1.03	0.00	1.02	1.05	1.03	0.00	1.02	1.04
4	1.05	0.00	1.03	1.06	1.04	0.00	1.03	1.05
5	1.05	0.00	1.04	1.07	1.04	0.00	1.03	1.06
6	1.06	0.00	1.05	1.07	1.05	0.00	1.04	1.07
7	1.08	0.00	1.06	1.09	1.06	0.00	1.05	1.08
8	1.09	0.00	1.07	1.10	1.07	0.00	1.06	1.08
9	1.11	0.00	1.10	1.13	1.09	0.00	1.08	1.11
10 (least deprived)	1.14	0.00	1.12	1.15	1.11	0.00	1.09	1.12
**Number of QOF diseases**								
0 (reference)	-	-	-	-	-	-	-	-
1	1.05	0.00	1.04	1.06	1.00	0.79	0.99	1.01
2	1.07	0.00	1.06	1.08	0.99	0.05	0.98	1.00
3	0.99	0.15	0.98	1.00	0.91	0.00	0.90	0.92
4 or more	0.87	0.00	0.86	0.88	0.77	0.00	0.76	0.78
**Number of non-QOF diseases**								
0 (reference)	-	-	-	-	-	-	-	-
1	1.05	0.00	1.04	1.06	0.99	0.11	0.98	1.00
2	1.04	0.00	1.03	1.05	0.96	0.00	0.95	0.97
3	1.04	0.00	1.03	1.05	0.93	0.00	0.92	0.94
4 or more	1.05	0.00	1.04	1.06	0.88	0.00	0.87	0.89
**Calendar year of diagnosis**								
2015 (reference)	-	-	-	-	-	-	-	-
2016	1.00	0.45	0.99	1.01	1.02	0.00	1.01	1.03
2017	0.99	0.00	0.98	0.99	1.02	0.00	1.01	1.03
2018	0.91	0.00	0.90	0.92	0.96	0.00	0.95	0.97
2019	0.79	0.00	0.79	0.80	0.86	0.00	0.86	0.87
**Average number of consultations in year 1**								
Less than 1 (reference)					-	-	-	-
1-2					1.53	0.00	1.52	1.55
3-4					1.87	0.00	1.85	1.89

For peer review only

5-9					2.17	0.00	2.15	2.20
10 or more					2.59	0.00	2.57	2.62

From negative binomial regression models, including practice-level fixed effects (not shown)

Table A5: Associations of rate of codes in year one following diagnosis for conditions not included in QOF (N=3617348)

	**Primary analysis**				**Sensitivity analysis including consultation number**			
			**95% CI**				**95% CI**	
**Variable**	**IRR**	**P-value**	**Lower**	**Upper**	**IRR**	**P-value**	**Lower**	**Upper**
**Age category (years)**								
Under 40	1.10	0.00	1.10	1.11	1.09	0.00	1.08	1.10
40-49	1.01	0.00	1.00	1.02	1.02	0.00	1.01	1.03
50-59	0.98	0.00	0.98	0.99	0.99	0.09	0.99	1.00
60-69 (reference)	-	-	-	-	-	-	-	-
70-79	1.05	0.00	1.05	1.06	1.03	0.00	1.02	1.03
80 or more	1.02	0.00	1.02	1.03	0.98	0.00	0.97	0.99
**Sex**								
Female (reference)	-	-	-	-	-	-	-	-
Male	1.00	0.03	0.99	1.00	1.13	0.00	1.12	1.13
**Ethnicity category**								
White (reference)	-	-	-	-	-	-	-	-
South Asian	0.95	0.00	0.94	0.96	0.89	0.00	0.88	0.90
Black	0.89	0.00	0.88	0.90	0.86	0.00	0.85	0.87
Other	0.90	0.00	0.88	0.91	0.89	0.00	0.88	0.91
Mixed	0.95	0.00	0.93	0.97	0.92	0.00	0.91	0.94
Missing	0.99	0.14	0.99	1.00	1.06	0.00	1.05	1.06
**IMD decile**								
1 (most deprived)	-	-	-	-	-	-	-	-
2	1.00	0.86	0.99	1.01	0.99	0.06	0.98	1.00
3	1.01	0.01	1.00	1.02	1.00	0.82	0.99	1.01
4	1.02	0.00	1.01	1.03	1.00	0.42	0.99	1.01
5	1.02	0.00	1.01	1.03	1.00	0.86	0.99	1.01
6	1.03	0.00	1.02	1.04	0.99	0.26	0.99	1.00
7	1.03	0.00	1.02	1.04	0.99	0.08	0.98	1.00
8	1.04	0.00	1.03	1.06	0.99	0.15	0.98	1.00
9	1.06	0.00	1.05	1.07	0.99	0.19	0.98	1.00
10 (least deprived)	1.06	0.00	1.05	1.07	0.98	0.00	0.97	0.99
**Number of QOF diseases**								
0 (reference)	-	-	-	-	-	-	-	-
1	1.16	0.00	1.15	1.16	1.02	0.00	1.02	1.03
2	1.09	0.00	1.08	1.09	0.94	0.00	0.93	0.94
3	1.06	0.00	1.05	1.07	0.90	0.00	0.89	0.91
4 or more	1.04	0.00	1.03	1.04	0.85	0.00	0.84	0.85
**Number of non-QOF diseases**								
0 (reference)	-	-	-	-	-	-	-	-
1	1.02	0.00	1.01	1.02	0.93	0.00	0.92	0.94
2	1.02	0.00	1.02	1.03	0.87	0.00	0.87	0.88
3	1.04	0.00	1.03	1.05	0.83	0.00	0.82	0.84
4 or more	1.06	0.00	1.06	1.07	0.74	0.00	0.74	0.75
**Calendar year of diagnosis**								
2015 (reference)	-	-	-	-	-	-	-	-
2016	1.00	0.55	0.99	1.00	1.03	0.00	1.02	1.03
2017	0.99	0.00	0.99	1.00	1.05	0.00	1.04	1.05
2018	0.99	0.00	0.98	0.99	1.07	0.00	1.06	1.07
2019	0.94	0.00	0.94	0.95	1.06	0.00	1.06	1.07
**Average number of consultations in year 1**								
Less than 1 (reference)					-	-	-	-

For peer review only

Variable	IRR	P-value	Lower	Upper	IRR	P-value	Lower	Upper
1-2					2.38	0.00	2.36	2.40
3-4					3.49	0.00	3.45	3.52
5-9					4.67	0.00	4.62	4.71
10 or more					6.37	0.00	6.31	6.44

From negative binomial regression models, including practice-level fixed effects (not shown)

Table A6: Associations of rate of codes in year two following diagnosis for conditions not included in QOF (N=3593019)

	Primary analysis				Sensitivity analysis including consultation number			
			95% CI				95% CI	
**Variable**	**IRR**	**P-value**	**Lower**	**Upper**	**IRR**	**P-value**	**Lower**	**Upper**
**Age category (years)**								
Under 40	1.27	0.00	1.26	1.28	1.26	0.00	1.25	1.28
40-49	1.03	0.00	1.02	1.04	1.03	0.00	1.02	1.04
50-59	0.98	0.00	0.97	0.99	0.99	0.10	0.98	1.00
60-69 (reference)	-	-	-	-	-	-	-	-
70-79	1.06	0.00	1.05	1.07	1.03	0.00	1.02	1.04
80 or more	1.06	0.00	1.05	1.08	1.01	0.18	1.00	1.02
**Sex**								
Female (reference)	-	-	-	-	-	-	-	-
Male	0.93	0.00	0.93	0.94	1.08	0.00	1.07	1.09
**Ethnicity category**								
White (reference)	-	-	-	-	-	-	-	-
South Asian	0.99	0.17	0.97	1.00	0.92	0.00	0.91	0.94
Black	0.94	0.00	0.92	0.95	0.91	0.00	0.89	0.92
Other	0.88	0.00	0.86	0.91	0.89	0.00	0.86	0.92
Mixed	0.94	0.00	0.91	0.98	0.92	0.00	0.89	0.95
Missing	0.96	0.00	0.95	0.97	1.05	0.00	1.03	1.06
**IMD decile**								
1 (most deprived)	-	-	-	-	-	-	-	-
2	1.01	0.10	1.00	1.03	1.00	0.79	0.99	1.02
3	1.03	0.00	1.02	1.05	1.02	0.00	1.01	1.04
4	1.04	0.00	1.02	1.05	1.02	0.01	1.01	1.04
5	1.05	0.00	1.04	1.07	1.03	0.00	1.01	1.04
6	1.06	0.00	1.04	1.08	1.03	0.00	1.01	1.04
7	1.07	0.00	1.06	1.09	1.03	0.00	1.01	1.05
8	1.10	0.00	1.08	1.11	1.04	0.00	1.03	1.06
9	1.13	0.00	1.11	1.14	1.06	0.00	1.04	1.08
10 (least deprived)	1.14	0.00	1.12	1.16	1.06	0.00	1.04	1.08
**Number of QOF diseases**								
0 (reference)	-	-	-	-	-	-	-	-
1	1.19	0.00	1.18	1.21	1.05	0.00	1.04	1.06
2	1.15	0.00	1.14	1.16	0.98	0.00	0.97	0.99
3	1.13	0.00	1.12	1.15	0.95	0.00	0.94	0.96
4 or more	1.16	0.00	1.14	1.17	0.93	0.00	0.92	0.94
**Number of non-QOF diseases**								
0 (reference)	-	-	-	-	-	-	-	-
1	1.04	0.00	1.03	1.06	0.94	0.00	0.93	0.95
2	1.09	0.00	1.08	1.11	0.90	0.00	0.89	0.91
3	1.13	0.00	1.11	1.14	0.86	0.00	0.85	0.87
4 or more	1.21	0.00	1.20	1.23	0.80	0.00	0.79	0.81
**Calendar year of diagnosis**								
2015 (reference)	-	-	-	-	-	-	-	-
2016	1.00	0.56	0.99	1.01	1.03	0.00	1.02	1.04
2017	1.00	0.43	0.99	1.01	1.06	0.00	1.05	1.07
2018	0.91	0.00	0.90	0.92	1.01	0.01	1.00	1.02
2019	0.79	0.00	0.79	0.80	0.93	0.00	0.92	0.94

For peer review only

For peer review only - http://bmjopen.bmj.com/site/about/guidelines.xhtml

**Average number of consultations in year 1**					
Less than 1 (reference)		-	-	-	-
1-2		2.76	0.00	2.72	2.81
3-4		4.06	0.00	4.00	4.12
5-9		5.40	0.00	5.32	5.48
10 or more		7.35	0.00	7.24	7.47

From negative binomial regression models, including practice-level fixed effects (not shown)

For peer review only

References

1. Head, A. *et al.* Inequalities in incident and prevalent multimorbidity in England, 2004–19: a population-based, descriptive study. *The Lancet Healthy Longevity* **2**, e489–e497 (2021).
2. Kuan, V. *et al.* A chronological map of 308 physical and mental health conditions from 4 million individuals in the English National Health Service. *The Lancet Digital Health* **1**, e63–e77 (2019).
3. Bisquera, A. *et al.* Inequalities in developing multimorbidity over time: A population-based cohort study from an urban, multi-ethnic borough in the United Kingdom. *Lancet Reg Health Eur* **12**, 100247 (2021).
4. Ashworth, M. *et al.* Journey to multimorbidity: longitudinal analysis exploring cardiovascular risk factors and sociodemographic determinants in an urban setting. *BMJ Open* **9**, (2019).
5. NHS Health and Social Care Information Centre. National Quality and Outcomes Framework Statistics for England 2004/05. https://files.digital.nhs.uk/publicationimport/pub01xxx/pub01946/qof-eng-04-05-rep.pdf.
6. Health & Social Care Information Centre. Quality and Outcomes Framework – Prevalence, Achievements and Exceptions Report. England, 2013-14. https://files.digital.nhs.uk/publicationimport/pub15xxx/pub15751/qof-1314-report-v1.1.pdf.
7. Health & Social Care Information Centre. Quality and Outcomes Framework – Prevalence, Achievements and Exceptions Report, England, 2014-15. https://files.digital.nhs.uk/publicationimport/pub18xxx/pub18887/qof-1415-report%20v1.1.pdf (2015).

For peer review only - http://bmjopen.bmj.com/site/about/guidelines.xhtml

The RECORD statement – checklist of items, extended from the STROBE statement, that should be reported in observational studies using routinely collected health data.

	Item No.	STROBE items	Location in manuscript where items are reported	RECORD items	Location in manuscript where items are reported
**Title and abstract**					
	1	(a) Indicate the study's design with a commonly used term in the title or the abstract (b) Provide in the abstract an informative and balanced summary of what was done and what was found	p1-3	RECORD 1.1: The type of data used should be specified in the title or abstract. When possible, the name of the databases used should be included.<br><br>RECORD 1.2: If applicable, the geographic region and timeframe within which the study took place should be reported in the title or abstract.<br><br>RECORD 1.3: If linkage between databases was conducted for the study, this should be clearly stated in the title or abstract.	p1<br><br>p2<br><br>N/A
**Introduction**					
Background rationale	2	Explain the scientific background and rationale for the investigation being reported	p4-5		
Objectives	3	State specific objectives, including any prespecified hypotheses	p5		
**Methods**					
Study Design	4	Present key elements of study design early in the paper	p5		
Setting	5	Describe the setting, locations, and relevant dates, including periods of recruitment, exposure, follow-up, and data collection	p5		

For peer review only

Participants	6	*(a) Cohort study* - Give the eligibility criteria, and the sources and methods of selection of participants. Describe methods of follow-up<br>*Case-control study* - Give the eligibility criteria, and the sources and methods of case ascertainment and control selection. Give the rationale for the choice of cases and controls<br>*Cross-sectional study* - Give the eligibility criteria, and the sources and methods of selection of participants<br><br>*(b) Cohort study* - For matched studies, give matching criteria and number of exposed and unexposed<br>*Case-control study* - For matched studies, give matching criteria and the number of controls per case	p5 and appendix p2	RECORD 6.1: The methods of study population selection (such as codes or algorithms used to identify subjects) should be listed in detail. If this is not possible, an explanation should be provided.<br><br>RECORD 6.2: Any validation studies of the codes or algorithms used to select the population should be referenced. If validation was conducted for this study and not published elsewhere, detailed methods and results should be provided.<br><br>RECORD 6.3: If the study involved linkage of databases, consider use of a flow diagram or other graphical display to demonstrate the data linkage process, including the number of individuals with linked data at each stage.	p5
Variables	7	Clearly define all outcomes, exposures, predictors, potential confounders, and effect modifiers. Give diagnostic criteria, if applicable.	p5 and appendix p2-3	RECORD 7.1: A complete list of codes and algorithms used to classify exposures, outcomes, confounders, and effect modifiers should be provided. If these cannot be reported, an explanation should be provided.	
Data sources/ measurement	8	For each variable of interest, give sources of data and details of methods of assessment (measurement).<br>Describe comparability of assessment methods if there is more than one group	p5 and appendix p2-3		

For peer review only

Bias	9	Describe any efforts to address potential sources of bias	p6-7		
Study size	10	Explain how the study size was arrived at	p8		
Quantitative variables	11	Explain how quantitative variables were handled in the analyses. If applicable, describe which groupings were chosen, and why	P5-6		
Statistical methods	12	(a) Describe all statistical methods, including those used to control for confounding<br>(b) Describe any methods used to examine subgroups and interactions<br>(c) Explain how missing data were addressed<br>(d) *Cohort study* - If applicable, explain how loss to follow-up was addressed<br>*Case-control study* - If applicable, explain how matching of cases and controls was addressed<br>*Cross-sectional study* - If applicable, describe analytical methods taking account of sampling strategy<br>(e) Describe any sensitivity analyses	p6-7		
Data access and cleaning methods		..		RECORD 12.1: Authors should describe the extent to which the investigators had access to the database population used to create the study population.	p5

For peer review only

				RECORD 12.2: Authors should provide information on the data cleaning methods used in the study.	
Linkage		..		RECORD 12.3: State whether the study included person-level, institutional-level, or other data linkage across two or more databases. The methods of linkage and methods of linkage quality evaluation should be provided.	N/A
**Results**					
Participants	13	(a) Report the numbers of individuals at each stage of the study (*e.g.*, numbers potentially eligible, examined for eligibility, confirmed eligible, included in the study, completing follow-up, and analysed)<br>(b) Give reasons for non-participation at each stage.<br>(c) Consider use of a flow diagram	p8	RECORD 13.1: Describe in detail the selection of the persons included in the study (*i.e.,* study population selection) including filtering based on data quality, data availability and linkage. The selection of included persons can be described in the text and/or by means of the study flow diagram.	p8
Descriptive data	14	(a) Give characteristics of study participants (*e.g.*, demographic, clinical, social) and information on exposures and potential confounders<br>(b) Indicate the number of participants with missing data for each variable of interest<br>(c) *Cohort study* - summarise follow-up time (*e.g.*, average and total amount)	p8, Table 1		
Outcome data	15	*Cohort study* - Report numbers of outcome events or summary measures over time<br>*Case-control study* - Report numbers in each exposure	p9-10		

For peer review only

		category, or summary measures of exposure<br>*Cross-sectional study* - Report numbers of outcome events or summary measures			
Main results	16	(a) Give unadjusted estimates and, if applicable, confounder-adjusted estimates and their precision (e.g., 95% confidence interval). Make clear which confounders were adjusted for and why they were included<br>(b) Report category boundaries when continuous variables were categorized<br>(c) If relevant, consider translating estimates of relative risk into absolute risk for a meaningful time period	p9-14, Figures 1-3		
Other analyses	17	Report other analyses done—e.g., analyses of subgroups and interactions, and sensitivity analyses	p11		
**Discussion**					
Key results	18	Summarise key results with reference to study objectives	p15		
Limitations	19	Discuss limitations of the study, taking into account sources of potential bias or imprecision. Discuss both direction and magnitude of any potential bias	p17	RECORD 19.1: Discuss the implications of using data that were not created or collected to answer the specific research question(s). Include discussion of misclassification bias, unmeasured confounding, missing data, and changing eligibility over time, as they pertain to the study being reported.	p17
Interpretation	20	Give a cautious overall interpretation of results considering objectives,	p15, p17-18		

For peer review only

		limitations, multiplicity of analyses, results from similar studies, and other relevant evidence			
Generalisability	21	Discuss the generalisability (external validity) of the study results	p17		
**Other Information**					
Funding	22	Give the source of funding and the role of the funders for the present study and, if applicable, for the original study on which the present article is based	p18		
Accessibility of protocol, raw data, and programming code		..		RECORD 22.1: Authors should provide information on how to access any supplemental information such as the study protocol, raw data, or programming code.	p18

*Reference: Benchimol EI, Smeeth L, Guttmann A, Harron K, Moher D, Petersen I, Sørensen HT, von Elm E, Langan SM, the RECORD Working Committee. The REporting of studies Conducted using Observational Routinely-collected health Data (RECORD) Statement. *PLoS Medicine* 2015; in press.

*Checklist is protected under Creative Commons Attribution (CC BY) license.

For peer review only

Identifying potential biases in code sequences in primary care electronic healthcare records: a retrospective cohort study of the determinants of code frequency

Journal:	*BMJ Open*
Manuscript ID	bmjopen-2023-072884.R1
Article Type:	Original research
Date Submitted by the Author:	03-Jul-2023
Complete List of Authors:	Beaney, Thomas; Imperial College London, Department of Primary Care and Public Health; Imperial College London, Department of Mathematics<br>Clarke, Jonathan; Imperial College of Science Technology and Medicine, Institute of Global Health Innovation<br>Salman, David; Imperial College London Department of Primary Care and Public Health; Imperial College London Faculty of Medicine, MSk lab<br>Woodcock, Thomas; Imperial College London, Department of Primary Care and Public Health<br>Majeed, Azeem; Imperial College London, Department of Primary Care and Public Health<br>Barahona, Mauricio; Imperial College London, Centre for Mathematics of Precision Healthcare; Imperial College London, Department of Mathematics<br>Aylin, Paul; Imperial College London, Department of Primary Care and Public Health
**Primary Subject Heading**:	Health informatics
Secondary Subject Heading:	General practice / Family practice, Health informatics, Health services research, Epidemiology
Keywords:	EPIDEMIOLOGY, Primary Health Care, Health informatics < BIOTECHNOLOGY & BIOINFORMATICS, STATISTICS & RESEARCH METHODS

SCHOLARONE™ Manuscripts

I, the Submitting Author has the right to grant and does grant on behalf of all authors of the Work (as defined in the below author licence), an exclusive licence and/or a non-exclusive licence for contributions from authors who are: i) UK Crown employees; ii) where BMJ has agreed a CC-BY licence shall apply, and/or iii) in accordance with the terms applicable for US Federal Government officers or employees acting as part of their official duties; on a worldwide, perpetual, irrevocable, royalty-free basis to BMJ Publishing Group Ltd ("BMJ") its licensees and where the relevant Journal is co-owned by BMJ to the co-owners of the Journal, to publish the Work in this journal and any other BMJ products and to exploit all rights, as set out in our licence.

The Submitting Author accepts and understands that any supply made under these terms is made by BMJ to the Submitting Author unless you are acting as an employee on behalf of your employer or a postgraduate student of an affiliated institution which is paying any applicable article publishing charge ("APC") for Open Access articles. Where the Submitting Author wishes to make the Work available on an Open Access basis (and intends to pay the relevant APC), the terms of reuse of such Open Access shall be governed by a Creative Commons licence – details of these licences and which Creative Commons licence will apply to this Work are set out in our licence referred to above.

Other than as permitted in any relevant BMJ Author's Self Archiving Policies, I confirm this Work has not been accepted for publication elsewhere, is not being considered for publication elsewhere and does not duplicate material already published. I confirm all authors consent to publication of this Work and authorise the granting of this licence.

For peer review only

Identifying potential biases in code sequences in primary care electronic healthcare records: a retrospective cohort study of the determinants of code frequency

Beaney T[1,2] (0000-0001-9709-7264), Clarke J[2], Salman D[1,3], Woodcock T[1], Majeed A[1], Barahona M[2], Aylin P[1]

1. Department of Primary Care and Public Health, Imperial College London, London, W6 8RP, United Kingdom
2. Centre for Mathematics of Precision Healthcare, Department of Mathematics, Imperial College London, London, SW7 2AZ, United Kingdom
3. MSk Lab, Department of Surgery and Cancer, Faculty of Medicine, Imperial College London, London, UK

Corresponding Author:
Dr Thomas Beaney
Department of Primary Care and Public Health, Imperial College London, London, W6 8RP, United Kingdom
Email: thomas.beaney@imperial.ac.uk

For peer review only

Abstract

Objectives

To determine whether the frequency of diagnostic codes for long-term conditions (LTCs) in primary care electronic health records (EHRs) is associated with i) disease coding incentives, ii) GP practice, iii) patient socio-demographic characteristics and iv) calendar year of diagnosis.

Design

Retrospective cohort study.

Setting

General practices in England from 2015 to 2022 contributing to the Clinical Practice Research Datalink Aurum dataset.

Participants

All patients registered to a GP with at least one incident LTC diagnosed between 01/01/2015 and 31/12/2019.

Primary and secondary outcome measures

The number of diagnostic codes for an LTC in i) the first and ii) the second year following diagnosis, stratified by inclusion in the Quality and Outcomes Framework (QOF) financial incentive programme.

Results

3,113,724 patients were included, with 7,723,365 incident LTCs. Conditions included in QOF had higher rates of annual coding than conditions not included in QOF (1.03 vs 0.32 per year, $p<0.0001$). There was significant variation in code frequency by GP practice which was not explained by patient socio-demographics. We found significant associations with patient socio-demographics, with a trend towards lower coding rates in people living in areas of higher deprivation for both QOF and non-QOF conditions. Code frequency was lower for conditions with follow-up time in 2020, associated with the onset of the COVID-19 pandemic.

Conclusions

The frequency of diagnostic codes for newly diagnosed LTCs is influenced by factors including patient socio-demographics, disease inclusion in QOF, GP practice, and the impact of the COVID-19 pandemic. Natural language processing or other methods using temporally-ordered code sequences should account for these factors to minimise potential bias.

For peer review only

Strengths and limitations

- This study used a large and representative sample of patients in England, including 3 million patients with one of 208 incident diseases developed over 5 years.
- We focussed on incident diseases during the study period to minimise bias from historic or inactive diseases.
- We found significant differences in the frequency of codes according to patient socio-demographics, GP practice, and disease inclusion in QOF, but could not determine whether these differences reflect differences in healthcare utilisation versus coding quality.

For peer review only

Background

Methods developed in natural language processing (NLP) are increasingly being employed to analyse routinely collected healthcare data, such as data recorded in the Electronic Healthcare Record (EHR).[1–6] These methods show promise across a range of tasks, including prediction of health outcomes,[1,5,6] and clustering of co-occurring diseases.[2] Although developed for the analysis of language data, such as the free text data found in 'unstructured' medical records, NLP methods can also be applied to coded or 'structured' data found in many EHR databases. Using structured data, disease codes arranged in a temporal sequence in a patient's EHR history can be considered analogous to words in a sentence or document.[5]

In primary care EHRs, diagnostic codes may be entered either during a consultation, or entered outside, such as on receiving communication of a new diagnosis from hospital, or retrospectively coding a pre-existing diagnosis. In predictive modelling scenarios, such as those used in NLP, codes from both sources are relevant to understanding a patient's health status. However, a potential problem facing sequence-based methods is the extent to which repeated codes are an objective marker of a patient's health status and a presentation with a particular condition or relate to the quality of coding in the EHR.[7] Although previous studies of EHR data in England have shown the prevalence of many long-term conditions (LTCs) to be comparable to those from national statistics, these are often calculated based on the presence of a single diagnostic code.[8] Whether repeated codes for LTCs are entered in the EHR subsequently may be determined by a range of factors, including patient characteristics, clinician incentives and organisational policies, which may vary over time.[9,10]

Unlike in secondary care, where diagnostic coding directly impacts on payments, General Practice in England receives funding primarily through capitated payments based on the size of the registered population[11] with no direct financial incentive for code entry during a consultation. However, around 10% of funding comes from the Quality and Outcomes Framework (QOF), introduced in the National Health Service for GPs in 2004.[11] QOF provides financial incentives for meeting targets for a set of chronic conditions, including regular clinical reviews, and has been credited with improvements to data collection for these conditions.[12–14] Codes for conditions in QOF may occur more frequently than for conditions not included in the incentive scheme, which could affect sequence-based methods using recurrent codes.

For peer review only

Analytical methods using temporally-ordered code sequences in the EHR may therefore be susceptible to biases in the frequency of codes entered following diagnosis, potentially resulting in models representing some people better than others. Awareness of the factors influencing the frequency of codes may help researchers using NLP methods by informing adjustment or sensitivity analyses. This study aims firstly to compare the frequency of repeated codes after diagnosis for a common set of LTCs. Secondly, we aim to determine whether the frequency of codes varies according to i) disease inclusion in QOF, ii) GP practice, iii) patient socio-demographic characteristics, and iv) calendar year of diagnosis.

Methods

Data source

This study used data from the Clinical Practice Research Datalink (CPRD) Aurum dataset, which contains primary care data for GP practices using EMIS Web software.[15] We included all patients assessed by CPRD to be research acceptable (meeting certain quality criteria such as a valid registration date and date of birth[16]) with a continuous period of registration at a GP practice in CPRD between 1st January 2014 and 31st December 2020 (i.e. without having deregistered in this period).[17] Patients were eligible if aged 18 years or over with at least one incident disease diagnosed between 1st January 2015 and 31st December 2019, allowing for at least one full year of practice registration before disease diagnosis and at least one full year of follow-up for each condition. Demographic data included age, sex, ethnicity and Index of Multiple Deprivation (IMD) of the area in which the patient resided, grouped into deciles where 1 is the most deprived and 10 the least deprived.[18] Ethnicity is recorded as one of five categories, with recording in CPRD found previously to have high concordance with national estimates.[19] We focussed on incident diseases to reduce the potential for confounding from historic conditions, some of which may no longer be active. Patients were followed up until the earliest of death, de-registration or the date of latest data extraction from their GP practice. Further information on the cohort structure is given in the appendix (p2).

Disease definitions

Diagnostic codes were extracted from the CPRD ‘Observation’ table and codes recorded during or outside of consultations were included. The date of the event (‘obsdate’) was used, in preference to the date the code was entered. We included a total of 208 LTCs. These were

For peer review only

defined based on a set of disease codes from Head *et al* (2021), who selected 211 chronic conditions from 308 acute and chronic disease phenotypes developed for the CALIBER study.[20,21] We reviewed codes and made changes to the code-lists for diabetes and added a new condition of 'chronic primary pain' (see appendix p2-3). We excluded conditions based only on laboratory results or anthropometric measurement codes as these may have different characteristics of coding frequency. As a result, measures of raised cholesterol used in the original CALIBER study were excluded. We also excluded BMI and eGFR measurements but included the diagnostic codes for obesity and Chronic Kidney Disease. We considered a single code as diagnostic for each condition and defined the diagnosis date for each condition as the date of the earliest code for that condition. Diseases were stratified according to whether they appeared in QOF by two primary care clinicians, TB and DS (see appendix p2-3).

Statistical analysis

Descriptive statistics

For each disease newly diagnosed during the study period, we calculated the yearly number of subsequent codes (excluding the first code representing diagnosis) during follow-up:

$$y_i = \frac{\sum_{j=1}^{N} c_{i,j}}{\sum_{j=1}^{N} f_{i,j}}$$

where y_i is the yearly number of codes following diagnosis for condition *i*, $c_{i,j}$ is the count of codes for condition *i* in patient *j*, and $f_{i,j}$ is the number of years of follow-up for condition *i* in patient *j*. T-tests were used to compare the mean yearly number of codes for QOF versus non-QOF conditions.

To examine variation in disease coding frequency by GP practice, we calculated, for each practice *k*, the mean number of codes per year for newly diagnosed diseases, p_k:

$$p_k = \frac{\sum_{j=1}^{N} \sum_{i=1}^{M} c_{i,j,k}}{\sum_{j=1}^{N} \sum_{i=1}^{M} f_{i,j,k}}$$

where $c_{i,j,k}$ is the count of codes for condition *i* in patient *j* in practice *k,* and $f_{i,j,k}$ is the number of years of follow-up for condition *i* in patient *j* in practice *k.* We then calculated the Pearson correlation coefficient between the mean number of codes per year in each practice

For peer review only

for QOF versus non-QOF conditions. We also compared the mean number of yearly codes in each practice stratified by the 2019 IMD decile of the GP practice. For conditions with at least two years of follow-up after the date of diagnosis, we calculated the ratio of the number of codes in the first year of diagnosis to the number of codes in subsequent years.

Regression analyses

Data were formatted as panel data with patients measured over multiple calendar years (appendix Table A1). We used mixed effects negative binomial regression to analyse the association between code frequency of newly diagnosed conditions in i) the first year following diagnosis and ii) the second year following diagnosis, with patient factors and calendar year of diagnosis. We separated the outcome variable (code frequency) into first and second year after diagnosis due to preliminary analyses indicating significant differences over time. We also stratified the regression analyses by QOF inclusion, given our hypothesis that it may be an effect modifier of the relationships. To account for cases where a patient may have more than one QOF or non-QOF condition diagnosed within the same year, we averaged the code frequency for all newly diagnosed QOF or non-QOF conditions in each calendar year.

Included as covariates in the model were patient socio-demographic factors including age, sex, ethnicity and IMD decile of residence. We also included the count of QOF and non-QOF conditions for each patient. Due to small numbers, we excluded patients with gender recorded in CPRD as 'indeterminate' or with missing IMD deciles. Age and the count of QOF and non-QOF conditions were time-updated at the start of each calendar year, and other covariates were held fixed. We incorporated random effects for patient and fixed effects for calendar year as we wished to explicitly model the effect of time. Use of a Poisson model was considered, but the conditional variance was found to be significantly higher than the conditional mean ($p<0.001$) indicating a negative binomial to have better fit.[22] Model fit was assessed by calculating randomized quantile residuals, which indicated no departure from normality on quantile-quantile plots.[23,24]

For each regression model, we calculated the predicted count of disease codes for each patient per year and then calculated the mean for each GP practice. This indicated that significant variation remained in the mean counts according to GP practice (appendix Figure A1). We therefore incorporated fixed effects for GP practice within the regression models to

For peer review only

account for practice-level variation (see appendix p5 for model equation). We also compared the Akaike Information Criteria (AIC) of models with and without practice fixed effects.

To assess whether code frequency was a function of overall number of primary care consultations, we conducted a sensitivity analysis including average number of yearly consultations (irrespective of condition) in year 1 or year 2 added as a covariate into the main regression models (categorised into <1, 1-2, 3-4, 5-9 or 10 or more). Python version 3.10.6 and Pandas version 1.4.3 were used in data processing and plots and Stata version 17.0 and R studio version 4.2.1 were used for regression analyses.

Patient and Public Involvement

This research programme is supported by a patient and public advisory group who fed back to the researchers on the diseases included in the study but were not directly involved in this study.

For peer review only

Results

A total of 6,174,115 patients aged 18 years or over and with a continuous registration period between 1st January 2014 and 31st December 2020 were eligible for inclusion in the study. Of these, 3,113,724 (50.4%) had at least one incident disease diagnosed between 1st January 2015 and 31st December 2019. Characteristics of the eligible population are shown in Table 1. 21.4% of patients were aged between 18-40 years as of the study start date, and 7.0% were aged 80 years or over. There were more women than men (54.1% versus 45.9%), most (76.7%) were of White ethnicity and there were relatively more patients in more deprived IMD deciles (51.7% in the most deprived half). Of patients with pre-existing conditions developed before the study start date, 31.6% had one or more QOF conditions, and 71.3% had one or more non-QOF conditions. Hypertension was the most prevalent pre-existing condition (24.1%), and the frequency of all pre-existing conditions are shown in the appendix Table A2. The 3,060,391 patients who were not eligible (as they did not develop an incident disease over the study period), were more likely to be younger and more likely to be male than those eligible (appendix Table A3).

For peer review only

Table 1: Socio-demographic characteristics of patients included in the study

Patient characteristic	Total	Percent
**Age (years)**		
18-39	665543	21.4%
40-49	562934	18.1%
50-59	604284	19.4%
60-69	585062	18.8%
70-79	476626	15.3%
80+	219275	7.0%
**Gender**		
Female	1684942	54.1%
Male	1428734	45.9%
Indeterminate	48	<0.1%
**Ethnicity**		
White	2388332	76.7%
South Asian	194477	6.2%
Black	103504	3.3%
Other	36430	1.2%
Mixed	27572	0.9%
Missing	363409	11.7%
**IMD decile**		
1 (most deprived)	358948	11.5%
2	320042	10.3%
3	320340	10.3%
4	323782	10.4%
5	287114	9.2%
6	303798	9.8%
7	304044	9.8%
8	298185	9.6%
9	305563	9.8%
10 (least deprived)	290214	9.3%
Missing	1694	0.1%
**Pre-existing QOF conditions***		
0	2130680	68.4%
1	393905	12.7%
2	224147	7.2%
3	142104	4.6%
4 or more	222888	7.2%
**Pre-existing non-QOF conditions***		
0	893765	28.7%
1	561300	18.0%
2	506053	16.3%
3	386912	12.4%
4 or more	765694	24.6%
**Total**	**3113724**	

* Pre-existing conditions defined as of study start date

For peer review only

Code frequency by disease and by time from diagnosis

A total of 7,723,365 diseases were diagnosed during the study period with follow-up times for each disease ranging from 1.0 to 7.2 years (mean 4.1 years). There was substantial variation in the yearly code frequency after diagnosis for each condition diagnosed during the study period. Diabetes (types 1, 2 and unspecified), polymyalgia rheumatica, motor neurone disease and dementia had the highest median number of codes per year (appendix Table A4). For many chronic diseases, yearly code frequency was low, for example, only 5% of patients with spina bifida had ≥0.5 codes per year. Conditions included in QOF on average had significantly higher mean number of yearly codes (1.03) than conditions not included in QOF (0.32; $p<0.0001$).

The number of codes was higher in the first year after diagnosis than in subsequent years for almost all conditions, except for secondary bowel or pleural malignancy and diabetic eye disease, for which code frequency was higher on average after the first year of diagnosis. QOF conditions on average had lower ratios of codes in the first compared to subsequent years than non-QOF conditions (4.8 versus 5.7 times higher in year 1). However, diseases representing major cardiovascular events, such as myocardial infarction, were coded much more frequently in the first year from diagnosis than in subsequent years (appendix Figure A2 and Figure A3).

Variation in coding frequency by GP practice

There was a wide range in the mean yearly number of codes per condition between GP practices, with higher code frequency for QOF compared to non-QOF conditions (appendix Figure A4). There was a strong correlation ($r = 0.88$) between GP practice mean code frequency for QOF and non-QOF conditions, indicating that those practices with high code frequency for QOF conditions also had high code frequency for non-QOF conditions (Figure 1). There was no observed trend according to the GP practice-level IMD decile (appendix Figure A5).

Figure 1: Scatterplot of mean yearly number of codes following diagnosis for QOF versus non-QOF conditions for each GP practice

For peer review only

We calculated the expected counts of codes for new diseases in year 1 and year 2 following diagnosis, predicted from negative binomial regression models. Expected mean counts per condition at GP practice level showed substantially less variation compared to the observed mean counts for both QOF and non-QOF conditions in year 1 and year 2 (appendix Figure A1) indicating substantial residual practice level variation independent of patient socio-demographic factors.

Variation in disease frequency by socio-demographics and over time

We found significant associations between code frequency in year 1 and year 2 following diagnosis with patient socio-demographic factors and calendar year of diagnosis for both QOF and non-QOF diseases from mixed effects negative binomial regression, after adjustment for number of pre-existing conditions (Figures 2 and 3, and appendix Tables A5 – A8). Inclusion of GP practice fixed effects in the regression models resulted in very similar coefficients for patient sociodemographic factors, and a significantly lower AIC indicating better model fit and so results are presented including practice-level effects.

Associations with QOF conditions

Younger patients tended to have a higher frequency of codes in the first year following diagnosis compared to older patients (Figure 1). However, in the second year from diagnosis, there was a U-shaped relationship with age, with the youngest and oldest age groups having the lowest rate of codes. Males had on average a small 3% increase (95% CI: 1.03 – 1.03) in the incidence rate of codes in year 1 and 11% (95% CI: 1.11 – 1.12) increase in year 2 compared with females. There was a strong relationship with ethnicity, with people of non-White ethnicities having lower rates of code frequency than people of White ethnicity in year 1, but higher rates in year 2. There was a strong trend towards higher code frequency in year 1 and year 2 with decreasing levels of deprivation.

Associations with non-QOF conditions

For conditions not included in QOF, relationships were more consistent across year 1 and year 2 following diagnosis (Figure 2). The 18–40-year age group had the highest rate of codes in both year 1 and year 2, with only small differences between other age groups. There was no difference in the rate of codes in males and females in year 1, but males had a lower rate of codes in year 2. Lower rates of codes were found in people of non-White ethnicities

For peer review only

compared to people of White ethnicity, except for South Asian ethnicity in year 2. Similar to QOF conditions, there was a strong trend towards higher code rates in year 1 and year 2 with decreasing deprivation.

Associations with calendar year

For both QOF and non-QOF conditions, code rates were similar for conditions diagnosed in 2016 and 2017 compared with 2015 (Figures 1 and 2). For codes in year 1, rates for conditions diagnosed in 2018 were similar to 2015, but rates for diseases diagnosed in 2019 were 5% and 6% lower than 2015 for QOF and non-QOF conditions, respectively. For codes in year 2, rates were significantly lower in 2018 (9% and 9% lower for QOF and non-QOF, respectively) and 2019 (21% and 21% lower for QOF and non-QOF, respectively) compared to 2015.

Adjustment for total number of consultations

A sensitivity analysis was used to adjust for total number of consultations in year 1 or year 2 from diagnosis (Tables A5-A8). Total number of consultations in each year were strongly linked to the rate of codes. For newly diagnosed QOF conditions, the associations with age, sex and ethnicity in years 1 and 2 remained significant after adjustment (Tables A5-A6). However, the association with deprivation was attenuated, although there remained an association with higher rates of codes with lower deprivation in year 2. For newly diagnosed non-QOF conditions, after adjustment for consultations, age and ethnicity remained significantly associated, but males had significantly higher rates of codes than females (Tables A7-A8). Associations with deprivation were attenuated, but there remained a small but significant association in year 2.

Figure 2: Associations of rate of codes in year one and year two following diagnosis with patient characteristics and calendar year, for conditions included in the Quality and Outcomes Framework (QOF)

Figure 3: Associations of rate of codes in year one and year two following diagnosis with patient characteristics and calendar year, for conditions not included in the Quality and Outcomes Framework (QOF)

For peer review only

Discussion

With an increased use of NLP methods incorporating temporally-ordered code sequences in the primary care EHR, we need to better understand the structure and frequency of repeated occurrences of diagnostic codes. Our study demonstrates significant associations in the frequency of codes for newly diagnosed conditions according to patient socio-demographic factors, GP practice, disease inclusion in QOF, and calendar year. We are unable to fully assess the extent to which the relationships in our study are explained by the quality of coding, or by how patients use healthcare services for a particular condition. However, a sensitivity analysis adjusting for total number of yearly consultations per patient yielded similar results, suggesting that variation in coding quality is likely to play a role. Our findings have implications for researchers using code sequences, emphasising the importance of considering these factors as potential sources of bias.

Patient socio-demographics

Patient characteristics including age, sex and ethnicity were strongly linked to code frequency, although associations were inconsistent across QOF and non-QOF conditions, and for QOF conditions, were not consistent across the first and second year from diagnosis. People of non-White ethnicity, for example, had lower code rates for QOF conditions in year 1, but higher in year 2, compared to people of White ethnicity. We found consistent patterns with deprivation, with lower code frequency in people living in more deprived areas. A sensitivity analysis adjusting for total number of consultations attenuated the association with deprivation, suggesting that the relationship of code frequency with deprivation was partially explained by total primary care contacts. These findings likely point to differences in the mix of conditions between patient groups, healthcare seeking behaviours, or access to care. For example, people living in areas of socio-economic deprivation may be less likely to attend for screening, preventive care and ongoing management of chronic diseases. Previous research also suggests that although rates of appointments are similar across deciles of socioeconomic deprivation,[25] the rate of missed appointments increases and consultation length decreases with increasing deprivation, which may impact on code frequency for these groups, rather than indicating differences in healthcare need.[26,27]

GP practice

Substantial variation was found in the frequency of codes between GP practices, which persisted after accounting for differences in patient mix in terms of age, sex, deprivation,

For peer review only

ethnicity, number of chronic conditions and in year of diagnosis. Although this may indicate unmeasured confounding in the characteristics of patients between practices, it likely represents policies and practices that influence coding which vary between organisations and clinicians.[9] For example, some GP practices may be more rigorous about coding data in clinical consultations and in correspondence from specialist services on diagnoses made in secondary care. Previous research has suggested that clinicians are more similar to those in the same practice than they are to clinicians in different practices with respect to treatment and diagnostic decisions.[28] Variation between clinicians in coding practices is likely to be significant both within and between practices, but this information was not accessible for the study, and its analysis would introduce multiple hierarchical dependencies outside the scope of this work. Future work could consider individual clinician effects on coding practices in the EHR.

QOF and non-QOF conditions

Code frequency was significantly higher for conditions included in QOF compared to conditions not included. Previous research has highlighted changes to policies and procedures within GP practices to meet targets, including improved disease registries, which may lead to an increased likelihood of a code being entered for a given condition. We found substantial variation between GP practices in the mean code frequency for QOF conditions, but interestingly, this was strongly correlated (r=0.88 and Figure 1) with code frequency for non-QOF conditions, suggesting that practice-level effects impact on coding across all conditions, rather than specifically those incentivised by QOF. However, it is not possible in our study to determine whether differences in code frequency between QOF and non-QOF conditions are explained by greater healthcare need or an increased number of healthcare contacts for QOF conditions, or are explained by higher likelihood of a condition being coded when a patient presents.

Calendar year

Accounting for calendar time in analyses of patient trajectories is a methodological concern, as the further back in time in the medical record, particularly before the advent of the EHR and QOF, the greater the chance that coding practices, and even disease categories, vary.[29] Although our study started relatively recently in 2015, and we cannot infer code frequency before this time, we found consistency in code frequency over a short time-span from 2015-2017. The decline in year 1 codes in 2019, and year 2 codes in 2018 and 2019 likely relates to

For peer review only

the impact of the COVID-19 pandemic which impacted significantly on health services in England from March 2020.[30] Previous studies have shown reductions in patients presenting with particular conditions, and a reduction in appointment numbers in primary and secondary healthcare in England. Analyses reliant on coding frequency should therefore consider using calendar year in addition to patient age in modelling patient trajectories, or limiting analyses to defined time period.

Strengths and limitations

A strength of our study is the inclusion of a large number of patients from a representative sample of primary care in England which makes our findings generalisable to the national population.[15] We included only patients with newly incident diseases to minimise potential confounding from diseases diagnosed historically, some of which might no longer be active. We also only included patients with continuous follow-up over the study period and with at least one year of full practice registration to reduce bias from overestimation of incidence immediately following registration.[17] We also excluded patients who died less than one year from a new diagnosis, which may impact on disease frequency estimates for disease which have poor survival. We considered using annualised rates for those with less than a full year of follow-up, but this resulted in very high annualised counts for some individuals with short follow-up and might introduce additional bias if patients were to seek out care in advance of re-registering at another GP practice.

Our study has focussed on structured healthcare data, whereas much of the consultation is recorded as unstructured ‘free-text’.[30] Although unstructured primary care data contains much richer information on the details of a presentation that may not be fully reflected in the coded entries, this information is not currently available from CPRD, but research in future could examine the agreement between structured and unstructured primary care EHR data. This would allow a more robust estimation of the content and diseases covered during a consultation. We stratified conditions according to QOF status given our hypothesis that it may influence coding frequency. However, we also found variation within categories; for example, polymyalgia rheumatica and motor neurone disease, which are not included in QOF, had high number of yearly codes, whereas cardiovascular events such as Transient Ischaemic Attack, included in QOF, had low yearly codes. Given the general, comparative nature of this paper, and its aim to examine relationships over many conditions, a condition-specific analysis of coding frequency was out of scope.

For peer review only

Implications for research

Our findings have implications for researchers using code sequences recorded in primary care structured data. The frequency of repeated diagnostic codes relates to patient and condition-specific factors, coding incentives and practice-level factors. Although we cannot determine if these findings represent disease burden and healthcare need, it is likely that biases in coding operate at various levels. Specific approaches to reduce the impact of bias will depend on the methodology, but our work does suggest general principles.

Firstly, to consider the potential for bias within the data source and whether stratification may reduce it, for example, by selecting a smaller number of healthcare organisations or a narrower time period. Secondly, to consider adjustment or inclusion of patient, condition, GP practice and calendar year variables within analytical models. However, such an approach is not always recommended, particularly if prediction is the aim, as inclusion of factors such as ethnicity in algorithms may reinforce existing bias.[31] In NLP, text style transfer is often used as a method to control for different styles of writing, which may have relevance to approaches to account for the different coding styles of clinicians.[32] However, these approaches are complicated within the EHR as people are likely to see multiple different clinicians over time, with a small set of codes recorded at each visit. Finally, it is vital that generated representations or predictions from modelling are evaluated in different patient subgroups.

Implications for clinical practice

Although difficult to determine the extent to which our findings are attributed to coding quality versus healthcare utilisation, previous studies have reported variability in coding across practices for specific conditions.[33,34] This highlights a need to improve the quality of coding in primary care, given its impact on the reliability and usefulness of the data for secondary purposes such as research. Improving the quality of coding in primary care poses several challenges, due to the different incentives for clinicians, who document most of the consultation in free text.[7] Potential strategies include implementing structured templates for recording consultations, or developing NLP methods capable of interpreting and codifying the free-text documented during clinical encounters, without adding to clinician workload.[7]

Conclusion

For peer review only

Our study found significant variation in the frequency of diagnostic codes recorded in the primary care EHR after diagnosis, related to patient socio-demographics, coding incentives and GP practice, and a significant reduction in the frequency of codes associated with the onset of the COVID-19 pandemic. These factors should be considered by researchers using NLP methods, or other approaches using temporally ordered sequences of codes in primary care EHRs, to reduce the risk of bias.

Funding

This research is funded through a clinical PhD fellowship awarded to TB from the Wellcome Trust 4i programme at Imperial College London (grant number N/A). JC acknowledge support from the Wellcome Trust (grant number N/A). MB acknowledges support from EPSRC grant EP/N014529/1 supporting the EPSRC Centre for Mathematics of Precision Healthcare. TW, AM and PA acknowledge support from the National Institute for Health and Care Research (NIHR) under the Applied Research Collaboration (ARC) Northwest London (grant number N/A). The views expressed in this publication are those of the authors and not necessarily those of the NHS, the NIHR, the Wellcome Trust or the Department of Health and Social Care.

Competing interests

The authors have no competing interests to declare

Contributor statement

TB conceptualised the study, conducted the data management and formal analysis and wrote the first draft of the manuscript. TB, JS, DS, TW, AM, MB and PA contributed to the study design, methodology, interpretation of findings and reviewing and editing the manuscript. TB is the guarantor and accepts full responsibility for the work and the conduct of the study, had access to the data, and controlled the decision to publish. The corresponding author attests that all listed authors meet authorship criteria and that no others meeting the criteria have been omitted.

Data sharing

The data used in this study are not publicly available as access is subject to approval processes. More information is available from CPRD: https://cprd.com/research-applications

For peer review only

Ethics approval

Data access to the Clinical Practice Research Datalink (CPRD) and ethical approval was granted by CPRD's Research Data Governance Process on 28th April 2022 (Protocol reference: 22_001818).

Acknowledgements

Data management was provided by the Big Data and Analytical Unit (BDAU) at the Institute of Global Health Innovation (IGHI).

For peer review only

References

1. Li, Y. *et al.* BEHRT: Transformer for Electronic Health Records. *Sci Rep* **10**, 7155 (2020).
2. Solares, J. R. A. *et al.* Transfer Learning in Electronic Health Records through Clinical Concept Embedding. 1–14 (2021).
3. Altuncu, M. T., Mayer, E., Yaliraki, S. N. & Barahona, M. From free text to clusters of content in health records: an unsupervised graph partitioning approach. *Applied Network Science* **4**, 2 (2019).
4. Kraljevic, Z. *et al.* Multi-domain clinical natural language processing with MedCAT: The Medical Concept Annotation Toolkit. *Artif Intell Med* **117**, 102083 (2021).
5. Rasmy, L., Xiang, Y., Xie, Z., Tao, C. & Zhi, D. Med-BERT: pretrained contextualized embeddings on large-scale structured electronic health records for disease prediction. *npj Digit. Med.* **4**, 1–13 (2021).
6. Choi, E. *et al.* Multi-layer representation learning for medical concepts. *Proceedings of the ACM SIGKDD International Conference on Knowledge Discovery and Data Mining* **13-17-Augu**, 1495–1504 (2016).

7. Shemtob, L., Beaney, T., Norton, J. & Majeed, A. How can we improve the quality of data collected in general practice? *BMJ* **380**, e071950 (2023).

8. Khan, N. F., Harrison, S. E. & Rose, P. W. Validity of diagnostic coding within the General Practice Research Database: a systematic review. *British Journal of General Practice* **60**, e128--e136 (2010).

9. Verheij, R. A., Curcin, V., Delaney, B. C. & McGilchrist, M. M. Possible Sources of Bias in Primary Care Electronic Health Record Data Use and Reuse. *J Med Internet Res* **20**, e185 (2018).

10. Bots, S. H., Groenwold, R. H. H. & Dekkers, O. M. Using electronic health record data for clinical research: a quick guide. *European Journal of Endocrinology* **186**, E1–E6 (2022).

11. Beech, J. & Beccy Baird. GP funding and contracts explained. *The King's Fund* https://www.kingsfund.org.uk/publications/gp-funding-and-contracts-explained (2020).

12. Forbes, L. J., Marchand, C., Doran, T. & Peckham, S. The role of the Quality and Outcomes Framework in the care of long-term conditions: a systematic review. *Br J Gen Pract* **67**, e775 (2017).

13. Minchin, M., Roland, M., Richardson, J., Rowark, S. & Guthrie, B. Quality of Care in the United Kingdom after Removal of Financial Incentives. *New England Journal of Medicine* **379**, 948–957 (2018).

14. Roland, M. & Guthrie, B. Quality and Outcomes Framework: what have we learnt? *BMJ* **354**, i4060 (2016).

15. Wolf, A. *et al.* Data resource profile: Clinical Practice Research Datalink (CPRD) Aurum. *International Journal of Epidemiology* **48**, 1740–1740g (2019).

16. Herrett, E. *et al.* Data Resource Profile: Clinical Practice Research Datalink (CPRD). *International Journal of Epidemiology* **44**, 827–836 (2015).

For peer review only

17. Lewis, J. D., Bilker, W. B., Weinstein, R. B. & Strom, B. L. The relationship between time since registration and measured incidence rates in the General Practice Research Database. *Pharmacoepidemiology and Drug Safety* **14**, 443–451 (2005).

18. Ministry of Housing & Communities & Local Government. English indices of deprivation 2019. https://www.gov.uk/government/statistics/english-indices-of-deprivation-2019.

19. Mathur, R. *et al.* Completeness and usability of ethnicity data in UK-based primary care and hospital databases. *Journal of Public Health* **36**, 684–692 (2014).

20. Head, A. *et al.* Inequalities in incident and prevalent multimorbidity in England, 2004–19: a population-based, descriptive study. *The Lancet Healthy Longevity* **2**, e489–e497 (2021).

21. Kuan, V. *et al.* A chronological map of 308 physical and mental health conditions from 4 million individuals in the English National Health Service. *The Lancet Digital Health* **1**, e63–e77 (2019).

22. Dean, C. B. Testing for Overdispersion in Poisson and Binomial Regression Models. *Journal of the American Statistical Association* **87**, 451–457 (1992).

23. Dunn, P. K. & Smyth, G. K. Randomized Quantile Residuals. *Journal of Computational and Graphical Statistics* **5**, 236–244 (1996).

24. Feng, C., Li, L. & Sadeghpour, A. A comparison of residual diagnosis tools for diagnosing regression models for count data. *BMC Med Res Methodol* **20**, 175 (2020).

25. Fisher, R., Dunn, P., Asaria, M. & Thorlby, R. Comparing general practice in areas of high and low socioeconomic deprivation in England. 30.

26. Ellis, D. A., McQueenie, R., McConnachie, A., Wilson, P. & Williamson, A. E. Demographic and practice factors predicting repeated non-attendance in primary care: a national retrospective cohort analysis. *The Lancet Public Health* **2**, e551–e559 (2017).

For peer review only

27. Gopfert, A., Deeny, S. R., Fisher, R. & Stafford, M. Primary care consultation length by deprivation and multimorbidity in England: an observational study using electronic patient records. *Br J Gen Pract* **71**, e185–e192 (2021).
28. Jong, J. de, Groenewegen, P. & Westert, G. Medical practice variation: does it cluster within general practitioners' practices? in *Morbidity, Performance and Quality in Primary Care* (CRC Press, 2006).
29. Gluckman, P. D. Evolving a definition of disease. *Archives of Disease in Childhood* **92**, 1053 (2007).
30. Majeed, A., Maile, E. J. & Bindman, A. B. The primary care response to COVID-19 in England's National Health Service. *J R Soc Med* **113**, 208–210 (2020).
31. Obermeyer, Z., Powers, B., Vogeli, C. & Mullainathan, S. Dissecting racial bias in an algorithm used to manage the health of populations. *Science* **366**, 447–453 (2019).
32. Jin, D., Jin, Z., Hu, Z., Vechtomova, O. & Mihalcea, R. Deep Learning for Text Style Transfer: A Survey. Preprint at http://arxiv.org/abs/2011.00416 (2021).
33. de Lusignan, S. *et al.* Problems with primary care data quality: osteoporosis as an exemplar. *Inform Prim Care* **12**, 147–156 (2004).
34. Rollason, W., Khunti, K. & de Lusignan, S. Variation in the recording of diabetes diagnostic data in primary care computer systems: implications for the quality of care. *Inform Prim Care* **17**, 113–119 (2009).

Figure 1 legend:

Note: different ranges used in each axis

Figure 2 legend:

For peer review only

Note: Points represent estimates of the incidence rate ratio and bars represent 95% confidence intervals from negative binomial regression models. Corresponding values and coefficients for pre-existing QOF and non-QOF conditions are given in appendix Tables A5 and A6.

Figure 3 legend:

Note: Points represent estimates of the incidence rate ratio and bars represent 95% confidence intervals from negative binomial regression models. Corresponding values and coefficients for pre-existing QOF and non-QOF conditions are given in appendix Tables A7 and A8.

For peer review only

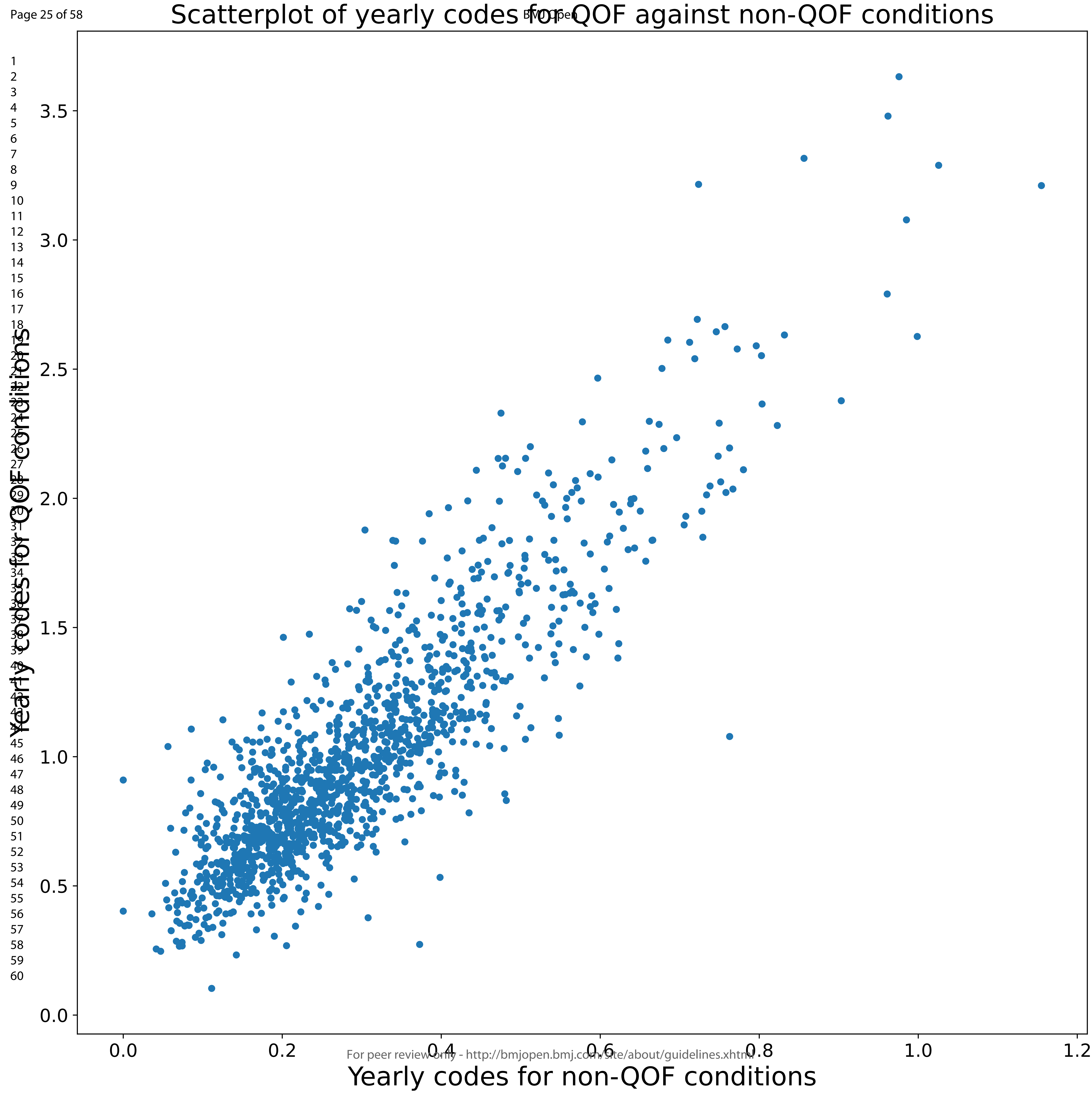

Scatterplot of yearly codes for QOF against non-QOF conditions
Yearly codes for QOF conditions
Yearly codes for non-QOF conditions
0.0
0.5
1.0
1.5
2.0
2.5
3.0
3.5
0.0
0.2
0.4
0.6
0.8
1.0
1.2

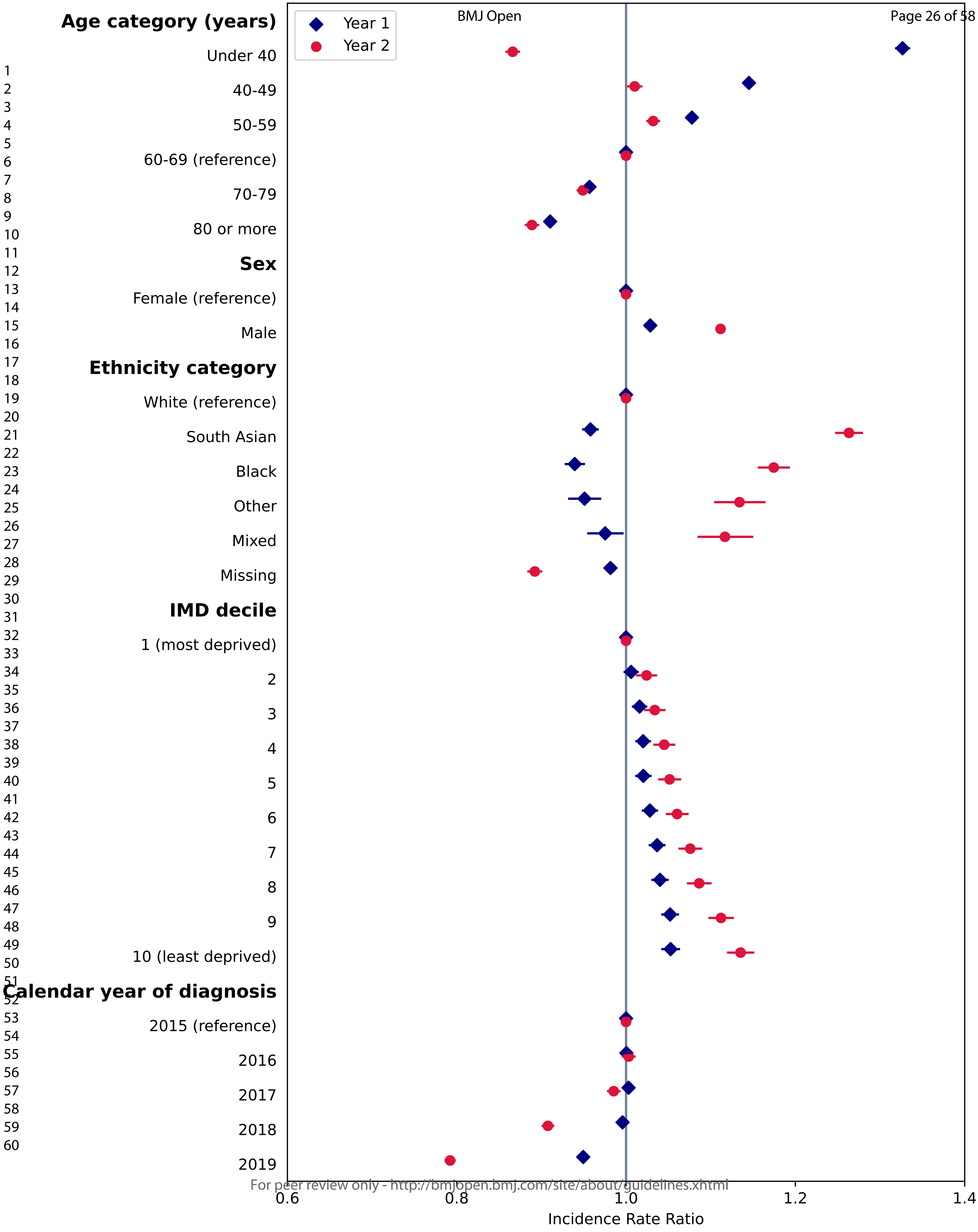
Year 1
Year 2
Age category (years)
Under 40
40-49
50-59
60-69 (reference)
70-79
80 or more
Sex
Female (reference)
Male
Ethnicity category
White (reference)
South Asian
Black
Other
Mixed
Missing
IMD decile
1 (most deprived)
2
3
4
5
6
7
8
9
10 (least deprived)
Calendar year of diagnosis
2015 (reference)
2016
2017
2018
2019
0.6
0.8
1.0
1.2
1.4
Incidence Rate Ratio

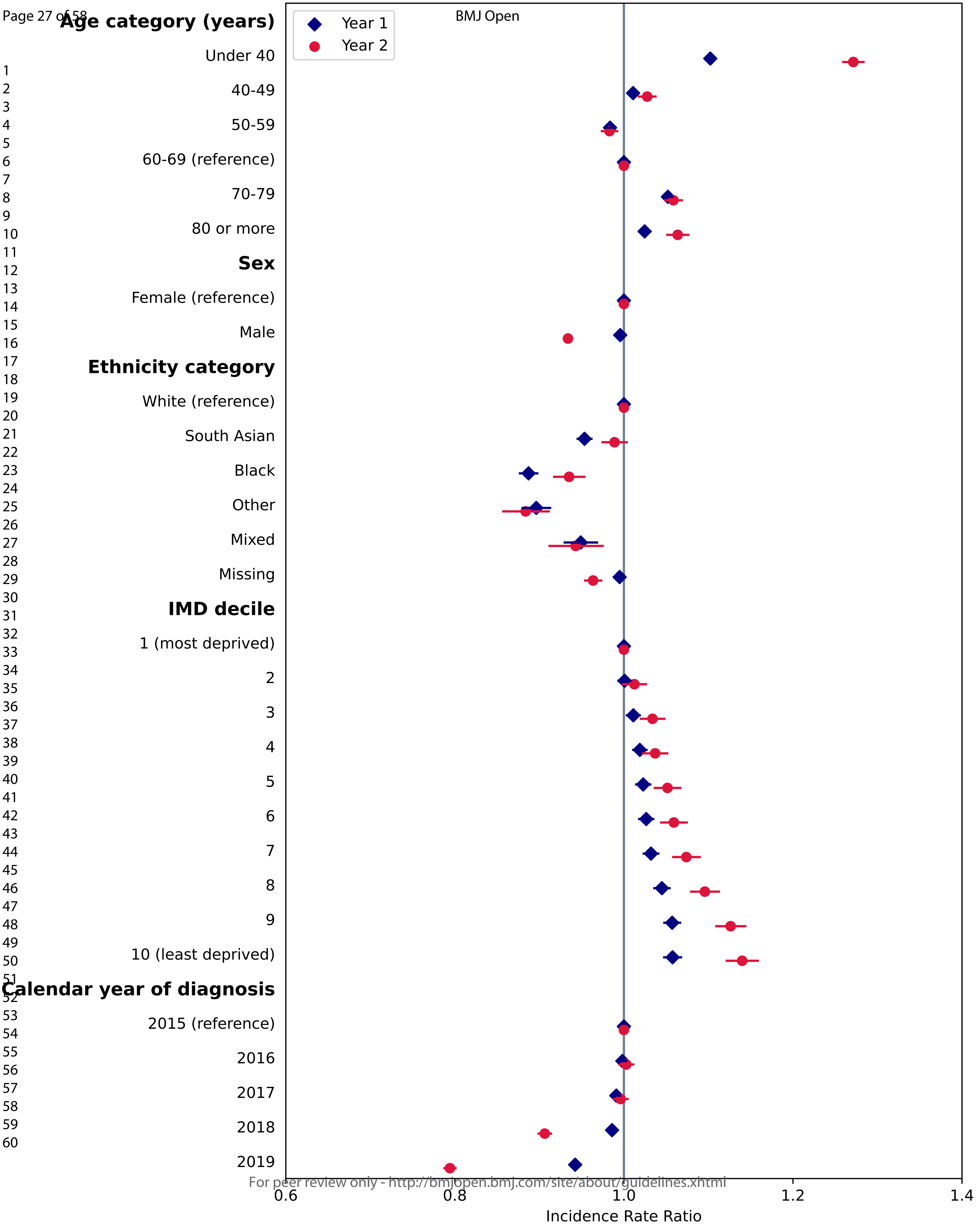
Age category (years)
Under 40
40-49
50-59
60-69 (reference)
70-79
80 or more
Sex
Female (reference)
Male
Ethnicity category
White (reference)
South Asian
Black
Other
Mixed
Missing
IMD decile
1 (most deprived)
2
3
4
5
6
7
8
9
10 (least deprived)
Calendar year of diagnosis
2015 (reference)
2016
2017
2018
2019
Year 1
Year 2
0.6
0.8
1.0
1.2
1.4
Incidence Rate Ratio

Appendix

Identifying potential biases in code sequences in primary care electronic healthcare records: a retrospective cohort study of the determinants of code frequency

Beaney T[1,2] (0000-0001-9709-7264), Clarke J[2], Salman D [1,3], Woodcock T[1], Majeed A[1], Barahona M[2], Aylin P[1]

1. Department of Primary Care and Public Health, Imperial College London, London, W6 8RP, United Kingdom
2. Centre for Mathematics of Precision Healthcare, Department of Mathematics, Imperial College London, London, SW7 2AZ, United Kingdom
3. MSk Lab, Department of Surgery and Cancer, Faculty of Medicine, Imperial College London, London, UK

Corresponding Author:

Dr Thomas Beaney

Department of Primary Care and Public Health, Imperial College London, London, W6 8RP, United Kingdom

Email: thomas.beaney@imperial.ac.uk

For peer review only

Patients were included with continuous registration dates between 1st January 2014 and 31st December 2020. The 1st January 2014 was chosen to allow for a full one year of registration at a GP practice prior to follow-up, to reduce the potential impact of bias from newly registered patients having pre-existing conditions coded for the first time at their new practice. The end date of 31st December 2020 was chosen to provide at least one full year of follow-up for conditions newly diagnosed in 2019. Patients were followed up until the earliest date of death, deregistration and latest date of data extraction from their practice, if after 31st December 2020. The earliest possible censoring date for a patient was 1st January 2021 and the last date of follow-up for a patient was 21st March 2022.

For peer review only

Chronic conditions

Diseases were mapped using code lists developed for the CALIBER study, and adapted for use in multimorbidity in CPRD Aurum.[1,2] We reviewed the codes in these lists, and made amendments to the code lists for diabetes. The 'other/unspecified' diabetes code list contained codes specific to both Type 1 and Type 2 diabetes, and we removed these to ensure the list included only codes where a more specific Type 1 or Type 2 diagnosis was not stated. We added chronic primary pain to the set of included conditions and created a new code list. Previous studies of multimorbidity in primary care settings have found a high prevalence and burden of chronic pain.[3,4] However, in order to avoid double counting of pain related to another chronic condition included, we excluded secondary causes, and included only primary pain conditions.

Assignment to QOF

Diseases were classified as included or not included in QOF by two clinicians with experience working as GPs: TB and DS. The first QOF year in 2004/2005 included eleven diseases, with new conditions added in subsequent years.[5] Rheumatoid arthritis was added to QOF in 2013/2014, but there were no subsequent additions of any of the diseases included in this study.[6] However, hypothyroidism was included in QOF from its start until 2014/15 when it was removed.[7] The thyroid disease category from CALIBER included codes for both hypothyroidism and hyperthyroidism. We therefore excluded the thyroid disease category from comparisons of QOF to avoid any carry-over effect from prior inclusion in QOF, and dilution from non-hypothyroid conditions. The following QOF conditions from 2014/15 to 2019/20 were included:

For peer review only - http://bmjopen.bmj.com/site/about/guidelines.xhtml

1. Coronary Heart Disease
2. Left Ventricular Dysfunction / Heart Failure (from 2006)
3. Stroke (and TIA from 2006)
4. Hypertension
5. Diabetes
6. COPD
7. Epilepsy
8. Cancer
9. Mental Health
10. Asthma
11. Dementia
12. Depression
13. CKD
14. Atrial fibrillation
15. Obesity
16. Learning disabilities
17. Palliative care
18. Smoking
19. Cardio-vascular disease (primary prevention)
20. Peripheral Arterial Disease (PAD)
21. Osteoporosis
22. Rheumatoid arthritis

For analyses of counts per calendar year, the total counts of disease codes were calculated for the first and second year from diagnosis. Counts were stratified according to whether a condition was included in QOF. A patient was included for a given calendar year if they had at least one QOF or non-QOF condition diagnosed in that year, as shown in Table A1.

For peer review only

Table A1: example of the stratification of condition and calendar year for each newly diagnosed condition for three hypothetical patients

Patient	Age	Condition	Calendar year	Count in year one	Count in year two
1	67	QOF	2015	0	0
1	68	QOF	2016	2	0
1	70	QOF	2018	4	2
1	67	Non-QOF	2015	1	1
2	28	Non-QOF	2019	1	2
3	52	QOF	2017	5	4
3	52	Non-QOF	2017	2	2

For peer review only

Statistical analyses

Mixed effects negative binomial models were constructed. We considered use of a zero-inflated model, but coefficients from the logit and negative binomial components of the model were similar, and so in the interests of interpretable findings, the more parsimonious negative binomial model was selected.

Equation for the mixed effects negative binomial regression model, including fixed effects for calendar year and GP practice and random effects for patient:

$$log(y_{i,j}) = \beta_0 + \beta_1 age_{i,j} + \beta_2 gender_{i,j} + \beta_3 ethnicity_{i,j} + \beta_4 IMD_{i.j} + \beta_5 year_{i.j} + \beta_6 GP_{i.j} + u_j$$

where *i* represents QOF or non-QOF conditions newly diagnosed in patient *j* and $y_{i,j}$ is the count of codes in the given year.

For peer review only

A2: Frequency and percentage of pre-existing diseases (as of 1st January 2015) for all 3,113,724 eligible patients

Pre-existing disease	Frequency	Percentage
Hypertension	751009	24.12%
Enthesopathy and synovial disorder	736087	23.64%
Dermatitis	710945	22.83%
Depression	568871	18.27%
Anxiety disorders	507406	16.30%
Allergic and chronic rhinitis	477053	15.32%
Asthma	456335	14.66%
Osteoarthritis (excl spine)	444668	14.28%
Gastro-oesophageal reflux disease	301839	9.69%
Obesity	294916	9.47%
Diabetes Mellitus: other or not specified	285681	9.17%
Hearing loss	279470	8.98%
Migraine	270415	8.68%
Type 2 Diabetes Mellitus	255578	8.21%
Irritable bowel syndrome	246744	7.92%
Abdominal Hernia	237968	7.64%
Acne	225183	7.23%
Chronic sinusitis	212496	6.82%
Thyroid Disease	204639	6.57%
Spondylosis	181722	5.84%
Gastritis and duodenitis	181668	5.83%
Cataract	160486	5.15%
Chronic Kidney Disease	158134	5.08%
Coronary Heart Disease (not otherwise specified)	144806	4.65%
Seborrheic dermatitis	143168	4.60%
Urinary Incontinence	137919	4.43%
Alcohol Misuse	132717	4.26%
Psoriasis	132694	4.26%
Diaphragmatic hernia	131539	4.22%
Diverticular Disease	131332	4.22%
Tinnitus	123308	3.96%
Gout	120568	3.87%
Stable Angina	120309	3.86%
Intervertebral disc disorders	117787	3.78%
Anaemia: other	116859	3.75%
Diabetic Eye Disease	102901	3.30%
Rosacea	96511	3.10%
Dysmenorrhoea	94881	3.05%

For peer review only

Benign Prostatic Hyperplasia	92304	2.96%
Osteoporosis	91850	2.95%
Primary Malignancy: Skin	89500	2.87%
COPD	84482	2.71%
Atrial Fibrillation	80645	2.59%
Peripheral Neuropathy	77117	2.48%
Chronic Fatigue Syndrome	67489	2.17%
Myocardial Infarction	67215	2.16%
Vitamin B12 deficiency anaemia	64015	2.06%
Glaucoma	58081	1.87%
Epilepsy	53058	1.70%
Stroke: not otherwise specified	50614	1.63%
Substance Misuse	50251	1.61%
Primary Malignancy: Breast	49737	1.60%
Venous thromboembolic disease (Excl PE)	47013	1.51%
Transient ischaemic attack	44616	1.43%
Fibromatosis	42701	1.37%
Neuropathic Bladder	42008	1.35%
Raynaud's syndrome	38879	1.25%
Endometriosis	37868	1.22%
Sleep apnoea	35743	1.15%
Heart failure	35364	1.14%
Peripheral Vascular Disease	32852	1.06%
Rheumatoid Arthritis	32070	1.03%
Macular degeneration	30761	0.99%
Chronic primary pain	29506	0.95%
Anterior and Intermediate Uveitis	28838	0.93%
Visual impairment and blindness	28372	0.91%
Polymyalgia Rheumatica	27447	0.88%
Primary Malignancy: Prostate	26288	0.84%
Ulcerative colitis	22236	0.71%
Nonrheumatic mitral valve disorders	20980	0.67%
Spinal stenosis	20820	0.67%
Nonrheumatic aortic valve disorders	20695	0.66%
Schizophrenia	20394	0.65%
Type 1 Diabetes Mellitus	19978	0.64%
Unstable Angina	18925	0.61%
Trigeminal neuralgia	18854	0.61%
Scleritis and episcleritis	18830	0.60%
Fatty Liver	18774	0.60%
Barrett's oesophagus	18152	0.58%
Supraventricular tachycardia	18128	0.58%
Intellectual disability	18073	0.58%
Pancreatitis	18043	0.58%

For peer review only

Bronchiectasis	18006	0.58%
Primary Malignancy: Melanoma	17594	0.57%
Personality disorders	17448	0.56%
Alopecia areata	17111	0.55%
Primary Malignancy: Bowel	16746	0.54%
Obsessive-compulsive disorder	15553	0.50%
Polycystic ovarian syndrome	14606	0.47%
Crohn's disease	14445	0.46%
Folate deficiency anaemia	13853	0.44%
Retinal vascular occlusions	13829	0.44%
Obstructive and reflux uropathy	13725	0.44%
Ischaemic stroke	13451	0.43%
Hidradenitis suppurativa	13305	0.43%
Vitiligo	13218	0.42%
Meniere's Disease	13192	0.42%
Bipolar affective disorder and mania	12856	0.41%
Coeliac disease	12625	0.41%
Diabetic Neuropathy	12517	0.40%
Chronic viral hepatitis	11885	0.38%
Thrombophilia	11527	0.37%
Psoriatic Arthritis	11201	0.36%
Eating Disorders	11171	0.36%
Dementia	10297	0.33%
Spondylolisthesis	10229	0.33%
Secondary Thrombocytopaenia	9800	0.31%
Congenital Septal Defect	9203	0.30%
Sarcoidosis	9090	0.29%
Multiple sclerosis	9070	0.29%
Benign essential tremor	9008	0.29%
Right bundle branch block combinations	8160	0.26%
Primary Malignancy: Bladder	8066	0.26%
Primary Malignancy: other	8021	0.26%
Glomerulonephritis	7950	0.26%
Autism and Asperger's syndrome	7920	0.25%
Non-Hodgkin Lymphoma	7579	0.24%
Hyperparathyroidism	7437	0.24%
Pleural effusion	7368	0.24%
Hyperkinetic disorders	7056	0.23%
Ankylosing spondylitis	7044	0.23%
Lupus Erythematosus	6976	0.22%
Cirrhosis	6768	0.22%
Alcoholic liver disease	6621	0.21%
Left bundle branch block	6512	0.21%
Subarachnoid haemorrhage	6158	0.20%

For peer review only

Collapsed vertebra	6082	0.20%
Autonomic Neuropathy	5496	0.18%
Cardiomyopathy: other	5465	0.18%
Parkinson's disease	5333	0.17%
Leukaemia	5243	0.17%
Giant Cell arteritis	5225	0.17%
Hyposplenism	4737	0.15%
HIV	4697	0.15%
Endometrial hyperplasia and hypertrophy	4655	0.15%
Primary Malignancy: Uterus	4589	0.15%
Sjogren's Syndrome	4559	0.15%
Spina bifida	4427	0.14%
Cerebral Palsy	4011	0.13%
Primary Thrombocytopaenia	3979	0.13%
Pleural plaque	3972	0.13%
Abdominal Aortic Aneurysm	3931	0.13%
Atrioventricular blocks	3920	0.13%
Chronic Cystitis	3892	0.12%
Intracerebral haemorrhage	3815	0.12%
Primary Malignancy: Ovary	3689	0.12%
Primary Malignancy: Cervix	3500	0.11%
Asbestosis	3358	0.11%
Other haemolytic anaemias	3152	0.10%
Primary Malignancy: Testis	3133	0.10%
Thalassaemia	3055	0.10%
Hypertrophic Nasal Turbinates	3022	0.10%
Primary Malignancy: Kidney	2988	0.10%
Polycythaemia vera	2864	0.09%
Primary Malignancy: Oropharyngeal	2809	0.09%
Autoimmune liver disease	2792	0.09%
Ventricular tachycardia	2720	0.09%
Secondary polycythaemia	2625	0.08%
Posterior Uveitis	2540	0.08%
Pulmonary Fibrosis	2523	0.08%
Hodgkin Lymphoma	2384	0.08%
Hypersplenism	2362	0.08%
Dilated cardiomyopathy	2359	0.08%
Primary Malignancy: Lung	2244	0.07%
Primary Malignancy: Thyroid	2172	0.07%
Rheumatic Valve Disorder	2034	0.07%
Secondary Malignancy_other	1975	0.06%
Down's syndrome	1928	0.06%
Multiple valve disorder	1834	0.06%
Idiopathic Intracranial Hypertension	1823	0.06%

For peer review only

Hypertrophic Cardiomyopathy	1779	0.06%
Oesophageal varices	1716	0.06%
Plasma Cell Malignancy	1610	0.05%
Scleroderma	1566	0.05%
Pericardial Effusion	1509	0.05%
Myasthenia gravis	1407	0.05%
Primary pulmonary hypertension	1345	0.04%
Sick sinus syndrome	1231	0.04%
Aplastic anaemias	1172	0.04%
Primary Malignancy: Brain	1131	0.04%
Immunodeficiencies	1071	0.03%
Cystic Fibrosis	985	0.03%
Primary Malignancy: Oesophageal	955	0.03%
Myelodysplastic Syndrome	927	0.03%
Portal hypertension	919	0.03%
Sickle Cell Disease	887	0.03%
Secondary pulmonary hypertension	824	0.03%
Angiodysplasia of colon	777	0.02%
Primary Malignancy: Bone	741	0.02%
Primary Malignancy: Stomach	694	0.02%
Hepatic failure	632	0.02%
Secondary Malignancy: Lymph Nodes	565	0.02%
Secondary Malignancy: Liver	491	0.02%
Tubulo-interstitial nephritis	365	0.01%
Motor neurone disease	347	0.01%
Primary Malignancy: Pancreas	302	0.01%
Enteropathic arthropathy	291	0.01%
Primary Malignancy: Liver	233	0.01%
Secondary Malignancy: Lung	223	0.01%
Secondary Malignancy: Bone	187	0.01%
Primary Malignancy: Biliary Tract	129	<0.01%
Secondary Malignancy: Brain	50	<0.01%
Secondary Malignancy: Peritoneum	24	<0.01%
Secondary Malignancy: Bowel	11	<0.01%
Secondary Malignancy: Adrenal Gland	*	<0.01%
Primary Malignancy: Multiple Sites	*	<0.01%
Primary Malignancy: Mesothelioma	*	<0.01%
Secondary Malignancy: Pleura	*	<0.01%

* diseases with frequency <10 suppressed as small counts

For peer review only

Table A3: characteristics of the 3,060,391 ineligible patients with no incident diseases over the study period

Patient characteristic	Total	Percent
**Age (years)**		
18-40	1476341	48.2%
40-49	689779	22.5%
50-59	435517	14.2%
60-69	291093	9.5%
70-79	129375	4.2%
80+	38286	1.3%
**Gender**		
Female	1357049	44.3%
Male	1703284	55.7%
Indeterminate	58	0.0%
**Total**	**3060391**	

For peer review only

Table A4: distribution of yearly codes over the whole follow-up period for each condition, ordered by median

Disease	5th centile	Median	95th centile	Mean	Standard deviation
Diabetes Mellitus_other or not specified	0.00	2.99	6.88	3.08	2.22
Polymyalgia Rheumatica	0.00	1.05	6.32	1.82	2.29
Motor neurone disease	0.00	0.95	12.15	2.86	5.41
Dementia	0.00	0.93	4.36	1.39	1.80
Type 2 Diabetes Mellitus	0.00	0.89	4.59	1.41	1.73
Type 1 Diabetes Mellitus	0.00	0.88	6.31	1.71	2.41
Depression	0.00	0.83	4.54	1.36	1.76
COPD	0.00	0.77	3.77	1.17	1.43
Heart failure	0.00	0.73	5.48	1.46	2.21
Rheumatoid Arthritis	0.00	0.70	5.50	1.43	2.23
Primary Malignancy_Mesothelioma	0.00	0.67	9.16	1.78	3.18
Primary Malignancy_Pancreas	0.00	0.67	13.41	2.63	5.12
Primary Malignancy_Brain	0.00	0.66	10.60	2.15	3.96
Primary Malignancy_Oesophageal	0.00	0.64	10.86	2.44	4.95
Myasthenia gravis	0.00	0.62	5.61	1.48	2.66
Multiple sclerosis	0.00	0.59	5.63	1.40	2.41
Parkinson's disease	0.00	0.59	4.52	1.20	1.77
Vitamin B12 deficiency anaemia	0.00	0.56	4.60	1.24	1.67
Bipolar affective disorder and mania	0.00	0.56	4.99	1.30	2.15
Plasma Cell Malignancy	0.00	0.54	10.32	2.15	4.67
Hypertension	0.00	0.54	2.95	0.88	1.12
Atrial Fibrillation	0.00	0.51	3.47	0.97	1.47
Primary Malignancy_Prostate	0.00	0.51	6.11	1.46	2.48
Intellectual disability	0.00	0.49	5.19	1.47	1.91
Primary Malignancy_Lung	0.00	0.45	8.17	1.73	3.55
Primary Malignancy_Biliary Tract	0.00	0.45	8.96	1.89	4.73
Giant Cell arteritis	0.00	0.44	5.73	1.36	2.47
Crohn's disease	0.00	0.42	5.41	1.24	2.32
Primary Malignancy_Breast	0.00	0.39	5.25	1.21	2.47
Hodgkin Lymphoma	0.00	0.38	5.41	1.24	2.55
Ulcerative colitis	0.00	0.38	4.27	1.00	1.87
Primary Malignancy_Oropharyngeal	0.00	0.37	6.84	1.44	2.95
Non-Hodgkin Lymphoma	0.00	0.37	5.52	1.22	2.53
Leukaemia	0.00	0.37	5.19	1.17	2.58
Secondary Malignancy_Brain	0.00	0.37	7.68	1.45	2.74
Stroke_not otherwise specified	0.00	0.34	2.11	0.59	0.89
Idiopathic Intracranial Hypertension	0.00	0.34	3.81	0.92	1.76
Thyroid Disease	0.00	0.33	2.56	0.68	1.16
Asthma	0.00	0.32	2.33	0.63	0.99
Primary Malignancy_Stomach	0.00	0.32	6.93	1.45	3.30
Chronic primary pain	0.00	0.32	3.23	0.79	1.34
Coronary Heart Disease (not otherwise specified)	0.00	0.31	2.02	0.56	0.85
Epilepsy	0.00	0.31	3.66	0.92	1.95
Psoriatic Arthritis	0.00	0.30	3.68	0.87	1.63

For peer review only

Chronic Fatigue Syndrome	0.00	0.29	3.22	0.76	1.31
Primary Malignancy_Bowel	0.00	0.29	5.25	1.15	2.88
Anxiety disorders	0.00	0.29	2.99	0.73	1.29
Primary Malignancy_Thyroid	0.00	0.28	4.05	0.88	1.76
Personality disorders	0.00	0.28	4.35	0.99	2.05
Schizophrenia	0.00	0.27	3.36	0.78	1.52
Primary Malignancy_Cervix	0.00	0.27	5.26	1.17	2.77
Autoimmune liver disease	0.00	0.26	3.63	0.85	1.82
Myelodysplastic Syndrome	0.00	0.26	4.88	1.15	2.95
Bronchiectasis	0.00	0.24	3.03	0.70	1.31
Hyperkinetic disorders	0.00	0.24	3.11	0.72	1.34
Primary Malignancy_Ovary	0.00	0.24	6.15	1.24	2.87
Primary Malignancy_Liver	0.00	0.23	3.64	0.95	2.99
Coeliac disease	0.00	0.23	2.13	0.52	0.85
Lupus Erythematosus	0.00	0.22	3.52	0.83	1.87
Myocardial Infarction	0.00	0.21	2.44	0.58	1.04
Primary Malignancy_Bone	0.00	0.21	4.03	0.97	3.29
Secondary Malignancy_other	0.00	0.21	5.92	1.18	2.65
Peripheral Vascular Disease	0.00	0.20	2.73	0.75	2.53
Ankylosing spondylitis	0.00	0.20	3.00	0.69	1.47
Primary Malignancy_Bladder	0.00	0.20	4.38	0.90	2.05
Primary Malignancy_Testis	0.00	0.20	3.58	0.81	1.50
Sarcoidosis	0.00	0.19	3.36	0.72	1.53
Abdominal Hernia	0.00	0.19	1.55	0.40	0.68
Secondary Malignancy_Peritoneum	0.00	0.19	4.21	1.30	3.31
Scleroderma	0.00	0.19	3.00	0.71	1.88
Primary Malignancy_Melanoma	0.00	0.18	3.06	0.67	1.71
Gout	0.00	0.17	1.74	0.43	0.73
Barrett's oesophagus	0.00	0.16	1.40	0.35	0.57
Glomerulonephritis	0.00	0.16	3.26	0.74	1.69
Osteoporosis	0.00	0.15	1.52	0.38	0.65
Primary Malignancy_Uterus	0.00	0.15	3.90	0.81	2.16
Cirrhosis	0.00	0.15	2.88	0.63	1.40
Diabetic Eye Disease	0.00	0.15	1.61	0.40	0.68
Intracerebral haemorrhage	0.00	0.15	2.58	0.56	1.10
Primary Malignancy_Kidney	0.00	0.14	2.93	0.66	1.67
Dilated cardiomyopathy	0.00	0.14	1.99	0.46	0.93
Eating Disorders	0.00	0.14	4.03	0.84	2.38
Abdominal Aortic Aneurysm	0.00	0.00	1.35	0.26	0.58
Acne	0.00	0.00	1.26	0.30	0.50
Alcohol Misuse	0.00	0.00	0.94	0.20	0.66
Alcoholic liver disease	0.00	0.00	1.90	0.42	1.09
Allergic and chronic rhinitis	0.00	0.00	0.56	0.10	0.27
Alopecia areata	0.00	0.00	0.87	0.17	0.45
Anaemia_other	0.00	0.00	1.49	0.33	0.78
Angiodysplasia of colon	0.00	0.00	0.87	0.17	0.49
Anterior and Intermediate Uveitis	0.00	0.00	1.18	0.25	0.66
Aplastic anaemias	0.00	0.00	2.19	0.47	1.42
Asbestosis	0.00	0.00	0.96	0.20	0.65
Atrioventricular blocks	0.00	0.00	0.64	0.11	0.33

For peer review only - http://bmjopen.bmj.com/site/about/guidelines.xhtml

Autism and Asperger's syndrome	0.00	0.00	1.10	0.25	0.58
Autonomic Neuropathy	0.00	0.00	2.46	0.47	1.34
Benign Prostatic Hyperplasia	0.00	0.00	1.08	0.25	0.50
Benign essential tremor	0.00	0.00	1.11	0.22	0.53
Cardiomyopathy_other	0.00	0.00	1.94	0.41	0.90
Cataract	0.00	0.00	1.16	0.27	0.50
Cerebral Palsy	0.00	0.00	0.73	0.16	0.48
Chronic Cystitis	0.00	0.00	1.88	0.37	1.03
Chronic Kidney Disease	0.00	0.00	1.16	0.26	0.65
Chronic sinusitis	0.00	0.00	0.72	0.13	0.39
Chronic viral hepatitis	0.00	0.00	1.89	0.40	0.90
Collapsed vertebra	0.00	0.00	1.64	0.34	0.77
Congenital Septal Defect	0.00	0.00	1.21	0.24	0.62
Cystic Fibrosis	0.00	0.00	2.21	0.31	1.00
Dermatitis	0.00	0.00	0.76	0.15	0.43
Diabetic Neuropathy	0.00	0.00	1.62	0.38	1.44
Diaphragmatic hernia	0.00	0.00	0.81	0.17	0.38
Diverticular Disease	0.00	0.00	0.96	0.20	0.51
Down's syndrome	0.00	0.00	0.48	0.10	0.19
Dysmenorrhoea	0.00	0.00	0.78	0.15	0.38
Endometrial hyperplasia and hypertrophy	0.00	0.00	0.90	0.17	0.57
Endometriosis	0.00	0.00	2.08	0.44	1.06
Enteropathic arthropathy	0.00	0.00	1.28	0.38	0.99
Enthesopathy and synovial disorder	0.00	0.00	0.86	0.18	0.43
Fatty Liver	0.00	0.00	0.75	0.14	0.34
Fibromatosis	0.00	0.00	0.85	0.17	0.39
Folate deficiency anaemia	0.00	0.00	0.52	0.09	0.25
Gastritis and duodenitis	0.00	0.00	0.73	0.14	0.39
Gastro-oesophageal reflux disease	0.00	0.00	0.88	0.18	0.43
Glaucoma	0.00	0.00	1.46	0.31	0.62
HIV	0.00	0.00	2.07	0.41	0.92
Hearing loss	0.00	0.00	0.77	0.16	0.34
Hepatic failure	0.00	0.00	2.22	0.46	1.07
Hidradenitis suppurativa	0.00	0.00	1.92	0.43	1.11
Hyperparathyroidism	0.00	0.00	1.84	0.41	0.84
Hypersplenism	0.00	0.00	0.99	0.21	0.58
Hypertrophic Cardiomyopathy	0.00	0.00	2.23	0.49	1.00
Hypertrophic Nasal Turbinates	0.00	0.00	0.28	0.04	0.16
Hyposplenism	0.00	0.00	1.50	0.34	0.71
Immunodeficiencies	0.00	0.00	1.62	0.36	1.11
Intervertebral disc disorders	0.00	0.00	1.75	0.36	0.91
Irritable bowel syndrome	0.00	0.00	0.66	0.13	0.32
Ischaemic stroke	0.00	0.00	2.03	0.46	0.99
Left bundle branch block	0.00	0.00	0.77	0.15	0.39
Macular degeneration	0.00	0.00	1.16	0.25	0.71
Meniere's Disease	0.00	0.00	1.58	0.33	0.77
Migraine	0.00	0.00	1.21	0.25	0.61
Multiple valve disorder	0.00	0.00	0.49	0.09	0.33
Neuropathic Bladder	0.00	0.00	0.74	0.15	0.36

For peer review only

Nonrheumatic aortic valve disorders	0.00	0.00	1.42	0.31	0.68
Nonrheumatic mitral valve disorders	0.00	0.00	0.84	0.16	0.52
Obesity	0.00	0.00	0.71	0.15	0.44
Obsessive-compulsive disorder	0.00	0.00	2.55	0.56	1.21
Obstructive and reflux uropathy	0.00	0.00	1.10	0.23	0.63
Oesophageal varices	0.00	0.00	1.62	0.38	0.74
Osteoarthritis (excl spine)	0.00	0.00	1.53	0.34	0.70
Other haemolytic anaemias	0.00	0.00	3.09	0.62	1.64
Pancreatitis	0.00	0.00	2.00	0.44	1.09
Pericardial Effusion	0.00	0.00	1.12	0.21	0.56
Peripheral Neuropathy	0.00	0.00	1.22	0.26	0.81
Pleural effusion	0.00	0.00	1.55	0.32	0.90
Pleural plaque	0.00	0.00	0.74	0.14	0.48
Polycystic ovarian syndrome	0.00	0.00	0.86	0.20	0.34
Polycythaemia vera	0.00	0.00	2.49	0.54	1.30
Portal hypertension	0.00	0.00	0.91	0.18	0.46
Posterior Uveitis	0.00	0.00	1.46	0.33	1.02
Primary Malignancy_Multiple Sites	0.00	0.00	0.00	0.00	0.00
Primary Malignancy_Skin	0.00	0.00	1.30	0.31	0.78
Primary Malignancy_other	0.00	0.00	4.42	0.90	2.44
Primary Thrombocytopaenia	0.00	0.00	2.41	0.59	1.96
Primary pulmonary hypertension	0.00	0.00	1.62	0.32	1.00
Psoriasis	0.00	0.00	1.44	0.32	0.75
Pulmonary Fibrosis	0.00	0.00	2.38	0.53	1.34
Raynaud's syndrome	0.00	0.00	0.85	0.16	0.45
Retinal vascular occlusions	0.00	0.00	1.93	0.42	0.93
Rheumatic Valve Disorder	0.00	0.00	0.70	0.13	0.41
Right bundle branch block combinations	0.00	0.00	0.47	0.08	0.25
Rosacea	0.00	0.00	0.93	0.20	0.41
Scleritis and episcleritis	0.00	0.00	0.70	0.13	0.49
Seborrheic dermatitis	0.00	0.00	0.61	0.11	0.31
Secondary Malignancy_Adrenal Gland	0.00	0.00	1.68	0.42	1.01
Secondary Malignancy_Bone	0.00	0.00	4.78	0.93	2.34
Secondary Malignancy_Bowel	0.00	0.00	6.36	1.41	2.42
Secondary Malignancy_Liver	0.00	0.00	4.82	0.91	2.26
Secondary Malignancy_Lung	0.00	0.00	6.04	1.10	2.27
Secondary Malignancy_Lymph Nodes	0.00	0.00	2.40	0.40	1.31
Secondary Malignancy_Pleura	0.00	0.00	5.69	0.94	2.50
Secondary Thrombocytopaenia	0.00	0.00	0.89	0.19	0.48
Secondary polycythaemia	0.00	0.00	1.64	0.32	0.78
Secondary pulmonary hypertension	0.00	0.00	1.29	0.27	0.83
Sick sinus syndrome	0.00	0.00	0.79	0.14	0.40
Sickle Cell Disease	0.00	0.00	0.98	0.29	1.07
Sjogren's Syndrome	0.00	0.00	1.48	0.31	0.68
Sleep apnoea	0.00	0.00	0.92	0.19	0.43
Spina bifida	0.00	0.00	0.48	0.11	0.44
Spinal stenosis	0.00	0.00	2.34	0.50	1.06
Spondylolisthesis	0.00	0.00	1.22	0.23	0.63
Spondylosis	0.00	0.00	1.01	0.21	0.57
Stable Angina	0.00	0.00	1.62	0.37	0.78

For peer review only

Subarachnoid haemorrhage	0.00	0.00	2.41	0.51	1.05
Substance Misuse	0.00	0.00	1.42	0.32	1.34
Supraventricular tachycardia	0.00	0.00	1.55	0.35	0.78
Thalassaemia	0.00	0.00	0.31	0.05	0.19
Thrombophilia	0.00	0.00	0.75	0.15	0.53
Tinnitus	0.00	0.00	0.85	0.17	0.43
Transient ischaemic attack	0.00	0.00	1.56	0.35	0.70
Trigeminal neuralgia	0.00	0.00	2.16	0.47	1.05
Tubulo-interstitial nephritis	0.00	0.00	2.70	0.50	1.23
Unstable Angina	0.00	0.00	1.17	0.23	0.58
Urinary Incontinence	0.00	0.00	0.87	0.18	0.38
Venous thromboembolic disease (Excl PE)	0.00	0.00	1.85	0.41	1.05
Ventricular tachycardia	0.00	0.00	1.64	0.32	0.75
Visual impairment and blindness	0.00	0.00	0.73	0.13	0.31
Vitiligo	0.00	0.00	0.73	0.14	0.32

For peer review only

Figure A1: Boxplots of observed and expected mean yearly codes at a GP practice level for QOF conditions in year 1 (A) and year 2 (B) and non-QOF conditions in year 1 (C) and year 2 (D) following diagnosis

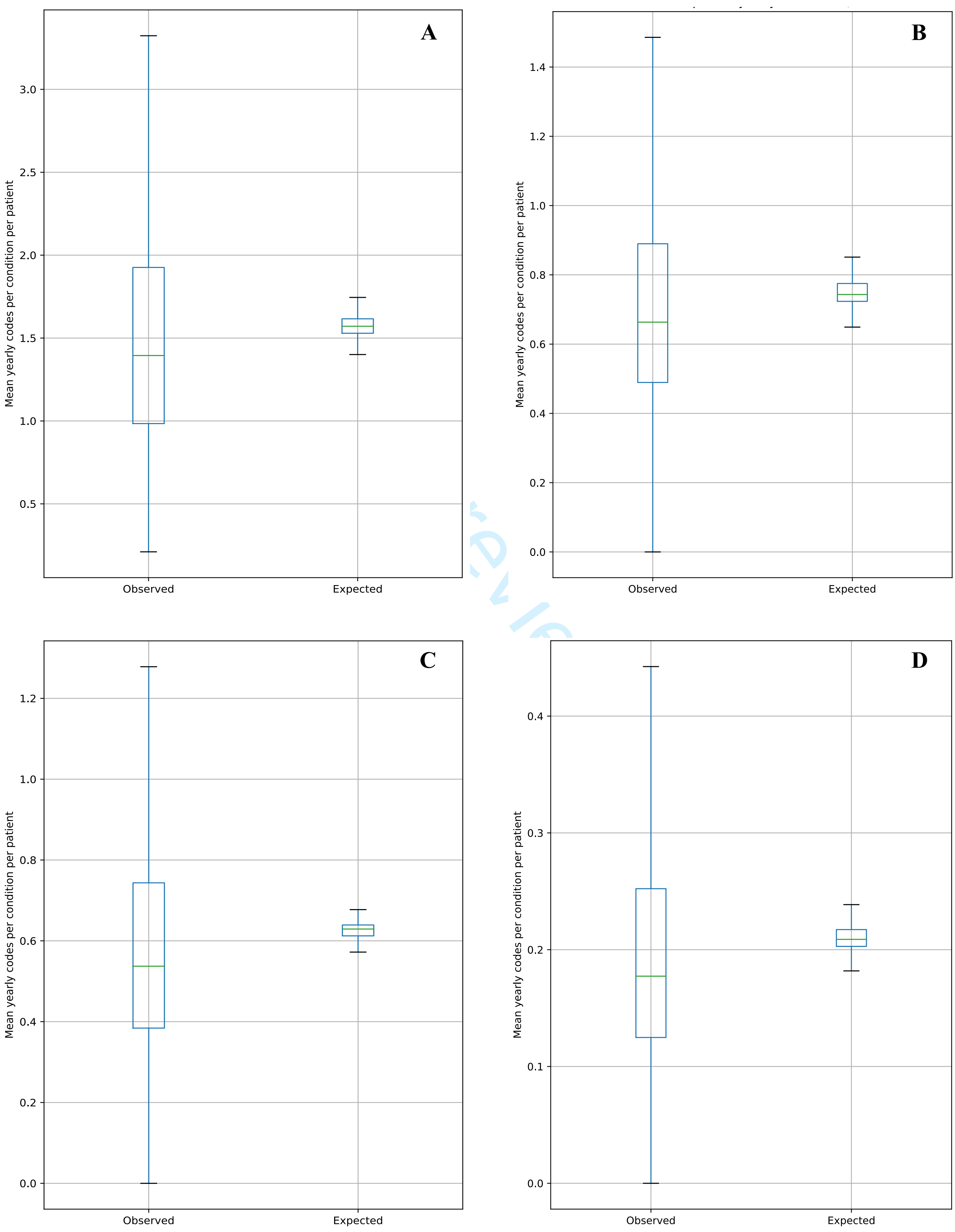

For peer review only - http://bmjopen.bmj.com/site/about/guidelines.xhtml

Figure A2: ratio of mean yearly codes in year 1 following diagnosis to subsequent years for QOF conditions

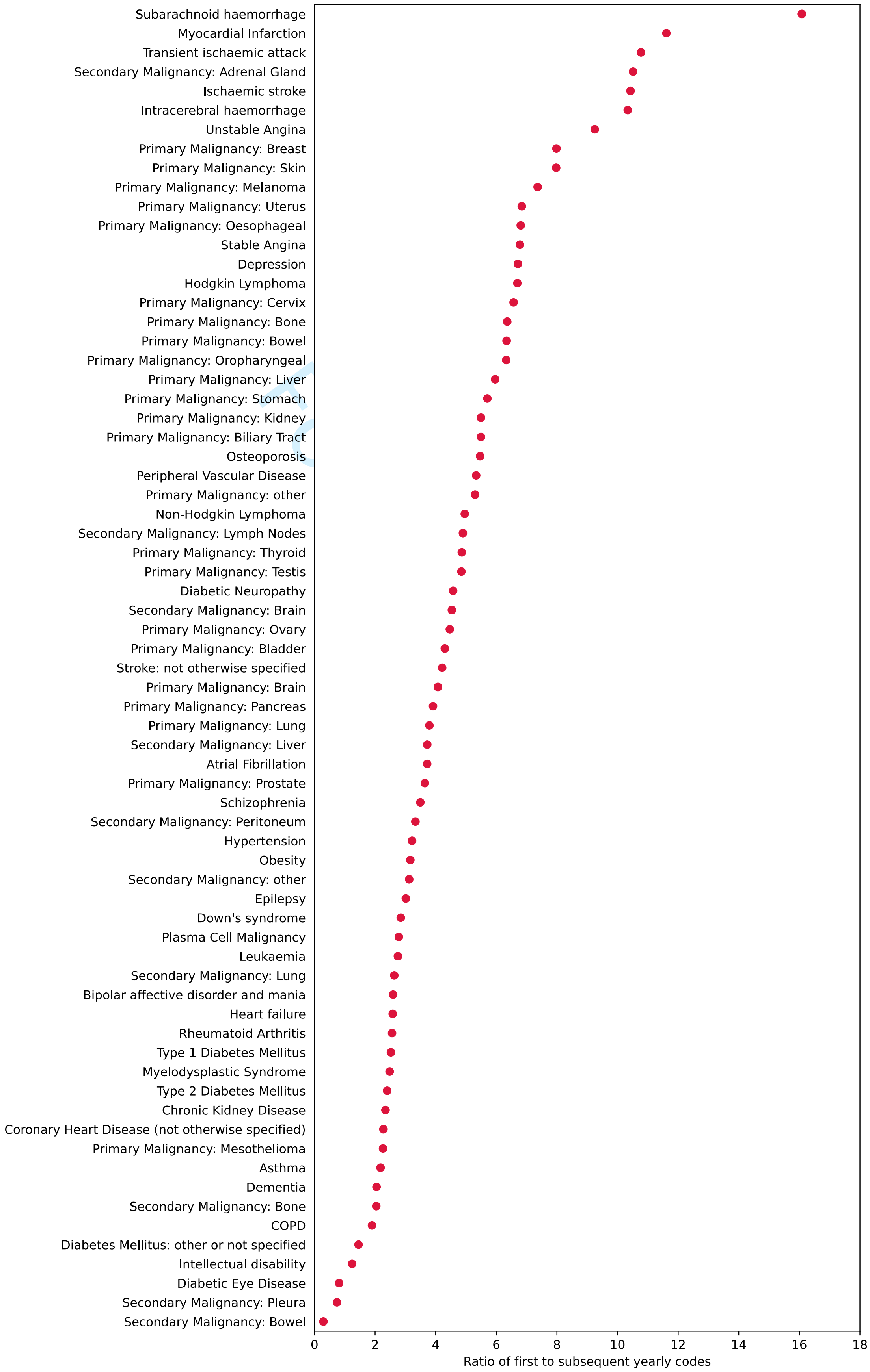

Figure A3: Ratio of mean yearly codes in year 1 following diagnosis to subsequent years for non-QOF conditions

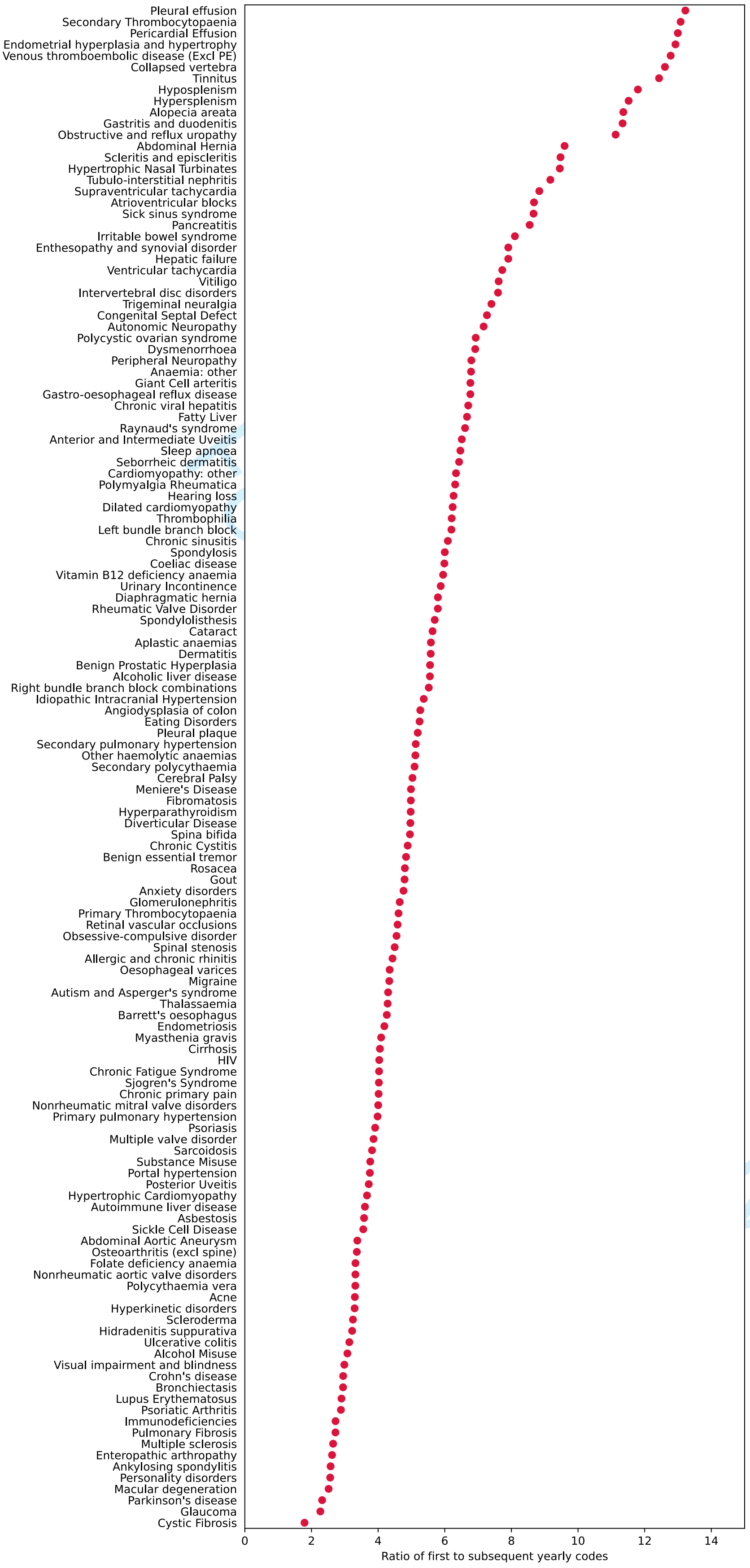

Figure A4: Boxplots of the distribution of mean yearly codes following diagnosis for newly diagnosed conditions by GP practice stratified by inclusion in QOF

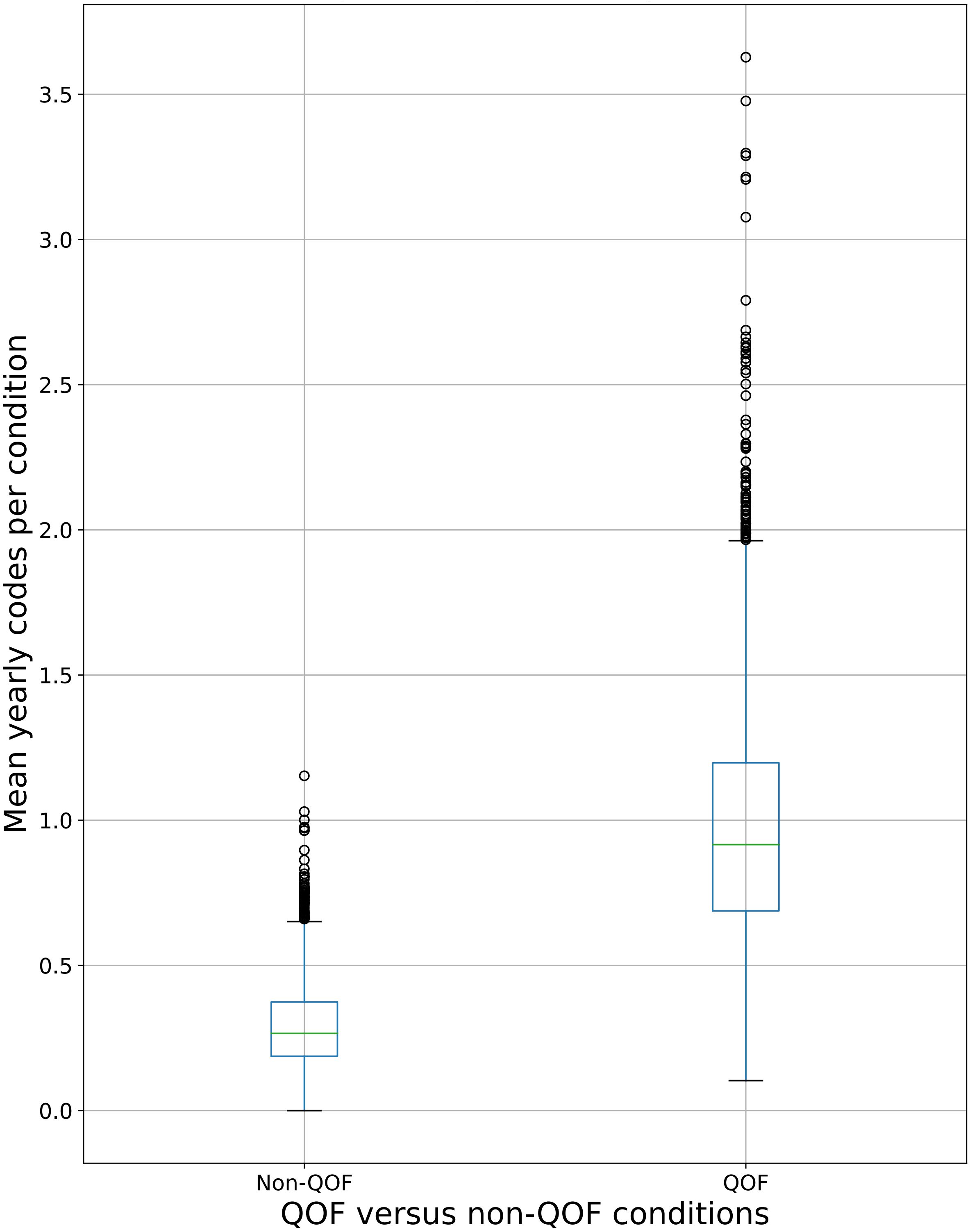

Figure A5: boxplots of mean yearly codes at a GP practice level by practice level Index of Multiple Deprivation decile (1 = most deprived, 10 = least deprived)

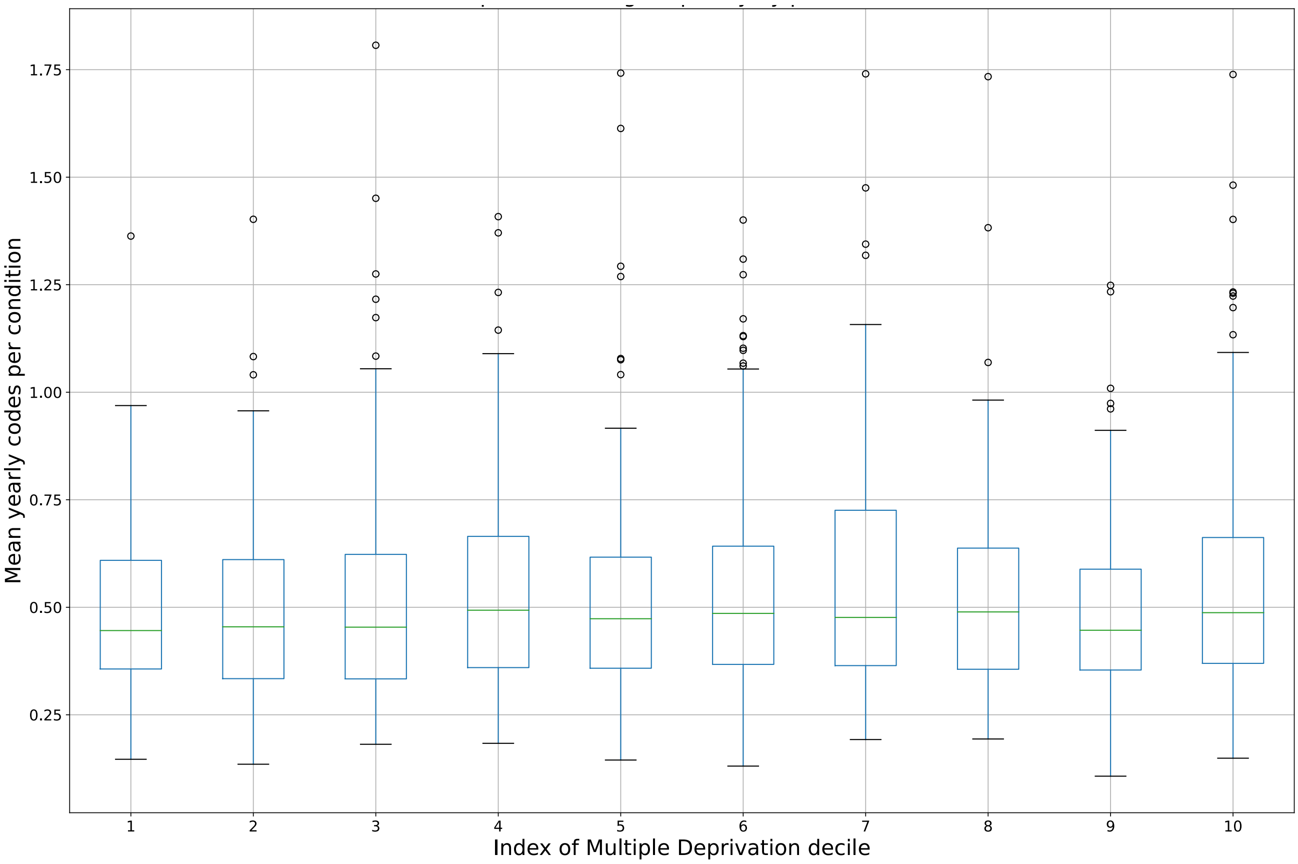

Footnote: combines QOF and non-QOF conditions

For peer review only - http://bmjopen.bmj.com/site/about/guidelines.xhtml

Table A5: Associations of rate of codes in year one following diagnosis for conditions included in QOF (N=1730485)

Variable	Primary analysis				Sensitivity analysis including consultation number			
			95% CI				95% CI	
	IRR	P-value	Lower	Upper	IRR	P-value	Lower	Upper
**Age category (years)**								
Under 40	1.33	0.00	1.32	1.34	1.30	0.00	1.29	1.31
40-49	1.15	0.00	1.14	1.15	1.14	0.00	1.13	1.15
50-59	1.08	0.00	1.07	1.08	1.07	0.00	1.07	1.08
60-69 (reference)	-	-	-	-	-	-	-	-
70-79	0.96	0.00	0.95	0.96	0.94	0.00	0.93	0.95
80 or more	0.91	0.00	0.90	0.92	0.88	0.00	0.87	0.88
**Sex**								
Female (reference)	-	-	-	-	-	-	-	-
Male	1.03	0.00	1.02	1.03	1.10	0.00	1.10	1.11
**Ethnicity category**								
White (reference)	-	-	-	-	-	-	-	-
South Asian	0.96	0.00	0.95	0.97	0.92	0.00	0.91	0.93
Black	0.94	0.00	0.93	0.95	0.94	0.00	0.93	0.95
Other	0.95	0.00	0.93	0.97	0.96	0.00	0.94	0.98
Mixed	0.98	0.03	0.95	1.00	0.97	0.00	0.95	0.99
Missing	0.98	0.00	0.97	0.99	1.01	0.00	1.00	1.02
**IMD decile**								
1 (most deprived)	-	-	-	-	-	-	-	-
2	1.01	0.19	1.00	1.01	1.00	0.95	0.99	1.01
3	1.02	0.00	1.01	1.03	1.01	0.08	1.00	1.02
4	1.02	0.00	1.01	1.03	1.01	0.01	1.00	1.02
5	1.02	0.00	1.01	1.03	1.01	0.06	1.00	1.02
6	1.03	0.00	1.02	1.04	1.01	0.02	1.00	1.02
7	1.04	0.00	1.03	1.05	1.02	0.00	1.01	1.03
8	1.04	0.00	1.03	1.05	1.01	0.01	1.00	1.02
9	1.05	0.00	1.04	1.06	1.02	0.00	1.01	1.03
10 (least deprived)	1.05	0.00	1.04	1.06	1.01	0.06	1.00	1.02
**Number of QOF diseases**								
0 (reference)	-	-	-	-	-	-	-	-
1	0.90	0.00	0.90	0.91	0.87	0.00	0.86	0.87
2	0.80	0.00	0.80	0.81	0.75	0.00	0.75	0.76
3	0.71	0.00	0.70	0.71	0.66	0.00	0.65	0.66
4 or more	0.63	0.00	0.62	0.63	0.56	0.00	0.55	0.56
**Number of non-QOF diseases**								
0 (reference)	-	-	-	-	-	-	-	-
1	1.16	0.00	1.16	1.17	1.08	0.00	1.07	1.08
2	1.13	0.00	1.12	1.14	1.02	0.00	1.01	1.02
3	1.12	0.00	1.11	1.12	0.97	0.00	0.96	0.98
4 or more	1.13	0.00	1.12	1.13	0.90	0.00	0.89	0.90
**Calendar year of diagnosis**								
2015 (reference)	-	-	-	-	-	-	-	-
2016	1.00	0.89	0.99	1.01	1.02	0.00	1.02	1.03
2017	1.00	0.34	1.00	1.01	1.05	0.00	1.04	1.05
2018	1.00	0.18	0.99	1.00	1.06	0.00	1.06	1.07
2019	0.95	0.00	0.94	0.96	1.04	0.00	1.04	1.05
**Average number of consultations in year 1**								
Less than 1 (reference)	-	-	-	-	-	-	-	-
1-2	-	-	-	-	1.62	0.00	1.60	1.63
3-4	-	-	-	-	2.21	0.00	2.19	2.23
5-9	-	-	-	-	2.87	0.00	2.84	2.89
10 or more	-	-	-	-	3.75	0.00	3.71	3.79

From negative binomial regression models, including practice-level fixed effects (not shown)

For peer review only

Table A6: Associations of rate of codes in year two following diagnosis for conditions included in QOF (N=1714684)

	Primary analysis				Sensitivity analysis including consultation number			
			95% CI				95% CI	
**Variable**	**IRR**	**P-value**	**Lower**	**Upper**	**IRR**	**P-value**	**Lower**	**Upper**
**Age category (years)**								
Under 40	0.87	0.00	0.86	0.87	0.86	0.00	0.86	0.87
40-49	1.01	0.03	1.00	1.02	1.01	0.22	1.00	1.01
50-59	1.03	0.00	1.02	1.04	1.03	0.00	1.02	1.04
60-69 (reference)	-	-	-	-	-	-	-	-
70-79	0.95	0.00	0.94	0.96	0.93	0.00	0.93	0.94
80 or more	0.89	0.00	0.88	0.90	0.86	0.00	0.85	0.87
**Sex**								
Female (reference)	-	-	-	-	-	-	-	-
Male	1.11	0.00	1.11	1.12	1.18	0.00	1.17	1.18
**Ethnicity category**								
White (reference)	-	-	-	-	-	-	-	-
South Asian	1.26	0.00	1.25	1.28	1.22	0.00	1.20	1.23
Black	1.17	0.00	1.16	1.19	1.17	0.00	1.15	1.19
Other	1.13	0.00	1.10	1.16	1.14	0.00	1.11	1.17
Mixed	1.12	0.00	1.08	1.15	1.11	0.00	1.07	1.14
Missing	0.89	0.00	0.88	0.90	0.93	0.00	0.92	0.93
**IMD decile**								
1 (most deprived)	-	-	-	-	-	-	-	-
2	1.02	0.00	1.01	1.04	1.02	0.00	1.01	1.03
3	1.03	0.00	1.02	1.05	1.03	0.00	1.02	1.04
4	1.05	0.00	1.03	1.06	1.04	0.00	1.03	1.05
5	1.05	0.00	1.04	1.07	1.04	0.00	1.03	1.06
6	1.06	0.00	1.05	1.07	1.05	0.00	1.04	1.07
7	1.08	0.00	1.06	1.09	1.06	0.00	1.05	1.08
8	1.09	0.00	1.07	1.10	1.07	0.00	1.06	1.08
9	1.11	0.00	1.10	1.13	1.09	0.00	1.08	1.11
10 (least deprived)	1.14	0.00	1.12	1.15	1.11	0.00	1.09	1.12
**Number of QOF diseases**								
0 (reference)	-	-	-	-	-	-	-	-
1	1.05	0.00	1.04	1.06	1.00	0.79	0.99	1.01
2	1.07	0.00	1.06	1.08	0.99	0.05	0.98	1.00
3	0.99	0.15	0.98	1.00	0.91	0.00	0.90	0.92
4 or more	0.87	0.00	0.86	0.88	0.77	0.00	0.76	0.78
**Number of non-QOF diseases**								
0 (reference)	-	-	-	-	-	-	-	-
1	1.05	0.00	1.04	1.06	0.99	0.11	0.98	1.00
2	1.04	0.00	1.03	1.05	0.96	0.00	0.95	0.97
3	1.04	0.00	1.03	1.05	0.93	0.00	0.92	0.94
4 or more	1.05	0.00	1.04	1.06	0.88	0.00	0.87	0.89
**Calendar year of diagnosis**								
2015 (reference)	-	-	-	-	-	-	-	-
2016	1.00	0.45	0.99	1.01	1.02	0.00	1.01	1.03
2017	0.99	0.00	0.98	0.99	1.02	0.00	1.01	1.03
2018	0.91	0.00	0.90	0.92	0.96	0.00	0.95	0.97
2019	0.79	0.00	0.79	0.80	0.86	0.00	0.86	0.87
**Average number of consultations in year 1**								
Less than 1 (reference)					-	-	-	-
2					1.53	0.00	1.52	1.55
3-4					1.87	0.00	1.85	1.89
5-9					2.17	0.00	2.15	2.20
10 or more					2.59	0.00	2.57	2.62

From negative binomial regression models, including practice-level fixed effects (not shown)

For peer review only - http://bmjopen.bmj.com/site/about/guidelines.xhtml

Table A7: Associations of rate of codes in year one following diagnosis for conditions not included in QOF (N=3617348)

	Primary analysis				Sensitivity analysis including consultation number			
			95% CI				95% CI	
**Variable**	**IRR**	**P-value**	**Lower**	**Upper**	**IRR**	**P-value**	**Lower**	**Upper**
**Age category (years)**								
Under 40	1.10	0.00	1.10	1.11	1.09	0.00	1.08	1.10
40-49	1.01	0.00	1.00	1.02	1.02	0.00	1.01	1.03
50-59	0.98	0.00	0.98	0.99	0.99	0.09	0.99	1.00
60-69 (reference)	-	-	-	-	-	-	-	-
70-79	1.05	0.00	1.05	1.06	1.03	0.00	1.02	1.03
80 or more	1.02	0.00	1.02	1.03	0.98	0.00	0.97	0.99
**Sex**								
Female (reference)	-	-	-	-	-	-	-	-
Male	1.00	0.03	0.99	1.00	1.13	0.00	1.12	1.13
**Ethnicity category**								
White (reference)	-	-	-	-	-	-	-	-
South Asian	0.95	0.00	0.94	0.96	0.89	0.00	0.88	0.90
Black	0.89	0.00	0.88	0.90	0.86	0.00	0.85	0.87
Other	0.90	0.00	0.88	0.91	0.89	0.00	0.88	0.91
Mixed	0.95	0.00	0.93	0.97	0.92	0.00	0.91	0.94
Missing	0.99	0.14	0.99	1.00	1.06	0.00	1.05	1.06
**IMD decile**								
1 (most deprived)	-	-	-	-	-	-	-	-
2	1.00	0.86	0.99	1.01	0.99	0.06	0.98	1.00
3	1.01	0.01	1.00	1.02	1.00	0.82	0.99	1.01
4	1.02	0.00	1.01	1.03	1.00	0.42	0.99	1.01
5	1.02	0.00	1.01	1.03	1.00	0.86	0.99	1.01
6	1.03	0.00	1.02	1.04	0.99	0.26	0.99	1.00
7	1.03	0.00	1.02	1.04	0.99	0.08	0.98	1.00
8	1.04	0.00	1.03	1.06	0.99	0.15	0.98	1.00
9	1.06	0.00	1.05	1.07	0.99	0.19	0.98	1.00
10 (least deprived)	1.06	0.00	1.05	1.07	0.98	0.00	0.97	0.99
**Number of QOF diseases**								
0 (reference)	-	-	-	-	-	-	-	-
1	1.16	0.00	1.15	1.16	1.02	0.00	1.02	1.03
2	1.09	0.00	1.08	1.09	0.94	0.00	0.93	0.94
3	1.06	0.00	1.05	1.07	0.90	0.00	0.89	0.91
4 or more	1.04	0.00	1.03	1.04	0.85	0.00	0.84	0.85
**Number of non-QOF diseases**								
0 (reference)	-	-	-	-	-	-	-	-
1	1.02	0.00	1.01	1.02	0.93	0.00	0.92	0.94
2	1.02	0.00	1.02	1.03	0.87	0.00	0.87	0.88
3	1.04	0.00	1.03	1.05	0.83	0.00	0.82	0.84
4 or more	1.06	0.00	1.06	1.07	0.74	0.00	0.74	0.75
**Calendar year of diagnosis**								
2015 (reference)	-	-	-	-	-	-	-	-
2016	1.00	0.55	0.99	1.00	1.03	0.00	1.02	1.03
2017	0.99	0.00	0.99	1.00	1.05	0.00	1.04	1.05
2018	0.99	0.00	0.98	0.99	1.07	0.00	1.06	1.07
2019	0.94	0.00	0.94	0.95	1.06	0.00	1.06	1.07
**Average number of consultations in year 1**								
Less than 1 (reference)					-	-	-	-
1-2					2.38	0.00	2.36	2.40
3-4					3.49	0.00	3.45	3.52
5-9					4.67	0.00	4.62	4.71
10 or more					6.37	0.00	6.31	6.44

From negative binomial regression models, including practice-level fixed effects (not shown)

For peer review only - http://bmjopen.bmj.com/site/about/guidelines.xhtml

For peer review only

Table A8: Associations of rate of codes in year two following diagnosis for conditions not included in QOF (N=3593019)

	Primary analysis				Sensitivity analysis including consultation number			
			95% CI				95% CI	
**Variable**	**IRR**	**P-value**	**Lower**	**Upper**	**IRR**	**P-value**	**Lower**	**Upper**
**Age category (years)**								
Under 40	1.27	0.00	1.26	1.28	1.26	0.00	1.25	1.28
40-49	1.03	0.00	1.02	1.04	1.03	0.00	1.02	1.04
50-59	0.98	0.00	0.97	0.99	0.99	0.10	0.98	1.00
60-69 (reference)	-	-	-	-	-	-	-	-
70-79	1.06	0.00	1.05	1.07	1.03	0.00	1.02	1.04
80 or more	1.06	0.00	1.05	1.08	1.01	0.18	1.00	1.02
**Sex**								
Female (reference)	-	-	-	-	-	-	-	-
Male	0.93	0.00	0.93	0.94	1.08	0.00	1.07	1.09
**Ethnicity category**								
White (reference)	-	-	-	-	-	-	-	-
South Asian	0.99	0.17	0.97	1.00	0.92	0.00	0.91	0.94
Black	0.94	0.00	0.92	0.95	0.91	0.00	0.89	0.92
Other	0.88	0.00	0.86	0.91	0.89	0.00	0.86	0.92
Mixed	0.94	0.00	0.91	0.98	0.92	0.00	0.89	0.95
Missing	0.96	0.00	0.95	0.97	1.05	0.00	1.03	1.06
**IMD decile**								
1 (most deprived)	-	-	-	-	-	-	-	-
2	1.01	0.10	1.00	1.03	1.00	0.79	0.99	1.02
3	1.03	0.00	1.02	1.05	1.02	0.00	1.01	1.04
4	1.04	0.00	1.02	1.05	1.02	0.01	1.01	1.04
5	1.05	0.00	1.04	1.07	1.03	0.00	1.01	1.04
6	1.06	0.00	1.04	1.08	1.03	0.00	1.01	1.04
7	1.07	0.00	1.06	1.09	1.03	0.00	1.01	1.05
8	1.10	0.00	1.08	1.11	1.04	0.00	1.03	1.06
9	1.13	0.00	1.11	1.14	1.06	0.00	1.04	1.08
10 (least deprived)	1.14	0.00	1.12	1.16	1.06	0.00	1.04	1.08
**Number of QOF diseases**								
0 (reference)	-	-	-	-	-	-	-	-
1	1.19	0.00	1.18	1.21	1.05	0.00	1.04	1.06
2	1.15	0.00	1.14	1.16	0.98	0.00	0.97	0.99
3	1.13	0.00	1.12	1.15	0.95	0.00	0.94	0.96
4 or more	1.16	0.00	1.14	1.17	0.93	0.00	0.92	0.94
**Number of non-QOF diseases**								
0 (reference)	-	-	-	-	-	-	-	-
1	1.04	0.00	1.03	1.06	0.94	0.00	0.93	0.95
2	1.09	0.00	1.08	1.11	0.90	0.00	0.89	0.91
3	1.13	0.00	1.11	1.14	0.86	0.00	0.85	0.87
4 or more	1.21	0.00	1.20	1.23	0.80	0.00	0.79	0.81
**Calendar year of diagnosis**								
2015 (reference)	-	-	-	-	-	-	-	-
2016	1.00	0.56	0.99	1.01	1.03	0.00	1.02	1.04
2017	1.00	0.43	0.99	1.01	1.06	0.00	1.05	1.07
2018	0.91	0.00	0.90	0.92	1.01	0.01	1.00	1.02
2019	0.79	0.00	0.79	0.80	0.93	0.00	0.92	0.94
**Average number of consultations in year 1**								
Less than 1 (reference)					-	-	-	-
1-2					2.76	0.00	2.72	2.81
3-4					4.06	0.00	4.00	4.12
5-9					5.40	0.00	5.32	5.48
10 or more					7.35	0.00	7.24	7.47

From negative binomial regression models, including practice-level fixed effects (not shown)

For peer review only - http://bmjopen.bmj.com/site/about/guidelines.xhtml

References

1. Head, A. *et al.* Inequalities in incident and prevalent multimorbidity in England, 2004–19: a population-based, descriptive study. *The Lancet Healthy Longevity* **2**, e489–e497 (2021).
2. Kuan, V. *et al.* A chronological map of 308 physical and mental health conditions from 4 million individuals in the English National Health Service. *The Lancet Digital Health* **1**, e63–e77 (2019).
3. Bisquera, A. *et al.* Inequalities in developing multimorbidity over time: A population-based cohort study from an urban, multi-ethnic borough in the United Kingdom. *Lancet Reg Health Eur* **12**, 100247 (2021).
4. Ashworth, M. *et al.* Journey to multimorbidity: longitudinal analysis exploring cardiovascular risk factors and sociodemographic determinants in an urban setting. *BMJ Open* **9**, (2019).
5. NHS Health and Social Care Information Centre. National Quality and Outcomes Framework Statistics for England 2004/05. https://files.digital.nhs.uk/publicationimport/pub01xxx/pub01946/qof-eng-04-05-rep.pdf.
6. Health & Social Care Information Centre. Quality and Outcomes Framework – Prevalence, Achievements and Exceptions Report. England, 2013-14. https://files.digital.nhs.uk/publicationimport/pub15xxx/pub15751/qof-1314-report-v1.1.pdf.
7. Health & Social Care Information Centre. Quality and Outcomes Framework – Prevalence, Achievements and Exceptions Report, England, 2014-15. https://files.digital.nhs.uk/publicationimport/pub18xxx/pub18887/qof-1415-report%20v1.1.pdf (2015).

For peer review only

For peer review only - http://bmjopen.bmj.com/site/about/guidelines.xhtml

The RECORD statement – checklist of items, extended from the STROBE statement, that should be reported in observational studies using routinely collected health data.

	Item No.	STROBE items	Location in manuscript where items are reported	RECORD items	Location in manuscript where items are reported
**Title and abstract**					
	1	(a) Indicate the study's design with a commonly used term in the title or the abstract (b) Provide in the abstract an informative and balanced summary of what was done and what was found	p1-3	RECORD 1.1: The type of data used should be specified in the title or abstract. When possible, the name of the databases used should be included.<br><br>RECORD 1.2: If applicable, the geographic region and timeframe within which the study took place should be reported in the title or abstract.<br><br>RECORD 1.3: If linkage between databases was conducted for the study, this should be clearly stated in the title or abstract.	p1<br><br>p2<br><br>N/A
**Introduction**					
Background rationale	2	Explain the scientific background and rationale for the investigation being reported	p4-5		
Objectives	3	State specific objectives, including any prespecified hypotheses	p5		
**Methods**					
Study Design	4	Present key elements of study design early in the paper	p5		
Setting	5	Describe the setting, locations, and relevant dates, including periods of recruitment, exposure, follow-up, and data collection	p5		

For peer review only

Participants	6	*(a) Cohort study* - Give the eligibility criteria, and the sources and methods of selection of participants. Describe methods of follow-up<br>*Case-control study* - Give the eligibility criteria, and the sources and methods of case ascertainment and control selection. Give the rationale for the choice of cases and controls<br>*Cross-sectional study* - Give the eligibility criteria, and the sources and methods of selection of participants<br><br>*(b) Cohort study* - For matched studies, give matching criteria and number of exposed and unexposed<br>*Case-control study* - For matched studies, give matching criteria and the number of controls per case	p5 and appendix p2	RECORD 6.1: The methods of study population selection (such as codes or algorithms used to identify subjects) should be listed in detail. If this is not possible, an explanation should be provided.<br><br>RECORD 6.2: Any validation studies of the codes or algorithms used to select the population should be referenced. If validation was conducted for this study and not published elsewhere, detailed methods and results should be provided.<br><br>RECORD 6.3: If the study involved linkage of databases, consider use of a flow diagram or other graphical display to demonstrate the data linkage process, including the number of individuals with linked data at each stage.	p5
Variables	7	Clearly define all outcomes, exposures, predictors, potential confounders, and effect modifiers. Give diagnostic criteria, if applicable.	p5 and appendix p2-3	RECORD 7.1: A complete list of codes and algorithms used to classify exposures, outcomes, confounders, and effect modifiers should be provided. If these cannot be reported, an explanation should be provided.	
Data sources/ measurement	8	For each variable of interest, give sources of data and details of methods of assessment (measurement).<br>Describe comparability of assessment methods if there is more than one group	p5 and appendix p2-3		

For peer review only

Bias	9	Describe any efforts to address potential sources of bias	p6-7		
Study size	10	Explain how the study size was arrived at	p8		
Quantitative variables	11	Explain how quantitative variables were handled in the analyses. If applicable, describe which groupings were chosen, and why	P5-6		
Statistical methods	12	(a) Describe all statistical methods, including those used to control for confounding<br>(b) Describe any methods used to examine subgroups and interactions<br>(c) Explain how missing data were addressed<br>(d) *Cohort study* - If applicable, explain how loss to follow-up was addressed<br>*Case-control study* - If applicable, explain how matching of cases and controls was addressed<br>*Cross-sectional study* - If applicable, describe analytical methods taking account of sampling strategy<br>(e) Describe any sensitivity analyses	p6-7		
Data access and cleaning methods		..		RECORD 12.1: Authors should describe the extent to which the investigators had access to the database population used to create the study population.	p5

For peer review only

				RECORD 12.2: Authors should provide information on the data cleaning methods used in the study.	
Linkage		..		RECORD 12.3: State whether the study included person-level, institutional-level, or other data linkage across two or more databases. The methods of linkage and methods of linkage quality evaluation should be provided.	N/A
**Results**					
Participants	13	(a) Report the numbers of individuals at each stage of the study (*e.g.*, numbers potentially eligible, examined for eligibility, confirmed eligible, included in the study, completing follow-up, and analysed)<br>(b) Give reasons for non-participation at each stage.<br>(c) Consider use of a flow diagram	p8	RECORD 13.1: Describe in detail the selection of the persons included in the study (*i.e.,* study population selection) including filtering based on data quality, data availability and linkage. The selection of included persons can be described in the text and/or by means of the study flow diagram.	p8
Descriptive data	14	(a) Give characteristics of study participants (*e.g.*, demographic, clinical, social) and information on exposures and potential confounders<br>(b) Indicate the number of participants with missing data for each variable of interest<br>(c) *Cohort study* - summarise follow-up time (*e.g.*, average and total amount)	p8, Table 1		
Outcome data	15	*Cohort study* - Report numbers of outcome events or summary measures over time<br>*Case-control study* - Report numbers in each exposure	p9-10		

		category, or summary measures of exposure<br>*Cross-sectional study* - Report numbers of outcome events or summary measures			
Main results	16	(a) Give unadjusted estimates and, if applicable, confounder-adjusted estimates and their precision (e.g., 95% confidence interval). Make clear which confounders were adjusted for and why they were included<br>(b) Report category boundaries when continuous variables were categorized<br>(c) If relevant, consider translating estimates of relative risk into absolute risk for a meaningful time period	p9-14, Figures 1-3		
Other analyses	17	Report other analyses done—e.g., analyses of subgroups and interactions, and sensitivity analyses	p11		
**Discussion**					
Key results	18	Summarise key results with reference to study objectives	p15		
Limitations	19	Discuss limitations of the study, taking into account sources of potential bias or imprecision. Discuss both direction and magnitude of any potential bias	p17	RECORD 19.1: Discuss the implications of using data that were not created or collected to answer the specific research question(s). Include discussion of misclassification bias, unmeasured confounding, missing data, and changing eligibility over time, as they pertain to the study being reported.	p17
Interpretation	20	Give a cautious overall interpretation of results considering objectives,	p15, p17-18		

For peer review only

		limitations, multiplicity of analyses, results from similar studies, and other relevant evidence			
Generalisability	21	Discuss the generalisability (external validity) of the study results	p17		
**Other Information**					
Funding	22	Give the source of funding and the role of the funders for the present study and, if applicable, for the original study on which the present article is based	p18		
Accessibility of protocol, raw data, and programming code		..		RECORD 22.1: Authors should provide information on how to access any supplemental information such as the study protocol, raw data, or programming code.	p18

*Reference: Benchimol EI, Smeeth L, Guttmann A, Harron K, Moher D, Petersen I, Sørensen HT, von Elm E, Langan SM, the RECORD Working Committee. The REporting of studies Conducted using Observational Routinely-collected health Data (RECORD) Statement. *PLoS Medicine* 2015; in press.

*Checklist is protected under Creative Commons Attribution (CC BY) license.

For peer review only

BMJ Open

Identifying potential biases in code sequences in primary care electronic healthcare records: a retrospective cohort study of the determinants of code frequency

Journal:	*BMJ Open*
Manuscript ID	bmjopen-2023-072884.R2
Article Type:	Original research
Date Submitted by the Author:	31-Aug-2023
Complete List of Authors:	Beaney, Thomas; Imperial College London, Department of Primary Care and Public Health; Imperial College London, Department of Mathematics<br>Clarke, Jonathan; Imperial College of Science Technology and Medicine, Institute of Global Health Innovation<br>Salman, David; Imperial College London Department of Primary Care and Public Health; Imperial College London Faculty of Medicine, MSk lab<br>Woodcock, Thomas; Imperial College London, Department of Primary Care and Public Health<br>Majeed, Azeem; Imperial College London, Department of Primary Care and Public Health<br>Barahona, Mauricio; Imperial College London, Centre for Mathematics of Precision Healthcare; Imperial College London, Department of Mathematics<br>Aylin, Paul; Imperial College London, Department of Primary Care and Public Health
**Primary Subject Heading**:	Health informatics
Secondary Subject Heading:	General practice / Family practice, Health informatics, Health services research, Epidemiology
Keywords:	EPIDEMIOLOGY, Primary Health Care, Health informatics < BIOTECHNOLOGY & BIOINFORMATICS, STATISTICS & RESEARCH METHODS

SCHOLARONE™
Manuscripts

I, the Submitting Author has the right to grant and does grant on behalf of all authors of the Work (as defined in the below author licence), an exclusive licence and/or a non-exclusive licence for contributions from authors who are: i) UK Crown employees; ii) where BMJ has agreed a CC-BY licence shall apply, and/or iii) in accordance with the terms applicable for US Federal Government officers or employees acting as part of their official duties; on a worldwide, perpetual, irrevocable, royalty-free basis to BMJ Publishing Group Ltd ("BMJ") its licensees and where the relevant Journal is co-owned by BMJ to the co-owners of the Journal, to publish the Work in this journal and any other BMJ products and to exploit all rights, as set out in our licence.

The Submitting Author accepts and understands that any supply made under these terms is made by BMJ to the Submitting Author unless you are acting as an employee on behalf of your employer or a postgraduate student of an affiliated institution which is paying any applicable article publishing charge ("APC") for Open Access articles. Where the Submitting Author wishes to make the Work available on an Open Access basis (and intends to pay the relevant APC), the terms of reuse of such Open Access shall be governed by a Creative Commons licence – details of these licences and which Creative Commons licence will apply to this Work are set out in our licence referred to above.

Other than as permitted in any relevant BMJ Author's Self Archiving Policies, I confirm this Work has not been accepted for publication elsewhere, is not being considered for publication elsewhere and does not duplicate material already published. I confirm all authors consent to publication of this Work and authorise the granting of this licence.

For peer review only

Identifying potential biases in code sequences in primary care electronic healthcare records: a retrospective cohort study of the determinants of code frequency

Beaney T[1,2] (0000-0001-9709-7264), Clarke J[2], Salman D[1,3], Woodcock T[1], Majeed A[1], Barahona M[2], Aylin P[1]

1. Department of Primary Care and Public Health, Imperial College London, London, W6 8RP, United Kingdom
2. Centre for Mathematics of Precision Healthcare, Department of Mathematics, Imperial College London, London, SW7 2AZ, United Kingdom
3. MSk Lab, Department of Surgery and Cancer, Faculty of Medicine, Imperial College London, London, UK

Corresponding Author:
Dr Thomas Beaney
Department of Primary Care and Public Health, Imperial College London, London, W6 8RP, United Kingdom
Email: thomas.beaney@imperial.ac.uk

For peer review only

Abstract

Objectives

To determine whether the frequency of diagnostic codes for long-term conditions (LTCs) in primary care electronic health records (EHRs) is associated with i) disease coding incentives, ii) GP practice, iii) patient socio-demographic characteristics and iv) calendar year of diagnosis.

Design

Retrospective cohort study.

Setting

General practices in England from 2015 to 2022 contributing to the Clinical Practice Research Datalink Aurum dataset.

Participants

All patients registered to a GP with at least one incident LTC diagnosed between 01/01/2015 and 31/12/2019.

Primary and secondary outcome measures

The number of diagnostic codes for an LTC in i) the first and ii) the second year following diagnosis, stratified by inclusion in the Quality and Outcomes Framework (QOF) financial incentive programme.

Results

3,113,724 patients were included, with 7,723,365 incident LTCs. Conditions included in QOF had higher rates of annual coding than conditions not included in QOF (1.03 vs 0.32 per year, $p<0.0001$). There was significant variation in code frequency by GP practice which was not explained by patient socio-demographics. We found significant associations with patient socio-demographics, with a trend towards lower coding rates in people living in areas of higher deprivation for both QOF and non-QOF conditions. Code frequency was lower for conditions with follow-up time in 2020, associated with the onset of the COVID-19 pandemic.

Conclusions

The frequency of diagnostic codes for newly diagnosed LTCs is influenced by factors including patient socio-demographics, disease inclusion in QOF, GP practice, and the impact of the COVID-19 pandemic. Natural language processing or other methods using temporally-ordered code sequences should account for these factors to minimise potential bias.

For peer review only

Strengths and limitations

- This study used a large and representative sample of patients in England, including 3 million patients with one of 208 incident diseases developed over 5 years.
- We focussed on incident diseases during the study period to minimise bias from historic or inactive diseases.
- We found significant differences in the frequency of codes according to patient socio-demographics, GP practice, and disease inclusion in QOF, but could not determine whether these differences reflect differences in healthcare utilisation versus coding quality.

For peer review only

Background

Methods developed in natural language processing (NLP) are increasingly being employed to analyse routinely collected healthcare data, such as data recorded in the Electronic Healthcare Record (EHR).(1–6) These methods show promise across a range of tasks, including prediction of health outcomes,(1,5,6) and clustering of co-occurring diseases.(2) Although developed for the analysis of language data, such as the free text data found in 'unstructured' medical records, NLP methods can also be applied to coded or 'structured' data found in many EHR databases. Using structured data, disease codes arranged in a temporal sequence in a patient's EHR history can be considered analogous to words in a sentence or document.(5)

In primary care EHRs, diagnostic codes may be entered either during a consultation, or entered outside, such as on receiving communication of a new diagnosis from hospital, or retrospectively coding a pre-existing diagnosis. In predictive modelling scenarios, such as those used in NLP, codes from both sources are relevant to understanding a patient's health status. However, a potential problem facing sequence-based methods is the extent to which repeated codes are an objective marker of a patient's health status and a presentation with a particular condition or relate to the quality of coding in the EHR.(7) Although previous studies of EHR data in England have shown the prevalence of many long-term conditions (LTCs) to be comparable to those from national statistics, these are often calculated based on the presence of a single diagnostic code.(8) Whether repeated codes for LTCs are entered in the EHR subsequently may be determined by a range of factors, including patient characteristics, clinician incentives and organisational policies, which may vary over time.(9,10)

Unlike in secondary care, where diagnostic coding directly impacts on payments, General Practice in England receives funding primarily through capitated payments based on the size of the registered population(11) with no direct financial incentive for code entry during a consultation. However, around 10% of funding comes from the Quality and Outcomes Framework (QOF), introduced in the National Health Service for GPs in 2004.(11) QOF provides financial incentives for meeting targets for a set of chronic conditions, including regular clinical reviews, and has been credited with improvements to data collection for these conditions.(12–14) Codes for conditions in QOF may occur more frequently than for

For peer review only

conditions not included in the incentive scheme, which could affect sequence-based methods using recurrent codes.

Analytical methods using temporally-ordered code sequences in the EHR may therefore be susceptible to biases in the frequency of codes entered following diagnosis, potentially resulting in models representing some people better than others. Awareness of the factors influencing the frequency of codes may help researchers using NLP methods by informing adjustment or sensitivity analyses. This study aims firstly to compare the frequency of repeated codes after diagnosis for a common set of LTCs. Secondly, we aim to determine whether the frequency of codes varies according to i) disease inclusion in QOF, ii) GP practice, iii) patient socio-demographic characteristics, and iv) calendar year of diagnosis.

Methods

Data source

This study used data from the Clinical Practice Research Datalink (CPRD) Aurum dataset, which contains primary care data for GP practices using EMIS Web software.(15) We included all patients assessed by CPRD to be research acceptable (meeting certain quality criteria such as a valid registration date and date of birth(16)) with a continuous period of registration at a GP practice in CPRD between 1st January 2014 and 31st December 2020 (i.e. without having deregistered in this period).(17) Patients were eligible if aged 18 years or over with at least one incident disease diagnosed between 1st January 2015 and 31st December 2019, allowing for at least one full year of practice registration before disease diagnosis and at least one full year of follow-up for each condition. Demographic data included age, sex, ethnicity and Index of Multiple Deprivation (IMD) of the area in which the patient resided, grouped into deciles where 1 is the most deprived and 10 the least deprived.(18) Ethnicity is recorded as one of five categories, with recording in CPRD found previously to have high concordance with national estimates.(19) We focussed on incident diseases to reduce the potential for confounding from historic conditions, some of which may no longer be active. Patients were followed up until the earliest of death, de-registration or the date of latest data extraction from their GP practice. Further information on the cohort structure is given in the appendix (p2).

For peer review only

Disease definitions

Diagnostic codes were extracted from the CPRD 'Observation' table and codes recorded during or outside of consultations were included. The date that the event occurred ('obsdate') was used, in preference to the date the code was entered. We included a total of 208 LTCs. These were defined based on a set of disease codes from Head *et al* (2021), who selected 211 chronic conditions from 308 acute and chronic disease phenotypes developed for the CALIBER study.(20,21) We reviewed codes and made changes to the code-lists for diabetes and added a new condition of 'chronic primary pain' (see appendix p2-3). In CALIBER, conditions related to raised cholesterol or triglycerides are based only on laboratory results, rather than diagnostic disease codes. We excluded these conditions given that laboratory measurements may have different characteristics of coding frequency. Likewise, for obesity and Chronic Kidney Disease, we used the diagnostic codes included in the code lists, but did not include BMI and eGFR measurements. We considered a single code as diagnostic for each condition and defined the diagnosis date for each condition as the date of the earliest code for that condition. Diseases were stratified according to whether they appeared in QOF by two primary care clinicians, TB and DS (see appendix p2-3).

Statistical analysis

<u>Descriptive statistics</u>

For each disease newly diagnosed during the study period, we calculated the yearly number of subsequent codes (excluding the first code representing diagnosis) during follow-up:

$$y_i = \frac{\sum_{j=1}^{N} c_{i,j}}{\sum_{j=1}^{N} f_{i,j}}$$

where y_i is the yearly number of codes following diagnosis for condition i, $c_{i,j}$ is the count of codes for condition i in patient j, and $f_{i,j}$ is the number of years of follow-up for condition i in patient j. T-tests were used to compare the mean yearly number of codes for QOF versus non-QOF conditions.

To examine variation in disease coding frequency by GP practice, we calculated, for each practice k, the mean number of codes per year for newly diagnosed diseases, p_k:

For peer review only

$$p_k = \frac{\sum_{j=1}^{N}\sum_{i=1}^{M} c_{i,j,k}}{\sum_{j=1}^{N}\sum_{i=1}^{M} f_{i,j,k}}$$

where $c_{i,j,k}$ is the count of codes for condition *i* in patient *j* in practice *k,* and $f_{i,j,k}$ is the number of years of follow-up for condition *i* in patient *j* in practice *k.* We then calculated the Pearson correlation coefficient between the mean number of codes per year in each practice for QOF versus non-QOF conditions. We also compared the mean number of yearly codes in each practice stratified by the 2019 IMD decile of the GP practice. For conditions with at least two years of follow-up after the date of diagnosis, we calculated the ratio of the number of codes in the first year of diagnosis to the number of codes in subsequent years.

Regression analyses

Data were formatted as panel data with patients measured over multiple calendar years (appendix Table A1). We used mixed effects negative binomial regression to analyse the association between code frequency of newly diagnosed conditions in i) the first year following diagnosis and ii) the second year following diagnosis, with patient factors and calendar year of diagnosis. We separated the outcome variable (code frequency) into first and second year after diagnosis due to preliminary analyses indicating significant differences over time. We also stratified the regression analyses by QOF inclusion, given our hypothesis that it may be an effect modifier of the relationships. To account for cases where a patient may have more than one QOF or non-QOF condition diagnosed within the same year, we averaged the code frequency for all newly diagnosed QOF or non-QOF conditions in each calendar year.

Included as covariates in the model were patient socio-demographic factors including age, sex, ethnicity and IMD decile of residence. We also included the count of QOF and non-QOF conditions for each patient. Due to small numbers, we excluded patients with gender recorded in CPRD as ‘indeterminate’ or with missing IMD deciles. Age and the count of QOF and non-QOF conditions were time-updated at the start of each calendar year, and other covariates were held fixed. We incorporated random effects for patient and fixed effects for calendar year as we wished to explicitly model the effect of time. Use of a Poisson model was considered, but the conditional variance was found to be significantly higher than the conditional mean ($p<0.001$) indicating a negative binomial to have better fit.(22) Model fit

For peer review only

was assessed by calculating randomized quantile residuals, which indicated no departure from normality on quantile-quantile plots.(23,24)

For each regression model, we calculated the predicted count of disease codes for each patient per year and then calculated the mean for each GP practice. This indicated that significant variation remained in the mean counts according to GP practice (appendix Figure A1). We therefore incorporated fixed effects for GP practice within the regression models to account for practice-level variation (see appendix p5 for model equation). We also compared the Akaike Information Criteria (AIC) of models with and without practice fixed effects.

To assess whether code frequency was a function of overall number of primary care consultations, we conducted a sensitivity analysis including average number of yearly consultations (irrespective of condition) in year 1 or year 2 added as a covariate into the main regression models (categorised into <1, 1-2, 3-4, 5-9 or 10 or more). Python version 3.10.6 and Pandas version 1.4.3 were used in data processing and plots and Stata version 17.0 and R studio version 4.2.1 were used for regression analyses.

Patient and Public Involvement

This research programme is supported by a patient and public advisory group who fed back to the researchers on the diseases included in the study but were not directly involved in this study.

For peer review only

Results

A total of 6,174,115 patients aged 18 years or over and with a continuous registration period between 1st January 2014 and 31st December 2020 were eligible for inclusion in the study. Of these, 3,113,724 (50.4%) had at least one incident disease diagnosed between 1st January 2015 and 31st December 2019. Characteristics of the eligible population are shown in Table 1. 21.4% of patients were aged between 18-40 years as of the study start date, and 7.0% were aged 80 years or over. There were more women than men (54.1% versus 45.9%), most (76.7%) were of White ethnicity and there were relatively more patients in more deprived IMD deciles (51.7% in the most deprived half). Of patients with pre-existing conditions developed before the study start date, 31.6% had one or more QOF conditions, and 71.3% had one or more non-QOF conditions. Hypertension was the most prevalent pre-existing condition (24.1%), and the frequency of all pre-existing conditions are shown in the appendix Table A2. The 3,060,391 patients who were not eligible (as they did not develop an incident disease over the study period), were more likely to be younger and more likely to be male than those eligible (appendix Table A3).

For peer review only

Table 1: Socio-demographic characteristics of patients included in the study

Patient characteristic	Total	Percent
**Age (years)**		
18-39	665543	21.4%
40-49	562934	18.1%
50-59	604284	19.4%
60-69	585062	18.8%
70-79	476626	15.3%
80+	219275	7.0%
**Gender**		
Female	1684942	54.1%
Male	1428734	45.9%
Indeterminate	48	<0.1%
**Ethnicity**		
White	2388332	76.7%
South Asian	194477	6.2%
Black	103504	3.3%
Other	36430	1.2%
Mixed	27572	0.9%
Missing	363409	11.7%
**IMD decile**		
1 (most deprived)	358948	11.5%
2	320042	10.3%
3	320340	10.3%
4	323782	10.4%
5	287114	9.2%
6	303798	9.8%
7	304044	9.8%
8	298185	9.6%
9	305563	9.8%
10 (least deprived)	290214	9.3%
Missing	1694	0.1%
**Pre-existing QOF conditions***		
0	2130680	68.4%
1	393905	12.7%
2	224147	7.2%
3	142104	4.6%
4 or more	222888	7.2%
**Pre-existing non-QOF conditions***		
0	893765	28.7%
1	561300	18.0%
2	506053	16.3%
3	386912	12.4%
4 or more	765694	24.6%
**Total**	**3113724**	

* Pre-existing conditions defined as of study start date

For peer review only

Code frequency by disease and by time from diagnosis

A total of 7,723,365 diseases were diagnosed during the study period with follow-up times for each disease ranging from 1.0 to 7.2 years (mean 4.1 years). There was substantial variation in the yearly code frequency after diagnosis for each condition diagnosed during the study period. Diabetes (types 1, 2 and unspecified), polymyalgia rheumatica, motor neurone disease and dementia had the highest median number of codes per year (appendix Table A4). For many chronic diseases, yearly code frequency was low, for example, only 5% of patients with spina bifida had ≥0.5 codes per year. Conditions included in QOF on average had significantly higher mean number of yearly codes (1.03) than conditions not included in QOF (0.32; p<0.0001).

The number of codes was higher in the first year after diagnosis than in subsequent years for almost all conditions, except for secondary bowel or pleural malignancy and diabetic eye disease, for which code frequency was higher on average after the first year of diagnosis. QOF conditions on average had lower ratios of codes in the first compared to subsequent years than non-QOF conditions (4.8 versus 5.7 times higher in year 1). However, diseases representing major cardiovascular events, such as myocardial infarction, were coded much more frequently in the first year from diagnosis than in subsequent years (appendix Figure A2 and Figure A3).

Variation in coding frequency by GP practice

There was a wide range in the mean yearly number of codes per condition between GP practices, with higher code frequency for QOF compared to non-QOF conditions (appendix Figure A4). There was a strong correlation (r = 0.88) between GP practice mean code frequency for QOF and non-QOF conditions, indicating that those practices with high code frequency for QOF conditions also had high code frequency for non-QOF conditions (Figure 1). There was no observed trend according to the GP practice-level IMD decile (appendix Figure A5).

Figure 1: Scatterplot of mean yearly number of codes following diagnosis for QOF versus non-QOF conditions for each GP practice

For peer review only

We calculated the expected counts of codes for new diseases in year 1 and year 2 following diagnosis, predicted from negative binomial regression models. Expected mean counts per condition at GP practice level showed substantially less variation compared to the observed mean counts for both QOF and non-QOF conditions in year 1 and year 2 (appendix Figure A1) indicating substantial residual practice level variation independent of patient socio-demographic factors.

Variation in disease frequency by socio-demographics and over time

We found significant associations between code frequency in year 1 and year 2 following diagnosis with patient socio-demographic factors and calendar year of diagnosis for both QOF and non-QOF diseases from mixed effects negative binomial regression, after adjustment for number of pre-existing conditions (Figures 2 and 3, and appendix Tables A5 – A8). Inclusion of GP practice fixed effects in the regression models resulted in very similar coefficients for patient sociodemographic factors, and a significantly lower AIC indicating better model fit and so results are presented including practice-level effects.

<u>Associations with QOF conditions</u>

Younger patients tended to have a higher frequency of codes in the first year following diagnosis compared to older patients (Figure 1). However, in the second year from diagnosis, there was a U-shaped relationship with age, with the youngest and oldest age groups having the lowest rate of codes. Males had on average a small 3% increase (95% CI: 1.03 – 1.03) in the incidence rate of codes in year 1 and 11% (95% CI: 1.11 – 1.12) increase in year 2 compared with females. There was a strong relationship with ethnicity, with people of non-White ethnicities having lower rates of code frequency than people of White ethnicity in year 1, but higher rates in year 2. There was a strong trend towards higher code frequency in year 1 and year 2 with decreasing levels of deprivation.

<u>Associations with non-QOF conditions</u>

For conditions not included in QOF, relationships were more consistent across year 1 and year 2 following diagnosis (Figure 2). The 18–40-year age group had the highest rate of codes in both year 1 and year 2, with only small differences between other age groups. There was no difference in the rate of codes in males and females in year 1, but males had a lower rate of codes in year 2. Lower rates of codes were found in people of non-White ethnicities

For peer review only

compared to people of White ethnicity, except for South Asian ethnicity in year 2. Similar to QOF conditions, there was a strong trend towards higher code rates in year 1 and year 2 with decreasing deprivation.

Associations with calendar year

For both QOF and non-QOF conditions, code rates were similar for conditions diagnosed in 2016 and 2017 compared with 2015 (Figures 1 and 2). For codes in year 1, rates for conditions diagnosed in 2018 were similar to 2015, but rates for diseases diagnosed in 2019 were 5% and 6% lower than 2015 for QOF and non-QOF conditions, respectively. For codes in year 2, rates were significantly lower in 2018 (9% and 9% lower for QOF and non-QOF, respectively) and 2019 (21% and 21% lower for QOF and non-QOF, respectively) compared to 2015.

Adjustment for total number of consultations

A sensitivity analysis was used to adjust for total number of consultations in year 1 or year 2 from diagnosis (Tables A5-A8). Total number of consultations in each year were strongly linked to the rate of codes. For newly diagnosed QOF conditions, the associations with age, sex and ethnicity in years 1 and 2 remained significant after adjustment (Tables A5-A6). However, the association with deprivation was attenuated, although there remained an association with higher rates of codes with lower deprivation in year 2. For newly diagnosed non-QOF conditions, after adjustment for consultations, age and ethnicity remained significantly associated, but males had significantly higher rates of codes than females (Tables A7-A8). Associations with deprivation were attenuated, but there remained a small but significant association in year 2.

Figure 2: Associations of rate of codes in year one and year two following diagnosis with patient characteristics and calendar year, for conditions included in the Quality and Outcomes Framework (QOF)

Figure 3: Associations of rate of codes in year one and year two following diagnosis with patient characteristics and calendar year, for conditions not included in the Quality and Outcomes Framework (QOF)

For peer review only

Discussion

With an increased use of NLP methods incorporating temporally-ordered code sequences in the primary care EHR, we need to better understand the structure and frequency of repeated occurrences of diagnostic codes. Our study demonstrates significant associations in the frequency of codes for newly diagnosed conditions according to patient socio-demographic factors, GP practice, disease inclusion in QOF, and calendar year. We are unable to fully assess the extent to which the relationships in our study are explained by the quality of coding, or by how patients use healthcare services for a particular condition. However, a sensitivity analysis adjusting for total number of yearly consultations per patient yielded similar results, suggesting that variation in coding quality is likely to play a role. Our findings have implications for researchers using code sequences, emphasising the importance of considering these factors as potential sources of bias.

<u>Patient socio-demographics</u>

Patient characteristics including age, sex and ethnicity were strongly linked to code frequency, although associations were inconsistent across QOF and non-QOF conditions, and for QOF conditions, were not consistent across the first and second year from diagnosis. People of non-White ethnicity, for example, had lower code rates for QOF conditions in year 1, but higher in year 2, compared to people of White ethnicity. We found consistent patterns with deprivation, with lower code frequency in people living in more deprived areas. A sensitivity analysis adjusting for total number of consultations attenuated the association with deprivation, suggesting that the relationship of code frequency with deprivation was partially explained by total primary care contacts. These findings likely point to differences in the mix of conditions between patient groups, healthcare seeking behaviours, or access to care. For example, people living in areas of socio-economic deprivation may be less likely to attend for screening, preventive care and ongoing management of chronic diseases. Previous research also suggests that although rates of appointments are similar across deciles of socioeconomic deprivation,(25) the rate of missed appointments increases and consultation length decreases with increasing deprivation, which may impact on code frequency for these groups, rather than indicating differences in healthcare need.(26,27)

<u>GP practice</u>

Substantial variation was found in the frequency of codes between GP practices, which persisted after accounting for differences in patient mix in terms of age, sex, deprivation,

For peer review only

ethnicity, number of chronic conditions and in year of diagnosis. Although this may indicate unmeasured confounding in the characteristics of patients between practices, it likely represents policies and practices that influence coding which vary between organisations and clinicians.(9) For example, some GP practices may be more rigorous about coding data in clinical consultations and in correspondence from specialist services on diagnoses made in secondary care. Previous research has suggested that clinicians are more similar to those in the same practice than they are to clinicians in different practices with respect to treatment and diagnostic decisions.(28) Variation between clinicians in coding practices is likely to be significant both within and between practices, but this information was not accessible for the study, and its analysis would introduce multiple hierarchical dependencies outside the scope of this work. Future work could consider individual clinician effects on coding practices in the EHR.

QOF and non-QOF conditions

Code frequency was significantly higher for conditions included in QOF compared to conditions not included. Previous research has highlighted changes to policies and procedures within GP practices to meet targets, including improved disease registries, which may lead to an increased likelihood of a code being entered for a given condition. We found substantial variation between GP practices in the mean code frequency for QOF conditions, but interestingly, this was strongly correlated (r=0.88 and Figure 1) with code frequency for non-QOF conditions, suggesting that practice-level effects impact on coding across all conditions, rather than specifically those incentivised by QOF. However, it is not possible in our study to determine whether differences in code frequency between QOF and non-QOF conditions are explained by greater healthcare need or an increased number of healthcare contacts for QOF conditions, or are explained by higher likelihood of a condition being coded when a patient presents.

Calendar year

Accounting for calendar time in analyses of patient trajectories is a methodological concern, as the further back in time in the medical record, particularly before the advent of the EHR and QOF, the greater the chance that coding practices, and even disease categories, vary.(29) Although our study started relatively recently in 2015, and we cannot infer code frequency before this time, we found consistency in code frequency over a short time-span from 2015-2017. The decline in year 1 codes in 2019, and year 2 codes in 2018 and 2019 likely relates to

For peer review only

the impact of the COVID-19 pandemic which impacted significantly on health services in England from March 2020.(30) Previous studies have shown reductions in patients presenting with particular conditions, and a reduction in appointment numbers in primary and secondary healthcare in England. Analyses reliant on coding frequency should therefore consider using calendar year in addition to patient age in modelling patient trajectories, or limiting analyses to defined time period.

Strengths and limitations

A strength of our study is the inclusion of a large number of patients from a representative sample of primary care in England which makes our findings generalisable to the national population.(15) We included only patients with newly incident diseases to minimise potential confounding from diseases diagnosed historically, some of which might no longer be active. We also only included patients with continuous follow-up over the study period and with at least one year of full practice registration to reduce bias from overestimation of incidence immediately following registration.(17) We also excluded patients who died less than one year from a new diagnosis, which may impact on disease frequency estimates for disease which have poor survival. We considered using annualised rates for those with less than a full year of follow-up, but this resulted in very high annualised counts for some individuals with short follow-up and might introduce additional bias if patients were to seek out care in advance of re-registering at another GP practice.

Our study has focussed on structured healthcare data, whereas much of the consultation is recorded as unstructured 'free-text'.(7) Although unstructured primary care data contains much richer information on the details of a presentation that may not be fully reflected in the coded entries, this information is not currently available from CPRD, but research in future could examine the agreement between structured and unstructured primary care EHR data. This would allow a more robust estimation of the content and diseases covered during a consultation. We stratified conditions according to QOF status given our hypothesis that it may influence coding frequency. However, we also found variation within categories; for example, polymyalgia rheumatica and motor neurone disease, which are not included in QOF, had high number of yearly codes, whereas cardiovascular events such as Transient Ischaemic Attack, included in QOF, had low yearly codes. Given the general, comparative nature of this paper, and its aim to examine relationships over many conditions, a condition-specific analysis of coding frequency was out of scope.

For peer review only

Implications for research

Our findings have implications for researchers using code sequences recorded in primary care structured data. The frequency of repeated diagnostic codes relates to patient and condition-specific factors, coding incentives and practice-level factors. Although we cannot determine if these findings represent disease burden and healthcare need, it is likely that biases in coding operate at various levels. Specific approaches to reduce the impact of bias will depend on the methodology, but our work does suggest general principles.

Firstly, to consider the potential for bias within the data source and whether stratification may reduce it, for example, by selecting a smaller number of healthcare organisations or a narrower time period. Secondly, to consider adjustment or inclusion of patient, condition, GP practice and calendar year variables within analytical models. However, such an approach is not always recommended, particularly if prediction is the aim, as inclusion of factors such as ethnicity in algorithms may reinforce existing bias.(31) In NLP, text style transfer is often used as a method to control for different styles of writing, which may have relevance to approaches to account for the different coding styles of clinicians.(32) However, these approaches are complicated within the EHR as people are likely to see multiple different clinicians over time, with a small set of codes recorded at each visit. Finally, it is vital that generated representations or predictions from modelling are evaluated in different patient subgroups.

Implications for clinical practice

Although difficult to determine the extent to which our findings are attributed to coding quality versus healthcare utilisation, previous studies have reported variability in coding across practices for specific conditions.(33,34) This highlights a need to improve the quality of coding in primary care, given its impact on the reliability and usefulness of the data for secondary purposes such as research. Improving the quality of coding in primary care poses several challenges, due to the different incentives for clinicians, who document most of the consultation in free text.(7) Potential strategies include implementing structured templates for recording consultations, or developing NLP methods capable of interpreting and codifying the free-text documented during clinical encounters, without adding to clinician workload.(7)

Conclusion

For peer review only

Our study found significant variation in the frequency of diagnostic codes recorded in the primary care EHR after diagnosis, related to patient socio-demographics, coding incentives and GP practice, and a significant reduction in the frequency of codes associated with the onset of the COVID-19 pandemic. These factors should be considered by researchers using NLP methods, or other approaches using temporally ordered sequences of codes in primary care EHRs, to reduce the risk of bias.

Funding

This research is funded through a clinical PhD fellowship awarded to TB from the Wellcome Trust 4i programme at Imperial College London (grant number N/A). JC acknowledge support from the Wellcome Trust (grant number N/A). MB acknowledges support from EPSRC grant EP/N014529/1 supporting the EPSRC Centre for Mathematics of Precision Healthcare. TW, AM and PA acknowledge support from the National Institute for Health and Care Research (NIHR) under the Applied Research Collaboration (ARC) Northwest London (grant number N/A). The views expressed in this publication are those of the authors and not necessarily those of the NHS, the NIHR, the Wellcome Trust or the Department of Health and Social Care.

Competing interests

The authors have no competing interests to declare

Contributor statement

TB conceptualised the study, conducted the data management and formal analysis and wrote the first draft of the manuscript. TB, JS, DS, TW, AM, MB and PA contributed to the study design, methodology, interpretation of findings and reviewing and editing the manuscript. TB is the guarantor and accepts full responsibility for the work and the conduct of the study, had access to the data, and controlled the decision to publish. The corresponding author attests that all listed authors meet authorship criteria and that no others meeting the criteria have been omitted.

Data sharing

The data used in this study are not publicly available as access is subject to approval processes. More information is available from CPRD: https://cprd.com/research-applications

For peer review only

Ethics approval

Data access to the Clinical Practice Research Datalink (CPRD) and ethical approval was granted by CPRD's Research Data Governance Process on 28th April 2022 (Protocol reference: 22_001818).

Acknowledgements

Data management was provided by the Big Data and Analytical Unit (BDAU) at the Institute of Global Health Innovation (IGHI).

For peer review only

References

1. Li Y, Rao S, Solares JRA, Hassaine A, Ramakrishnan R, Canoy D, et al. BEHRT: Transformer for Electronic Health Records. Sci Rep. 2020 Apr 28;10(1):7155.

2. Solares JRA, Zhu Y, Hassaine A, Rao S, Li Y, Mamouei M, et al. Transfer Learning in Electronic Health Records through Clinical Concept Embedding. 2021;1–14.

3. Altuncu MT, Mayer E, Yaliraki SN, Barahona M. From free text to clusters of content in health records: an unsupervised graph partitioning approach. Applied Network Science. 2019 Dec;4(1):2.

4. Kraljevic Z, Searle T, Shek A, Roguski L, Noor K, Bean D, et al. Multi-domain clinical natural language processing with MedCAT: The Medical Concept Annotation Toolkit. Artif Intell Med. 2021 Jul;117:102083.

5. Rasmy L, Xiang Y, Xie Z, Tao C, Zhi D. Med-BERT: pretrained contextualized embeddings on large-scale structured electronic health records for disease prediction. npj Digit Med. 2021 May 20;4(1):1–13.

6. Choi E, Bahadori MT, Searles E, Coffey C, Thompson M, Bost J, et al. Multi-layer representation learning for medical concepts. Proceedings of the ACM SIGKDD International Conference on Knowledge Discovery and Data Mining. 2016;13-17-Augu:1495–504.

7. Shemtob L, Beaney T, Norton J, Majeed A. How can we improve the quality of data collected in general practice? BMJ. 2023 Mar 15;380:e071950.

8. Khan NF, Harrison SE, Rose PW. Validity of diagnostic coding within the General Practice Research Database: a systematic review. British Journal of General Practice. 2010;60(572):e128--e136.

9. Verheij RA, Curcin V, Delaney BC, McGilchrist MM. Possible Sources of Bias in Primary Care Electronic Health Record Data Use and Reuse. J Med Internet Res. 2018 May 29;20(5):e185.

10. Bots SH, Groenwold RHH, Dekkers OM. Using electronic health record data for clinical research: a quick guide. European Journal of Endocrinology. 2022 Apr 1;186(4):E1–6.

11. Beech J, Beccy Baird. The King's Fund. 2020 [cited 2023 Jun 23]. GP funding and contracts explained. Available from: https://www.kingsfund.org.uk/publications/gp-funding-and-contracts-explained

12. Forbes LJ, Marchand C, Doran T, Peckham S. The role of the Quality and Outcomes Framework in the care of long-term conditions: a systematic review. Br J Gen Pract. 2017 Nov 1;67(664):e775.

13. Minchin M, Roland M, Richardson J, Rowark S, Guthrie B. Quality of Care in the United Kingdom after Removal of Financial Incentives. New England Journal of Medicine. 2018 Sep 5;379(10):948–57.

14. Roland M, Guthrie B. Quality and Outcomes Framework: what have we learnt? BMJ. 2016 Aug 4;354:i4060.

15. Wolf A, Dedman D, Campbell J, Booth H, Lunn D, Chapman J, et al. Data resource profile: Clinical Practice Research Datalink (CPRD) Aurum. International Journal of Epidemiology. 2019 Dec 1;48(6):1740–1740g.

16. Herrett E, Gallagher AM, Bhaskaran K, Forbes H, Mathur R, van Staa T, et al. Data Resource Profile: Clinical Practice Research Datalink (CPRD). International Journal of Epidemiology. 2015 Jun 1;44(3):827–36.

17. Lewis JD, Bilker WB, Weinstein RB, Strom BL. The relationship between time since registration and measured incidence rates in the General Practice Research Database. Pharmacoepidemiology and Drug Safety. 2005 Jul 1;14(7):443–51.

18. Ministry of Housing, Communities & Local Government. English indices of deprivation 2019 [Internet]. [cited 2020 Oct 18]. Available from: https://www.gov.uk/government/statistics/english-indices-of-deprivation-2019

19. Mathur R, Bhaskaran K, Chaturvedi N, Leon DA, vanStaa T, Grundy E, et al. Completeness and usability of ethnicity data in UK-based primary care and hospital databases. Journal of Public Health. 2014 Dec 1;36(4):684–92.

20. Head A, Fleming K, Kypridemos C, Schofield P, Pearson-Stuttard J, O'Flaherty M. Inequalities in incident and prevalent multimorbidity in England, 2004–19: a population-based, descriptive study. The Lancet Healthy Longevity. 2021 Aug 1;2(8):e489–97.

21. Kuan V, Denaxas S, Gonzalez-Izquierdo A, Direk K, Bhatti O, Husain S, et al. A chronological map of 308 physical and mental health conditions from 4 million individuals in the English National Health Service. The Lancet Digital Health. 2019 Jun 1;1(2):e63–77.

22. Dean CB. Testing for Overdispersion in Poisson and Binomial Regression Models. Journal of the American Statistical Association. 1992;87(418):451–7.

For peer review only

23. Dunn PK, Smyth GK. Randomized Quantile Residuals. Journal of Computational and Graphical Statistics. 1996 Sep;5(3):236–44.

24. Feng C, Li L, Sadeghpour A. A comparison of residual diagnosis tools for diagnosing regression models for count data. BMC Med Res Methodol. 2020 Jul 1;20:175.

25. Fisher R, Dunn P, Asaria M, Thorlby R. Comparing general practice in areas of high and low socioeconomic deprivation in England. :30.

26. Ellis DA, McQueenie R, McConnachie A, Wilson P, Williamson AE. Demographic and practice factors predicting repeated non-attendance in primary care: a national retrospective cohort analysis. The Lancet Public Health. 2017 Dec 1;2(12):e551–9.

27. Gopfert A, Deeny SR, Fisher R, Stafford M. Primary care consultation length by deprivation and multimorbidity in England: an observational study using electronic patient records. Br J Gen Pract. 2021 Mar 1;71(704):e185–92.

28. Jong J de, Groenewegen P, Westert G. Medical practice variation: does it cluster within general practitioners' practices? In: Morbidity, Performance and Quality in Primary Care. CRC Press; 2006.

29. Gluckman PD. Evolving a definition of disease. Archives of Disease in Childhood. 2007 Dec;92(12):1053.

30. Majeed A, Maile EJ, Bindman AB. The primary care response to COVID-19 in England's National Health Service. J R Soc Med. 2020 Jun;113(6):208–10.

31. Obermeyer Z, Powers B, Vogeli C, Mullainathan S. Dissecting racial bias in an algorithm used to manage the health of populations. Science. 2019;366(6464):447–53.

32. Jin D, Jin Z, Hu Z, Vechtomova O, Mihalcea R. Deep Learning for Text Style Transfer: A Survey [Internet]. arXiv; 2021 [cited 2022 Nov 23]. Available from: http://arxiv.org/abs/2011.00416

33. de Lusignan S, Valentin T, Chan T, Hague N, Wood O, van Vlymen J, et al. Problems with primary care data quality: osteoporosis as an exemplar. Inform Prim Care. 2004;12(3):147–56.

34. Rollason W, Khunti K, de Lusignan S. Variation in the recording of diabetes diagnostic data in primary care computer systems: implications for the quality of care. Inform Prim Care. 2009;17(2):113–9.

Figure 1 legend:

Note: different ranges used in each axis

Figure 2 legend:

For peer review only

Note: Points represent estimates of the incidence rate ratio and bars represent 95% confidence intervals from negative binomial regression models. Corresponding values and coefficients for pre-existing QOF and non-QOF conditions are given in appendix Tables A5 and A6.

Figure 3 legend:

Note: Points represent estimates of the incidence rate ratio and bars represent 95% confidence intervals from negative binomial regression models. Corresponding values and coefficients for pre-existing QOF and non-QOF conditions are given in appendix Tables A7 and A8.

For peer review only

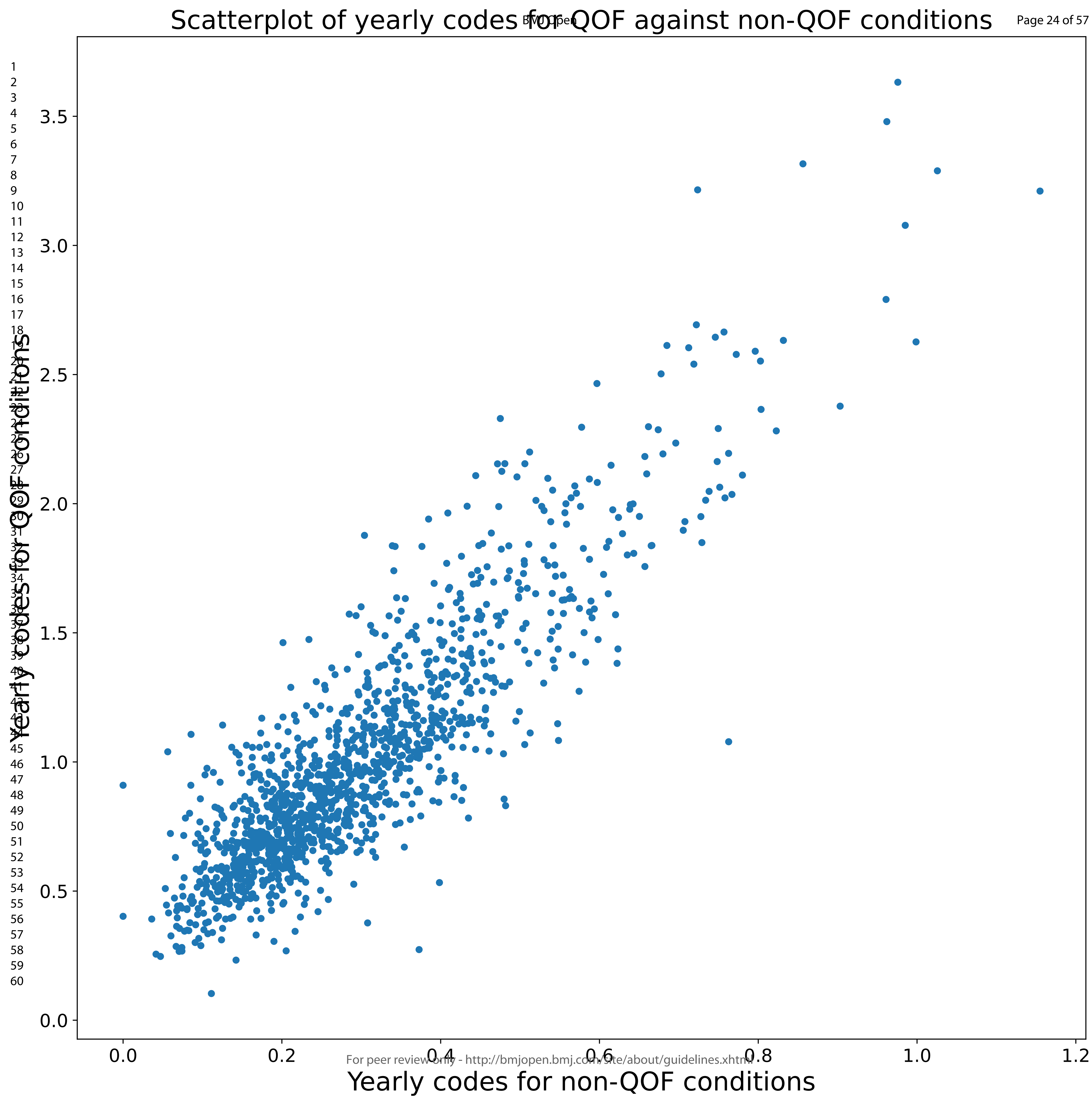
Scatterplot of yearly codes for QOF against non-QOF conditions
Yearly codes for QOF conditions
Yearly codes for non-QOF conditions
0.0
0.5
1.0
1.5
2.0
2.5
3.0
3.5
0.0
0.2
0.4
0.6
0.8
1.0
1.2

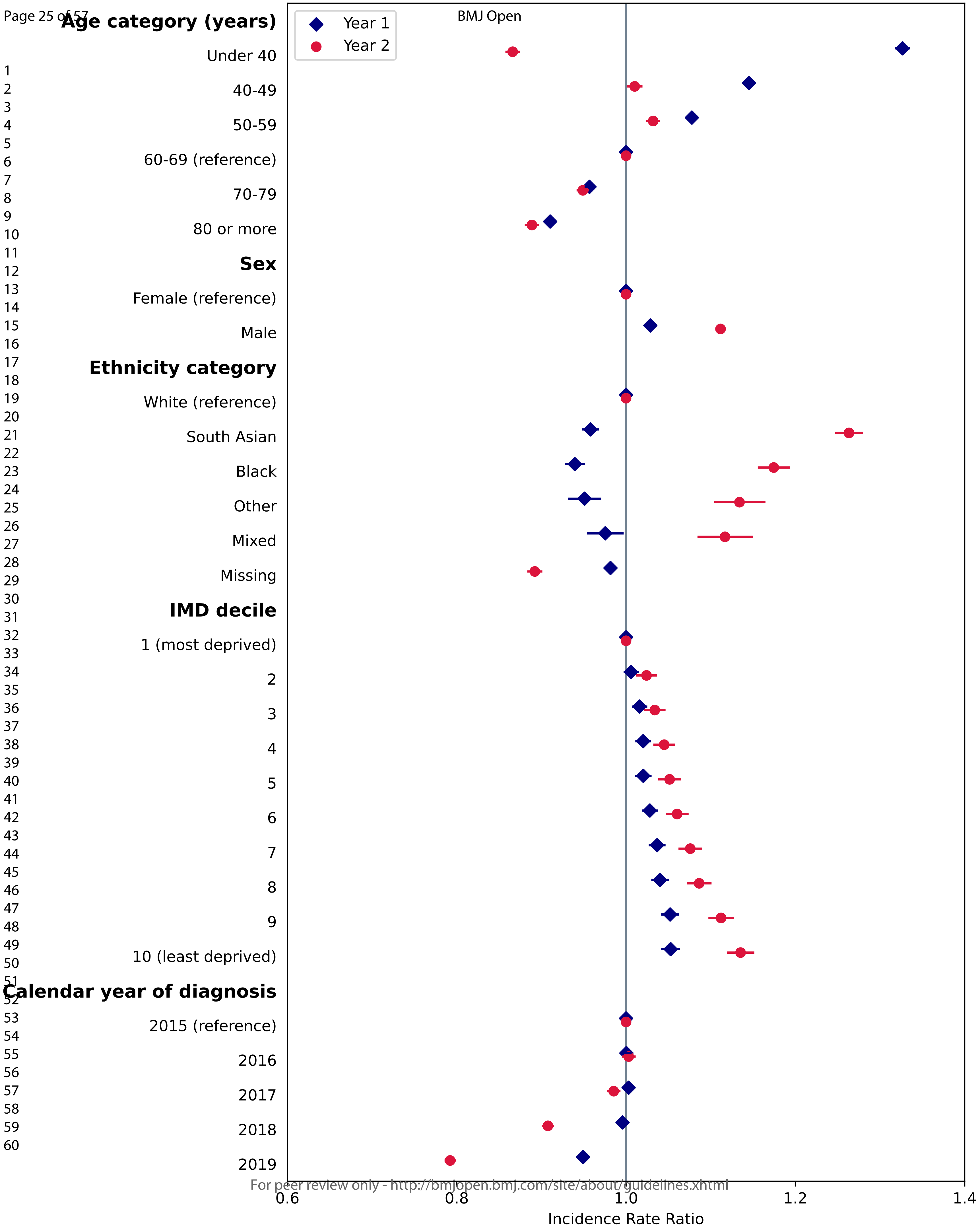
Year 1
Year 2
Age category (years)
Under 40
40-49
50-59
60-69 (reference)
70-79
80 or more
Sex
Female (reference)
Male
Ethnicity category
White (reference)
South Asian
Black
Other
Mixed
Missing
IMD decile
1 (most deprived)
2
3
4
5
6
7
8
9
10 (least deprived)
Calendar year of diagnosis
2015 (reference)
2016
2017
2018
2019
0.6
0.8
1.0
1.2
1.4
Incidence Rate Ratio

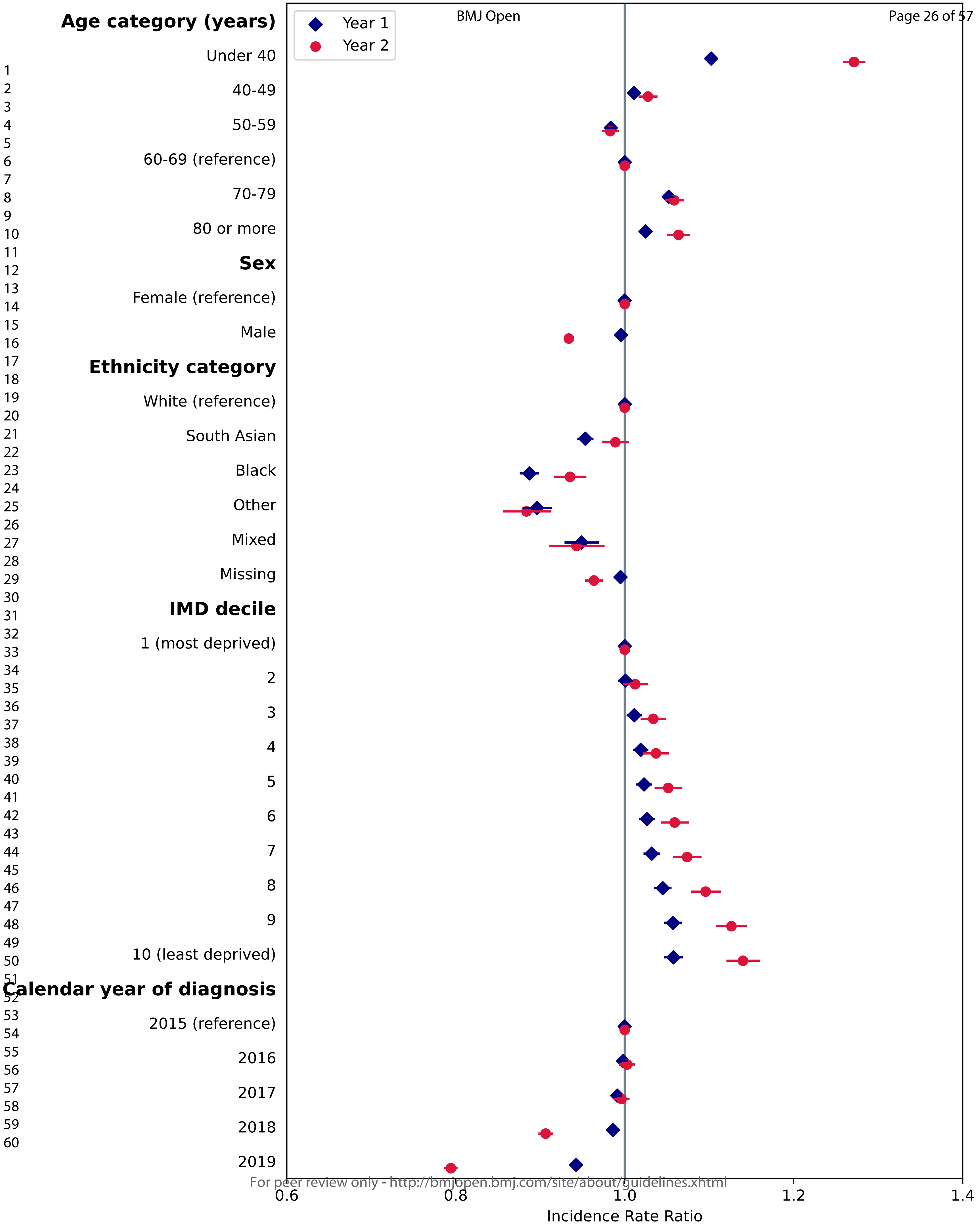
Year 1
Year 2
Age category (years)
Under 40
40-49
50-59
60-69 (reference)
70-79
80 or more
Sex
Female (reference)
Male
Ethnicity category
White (reference)
South Asian
Black
Other
Mixed
Missing
IMD decile
1 (most deprived)
2
3
4
5
6
7
8
9
10 (least deprived)
Calendar year of diagnosis
2015 (reference)
2016
2017
2018
2019
0.6
0.8
1.0
1.2
1.4
Incidence Rate Ratio

Appendix

Identifying potential biases in code sequences in primary care electronic healthcare records: a retrospective cohort study of the determinants of code frequency

Beaney T[1,2] (0000-0001-9709-7264), Clarke J[2], Salman D [1,3], Woodcock T[1], Majeed A[1], Barahona M[2], Aylin P[1]

1. Department of Primary Care and Public Health, Imperial College London, London, W6 8RP, United Kingdom
2. Centre for Mathematics of Precision Healthcare, Department of Mathematics, Imperial College London, London, SW7 2AZ, United Kingdom
3. MSk Lab, Department of Surgery and Cancer, Faculty of Medicine, Imperial College London, London, UK

Corresponding Author:
Dr Thomas Beaney
Department of Primary Care and Public Health, Imperial College London, London, W6 8RP, United Kingdom
Email: thomas.beaney@imperial.ac.uk

For peer review only

Patients were included with continuous registration dates between 1st January 2014 and 31st December 2020. The 1st January 2014 was chosen to allow for a full one year of registration at a GP practice prior to follow-up, to reduce the potential impact of bias from newly registered patients having pre-existing conditions coded for the first time at their new practice. The end date of 31st December 2020 was chosen to provide at least one full year of follow-up for conditions newly diagnosed in 2019. Patients were followed up until the earliest date of death, deregistration and latest date of data extraction from their practice, if after 31st December 2020. The earliest possible censoring date for a patient was 1st January 2021 and the last date of follow-up for a patient was 21st March 2022.

Chronic conditions

Diseases were mapped using code lists developed for the CALIBER study, and adapted for use in multimorbidity in CPRD Aurum.[1,2] We reviewed the codes in these lists, and made amendments to the code lists for diabetes. The 'other/unspecified' diabetes code list contained codes specific to both Type 1 and Type 2 diabetes, and we removed these to ensure the list included only codes where a more specific Type 1 or Type 2 diagnosis was not stated. We added chronic primary pain to the set of included conditions and created a new code list. Previous studies of multimorbidity in primary care settings have found a high prevalence and burden of chronic pain.[3,4] However, in order to avoid double counting of pain related to another chronic condition included, we excluded secondary causes, and included only primary pain conditions.

Assignment to QOF

Diseases were classified as included or not included in QOF by two clinicians with experience working as GPs: TB and DS. The first QOF year in 2004/2005 included eleven diseases, with new conditions added in subsequent years.[5] Rheumatoid arthritis was added to QOF in 2013/2014, but there were no subsequent additions of any of the diseases included in this study.[6] However, hypothyroidism was included in QOF from its start until 2014/15 when it was removed.[7] The thyroid disease category from CALIBER included codes for both hypothyroidism and hyperthyroidism. We therefore excluded the thyroid disease category from comparisons of QOF to avoid any carry-over effect from prior inclusion in QOF, and dilution from non-hypothyroid conditions. The following QOF conditions from 2014/15 to 2019/20 were included:

For peer review only - http://bmjopen.bmj.com/site/about/guidelines.xhtml

1. Coronary Heart Disease
2. Left Ventricular Dysfunction / Heart Failure (from 2006)
3. Stroke (and TIA from 2006)
4. Hypertension
5. Diabetes
6. COPD
7. Epilepsy
8. Cancer
9. Mental Health
10. Asthma
11. Dementia
12. Depression
13. CKD
14. Atrial fibrillation
15. Obesity
16. Learning disabilities
17. Palliative care
18. Smoking
19. Cardio-vascular disease (primary prevention)
20. Peripheral Arterial Disease (PAD)
21. Osteoporosis
22. Rheumatoid arthritis

For analyses of counts per calendar year, the total counts of disease codes were calculated for the first and second year from diagnosis. Counts were stratified according to whether a condition was included in QOF. A patient was included for a given calendar year if they had at least one QOF or non-QOF condition diagnosed in that year, as shown in Table A1.

For peer review only

Table A1: example of the stratification of condition and calendar year for each newly diagnosed condition for three hypothetical patients

Patient	Age	Condition	Calendar year	Count in year one	Count in year two
1	67	QOF	2015	0	0
1	68	QOF	2016	2	0
1	70	QOF	2018	4	2
1	67	Non-QOF	2015	1	1
2	28	Non-QOF	2019	1	2
3	52	QOF	2017	5	4
3	52	Non-QOF	2017	2	2

For peer review only

Statistical analyses

Mixed effects negative binomial models were constructed. We considered use of a zero-inflated model, but coefficients from the logit and negative binomial components of the model were similar, and so in the interests of interpretable findings, the more parsimonious negative binomial model was selected.

Equation for the mixed effects negative binomial regression model, including fixed effects for calendar year and GP practice and random effects for patient:

$$log(y_{i,j}) = \beta_0 + \beta_1 age_{i,j} + \beta_2 gender_{i,j} + \beta_3 ethnicity_{i,j} + \beta_4 IMD_{i.j} + \beta_5 year_{i.j} + \beta_6 GP_{i.j} + u_j$$

where *i* represents QOF or non-QOF conditions newly diagnosed in patient *j* and $y_{i,j}$ is the count of codes in the given year.

For peer review only - http://bmjopen.bmj.com/site/about/guidelines.xhtml

A2: Frequency and percentage of pre-existing diseases (as of 1st January 2015) for all 3,113,724 eligible patients

Pre-existing disease	Frequency	Percentage
Hypertension	751009	24.12%
Enthesopathy and synovial disorder	736087	23.64%
Dermatitis	710945	22.83%
Depression	568871	18.27%
Anxiety disorders	507406	16.30%
Allergic and chronic rhinitis	477053	15.32%
Asthma	456335	14.66%
Osteoarthritis (excl spine)	444668	14.28%
Gastro-oesophageal reflux disease	301839	9.69%
Obesity	294916	9.47%
Diabetes Mellitus: other or not specified	285681	9.17%
Hearing loss	279470	8.98%
Migraine	270415	8.68%
Type 2 Diabetes Mellitus	255578	8.21%
Irritable bowel syndrome	246744	7.92%
Abdominal Hernia	237968	7.64%
Acne	225183	7.23%
Chronic sinusitis	212496	6.82%
Thyroid Disease	204639	6.57%
Spondylosis	181722	5.84%
Gastritis and duodenitis	181668	5.83%
Cataract	160486	5.15%
Chronic Kidney Disease	158134	5.08%
Coronary Heart Disease (not otherwise specified)	144806	4.65%
Seborrheic dermatitis	143168	4.60%
Urinary Incontinence	137919	4.43%
Alcohol Misuse	132717	4.26%
Psoriasis	132694	4.26%
Diaphragmatic hernia	131539	4.22%
Diverticular Disease	131332	4.22%
Tinnitus	123308	3.96%
Gout	120568	3.87%
Stable Angina	120309	3.86%
Intervertebral disc disorders	117787	3.78%
Anaemia: other	116859	3.75%
Diabetic Eye Disease	102901	3.30%
Rosacea	96511	3.10%
Dysmenorrhoea	94881	3.05%

For peer review only - http://bmjopen.bmj.com/site/about/guidelines.xhtml

Benign Prostatic Hyperplasia	92304	2.96%
Osteoporosis	91850	2.95%
Primary Malignancy: Skin	89500	2.87%
COPD	84482	2.71%
Atrial Fibrillation	80645	2.59%
Peripheral Neuropathy	77117	2.48%
Chronic Fatigue Syndrome	67489	2.17%
Myocardial Infarction	67215	2.16%
Vitamin B12 deficiency anaemia	64015	2.06%
Glaucoma	58081	1.87%
Epilepsy	53058	1.70%
Stroke: not otherwise specified	50614	1.63%
Substance Misuse	50251	1.61%
Primary Malignancy: Breast	49737	1.60%
Venous thromboembolic disease (Excl PE)	47013	1.51%
Transient ischaemic attack	44616	1.43%
Fibromatosis	42701	1.37%
Neuropathic Bladder	42008	1.35%
Raynaud's syndrome	38879	1.25%
Endometriosis	37868	1.22%
Sleep apnoea	35743	1.15%
Heart failure	35364	1.14%
Peripheral Vascular Disease	32852	1.06%
Rheumatoid Arthritis	32070	1.03%
Macular degeneration	30761	0.99%
Chronic primary pain	29506	0.95%
Anterior and Intermediate Uveitis	28838	0.93%
Visual impairment and blindness	28372	0.91%
Polymyalgia Rheumatica	27447	0.88%
Primary Malignancy: Prostate	26288	0.84%
Ulcerative colitis	22236	0.71%
Nonrheumatic mitral valve disorders	20980	0.67%
Spinal stenosis	20820	0.67%
Nonrheumatic aortic valve disorders	20695	0.66%
Schizophrenia	20394	0.65%
Type 1 Diabetes Mellitus	19978	0.64%
Unstable Angina	18925	0.61%
Trigeminal neuralgia	18854	0.61%
Scleritis and episcleritis	18830	0.60%
Fatty Liver	18774	0.60%
Barrett's oesophagus	18152	0.58%
Supraventricular tachycardia	18128	0.58%
Intellectual disability	18073	0.58%
Pancreatitis	18043	0.58%

For peer review only

Bronchiectasis	18006	0.58%
Primary Malignancy: Melanoma	17594	0.57%
Personality disorders	17448	0.56%
Alopecia areata	17111	0.55%
Primary Malignancy: Bowel	16746	0.54%
Obsessive-compulsive disorder	15553	0.50%
Polycystic ovarian syndrome	14606	0.47%
Crohn's disease	14445	0.46%
Folate deficiency anaemia	13853	0.44%
Retinal vascular occlusions	13829	0.44%
Obstructive and reflux uropathy	13725	0.44%
Ischaemic stroke	13451	0.43%
Hidradenitis suppurativa	13305	0.43%
Vitiligo	13218	0.42%
Meniere's Disease	13192	0.42%
Bipolar affective disorder and mania	12856	0.41%
Coeliac disease	12625	0.41%
Diabetic Neuropathy	12517	0.40%
Chronic viral hepatitis	11885	0.38%
Thrombophilia	11527	0.37%
Psoriatic Arthritis	11201	0.36%
Eating Disorders	11171	0.36%
Dementia	10297	0.33%
Spondylolisthesis	10229	0.33%
Secondary Thrombocytopaenia	9800	0.31%
Congenital Septal Defect	9203	0.30%
Sarcoidosis	9090	0.29%
Multiple sclerosis	9070	0.29%
Benign essential tremor	9008	0.29%
Right bundle branch block combinations	8160	0.26%
Primary Malignancy: Bladder	8066	0.26%
Primary Malignancy: other	8021	0.26%
Glomerulonephritis	7950	0.26%
Autism and Asperger's syndrome	7920	0.25%
Non-Hodgkin Lymphoma	7579	0.24%
Hyperparathyroidism	7437	0.24%
Pleural effusion	7368	0.24%
Hyperkinetic disorders	7056	0.23%
Ankylosing spondylitis	7044	0.23%
Lupus Erythematosus	6976	0.22%
Cirrhosis	6768	0.22%
Alcoholic liver disease	6621	0.21%
Left bundle branch block	6512	0.21%
Subarachnoid haemorrhage	6158	0.20%

For peer review only

Collapsed vertebra	6082	0.20%
Autonomic Neuropathy	5496	0.18%
Cardiomyopathy: other	5465	0.18%
Parkinson's disease	5333	0.17%
Leukaemia	5243	0.17%
Giant Cell arteritis	5225	0.17%
Hyposplenism	4737	0.15%
HIV	4697	0.15%
Endometrial hyperplasia and hypertrophy	4655	0.15%
Primary Malignancy: Uterus	4589	0.15%
Sjogren's Syndrome	4559	0.15%
Spina bifida	4427	0.14%
Cerebral Palsy	4011	0.13%
Primary Thrombocytopaenia	3979	0.13%
Pleural plaque	3972	0.13%
Abdominal Aortic Aneurysm	3931	0.13%
Atrioventricular blocks	3920	0.13%
Chronic Cystitis	3892	0.12%
Intracerebral haemorrhage	3815	0.12%
Primary Malignancy: Ovary	3689	0.12%
Primary Malignancy: Cervix	3500	0.11%
Asbestosis	3358	0.11%
Other haemolytic anaemias	3152	0.10%
Primary Malignancy: Testis	3133	0.10%
Thalassaemia	3055	0.10%
Hypertrophic Nasal Turbinates	3022	0.10%
Primary Malignancy: Kidney	2988	0.10%
Polycythaemia vera	2864	0.09%
Primary Malignancy: Oropharyngeal	2809	0.09%
Autoimmune liver disease	2792	0.09%
Ventricular tachycardia	2720	0.09%
Secondary polycythaemia	2625	0.08%
Posterior Uveitis	2540	0.08%
Pulmonary Fibrosis	2523	0.08%
Hodgkin Lymphoma	2384	0.08%
Hypersplenism	2362	0.08%
Dilated cardiomyopathy	2359	0.08%
Primary Malignancy: Lung	2244	0.07%
Primary Malignancy: Thyroid	2172	0.07%
Rheumatic Valve Disorder	2034	0.07%
Secondary Malignancy_other	1975	0.06%
Down's syndrome	1928	0.06%
Multiple valve disorder	1834	0.06%
Idiopathic Intracranial Hypertension	1823	0.06%

For peer review only

Hypertrophic Cardiomyopathy	1779	0.06%
Oesophageal varices	1716	0.06%
Plasma Cell Malignancy	1610	0.05%
Scleroderma	1566	0.05%
Pericardial Effusion	1509	0.05%
Myasthenia gravis	1407	0.05%
Primary pulmonary hypertension	1345	0.04%
Sick sinus syndrome	1231	0.04%
Aplastic anaemias	1172	0.04%
Primary Malignancy: Brain	1131	0.04%
Immunodeficiencies	1071	0.03%
Cystic Fibrosis	985	0.03%
Primary Malignancy: Oesophageal	955	0.03%
Myelodysplastic Syndrome	927	0.03%
Portal hypertension	919	0.03%
Sickle Cell Disease	887	0.03%
Secondary pulmonary hypertension	824	0.03%
Angiodysplasia of colon	777	0.02%
Primary Malignancy: Bone	741	0.02%
Primary Malignancy: Stomach	694	0.02%
Hepatic failure	632	0.02%
Secondary Malignancy: Lymph Nodes	565	0.02%
Secondary Malignancy: Liver	491	0.02%
Tubulo-interstitial nephritis	365	0.01%
Motor neurone disease	347	0.01%
Primary Malignancy: Pancreas	302	0.01%
Enteropathic arthropathy	291	0.01%
Primary Malignancy: Liver	233	0.01%
Secondary Malignancy: Lung	223	0.01%
Secondary Malignancy: Bone	187	0.01%
Primary Malignancy: Biliary Tract	129	<0.01%
Secondary Malignancy: Brain	50	<0.01%
Secondary Malignancy: Peritoneum	24	<0.01%
Secondary Malignancy: Bowel	11	<0.01%
Secondary Malignancy: Adrenal Gland	*	<0.01%
Primary Malignancy: Multiple Sites	*	<0.01%
Primary Malignancy: Mesothelioma	*	<0.01%
Secondary Malignancy: Pleura	*	<0.01%

* diseases with frequency <10 suppressed as small counts

For peer review only

Table A3: characteristics of the 3,060,391 ineligible patients with no incident diseases over the study period

Patient characteristic	Total	Percent
**Age (years)**		
18-40	1476341	48.2%
40-49	689779	22.5%
50-59	435517	14.2%
60-69	291093	9.5%
70-79	129375	4.2%
80+	38286	1.3%
**Gender**		
Female	1357049	44.3%
Male	1703284	55.7%
Indeterminate	58	0.0%
**Total**	**3060391**	

For peer review only

Table A4: distribution of yearly codes over the whole follow-up period for each condition, ordered by median

Disease	5th centile	Median	95th centile	Mean	Standard deviation
Diabetes Mellitus_other or not specified	0.00	2.99	6.88	3.08	2.22
Polymyalgia Rheumatica	0.00	1.05	6.32	1.82	2.29
Motor neurone disease	0.00	0.95	12.15	2.86	5.41
Dementia	0.00	0.93	4.36	1.39	1.80
Type 2 Diabetes Mellitus	0.00	0.89	4.59	1.41	1.73
Type 1 Diabetes Mellitus	0.00	0.88	6.31	1.71	2.41
Depression	0.00	0.83	4.54	1.36	1.76
COPD	0.00	0.77	3.77	1.17	1.43
Heart failure	0.00	0.73	5.48	1.46	2.21
Rheumatoid Arthritis	0.00	0.70	5.50	1.43	2.23
Primary Malignancy_Mesothelioma	0.00	0.67	9.16	1.78	3.18
Primary Malignancy_Pancreas	0.00	0.67	13.41	2.63	5.12
Primary Malignancy_Brain	0.00	0.66	10.60	2.15	3.96
Primary Malignancy_Oesophageal	0.00	0.64	10.86	2.44	4.95
Myasthenia gravis	0.00	0.62	5.61	1.48	2.66
Multiple sclerosis	0.00	0.59	5.63	1.40	2.41
Parkinson's disease	0.00	0.59	4.52	1.20	1.77
Vitamin B12 deficiency anaemia	0.00	0.56	4.60	1.24	1.67
Bipolar affective disorder and mania	0.00	0.56	4.99	1.30	2.15
Plasma Cell Malignancy	0.00	0.54	10.32	2.15	4.67
Hypertension	0.00	0.54	2.95	0.88	1.12
Atrial Fibrillation	0.00	0.51	3.47	0.97	1.47
Primary Malignancy_Prostate	0.00	0.51	6.11	1.46	2.48
Intellectual disability	0.00	0.49	5.19	1.47	1.91
Primary Malignancy_Lung	0.00	0.45	8.17	1.73	3.55
Primary Malignancy_Biliary Tract	0.00	0.45	8.96	1.89	4.73
Giant Cell arteritis	0.00	0.44	5.73	1.36	2.47
Crohn's disease	0.00	0.42	5.41	1.24	2.32
Primary Malignancy_Breast	0.00	0.39	5.25	1.21	2.47
Hodgkin Lymphoma	0.00	0.38	5.41	1.24	2.55
Ulcerative colitis	0.00	0.38	4.27	1.00	1.87
Primary Malignancy_Oropharyngeal	0.00	0.37	6.84	1.44	2.95
Non-Hodgkin Lymphoma	0.00	0.37	5.52	1.22	2.53
Leukaemia	0.00	0.37	5.19	1.17	2.58
Secondary Malignancy_Brain	0.00	0.37	7.68	1.45	2.74
Stroke_not otherwise specified	0.00	0.34	2.11	0.59	0.89
Idiopathic Intracranial Hypertension	0.00	0.34	3.81	0.92	1.76
Thyroid Disease	0.00	0.33	2.56	0.68	1.16
Asthma	0.00	0.32	2.33	0.63	0.99
Primary Malignancy_Stomach	0.00	0.32	6.93	1.45	3.30
Chronic primary pain	0.00	0.32	3.23	0.79	1.34
Coronary Heart Disease (not otherwise specified)	0.00	0.31	2.02	0.56	0.85
Epilepsy	0.00	0.31	3.66	0.92	1.95
Psoriatic Arthritis	0.00	0.30	3.68	0.87	1.63

For peer review only

Chronic Fatigue Syndrome	0.00	0.29	3.22	0.76	1.31
Primary Malignancy_Bowel	0.00	0.29	5.25	1.15	2.88
Anxiety disorders	0.00	0.29	2.99	0.73	1.29
Primary Malignancy_Thyroid	0.00	0.28	4.05	0.88	1.76
Personality disorders	0.00	0.28	4.35	0.99	2.05
Schizophrenia	0.00	0.27	3.36	0.78	1.52
Primary Malignancy_Cervix	0.00	0.27	5.26	1.17	2.77
Autoimmune liver disease	0.00	0.26	3.63	0.85	1.82
Myelodysplastic Syndrome	0.00	0.26	4.88	1.15	2.95
Bronchiectasis	0.00	0.24	3.03	0.70	1.31
Hyperkinetic disorders	0.00	0.24	3.11	0.72	1.34
Primary Malignancy_Ovary	0.00	0.24	6.15	1.24	2.87
Primary Malignancy_Liver	0.00	0.23	3.64	0.95	2.99
Coeliac disease	0.00	0.23	2.13	0.52	0.85
Lupus Erythematosus	0.00	0.22	3.52	0.83	1.87
Myocardial Infarction	0.00	0.21	2.44	0.58	1.04
Primary Malignancy_Bone	0.00	0.21	4.03	0.97	3.29
Secondary Malignancy_other	0.00	0.21	5.92	1.18	2.65
Peripheral Vascular Disease	0.00	0.20	2.73	0.75	2.53
Ankylosing spondylitis	0.00	0.20	3.00	0.69	1.47
Primary Malignancy_Bladder	0.00	0.20	4.38	0.90	2.05
Primary Malignancy_Testis	0.00	0.20	3.58	0.81	1.50
Sarcoidosis	0.00	0.19	3.36	0.72	1.53
Abdominal Hernia	0.00	0.19	1.55	0.40	0.68
Secondary Malignancy_Peritoneum	0.00	0.19	4.21	1.30	3.31
Scleroderma	0.00	0.19	3.00	0.71	1.88
Primary Malignancy_Melanoma	0.00	0.18	3.06	0.67	1.71
Gout	0.00	0.17	1.74	0.43	0.73
Barrett's oesophagus	0.00	0.16	1.40	0.35	0.57
Glomerulonephritis	0.00	0.16	3.26	0.74	1.69
Osteoporosis	0.00	0.15	1.52	0.38	0.65
Primary Malignancy_Uterus	0.00	0.15	3.90	0.81	2.16
Cirrhosis	0.00	0.15	2.88	0.63	1.40
Diabetic Eye Disease	0.00	0.15	1.61	0.40	0.68
Intracerebral haemorrhage	0.00	0.15	2.58	0.56	1.10
Primary Malignancy_Kidney	0.00	0.14	2.93	0.66	1.67
Dilated cardiomyopathy	0.00	0.14	1.99	0.46	0.93
Eating Disorders	0.00	0.14	4.03	0.84	2.38
Abdominal Aortic Aneurysm	0.00	0.00	1.35	0.26	0.58
Acne	0.00	0.00	1.26	0.30	0.50
Alcohol Misuse	0.00	0.00	0.94	0.20	0.66
Alcoholic liver disease	0.00	0.00	1.90	0.42	1.09
Allergic and chronic rhinitis	0.00	0.00	0.56	0.10	0.27
Alopecia areata	0.00	0.00	0.87	0.17	0.45
Anaemia_other	0.00	0.00	1.49	0.33	0.78
Angiodysplasia of colon	0.00	0.00	0.87	0.17	0.49
Anterior and Intermediate Uveitis	0.00	0.00	1.18	0.25	0.66
Aplastic anaemias	0.00	0.00	2.19	0.47	1.42
Asbestosis	0.00	0.00	0.96	0.20	0.65
Atrioventricular blocks	0.00	0.00	0.64	0.11	0.33

For peer review only

For peer review only - http://bmjopen.bmj.com/site/about/guidelines.xhtml

Autism and Asperger's syndrome	0.00	0.00	1.10	0.25	0.58
Autonomic Neuropathy	0.00	0.00	2.46	0.47	1.34
Benign Prostatic Hyperplasia	0.00	0.00	1.08	0.25	0.50
Benign essential tremor	0.00	0.00	1.11	0.22	0.53
Cardiomyopathy_other	0.00	0.00	1.94	0.41	0.90
Cataract	0.00	0.00	1.16	0.27	0.50
Cerebral Palsy	0.00	0.00	0.73	0.16	0.48
Chronic Cystitis	0.00	0.00	1.88	0.37	1.03
Chronic Kidney Disease	0.00	0.00	1.16	0.26	0.65
Chronic sinusitis	0.00	0.00	0.72	0.13	0.39
Chronic viral hepatitis	0.00	0.00	1.89	0.40	0.90
Collapsed vertebra	0.00	0.00	1.64	0.34	0.77
Congenital Septal Defect	0.00	0.00	1.21	0.24	0.62
Cystic Fibrosis	0.00	0.00	2.21	0.31	1.00
Dermatitis	0.00	0.00	0.76	0.15	0.43
Diabetic Neuropathy	0.00	0.00	1.62	0.38	1.44
Diaphragmatic hernia	0.00	0.00	0.81	0.17	0.38
Diverticular Disease	0.00	0.00	0.96	0.20	0.51
Down's syndrome	0.00	0.00	0.48	0.10	0.19
Dysmenorrhoea	0.00	0.00	0.78	0.15	0.38
Endometrial hyperplasia and hypertrophy	0.00	0.00	0.90	0.17	0.57
Endometriosis	0.00	0.00	2.08	0.44	1.06
Enteropathic arthropathy	0.00	0.00	1.28	0.38	0.99
Enthesopathy and synovial disorder	0.00	0.00	0.86	0.18	0.43
Fatty Liver	0.00	0.00	0.75	0.14	0.34
Fibromatosis	0.00	0.00	0.85	0.17	0.39
Folate deficiency anaemia	0.00	0.00	0.52	0.09	0.25
Gastritis and duodenitis	0.00	0.00	0.73	0.14	0.39
Gastro-oesophageal reflux disease	0.00	0.00	0.88	0.18	0.43
Glaucoma	0.00	0.00	1.46	0.31	0.62
HIV	0.00	0.00	2.07	0.41	0.92
Hearing loss	0.00	0.00	0.77	0.16	0.34
Hepatic failure	0.00	0.00	2.22	0.46	1.07
Hidradenitis suppurativa	0.00	0.00	1.92	0.43	1.11
Hyperparathyroidism	0.00	0.00	1.84	0.41	0.84
Hypersplenism	0.00	0.00	0.99	0.21	0.58
Hypertrophic Cardiomyopathy	0.00	0.00	2.23	0.49	1.00
Hypertrophic Nasal Turbinates	0.00	0.00	0.28	0.04	0.16
Hyposplenism	0.00	0.00	1.50	0.34	0.71
Immunodeficiencies	0.00	0.00	1.62	0.36	1.11
Intervertebral disc disorders	0.00	0.00	1.75	0.36	0.91
Irritable bowel syndrome	0.00	0.00	0.66	0.13	0.32
Ischaemic stroke	0.00	0.00	2.03	0.46	0.99
Left bundle branch block	0.00	0.00	0.77	0.15	0.39
Macular degeneration	0.00	0.00	1.16	0.25	0.71
Meniere's Disease	0.00	0.00	1.58	0.33	0.77
Migraine	0.00	0.00	1.21	0.25	0.61
Multiple valve disorder	0.00	0.00	0.49	0.09	0.33
Neuropathic Bladder	0.00	0.00	0.74	0.15	0.36

For peer review only

Nonrheumatic aortic valve disorders	0.00	0.00	1.42	0.31	0.68
Nonrheumatic mitral valve disorders	0.00	0.00	0.84	0.16	0.52
Obesity	0.00	0.00	0.71	0.15	0.44
Obsessive-compulsive disorder	0.00	0.00	2.55	0.56	1.21
Obstructive and reflux uropathy	0.00	0.00	1.10	0.23	0.63
Oesophageal varices	0.00	0.00	1.62	0.38	0.74
Osteoarthritis (excl spine)	0.00	0.00	1.53	0.34	0.70
Other haemolytic anaemias	0.00	0.00	3.09	0.62	1.64
Pancreatitis	0.00	0.00	2.00	0.44	1.09
Pericardial Effusion	0.00	0.00	1.12	0.21	0.56
Peripheral Neuropathy	0.00	0.00	1.22	0.26	0.81
Pleural effusion	0.00	0.00	1.55	0.32	0.90
Pleural plaque	0.00	0.00	0.74	0.14	0.48
Polycystic ovarian syndrome	0.00	0.00	0.86	0.20	0.34
Polycythaemia vera	0.00	0.00	2.49	0.54	1.30
Portal hypertension	0.00	0.00	0.91	0.18	0.46
Posterior Uveitis	0.00	0.00	1.46	0.33	1.02
Primary Malignancy_Multiple Sites	0.00	0.00	0.00	0.00	0.00
Primary Malignancy_Skin	0.00	0.00	1.30	0.31	0.78
Primary Malignancy_other	0.00	0.00	4.42	0.90	2.44
Primary Thrombocytopaenia	0.00	0.00	2.41	0.59	1.96
Primary pulmonary hypertension	0.00	0.00	1.62	0.32	1.00
Psoriasis	0.00	0.00	1.44	0.32	0.75
Pulmonary Fibrosis	0.00	0.00	2.38	0.53	1.34
Raynaud's syndrome	0.00	0.00	0.85	0.16	0.45
Retinal vascular occlusions	0.00	0.00	1.93	0.42	0.93
Rheumatic Valve Disorder	0.00	0.00	0.70	0.13	0.41
Right bundle branch block combinations	0.00	0.00	0.47	0.08	0.25
Rosacea	0.00	0.00	0.93	0.20	0.41
Scleritis and episcleritis	0.00	0.00	0.70	0.13	0.49
Seborrheic dermatitis	0.00	0.00	0.61	0.11	0.31
Secondary Malignancy_Adrenal Gland	0.00	0.00	1.68	0.42	1.01
Secondary Malignancy_Bone	0.00	0.00	4.78	0.93	2.34
Secondary Malignancy_Bowel	0.00	0.00	6.36	1.41	2.42
Secondary Malignancy_Liver	0.00	0.00	4.82	0.91	2.26
Secondary Malignancy_Lung	0.00	0.00	6.04	1.10	2.27
Secondary Malignancy_Lymph Nodes	0.00	0.00	2.40	0.40	1.31
Secondary Malignancy_Pleura	0.00	0.00	5.69	0.94	2.50
Secondary Thrombocytopaenia	0.00	0.00	0.89	0.19	0.48
Secondary polycythaemia	0.00	0.00	1.64	0.32	0.78
Secondary pulmonary hypertension	0.00	0.00	1.29	0.27	0.83
Sick sinus syndrome	0.00	0.00	0.79	0.14	0.40
Sickle Cell Disease	0.00	0.00	0.98	0.29	1.07
Sjogren's Syndrome	0.00	0.00	1.48	0.31	0.68
Sleep apnoea	0.00	0.00	0.92	0.19	0.43
Spina bifida	0.00	0.00	0.48	0.11	0.44
Spinal stenosis	0.00	0.00	2.34	0.50	1.06
Spondylolisthesis	0.00	0.00	1.22	0.23	0.63
Spondylosis	0.00	0.00	1.01	0.21	0.57
Stable Angina	0.00	0.00	1.62	0.37	0.78

For peer review only - http://bmjopen.bmj.com/site/about/guidelines.xhtml

Subarachnoid haemorrhage	0.00	0.00	2.41	0.51	1.05
Substance Misuse	0.00	0.00	1.42	0.32	1.34
Supraventricular tachycardia	0.00	0.00	1.55	0.35	0.78
Thalassaemia	0.00	0.00	0.31	0.05	0.19
Thrombophilia	0.00	0.00	0.75	0.15	0.53
Tinnitus	0.00	0.00	0.85	0.17	0.43
Transient ischaemic attack	0.00	0.00	1.56	0.35	0.70
Trigeminal neuralgia	0.00	0.00	2.16	0.47	1.05
Tubulo-interstitial nephritis	0.00	0.00	2.70	0.50	1.23
Unstable Angina	0.00	0.00	1.17	0.23	0.58
Urinary Incontinence	0.00	0.00	0.87	0.18	0.38
Venous thromboembolic disease (Excl PE)	0.00	0.00	1.85	0.41	1.05
Ventricular tachycardia	0.00	0.00	1.64	0.32	0.75
Visual impairment and blindness	0.00	0.00	0.73	0.13	0.31
Vitiligo	0.00	0.00	0.73	0.14	0.32

For peer review only

Figure A1: Boxplots of observed and expected mean yearly codes at a GP practice level for QOF conditions in year 1 (A) and year 2 (B) and non-QOF conditions in year 1 (C) and year 2 (D) following diagnosis

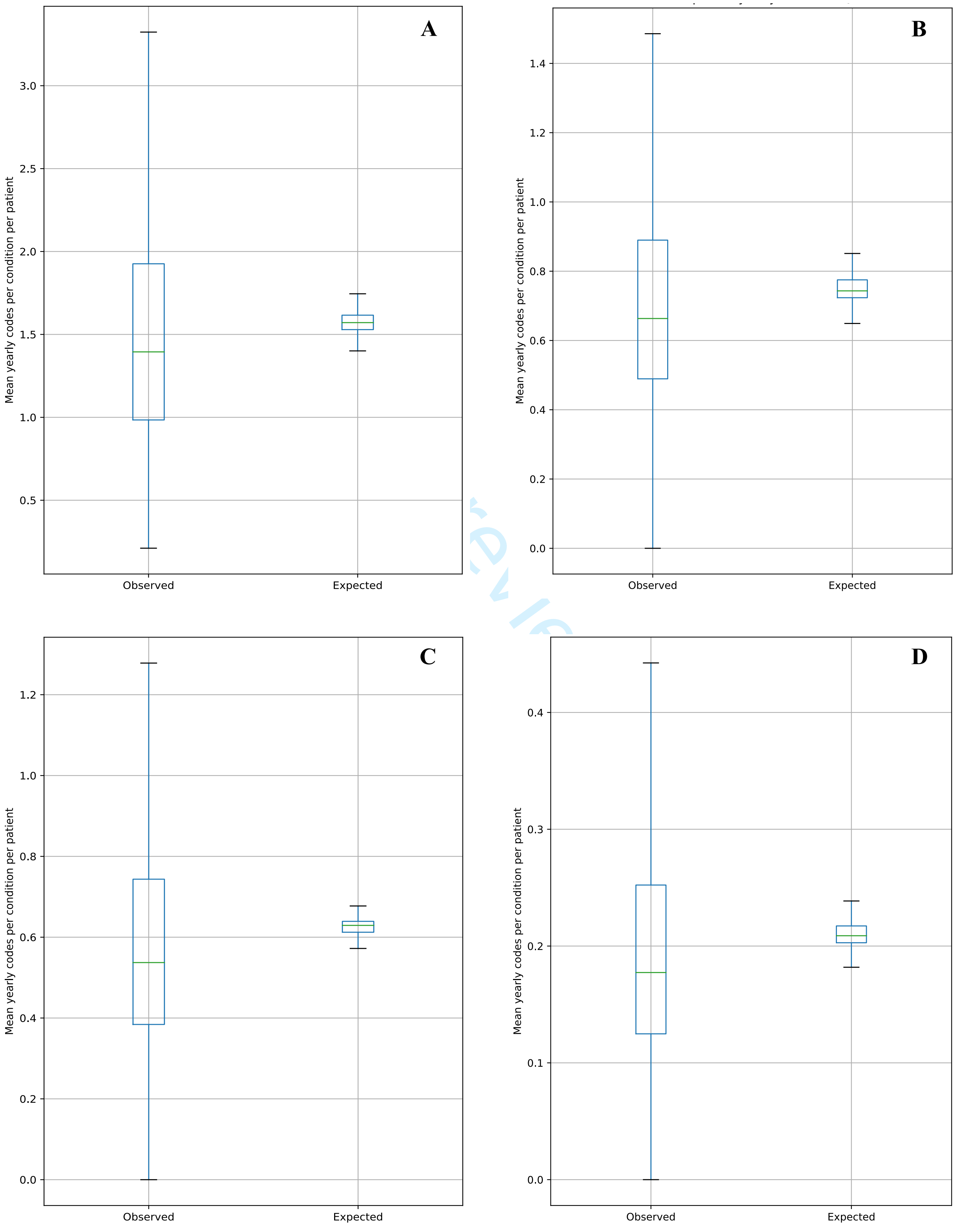

For peer review only - http://bmjopen.bmj.com/site/about/guidelines.xhtml

Figure A2: ratio of mean yearly codes in year 1 following diagnosis to subsequent years for QOF conditions

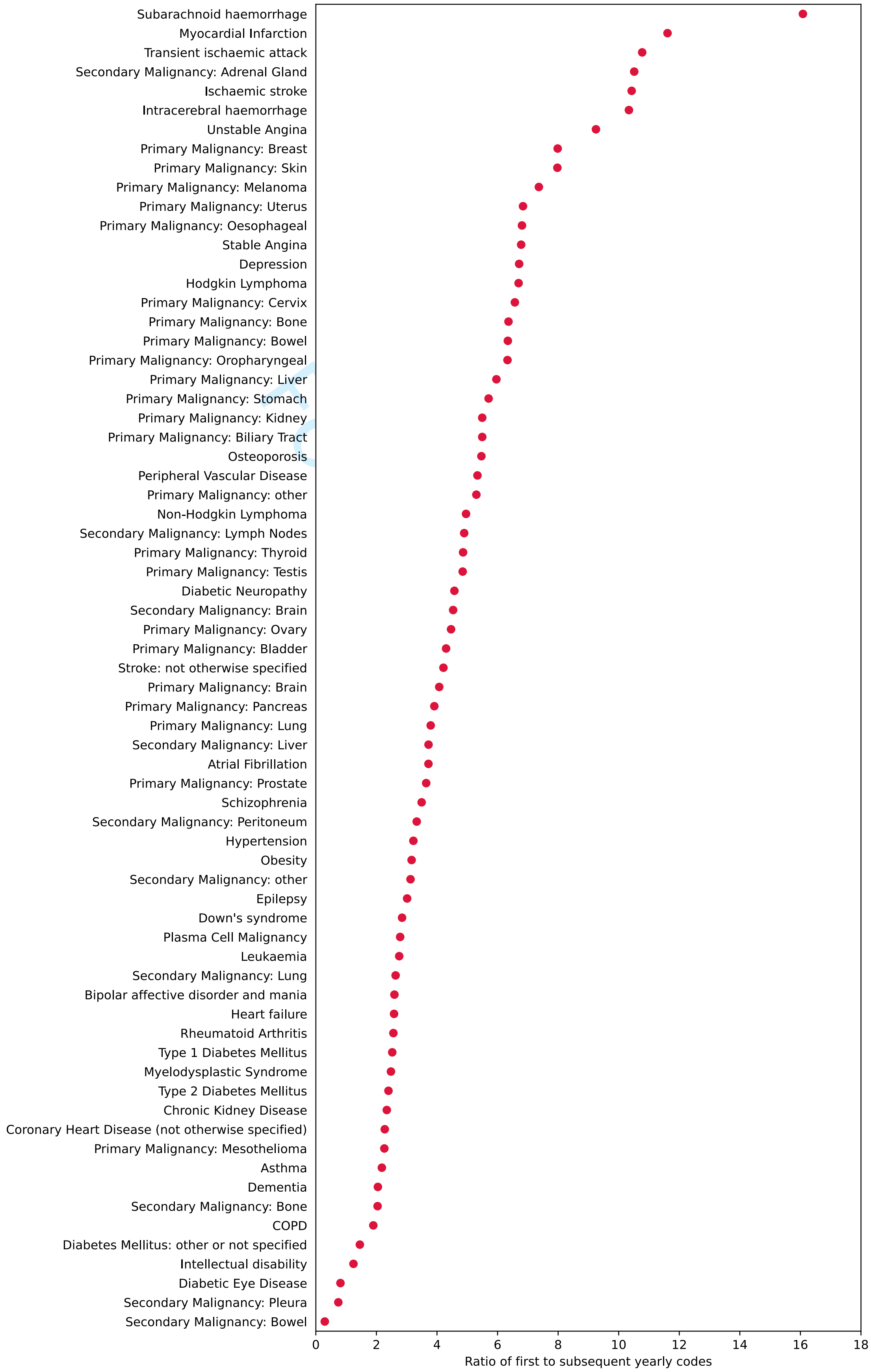

Figure A3: Ratio of mean yearly codes in year 1 following diagnosis to subsequent years for non-QOF conditions

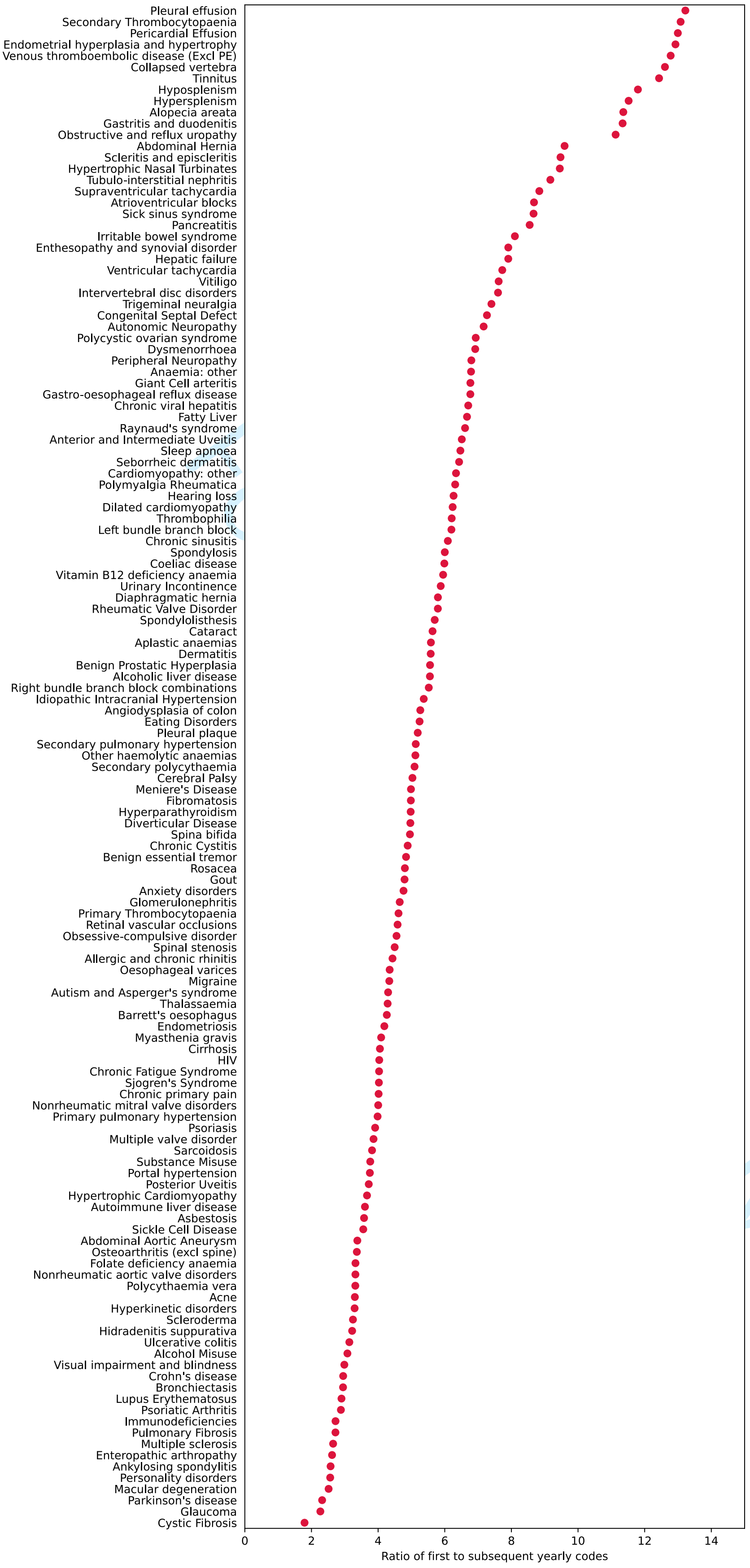

19

Figure A4: Boxplots of the distribution of mean yearly codes following diagnosis for newly diagnosed conditions by GP practice stratified by inclusion in QOF

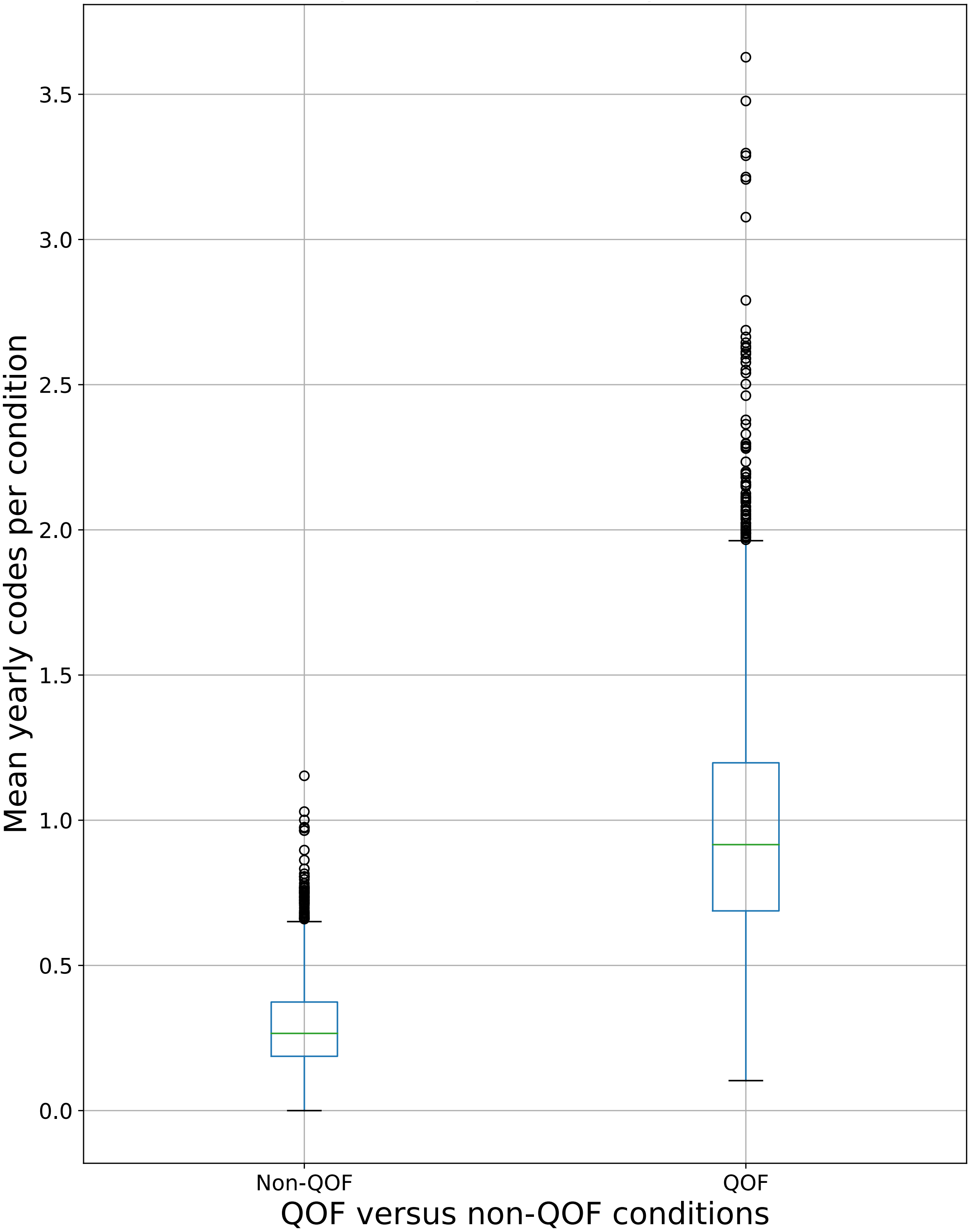

Figure A5: boxplots of mean yearly codes at a GP practice level by practice level Index of Multiple Deprivation decile (1 = most deprived, 10 = least deprived)

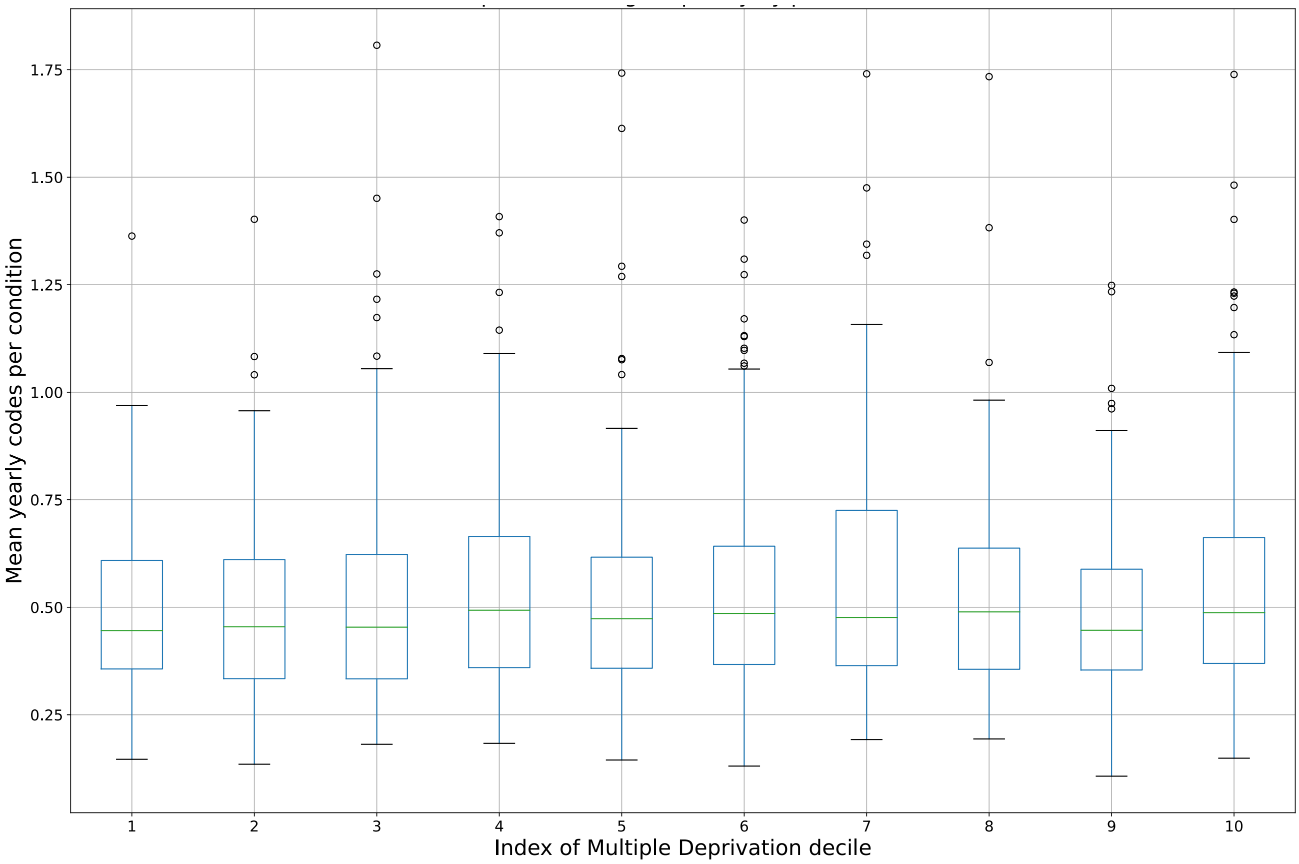

Footnote: combines QOF and non-QOF conditions

For peer review only

Table A5: Associations of rate of codes in year one following diagnosis for conditions included in QOF (N=1730485)

Variable	Primary analysis				Sensitivity analysis including consultation number			
			95% CI				95% CI	
	IRR	P-value	Lower	Upper	IRR	P-value	Lower	Upper
**Age category (years)**								
Under 40	1.33	0.00	1.32	1.34	1.30	0.00	1.29	1.31
40-49	1.15	0.00	1.14	1.15	1.14	0.00	1.13	1.15
50-59	1.08	0.00	1.07	1.08	1.07	0.00	1.07	1.08
60-69 (reference)	-	-	-	-	-	-	-	-
70-79	0.96	0.00	0.95	0.96	0.94	0.00	0.93	0.95
80 or more	0.91	0.00	0.90	0.92	0.88	0.00	0.87	0.88
**Sex**								
Female (reference)	-	-	-	-	-	-	-	-
Male	1.03	0.00	1.02	1.03	1.10	0.00	1.10	1.11
**Ethnicity category**								
White (reference)	-	-	-	-	-	-	-	-
South Asian	0.96	0.00	0.95	0.97	0.92	0.00	0.91	0.93
Black	0.94	0.00	0.93	0.95	0.94	0.00	0.93	0.95
Other	0.95	0.00	0.93	0.97	0.96	0.00	0.94	0.98
Mixed	0.98	0.03	0.95	1.00	0.97	0.00	0.95	0.99
Missing	0.98	0.00	0.97	0.99	1.01	0.00	1.00	1.02
**IMD decile**								
1 (most deprived)	-	-	-	-	-	-	-	-
2	1.01	0.19	1.00	1.01	1.00	0.95	0.99	1.01
3	1.02	0.00	1.01	1.03	1.01	0.08	1.00	1.02
4	1.02	0.00	1.01	1.03	1.01	0.01	1.00	1.02
5	1.02	0.00	1.01	1.03	1.01	0.06	1.00	1.02
6	1.03	0.00	1.02	1.04	1.01	0.02	1.00	1.02
7	1.04	0.00	1.03	1.05	1.02	0.00	1.01	1.03
8	1.04	0.00	1.03	1.05	1.01	0.01	1.00	1.02
9	1.05	0.00	1.04	1.06	1.02	0.00	1.01	1.03
10 (least deprived)	1.05	0.00	1.04	1.06	1.01	0.06	1.00	1.02
**Number of QOF diseases**								
0 (reference)	-	-	-	-	-	-	-	-
1	0.90	0.00	0.90	0.91	0.87	0.00	0.86	0.87
2	0.80	0.00	0.80	0.81	0.75	0.00	0.75	0.76
3	0.71	0.00	0.70	0.71	0.66	0.00	0.65	0.66
4 or more	0.63	0.00	0.62	0.63	0.56	0.00	0.55	0.56
**Number of non-QOF diseases**								
0 (reference)	-	-	-	-	-	-	-	-
1	1.16	0.00	1.16	1.17	1.08	0.00	1.07	1.08
2	1.13	0.00	1.12	1.14	1.02	0.00	1.01	1.02
3	1.12	0.00	1.11	1.12	0.97	0.00	0.96	0.98
4 or more	1.13	0.00	1.12	1.13	0.90	0.00	0.89	0.90
**Calendar year of diagnosis**								
2015 (reference)	-	-	-	-	-	-	-	-
2016	1.00	0.89	0.99	1.01	1.02	0.00	1.02	1.03
2017	1.00	0.34	1.00	1.01	1.05	0.00	1.04	1.05
2018	1.00	0.18	0.99	1.00	1.06	0.00	1.06	1.07
2019	0.95	0.00	0.94	0.96	1.04	0.00	1.04	1.05
**Average number of consultations in year 1**								
Less than 1 (reference)	-	-	-	-	-	-	-	-
1-2	-	-	-	-	1.62	0.00	1.60	1.63
3-4	-	-	-	-	2.21	0.00	2.19	2.23
5-9	-	-	-	-	2.87	0.00	2.84	2.89
10 or more	-	-	-	-	3.75	0.00	3.71	3.79

From negative binomial regression models, including practice-level fixed effects (not shown)

For peer review only - http://bmjopen.bmj.com/site/about/guidelines.xhtml

Table A6: Associations of rate of codes in year two following diagnosis for conditions included in QOF (N=1714684)

	Primary analysis				Sensitivity analysis including consultation number			
			95% CI				95% CI	
**Variable**	**IRR**	**P-value**	**Lower**	**Upper**	**IRR**	**P-value**	**Lower**	**Upper**
**Age category (years)**								
Under 40	0.87	0.00	0.86	0.87	0.86	0.00	0.86	0.87
40-49	1.01	0.03	1.00	1.02	1.01	0.22	1.00	1.01
50-59	1.03	0.00	1.02	1.04	1.03	0.00	1.02	1.04
60-69 (reference)	-	-	-	-	-	-	-	-
70-79	0.95	0.00	0.94	0.96	0.93	0.00	0.93	0.94
80 or more	0.89	0.00	0.88	0.90	0.86	0.00	0.85	0.87
**Sex**								
Female (reference)	-	-	-	-	-	-	-	-
Male	1.11	0.00	1.11	1.12	1.18	0.00	1.17	1.18
**Ethnicity category**								
White (reference)	-	-	-	-	-	-	-	-
South Asian	1.26	0.00	1.25	1.28	1.22	0.00	1.20	1.23
Black	1.17	0.00	1.16	1.19	1.17	0.00	1.15	1.19
Other	1.13	0.00	1.10	1.16	1.14	0.00	1.11	1.17
Mixed	1.12	0.00	1.08	1.15	1.11	0.00	1.07	1.14
Missing	0.89	0.00	0.88	0.90	0.93	0.00	0.92	0.93
**IMD decile**								
1 (most deprived)	-	-	-	-	-	-	-	-
2	1.02	0.00	1.01	1.04	1.02	0.00	1.01	1.03
3	1.03	0.00	1.02	1.05	1.03	0.00	1.02	1.04
4	1.05	0.00	1.03	1.06	1.04	0.00	1.03	1.05
5	1.05	0.00	1.04	1.07	1.04	0.00	1.03	1.06
6	1.06	0.00	1.05	1.07	1.05	0.00	1.04	1.07
7	1.08	0.00	1.06	1.09	1.06	0.00	1.05	1.08
8	1.09	0.00	1.07	1.10	1.07	0.00	1.06	1.08
9	1.11	0.00	1.10	1.13	1.09	0.00	1.08	1.11
10 (least deprived)	1.14	0.00	1.12	1.15	1.11	0.00	1.09	1.12
**Number of QOF diseases**								
0 (reference)	-	-	-	-	-	-	-	-
1	1.05	0.00	1.04	1.06	1.00	0.79	0.99	1.01
2	1.07	0.00	1.06	1.08	0.99	0.05	0.98	1.00
3	0.99	0.15	0.98	1.00	0.91	0.00	0.90	0.92
4 or more	0.87	0.00	0.86	0.88	0.77	0.00	0.76	0.78
**Number of non-QOF diseases**								
0 (reference)	-	-	-	-	-	-	-	-
1	1.05	0.00	1.04	1.06	0.99	0.11	0.98	1.00
2	1.04	0.00	1.03	1.05	0.96	0.00	0.95	0.97
3	1.04	0.00	1.03	1.05	0.93	0.00	0.92	0.94
4 or more	1.05	0.00	1.04	1.06	0.88	0.00	0.87	0.89
**Calendar year of diagnosis**								
2015 (reference)	-	-	-	-	-	-	-	-
2016	1.00	0.45	0.99	1.01	1.02	0.00	1.01	1.03
2017	0.99	0.00	0.98	0.99	1.02	0.00	1.01	1.03
2018	0.91	0.00	0.90	0.92	0.96	0.00	0.95	0.97
2019	0.79	0.00	0.79	0.80	0.86	0.00	0.86	0.87
**Average number of consultations in year 1**								
Less than 1 (reference)					-	-	-	-
2					1.53	0.00	1.52	1.55
3-4					1.87	0.00	1.85	1.89
5-9					2.17	0.00	2.15	2.20
10 or more					2.59	0.00	2.57	2.62

From negative binomial regression models, including practice-level fixed effects (not shown)

For peer review only - http://bmjopen.bmj.com/site/about/guidelines.xhtml

Table A7: Associations of rate of codes in year one following diagnosis for conditions not included in QOF (N=3617348)

	Primary analysis				Sensitivity analysis including consultation number			
			95% CI				95% CI	
**Variable**	**IRR**	**P-value**	**Lower**	**Upper**	**IRR**	**P-value**	**Lower**	**Upper**
**Age category (years)**								
Under 40	1.10	0.00	1.10	1.11	1.09	0.00	1.08	1.10
40-49	1.01	0.00	1.00	1.02	1.02	0.00	1.01	1.03
50-59	0.98	0.00	0.98	0.99	0.99	0.09	0.99	1.00
60-69 (reference)	-	-	-	-	-	-	-	-
70-79	1.05	0.00	1.05	1.06	1.03	0.00	1.02	1.03
80 or more	1.02	0.00	1.02	1.03	0.98	0.00	0.97	0.99
**Sex**								
Female (reference)	-	-	-	-	-	-	-	-
Male	1.00	0.03	0.99	1.00	1.13	0.00	1.12	1.13
**Ethnicity category**								
White (reference)	-	-	-	-	-	-	-	-
South Asian	0.95	0.00	0.94	0.96	0.89	0.00	0.88	0.90
Black	0.89	0.00	0.88	0.90	0.86	0.00	0.85	0.87
Other	0.90	0.00	0.88	0.91	0.89	0.00	0.88	0.91
Mixed	0.95	0.00	0.93	0.97	0.92	0.00	0.91	0.94
Missing	0.99	0.14	0.99	1.00	1.06	0.00	1.05	1.06
**IMD decile**								
1 (most deprived)	-	-	-	-	-	-	-	-
2	1.00	0.86	0.99	1.01	0.99	0.06	0.98	1.00
3	1.01	0.01	1.00	1.02	1.00	0.82	0.99	1.01
4	1.02	0.00	1.01	1.03	1.00	0.42	0.99	1.01
5	1.02	0.00	1.01	1.03	1.00	0.86	0.99	1.01
6	1.03	0.00	1.02	1.04	0.99	0.26	0.99	1.00
7	1.03	0.00	1.02	1.04	0.99	0.08	0.98	1.00
8	1.04	0.00	1.03	1.06	0.99	0.15	0.98	1.00
9	1.06	0.00	1.05	1.07	0.99	0.19	0.98	1.00
10 (least deprived)	1.06	0.00	1.05	1.07	0.98	0.00	0.97	0.99
**Number of QOF diseases**								
0 (reference)	-	-	-	-	-	-	-	-
1	1.16	0.00	1.15	1.16	1.02	0.00	1.02	1.03
2	1.09	0.00	1.08	1.09	0.94	0.00	0.93	0.94
3	1.06	0.00	1.05	1.07	0.90	0.00	0.89	0.91
4 or more	1.04	0.00	1.03	1.04	0.85	0.00	0.84	0.85
**Number of non-QOF diseases**								
0 (reference)	-	-	-	-	-	-	-	-
1	1.02	0.00	1.01	1.02	0.93	0.00	0.92	0.94
2	1.02	0.00	1.02	1.03	0.87	0.00	0.87	0.88
3	1.04	0.00	1.03	1.05	0.83	0.00	0.82	0.84
4 or more	1.06	0.00	1.06	1.07	0.74	0.00	0.74	0.75
**Calendar year of diagnosis**								
2015 (reference)	-	-	-	-	-	-	-	-
2016	1.00	0.55	0.99	1.00	1.03	0.00	1.02	1.03
2017	0.99	0.00	0.99	1.00	1.05	0.00	1.04	1.05
2018	0.99	0.00	0.98	0.99	1.07	0.00	1.06	1.07
2019	0.94	0.00	0.94	0.95	1.06	0.00	1.06	1.07
**Average number of consultations in year 1**								
Less than 1 (reference)					-	-	-	-
1-2					2.38	0.00	2.36	2.40
3-4					3.49	0.00	3.45	3.52
5-9					4.67	0.00	4.62	4.71
10 or more					6.37	0.00	6.31	6.44

From negative binomial regression models, including practice-level fixed effects (not shown)

For peer review only - http://bmjopen.bmj.com/site/about/guidelines.xhtml

Table A8: Associations of rate of codes in year two following diagnosis for conditions not included in QOF (N=3593019)

	Primary analysis				Sensitivity analysis including consultation number			
			95% CI				95% CI	
**Variable**	**IRR**	**P-value**	**Lower**	**Upper**	**IRR**	**P-value**	**Lower**	**Upper**
**Age category (years)**								
Under 40	1.27	0.00	1.26	1.28	1.26	0.00	1.25	1.28
40-49	1.03	0.00	1.02	1.04	1.03	0.00	1.02	1.04
50-59	0.98	0.00	0.97	0.99	0.99	0.10	0.98	1.00
60-69 (reference)	-	-	-	-	-	-	-	-
70-79	1.06	0.00	1.05	1.07	1.03	0.00	1.02	1.04
80 or more	1.06	0.00	1.05	1.08	1.01	0.18	1.00	1.02
**Sex**								
Female (reference)	-	-	-	-	-	-	-	-
Male	0.93	0.00	0.93	0.94	1.08	0.00	1.07	1.09
**Ethnicity category**								
White (reference)	-	-	-	-	-	-	-	-
South Asian	0.99	0.17	0.97	1.00	0.92	0.00	0.91	0.94
Black	0.94	0.00	0.92	0.95	0.91	0.00	0.89	0.92
Other	0.88	0.00	0.86	0.91	0.89	0.00	0.86	0.92
Mixed	0.94	0.00	0.91	0.98	0.92	0.00	0.89	0.95
Missing	0.96	0.00	0.95	0.97	1.05	0.00	1.03	1.06
**IMD decile**								
1 (most deprived)	-	-	-	-	-	-	-	-
2	1.01	0.10	1.00	1.03	1.00	0.79	0.99	1.02
3	1.03	0.00	1.02	1.05	1.02	0.00	1.01	1.04
4	1.04	0.00	1.02	1.05	1.02	0.01	1.01	1.04
5	1.05	0.00	1.04	1.07	1.03	0.00	1.01	1.04
6	1.06	0.00	1.04	1.08	1.03	0.00	1.01	1.04
7	1.07	0.00	1.06	1.09	1.03	0.00	1.01	1.05
8	1.10	0.00	1.08	1.11	1.04	0.00	1.03	1.06
9	1.13	0.00	1.11	1.14	1.06	0.00	1.04	1.08
10 (least deprived)	1.14	0.00	1.12	1.16	1.06	0.00	1.04	1.08
**Number of QOF diseases**								
0 (reference)	-	-	-	-	-	-	-	-
1	1.19	0.00	1.18	1.21	1.05	0.00	1.04	1.06
2	1.15	0.00	1.14	1.16	0.98	0.00	0.97	0.99
3	1.13	0.00	1.12	1.15	0.95	0.00	0.94	0.96
4 or more	1.16	0.00	1.14	1.17	0.93	0.00	0.92	0.94
**Number of non-QOF diseases**								
0 (reference)	-	-	-	-	-	-	-	-
1	1.04	0.00	1.03	1.06	0.94	0.00	0.93	0.95
2	1.09	0.00	1.08	1.11	0.90	0.00	0.89	0.91
3	1.13	0.00	1.11	1.14	0.86	0.00	0.85	0.87
4 or more	1.21	0.00	1.20	1.23	0.80	0.00	0.79	0.81
**Calendar year of diagnosis**								
2015 (reference)	-	-	-	-	-	-	-	-
2016	1.00	0.56	0.99	1.01	1.03	0.00	1.02	1.04
2017	1.00	0.43	0.99	1.01	1.06	0.00	1.05	1.07
2018	0.91	0.00	0.90	0.92	1.01	0.01	1.00	1.02
2019	0.79	0.00	0.79	0.80	0.93	0.00	0.92	0.94
**Average number of consultations in year 1**								
Less than 1 (reference)					-	-	-	-
1-2					2.76	0.00	2.72	2.81
3-4					4.06	0.00	4.00	4.12
5-9					5.40	0.00	5.32	5.48
10 or more					7.35	0.00	7.24	7.47

From negative binomial regression models, including practice-level fixed effects (not shown)

For peer review only - http://bmjopen.bmj.com/site/about/guidelines.xhtml

References

1. Head, A. *et al.* Inequalities in incident and prevalent multimorbidity in England, 2004–19: a population-based, descriptive study. *The Lancet Healthy Longevity* **2**, e489–e497 (2021).
2. Kuan, V. *et al.* A chronological map of 308 physical and mental health conditions from 4 million individuals in the English National Health Service. *The Lancet Digital Health* **1**, e63–e77 (2019).
3. Bisquera, A. *et al.* Inequalities in developing multimorbidity over time: A population-based cohort study from an urban, multi-ethnic borough in the United Kingdom. *Lancet Reg Health Eur* **12**, 100247 (2021).
4. Ashworth, M. *et al.* Journey to multimorbidity: longitudinal analysis exploring cardiovascular risk factors and sociodemographic determinants in an urban setting. *BMJ Open* **9**, (2019).
5. NHS Health and Social Care Information Centre. National Quality and Outcomes Framework Statistics for England 2004/05. https://files.digital.nhs.uk/publicationimport/pub01xxx/pub01946/qof-eng-04-05-rep.pdf.
6. Health & Social Care Information Centre. Quality and Outcomes Framework – Prevalence, Achievements and Exceptions Report. England, 2013-14. https://files.digital.nhs.uk/publicationimport/pub15xxx/pub15751/qof-1314-report-v1.1.pdf.
7. Health & Social Care Information Centre. Quality and Outcomes Framework – Prevalence, Achievements and Exceptions Report, England, 2014-15. https://files.digital.nhs.uk/publicationimport/pub18xxx/pub18887/qof-1415-report%20v1.1.pdf (2015).

For peer review only

For peer review only - http://bmjopen.bmj.com/site/about/guidelines.xhtml

The RECORD statement – checklist of items, extended from the STROBE statement, that should be reported in observational studies using routinely collected health data.

	Item No.	STROBE items	Location in manuscript where items are reported	RECORD items	Location in manuscript where items are reported
**Title and abstract**					
	1	(a) Indicate the study's design with a commonly used term in the title or the abstract (b) Provide in the abstract an informative and balanced summary of what was done and what was found	p1-3	RECORD 1.1: The type of data used should be specified in the title or abstract. When possible, the name of the databases used should be included.<br><br>RECORD 1.2: If applicable, the geographic region and timeframe within which the study took place should be reported in the title or abstract.<br><br>RECORD 1.3: If linkage between databases was conducted for the study, this should be clearly stated in the title or abstract.	p1<br><br>p2<br><br>N/A
**Introduction**					
Background rationale	2	Explain the scientific background and rationale for the investigation being reported	p4-5		
Objectives	3	State specific objectives, including any prespecified hypotheses	p5		
**Methods**					
Study Design	4	Present key elements of study design early in the paper	p5		
Setting	5	Describe the setting, locations, and relevant dates, including periods of recruitment, exposure, follow-up, and data collection	p5		

For peer review only

Participants	6	*(a) Cohort study* - Give the eligibility criteria, and the sources and methods of selection of participants. Describe methods of follow-up<br>*Case-control study* - Give the eligibility criteria, and the sources and methods of case ascertainment and control selection. Give the rationale for the choice of cases and controls<br>*Cross-sectional study* - Give the eligibility criteria, and the sources and methods of selection of participants<br><br>*(b) Cohort study* - For matched studies, give matching criteria and number of exposed and unexposed<br>*Case-control study* - For matched studies, give matching criteria and the number of controls per case	p5 and appendix p2	RECORD 6.1: The methods of study population selection (such as codes or algorithms used to identify subjects) should be listed in detail. If this is not possible, an explanation should be provided.<br><br>RECORD 6.2: Any validation studies of the codes or algorithms used to select the population should be referenced. If validation was conducted for this study and not published elsewhere, detailed methods and results should be provided.<br><br>RECORD 6.3: If the study involved linkage of databases, consider use of a flow diagram or other graphical display to demonstrate the data linkage process, including the number of individuals with linked data at each stage.	p5
Variables	7	Clearly define all outcomes, exposures, predictors, potential confounders, and effect modifiers. Give diagnostic criteria, if applicable.	p5 and appendix p2-3	RECORD 7.1: A complete list of codes and algorithms used to classify exposures, outcomes, confounders, and effect modifiers should be provided. If these cannot be reported, an explanation should be provided.	
Data sources/ measurement	8	For each variable of interest, give sources of data and details of methods of assessment (measurement).<br>Describe comparability of assessment methods if there is more than one group	p5 and appendix p2-3		

For peer review only

Bias	9	Describe any efforts to address potential sources of bias	p6-7		
Study size	10	Explain how the study size was arrived at	p8		
Quantitative variables	11	Explain how quantitative variables were handled in the analyses. If applicable, describe which groupings were chosen, and why	P5-6		
Statistical methods	12	(a) Describe all statistical methods, including those used to control for confounding<br>(b) Describe any methods used to examine subgroups and interactions<br>(c) Explain how missing data were addressed<br>(d) *Cohort study* - If applicable, explain how loss to follow-up was addressed<br>*Case-control study* - If applicable, explain how matching of cases and controls was addressed<br>*Cross-sectional study* - If applicable, describe analytical methods taking account of sampling strategy<br>(e) Describe any sensitivity analyses	p6-7		
Data access and cleaning methods		..		RECORD 12.1: Authors should describe the extent to which the investigators had access to the database population used to create the study population.	p5

For peer review only

				RECORD 12.2: Authors should provide information on the data cleaning methods used in the study.	
Linkage		..		RECORD 12.3: State whether the study included person-level, institutional-level, or other data linkage across two or more databases. The methods of linkage and methods of linkage quality evaluation should be provided.	N/A
**Results**					
Participants	13	(a) Report the numbers of individuals at each stage of the study (*e.g.*, numbers potentially eligible, examined for eligibility, confirmed eligible, included in the study, completing follow-up, and analysed)<br>(b) Give reasons for non-participation at each stage.<br>(c) Consider use of a flow diagram	p8	RECORD 13.1: Describe in detail the selection of the persons included in the study (*i.e.,* study population selection) including filtering based on data quality, data availability and linkage. The selection of included persons can be described in the text and/or by means of the study flow diagram.	p8
Descriptive data	14	(a) Give characteristics of study participants (*e.g.*, demographic, clinical, social) and information on exposures and potential confounders<br>(b) Indicate the number of participants with missing data for each variable of interest<br>(c) *Cohort study* - summarise follow-up time (*e.g.*, average and total amount)	p8, Table 1		
Outcome data	15	*Cohort study* - Report numbers of outcome events or summary measures over time<br>*Case-control study* - Report numbers in each exposure	p9-10		

For peer review only

		category, or summary measures of exposure<br>*Cross-sectional study* - Report numbers of outcome events or summary measures			
Main results	16	(a) Give unadjusted estimates and, if applicable, confounder-adjusted estimates and their precision (e.g., 95% confidence interval). Make clear which confounders were adjusted for and why they were included<br>(b) Report category boundaries when continuous variables were categorized<br>(c) If relevant, consider translating estimates of relative risk into absolute risk for a meaningful time period	p9-14, Figures 1-3		
Other analyses	17	Report other analyses done—e.g., analyses of subgroups and interactions, and sensitivity analyses	p11		
**Discussion**					
Key results	18	Summarise key results with reference to study objectives	p15		
Limitations	19	Discuss limitations of the study, taking into account sources of potential bias or imprecision. Discuss both direction and magnitude of any potential bias	p17	RECORD 19.1: Discuss the implications of using data that were not created or collected to answer the specific research question(s). Include discussion of misclassification bias, unmeasured confounding, missing data, and changing eligibility over time, as they pertain to the study being reported.	p17
Interpretation	20	Give a cautious overall interpretation of results considering objectives,	p15, p17-18		

For peer review only

		limitations, multiplicity of analyses, results from similar studies, and other relevant evidence			
Generalisability	21	Discuss the generalisability (external validity) of the study results	p17		
**Other Information**					
Funding	22	Give the source of funding and the role of the funders for the present study and, if applicable, for the original study on which the present article is based	p18		
Accessibility of protocol, raw data, and programming code		..		RECORD 22.1: Authors should provide information on how to access any supplemental information such as the study protocol, raw data, or programming code.	p18

*Reference: Benchimol EI, Smeeth L, Guttmann A, Harron K, Moher D, Petersen I, Sørensen HT, von Elm E, Langan SM, the RECORD Working Committee. The REporting of studies Conducted using Observational Routinely-collected health Data (RECORD) Statement. *PLoS Medicine* 2015; in press.

*Checklist is protected under Creative Commons Attribution (CC BY) license.

For peer review only
